# Supplementary material for: Assessment of Schistosoma mansoni and soil-transmitted helminth infections and the diagnostic performance of the circulating cathodic antigen test among schoolchildren in Tulla district, Sidama region, Southern Ethiopia
Source: PLoS One. 2025 Dec 12;20(12):e0337919. doi: 10.1371/journal.pone.0337919 (PMC12700372; doi:10.1371/journal.pone.0337919)
Supplement: S1 Table — (DOCX) [file pone.0337919.s001.docx]

S1 Table: Questionnaire on socio-economic, environmental and hygienic practices

| **Part one: Information about the demography of the child** | | | | |
| --- | --- | --- | --- | --- |
| **S. N** | **Questions/attributes** | **Response** | **Code** | **Skip pattern** |
| Q101 | Age in years |  | | |
| Q102 | Sex | Male | 1 |  |
|  |  | Female | 2 |  |
| Q104 | Grade level | 1-4 | 1 |  |
|  |  | 5-8 | 2 |  |
| Q105 | School | Bushulo | 1 |  |
|  |  | Finchawa | 2 |  |
|  |  | Gemeto | 3 |  |
|  |  | Tulla | 4 |  |
| Q106 | Residence | Within 1 km | 1 |  |
|  |  | More than 1 km | 2 |  |
| **Part two: About Guardian** | | | | |
| Q201 | Educational status of father? | No formal education | 1 |  |
|  |  | Primary school | 2 |  |
|  |  | High school | 3 |  |
|  |  | College and above | 4 |  |
| Q202 | Educational status of mother/primary care giver? | No formal education | 1 |  |
|  |  | Primary school | 2 |  |
|  |  | High school | 3 |  |
|  |  | College and above | 4 |  |
| Q203 | Occupation of family | Civil Servant | 1 |  |
|  |  | Merchant | 2 |  |
|  |  | Farmer | 3 |  |
|  |  | Private (daily laborer) | 4 |  |
|  |  |  |  |  |

| **Part three: Information about water contact and sanitation** | | | | | |
| --- | --- | --- | --- | --- | --- |
| Q301 | Does the child involve in irrigation practice? | Yes | 1 | |  |
|  |  | No | 2 | |  |
| Q302 | Frequency of water contact | 1-3 days/week | 1 | |  |
|  |  | 4 and above days/week | 2 | |  |
| Q303 | Does the child have swimming habit? | Yes | 1 | |  |
|  |  | No | 2 | |  |
| Q304 | Does the child involve in fishing? | Yes | 1 | |  |
|  |  | No | 2 | |  |
| Q305 | Main water source | Pipe | 1 | |  |
|  |  | Well | 2 | |  |
|  |  | Lake | 3 | |  |
| Q306 | Does your child practice bathing in the lake? | Yes | 1 | |  |
|  |  | No | 2 | |  |
| Q307 | Does the child wash his/her hand before meal | Yes | | 1 |  |
|  |  | No | | 2 |  |
| Q308 | Latrine availability at home | Yes | | 1 |  |
|  |  | No | | 2 |  |
| Q309 | Does the child wash his/her hand after latrine | Yes | | 1 |  |
|  |  | No | | 2 |  |
| Q310 | Open field defecation | Yes | | 1 |  |
|  |  | No | | 2 |  |
| Q311 | Finger nail trimming | Yes | | 1 |  |
|  |  | No | | 2 |  |
| Q312 | Shoe wearing habit | Yes | | 1 |  |
|  |  | No | | 2 |  |
| **Part five: Information related to symptoms of the infection** | | | | | |
| Q501 | Does the child have any tummy pain? | Yes | | 1 |  |
|  |  | No | | 2 |  |
| Q502 | Does the child have diarrhea? | Yes | | 1 |  |
|  |  | No | | 2 |  |
| Q503 | If your answer for Q503, is “yes”, is it bloody? | Yes | | 1 |  |
|  |  | No | | 2 |  |

Adapted: from research journals (Ogweno *et al.*, 2023).
