# Supplementary material for: Assessment of Schistosoma mansoni and soil-transmitted helminth infections and the diagnostic performance of the circulating cathodic antigen test among schoolchildren in Tulla district, Sidama region, Southern Ethiopia
Source: PLoS One. 2025 Dec 12;20(12):e0337919. doi: 10.1371/journal.pone.0337919 (PMC12700372; doi:10.1371/journal.pone.0337919)
Supplement: Attachment — Submitted filename: PONE-D-25-23474 MAY 2025.pdf [file pone.0337919.s004.pdf]

PLOS ONE

Assessment of Schistosoma mansoni and Soil-transmitted Helminth Infections and the Diagnostic Performance of the Circulating Cathodic Antigen Test among Schoolchildren in Tulla District, Sidama Region, Southern Ethiopia.

--Manuscript Draft--

**Manuscript Number:**	PONE-D-25-23474
**Article Type:**	Research Article
**Full Title:**	Assessment of Schistosoma mansoni and Soil-transmitted Helminth Infections and the Diagnostic Performance of the Circulating Cathodic Antigen Test among Schoolchildren in Tulla District, Sidama Region, Southern Ethiopia.
**Short Title:**	Schistosoma mansoni Infection and Performance its antigen test
**Corresponding Author:**	Bamlaku Tadege Tadege, PhD<br>Hawassa University College of Medicine and Health Sciences<br>Hawassa, ETHIOPIA
**Keywords:**	Prevalence; Infection intensity; Schistosoma mansoni; Soil-transmitted helminth; Diagnostic performance; Kato-Katz technique; Point-of-care circulating cathodic antigen
**Abstract:**	Background: Soil-transmitted helminths and Schistosoma mansoni pose significant public health challenges in areas with poor sanitation. While Schistosoma mansoni infection leads to chronic illness and mortality, research on its intensity remains limited in the study area. Diagnosis traditionally relies on Kato-Katz method, though the POC-CCA urine test offers rapid alternative. This study examined prevalence, intensity, and risk factors of Schistosoma mansoni and Soil-transmitted helminth infections among schoolchildren while evaluating the diagnostic performance of POC-CCA test.<br>Methods: School-based cross-sectional study was conducted in Tulla district, Sidama region, among 477 students (April–June 2024). Socio-demographic and risk factor data were collected via a KOBO Collect questionnaire. Stool samples were analyzed using Kato-Katz, and urine samples were tested using POC-CCA test. Data were processed in SPSS v26 using logistic regression, with $p<0.05$ considered statistically significant. Diagnostic accuracy was evaluated using 2×2 contingency tables and McNemar's chi-square test.<br>Result: The overall prevalence of Schistosoma mansoni was 36.5%, (20.1% by Kato-Katz, and 34.4% by POC-CCA). The prevalence of soil-transmitted helminths was 48.8%. The leading parasite was Ascaris lumbricoides (34%), followed by Trichuris trichuria (8.2%) and hookworm (5.7%). Other helminths included Taenia species 1.3%, Hymenolepis nana 0.6%, and Enterobius vermicularis 0.2%. Infection intensity of Schistosoma mansoni was classified as light 34.4%, moderate 56.2%, and heavy 9.4%. Involvement in irrigation, swimming, bathing, and school location were significantly associated with Schistosoma mansoni infection. school location, educational status, lack of latrine, hand washing hands practice and fingernail trimming practice were significantly associated with STH infections. The POC-CCA rapid test had significantly higher sensitivity of 89.6% (McNemar test $\chi^2 m = 52.3$, $p < 0.001$) compared to Kato-Katz technique.<br>Conclusions: Moderate Schistosoma mansoni and soil-transmitted helminth infection and poor hygiene practice underscore the necessity for additional control measures. Incorporate POC-CCA testing alongside Kato-Katz method could optimize diagnostic accuracy and improve disease management outcomes.
**Order of Authors:**	Bamlaku Tadege Tadege, PhD
	Fitsum Getachew Getachew, MSC
	Demissie Assegu Fenta, MSC
	Biniyam Kijineh Kijineh, MSC
**Additional Information:**	
**Question**	**Response**

**Financial Disclosure**<br><br>Enter a financial disclosure statement that describes the sources of funding for the work included in this submission. Review the submission guidelines for detailed requirements. View published research articles from *PLOS ONE* for specific examples.<br><br>This statement is required for submission and **will appear in the published article** if the submission is accepted. Please make sure it is accurate.<br><br>**Funded studies**<br>Enter a statement with the following details:<br>• Initials of the authors who received each award<br>• Grant numbers awarded to each author<br>• The full name of each funder<br>• URL of each funder website<br>• Did the sponsors or funders play any role in the study design, data collection and analysis, decision to publish, or preparation of the manuscript?<br><br>Did you receive funding for this work?	The author(s) received no specific funding for this work.
**Competing Interests**<br><br>Use the instructions below to enter a competing interest statement for this submission. On behalf of all authors, disclose any competing interests that could be perceived to bias this work—acknowledging all financial support and any other relevant financial or non-financial competing interests.<br><br>This statement is required for submission and **will appear in the published article** if the submission is accepted. Please make sure it is accurate and that any funding sources listed in your Funding Information later in the submission form are also declared in your Financial Disclosure statement.	There is no any competing interest for this research. All authors dead and agreed to submit this article to PLoS ONE journal. We read also the journal guideline

View published research articles from *PLOS ONE* for specific examples.<br><br>**NO authors have competing interests**<br><br>Enter: *The authors have declared that no competing interests exist.*<br><br>**Authors with competing interests**<br><br>Enter competing interest details beginning with this statement:<br><br>*I have read the journal's policy and the authors of this manuscript have the following competing interests: [insert competing interests here]*<br><br>* typeset	
**Ethics Statement**<br><br>Enter an ethics statement for this submission. This statement is required if the study involved:<br><br>• Human participants<br>• Human specimens or tissue<br>• Vertebrate animals or cephalopods<br>• Vertebrate embryos or tissues<br>• Field research<br><br>Write "N/A" if the submission does not require an ethics statement.<br><br>General guidance is provided below. Consult the submission guidelines for detailed instructions. **Make sure that all information entered here is included in the Methods section of the manuscript.**	Ethical clearance was obtained from Hawassa University, College of Medicine, and other Health Sciences Internal Review Board (IRB) with a reference number: IRB/172/16. A letter of support from Hawassa University School of Medical Laboratory was provided to the Sidama Regional State Health Bureau, as well as to the health and education offices in Tulla district and the directors of the selected schools. Participants in the study were informed about its purpose and objectives and invited to participate voluntarily. Written informed consent was obtained from the parents or guardians of the participating children, while assent was secured from children aged 12 and above. After obtaining permission, the questionnaire was administered to the parents (guardians) and children aged 12 and above. Stool and urine samples were collected from the volunteer children. To ensure confidentiality, participant data was recorded using codes instead of names. All positive cases were referred to the nearest health institution for appropriate treatment according to national guidelines.

**Format for specific study types**<br><br>**Human Subject Research (involving human participants and/or tissue)**<br>• Give the name of the institutional review board or ethics committee that approved the study<br>• Include the approval number and/or a statement indicating approval of this research<br>• Indicate the form of consent obtained (written/oral) or the reason that consent was not obtained (e.g. the data were analyzed anonymously)<br><br>**Animal Research (involving vertebrate animals, embryos or tissues)**<br>• Provide the name of the Institutional Animal Care and Use Committee (IACUC) or other relevant ethics board that reviewed the study protocol, and indicate whether they approved this research or granted a formal waiver of ethical approval<br>• Include an approval number if one was obtained<br>• If the study involved *non-human primates*, add *additional details* about animal welfare and steps taken to ameliorate suffering<br>• If anesthesia, euthanasia, or any kind of animal sacrifice is part of the study, include briefly which substances and/or methods were applied<br><br>**Field Research**<br><br>Include the following details if this study involves the collection of plant, animal, or other materials from a natural setting:<br>• Field permit number<br>• Name of the institution or relevant body that granted permission	
**Data Availability**<br><br>Authors are required to make all data underlying the findings described fully available, without restriction, and from the time of publication. PLOS allows rare exceptions to address legal and ethical concerns. See the PLOS Data Policy and FAQ for detailed information.	Yes - all data are fully available without restriction

A Data Availability Statement describing where the data can be found is required at submission. Your answers to this question constitute the Data Availability Statement and **will be published in the article**, if accepted.<br><br>**Important:** Stating 'data available on request from the author' is not sufficient. If your data are only available upon request, select 'No' for the first question and explain your exceptional situation in the text box.<br><br>Do the authors confirm that all data underlying the findings described in their manuscript are fully available without restriction?	
**Describe where the data may be found in full sentences. If you are copying our sample text, replace any instances of XXX with the appropriate details.**<br><br>• If the data are **held or will be held in a public repository**, include URLs, accession numbers or DOIs. If this information will only be available after acceptance, indicate this by ticking the box below. For example: *All XXX files are available from the XXX database (accession number(s) XXX, XXX.*).<br>• If the data are all contained **within the manuscript and/or Supporting Information files**, enter the following: *All relevant data are within the manuscript and its Supporting Information files.*<br>• If neither of these applies but you are able to provide **details of access elsewhere**, with or without limitations, please do so. For example:<br><br>*Data cannot be shared publicly because of [XXX]. Data are available from the XXX Institutional Data Access / Ethics Committee (contact via XXX) for researchers who meet the criteria for access to confidential data.*<br><br>*The data underlying the results presented in the study are available from (include the name of the third party*	data are all contained within the manuscript and/or Supporting Information files, enter the following: All relevant data are within the manuscript and its Supporting Information files.

and contact information or URL).<br>• This text is appropriate if the data are owned by a third party and authors do not have permission to share the data.<br><br>* typeset	
Additional data availability information:	

Manuscript

Click here to access/download;Manuscript;Manuscript FG-NEW (3) for submission.docx

Assessment of *Schistosoma mansoni* and Soil-transmitted Helminth Infections and the Diagnostic Performance of the Circulating Cathodic Antigen Test among Schoolchildren in Tulla District, Sidama Region, Southern Ethiopia.

Short title: *Schistosoma mansoni* Infection and Performance its antigen test

Fitsum Getachew[1,2*], Demissie Assegu[1], Biniyam Kijineh[3] and Bamlaku Tadege[1*]

[1] School of Medical Laboratory Sciences, Hawassa University, Hawassa, Ethiopia.

[2] School of Medical Laboratory Sciences, Wolaita Sodo University, Wolaita, Ethiopia.

[3] School of Medical Laboratory Sciences Wachemo University Ethiopia, Hossana, Ethiopia.

*E-mail of corresponding author: abelbamlaku@yahoo.com

Consent for publication

All authors have read the manuscript and consented to submit the manuscript

Competing interests

All authors declare that they have no competing interests.

Authors' contributions

FG: conceptualization, data curation, formal analysis, investigation, methodology, writing – original draft; DA & BK: writing – review & editing, BT conceptualization, supervision, writing – review & editing, data curation and formal analysis.

Authors' addresses

fitsumgetachew6@gmail.com (FG)

abelbamlaku@yahoo.com (BT)

demisissieasegu@yahoo.com (DA)

kijinehb@gmail.com (BK)

Funding

This study was funded by Hawassa University

Abstract

Background: Soil-transmitted helminths and *Schistosoma mansoni* pose significant public health challenges in areas with poor sanitation. While *Schistosoma mansoni* infection leads to chronic illness and mortality, research on its intensity remains limited in the study area. Diagnosis traditionally relies on Kato-Katz method, though the POC-CCA urine test offers rapid alternative. This study examined prevalence, intensity, and risk factors of *Schistosoma mansoni* and Soil-transmitted helminth infections among schoolchildren while evaluating the diagnostic performance of POC-CCA test.

Methods: School-based cross-sectional study was conducted in Tulla district, Sidama region, among 477 students (April–June 2024). Socio-demographic and risk factor data were collected via a KOBO Collect questionnaire. Stool samples were analyzed using Kato-Katz, and urine samples were tested using POC-CCA test. Data were processed in SPSS v26 using logistic regression, with $p<0.05$ considered statistically significant. Diagnostic accuracy was evaluated using 2×2 contingency tables and McNemar's chi-square test.

Result: The overall prevalence of *Schistosoma mansoni* was 36.5%, (20.1% by Kato-Katz, and 34.4% by POC-CCA). The prevalence of soil-transmitted helminths was 48.8%. The leading parasite was *Ascaris lumbricoides (*34%)*,* followed by *Trichuris trichuria* (8.2%) and hookworm (5.7%). Other helminths included Taenia species 1.3%, *Hymenolepis nana* 0.6%, and *Enterobius vermicularis* 0.2%. Infection intensity of Schistosoma mansoni was classified as light 34.4%, moderate 56.2%, and heavy 9.4%. Involvement in irrigation, swimming, bathing, and school location were significantly associated with *Schistosoma mansoni* infection. school location, educational status, lack of latrine, hand washing hands practice and fingernail trimming practice were significantly associated with STH infections. The POC-CCA rapid test had significantly

higher sensitivity of 89.6% (McNemar test χ^2m = 52.3, $p < 0.001$) compared to Kato-Katz technique.

Conclusions: Moderate Schistosoma mansoni and soil-transmitted helminth infection and poor hygiene practice underscore the necessity for additional control measures. Incorporate POC-CCA testing alongside Kato-Katz method could optimize diagnostic accuracy and improve disease management outcomes.

Keywords: Prevalence, Infection intensity, *Schistosoma mansoni,* Soil-transmitted helminth, Diagnostic performance, Kato-Katz technique, Point-of-care circulating cathodic antigen.

Introduction

Background

Schistosomiasis and soil-transmitted helminthiases (STH) are worm infections in the group of neglected tropical diseases (NTDs) (1). Schistosomiasis is a water-based, vector-borne parasite transmitted indirectly through freshwater snails (2). People who live in areas with inadequate water and sanitation systems are susceptible to this most common and deadly infection (3). Globally, schistosomiasis is recognized as endemic in 78 low and middle-income countries in tropical and subtropical areas, estimated to affect >251 million people (4). Furthermore, 243 million people require preventative chemotherapy, including 111 million school-age children, of which 226 million are in Africa (5). It causes significant morbidity, with an estimated 1.4-3.3 million disability-adjusted life years (DALYs) lost annually due to its health impacts (6).

STHs are intestinal parasitic worms transmitted to humans through fecally contaminated soil (7). The main STHs that affect humans are Ascaris lumbricoides (A. lumbricoides), Trichuris trichuria (T. trichuria), Necator americanus, and Ancylostoma duodenale (8). These parasites cause gastrointestinal infections and are most commonly found in tropical and subtropical regions, particularly impacting impoverished nations where schoolchildren are especially vulnerable (9).

Globally, an estimated 1.5 billion people are infected with STHs, including 654 million school-aged children living in areas where these parasites are prevalent (10). The Burden of Schistosoma and STHs leads to various health issues, including gastrointestinal bleeding, nutrient malabsorption, loss of appetite, anemia due to the depletion of iron and other essential proteins, growth retardation, impaired cognitive development, school absenteeism, and

disability-adjusted life-years lost (11–13). To control Schistosoma and STH-attributable morbidity, WHO recommends three interventions, including periodical administration of anthelmintic drugs to kill the worms, health education and improved water, hygiene and sanitation (WASH) to prevent re-infection. Today, mass drug administration (MDA) programs remain the mainstream strategy. During these programs, a single oral dose of albendazole (400 mg) or mebendazole (500 mg) for STH and praziquantel for Schistosoma are periodically administered to the at-risk groups (14). Although these deworming programs have been successful in reducing global Schistosoma and STH-attributable morbidity, re-infection perpetuates in the absence of improved WASH and health education (15).

Intestinal Schistosoma is diagnosed by microscopic detection of parasite eggs in stool using the Kato-Katz (KK) technique, which is widely used in epidemiological surveys (16). However, its diagnosis is challenging in low-endemic areas where prevalence and worm burden are reduced, due to factors like uneven egg distribution in stool samples, daily variations in egg excretion, and random distribution effects (17,18). On the other hand, the point-of-care (POC) cassette test is a relatively new diagnostic tool that detects circulating cathodic antigens (CCA) in urine samples, offering higher sensitivity and operational benefits (18,19). The study found that POC-CCA was more sensitive (86% vs. 62%) but less specific (72% vs. ~100%) compared to multiple KK smears from a single stool sample. Despite this, the study noted that only 1% of POC-CCA tests in non-endemic areas were false positives, indicating that the specificity of POC-CCA might be underestimated. Multivariable modeling also suggested that POC-CCA was significantly more sensitive than KK for detecting low infection intensities (<100 eggs/gram of stool) (20). However, before the POC-CCA assay can be fully utilized for monitoring and evaluating control programs, it is essential to assess its performance in endemic settings (21).

Therefore, this study aimed to evaluate the prevalence of *S. mansoni* and STH infections and intensity of *S. mansoni* in elementary school-aged children as well as the associated risk factors to evaluate ongoing controlling intervention. Additionally, it sought to assess the performance of the POC-CCA test compared to the Kato-Katz method for diagnosing *S. mansoni*.

Methods

Study area

The study was conducted in four elementary schools in Tulla district, Sidama region in Southern Ethiopia. The district is situated along the shore of Lake Hawassa and the Tikur Wuha River, approximately 289 kilometers south of Addis Ababa, the capital city of Ethiopia. The altitude is 1800 meters above sea level with an annual rainfall of 1123 mm and temperatures ranging from 13 to 27°C. Lake Hawassa is the primary water source for the residents of Hawella Tulla, catering to their domestic, agricultural, fishing, and other needs. According to ECSA, the district has a total population of 129,507, comprising 65,018 males and 64,499 females. The district has various social service institutions, including 27 health facilities and 69 schools. The health facilities consist of 1 primary hospital, 6 health centers, 18 rural health posts, 2 urban health posts, and 1 non-governmental health center. In terms of education, the district includes 21 primary schools, 5 secondary schools, 6 non-governmental primary schools, and 37 non-governmental kindergarten schools (Figure 1).

Study design, Study population, inclusion and exclusion criteria

A school-based cross-sectional study was conducted between April and June 2024 among four governmental elementary schoolchildren in the Tulla district. The source population was all schoolchildren in Tulla district, Sidama region, southern Ethiopia. The study population included schoolchildren who were enrolled in the selected schools during the study period and met the

established inclusion criteria (who had both parental or guardian informed consent and their assent to participate) and exclusion criteria (who had received anti-helminthic treatment within one month before the recruitment period, or who were unable to provide stool and urine samples were excluded from the study).

To determine the sample size, a single population proportion formula was used based on the following assumptions: a prevalence of *S. mansoni* of 31% (22), with a 5% margin of error and a 95% confidence level. As a result, the calculated sample size was 329. Considering the design or cluster effect between schools, the determined number was multiplied by 1.5, and then 5% was added for the non-response rate, giving the final sample size of 484.

To recruit participants for this study, we utilized a purposive sampling technique. In the initial stage, Bushulo and Finchawa, located near the coasts of Lake Hawassa in the Tulla sub-city, were selected alongside two primary schools farther from the lake, Gemeto and Tulla, with student populations of 1,820, 686, 2,050, and 1,439, respectively. Then, the predetermined sample size of 484 was proportionally allocated to the schools, resulting in 147 participants from Bushulo, 55 from Finchawa, 116 from Gemeto, and 166 from Tulla. The students' registration lists served as the sampling frame, and a systematic random sampling technique was employed to select the study subjects from each school. The k-value (sampling interval) was determined by dividing the total number of study participants in each school by the number of students to be selected. The students were then systematically selected at regular intervals (every 12th student), stratified by age (5–19 years) and grade levels (1–4 and 5–8) until the predetermined sample size was achieved. In cases where the selected student was absent from school, three repeated visits were made. If a student could not be found after three visits, they would be considered non-respondents.

The dependent variables were prevalence, infection intensity, and diagnostic performance of *Schistosoma mansoni* infection. The independent variables include socio-demographic characteristics (gender, age, grade level, school attended, educational status of the father and mother, and family occupation). The distance of the home from the lake and practice-related factors were also explored, focusing on habits such as swimming, fishing, shoe-wearing, washing clothes, hand washing before meal and after latrine, open-field defecation, trimming finger nail, bathing, and involvement in irrigation activities. Environmental factors considered included the availability of a latrine at home and the frequency of water contact. Clinical symptoms of the disease were also assessed among study participants.

The data was collected using a structured questionnaire. Two trained nurses interviewed the parents or guardians of the children through home-to-home visits, utilizing the KOBO Collect digital tool. A senior expert and the principal investigator supervised the data collection process, and regular meetings were held to address any challenges and ensure adherence to established protocols and standards.

Fecal and urine samples were collected in a screw-capped, dry, and clean container pre-labeled with the participant's unique identification number. The collected stool samples were transported within an hour of collection in suitable cool boxes at temperatures between 4°C and 6°C for subsequent examination. Samples from Gemeto and Tulla Elementary School were transported to Tulla Hospital, while samples from Bushulo and Finchawa Elementary School were transported to the Bushulo Mother and Child Health Specialty Center. Sample collection and transportation quality were supervised by the investigator to ensure adherence to procedures and the accuracy of the data results.

Stool sample processing using the Kato-Katz technique

The stool samples were examined using the KK technique to detect parasite eggs. A portion of the sample was processed using a calibrated template holding 41.7 mg of stool. Double slides were prepared for each stool sample. The number of eggs was counted from the double KK microscopic slides, and the average count was converted to eggs per gram of feces (EPG) using a correction factor of 24. Based on WHO cut-off values, the infection intensity was categorized as light (EPG 1-99), moderate (EPG 100-399), or heavy (EPG >400) (23).

Urine sample processing using POC-CCA rapid test

Each urine sample was processed immediately at the schools using the POC-CCA test, following the manufacturer's instructions (24). This on-site processing allowed for prompt results and minimized potential delays or degradation of the samples. 100μL of urine was transferred to the circular well of the test cassette and allowed to absorb completely into the specimen pad. The test results were read after 20 minutes of assay development. In cases of discrepancies, the tests were re-examined to ensure accuracy. The POC-CCA tests were scored as negative or positive based on the formation and intensity of the control band. Tests were considered invalid if an internal control band did not appear or if results were not read after more than 25 minutes. In such cases, samples were re-run with a new cassette and scored as necessary.

Data quality assurance

The questionnaire was initially developed in English and then translated into Amharic and Sidamu Afoo, the local language of the participants. It was then translated back into English to ensure consistency. A pretest was conducted on 5% of the sample size at Tullo Primary School, which was not part of the selected schools for the final study. The questionnaire was subsequently modified based on the findings from the pretest. The completeness of data

collection was overseen daily by the assigned supervisor. Schoolchildren were oriented to bring enough stool and urine samples. The senior laboratory technologist provided refresher training to laboratory professionals. Standard operating procedures (SOP) for pre-analytical, analytical, and post-analytical procedures were implemented throughout sample analysis. To guarantee quality control as an internal quality control measure, 10% of the slides were randomly selected and re-examined by a senior laboratory technologist at Tulla Hospital and Bushulo Mother and Child Health Specialty Centre and by another experienced technologist at HUCSH as part of an external laboratory evaluation. Both stool and urine samples were collected between 09:00-12:00 a.m. and 03:00-05:00 a.m. local time each day of the survey.

Data processing and analysis

Data was entered into the KOBO Collect digital tool and analyzed using SPSS version 26 software. The prevalence of *S. mansoni* and STH infection was presented as a percentage, while infection intensity was classified as low, moderate, or heavy according to WHO guidelines (4). The mean egg count was reported as a geometric mean. A logistic regression model was employed to identify potential determinants of *S. mansoni* and STH infections and assess the strength of associations. Crude odds ratios (COR) from bivariate analysis were calculated to identify significant variables associated with the outcome. To control for confounding effects, factors with a p-value ≤ 0.25 in bivariate analysis were included in multivariable logistic regression, allowing for the determination of adjusted odds ratios (AOR). A p-value < 0.05 was considered statistically significant. The diagnostic performance of the POC-CCA was assessed by calculating sensitivity, specificity, and other relevant metrics, with these tests regarded as reference standards (25). Kappa statistics was utilized to quantify agreement between the two diagnostic methods, and the concordance rate was calculated as the proportion of cases yielding

identical results. McNemar's chi-square test was applied to analyze paired proportions of positive and negative results to investigate detection rate differences further.

Ethical consideration

Ethical clearance was obtained from Hawassa University, College of Medicine, and other Health Sciences Internal Review Board (IRB) with a reference number: IRB/172/16. A letter of support from Hawassa University School of Medical Laboratory was provided to the Sidama Regional State Health Bureau, as well as to the health and education offices in Tulla district and the directors of the selected schools. Participants in the study were informed about its purpose and objectives and invited to participate voluntarily. Written informed consent was obtained from the parents or guardians of the participating children, while assent was secured from children aged 12 and above. After obtaining permission, the questionnaire was administered to the parents (guardians) and children aged 12 and above. Stool and urine samples were collected from the volunteer children. To ensure confidentiality, participant data was recorded using codes instead of names. All positive cases were referred to the nearest health institution for appropriate treatment according to national guidelines.

Result

Socio-demographic characteristics of the study participants

A total of 484 students were invited to participate in the study, of which 477 met the inclusion criteria and agreed to take part, yielding a response rate of 98.5%. The study sample included 208 (43.6%) males and 269 (56.4%) females. Participants' ages ranged from 5 to 19 years, with a mean age of 11.94 years and a standard deviation of 2.76 years. Most of the study participants (62.7%) were 10-14 years old. The majority of students reside within 1 km of a lake (66.5%) and are under the care of their private (daily laborer) parents or guardians (41.7%). The students were selected from 4 primary schools: Bushulo 143 (30%), Finchawa 53 (11.1%), Gemeto 116 (24.3%), and Tulla 165 (34.6%) (Table 2).

Prevalence of *Schistosoma mansoni* infection

The combined results of the 2 diagnostic methods showed an overall prevalence of *S. mansoni* to be 36.5%, with 20.1% (96/477) detected using the KK technique and 34.4% (164/477) detected using the POC-CCA test. The highest prevalence of *S. mansoni* infection, 30.5%, was observed among students attending schools near the lake (Bushulo and Finchawa). Overall, the prevalence of *S. mansoni* infection was highest among students aged 10 - 14 years (Table 2).

Infection intensity of *S. mansoni* infection

Out of 174 *S. mansoni* infected study participants, 96 tested positive by the KK technique, which uses a standard amount of stool and enables the quantification of ova per gram of stool. The minimum and maximum eggs per gram of stool were 24 and 672, respectively. Likewise, the arithmetic and geometric mean values were 189.75 and 139.39, respectively. According to WHO classification, among 96 students, 33 (34.4%) had light intensity, 54 (56.2%) had moderate intensity, and 9 (9.4%) had a heavy intensity of *S. mansoni* infection (Figure 2).

As indicated in Table 1, moderate infection with *S. mansoni* among males accounted for 53.9%, followed by 34.6% light infection and 11.5% heavy infection. Similarly, most infections with *S. mansoni* among the age group 15-19 years were of moderate intensity 72.7%, followed by light 18.2% and heavy intensity 9.1%. When compared school-wise, more than half of infections with *S. mansoni* among respondents from Bushulo primary school were of moderate intensity 61.5%, followed by light 27% and heavy 11.5%. However, most infections among respondents from Tulla primary school were of light intensity 64.3%, followed by moderate intensity 35.7%.

Table 1: Gender, age, and school-based variation in S. mansoni infection intensity among schoolchildren in Tulla District 2024 (n = 477).

**Variable**	**N**	**Intensity of infection n (%)**		
		**Light**	**Moderate**	**Heavy**
**Gender**				
Male	52	18(34.6)	28(53.9)	6(11.5)
Female	44	15(34.1)	26(59.1)	3(6.8)
**Age**				
5-9	7	2(28.6)	5(71.4)	0(0)
10-14	67	27(40.3)	33(49.25)	7(10.45)
15-19	22	4(18.2)	16(72.7)	2(9.1)
**Schoolchildren**				
Bushulo	52	14(27)	32(61.5)	6(11.5)
Finchawa	13	4(31)	7(53.8)	2(15.2)
Gemeto	17	6(35.3)	10(58.8)	1(5.9)
Tulla	14	9(64.3)	5(35.7)	0(0)

Key - N: refers to the total number of infected; whereas, n: refers to the number of infected according to intensity classes.

Potential associated risk factors of *Schistosoma mansoni* infections.

In the bivariate logistic regression analysis, activities such as washing clothes 13% (62/477), bathing 31.7% (151/477), swimming 32.9% (157/477), fishing 11.9% (57/477), and engaging in irrigation activities 24.9% (119/477), were identified as candidate factors of *S. mansoni* infection with a p-value ≤ 0.25. Socio-demographic factors such as gender, age, school (Bushulo), educational status of the father (primary school), educational status of the mother (primary school), and distance of residential area from the lake were also candidate predictors of *S. mansoni* infection (Table 2).

Multivariate logistic regression analysis was applied to avoid confounding factors and only students engaged in irrigation were 2.4 times more likely to be infected than those not involved (AOR = 2.43; 95% CI: 1.38-4.29, P = 0.002), schoolchildren with swimming habits exhibited a 2.4-fold increase in the likelihood of infection (AOR = 2.41; 95% CI: 1.43-4.34, P = 0.001) and participants who bathed in the lake were 1.8 times more likely to be infected than those who did not engage in this activity (AOR = 1.80; 95% CI: 1.05-3.12, P = 0.033). A school-wide analysis revealed that students attending Bushulo primary school were 4.7 times more likely to contract *S. mansoni* infection compared to their peers at Tulla primary school (AOR = 4.68; 95% CI: 1.87-11.74, P = 0.001) (Table 3).

Prevalence of Soil-transmitted helminth infections

Aside from *S. mansoni*, the prevalence of intestinal helminths among the schoolchildren was 233 (48.8%). The most common STH was *Ascaris lumbricoides* 162 (34%), followed by *Trichuris trichuria* 39 (8.2%) and Hookworm 27 (5.7%). Other helminth parasites included Taenia species 6 (1.3%), *Hymenolepis nana* 3 (0.6%), and *Enterobius vermicularis* 1 (0.2%) (Figure 3).

Potential associated risk factors of Soil-transmitted helminth (STH) infections.

In the bivariate logistic regression analysis, both hygiene-related practices and socio-demographic factors were identified as potential risk factors for STH infections. Household latrine availability (61%, 105/477), handwashing after latrine use (52.8%, 227/477), fingernail trimming (56.4%, 194/477), and handwashing before meals (53.4%, 196/477) were all associated with STH infections, with a p-value ≤ 0.25. Additionally, socio-demographic factors, including attending Tulla Elementary School, grade level, father's educational status, and family occupation (farmer), emerged as candidate predictors (Table 3).

In the multivariate logistic regression analysis, several socio-demographic and behavioral factors were found to significantly influence the prevalence of STH infections among schoolchildren. Students from Tulla Elementary School had 0.47 times lower odds of STH infection compared to their counterparts at Bushulo Elementary School (AOR = 0.469; 95% CI: 0.256-0.861, P = 0.015). Additionally, the educational background of parents played a crucial role, as children whose fathers had no formal education were found to be 4.2 times more likely to be infected with STH compared to those whose fathers had attained a college education or higher (AOR = 4.161; 95% CI: 1.276-13.570, P = 0.018). The presence of latrines at home also significantly influenced infection rates, with children having access to latrines exhibiting 0.63 times lower odds of infection than those without such facilities (AOR = 0.626; 95% CI: 0.393-0.996, P = 0.048). Furthermore, hygiene practices emerged as critical determinants, as children who washed their hands after using the latrine had a remarkable 0.2 times lower odds of contracting STH infections compared to those who did not (AOR = 0.199; 95% CI: 0.072-0.551, P = 0.002). Similarly, regular nail trimming was associated with a significant reduction in infection risk, with children

who trimmed their nails having 0.4 times lower odds of STH infection than those who did not (AOR = 0.413; 95% CI: 0.250-0.682, P = 0.001) (Table 3).

Detection and performance evaluation of the POC-CCA test

Out of 477 samples examined, the POC-CCA rapid test identified 86 (18.0%) true positives, 303 (63.5%) true negatives, and 10 (2.1%) discrepancies (false negatives) compared to the KK technique. Using KK as the gold standard or ‘reference test’ (25), the POC-CCA sensitivity was 89.6% (95% CI: 83.5-95.7) and specificity 79.6% (95% CI: 75.6-83.6) (Table 4).

Table 4: Performance of POC-CCA test using Kato-Katz as a gold standard among schoolchildren in Tulla district 2024 (n = 477).

Method	Result	KK (Gold standard) Pos	KK (Gold standard) Neg	Sensitivity % (95% CI)	Specificity % (95% CI)	NPV % (95% CI)	PPV % (95% CI)	LR^- % (95% CI)	LR^+ % (95% CI)
POC-CCA	Pos	86	78	89.6 (80-99.2)	79.6 (73.9-85.3)	96.8 (90.7-100)	52.4 (45.2-59.6)	0.131 (0.057-0.197)	4.39 (3.93-5.10)
	Neg	10	303						

Key - KK: Kato-Katz POC-CCA: Point of Care Circulating Cathodic Antigen, NPV: Negative Predictive value, PPV: Positive Predictive value, NLR^-: Negative Likely hood ratio, LR^+: Positive Likely hood ratio, Pos: Positive, Neg: Negative.

The agreement between the two diagnostic methods was quantified using Cohen’s Kappa statistics which yielded a kappa value of 0.55. Furthermore, the overall concordance rate between the KK and POC-CCA was 81.5%. However, to assess the statistical significance of the

differences in results between the two tests, we employed the McNemar Chi-square test. The result revealed a value of 52.3.

Discussion

This study aimed to assess the prevalence, intensity, and associated risk factors of *S. mansoni* and to evaluate the diagnostic performance of the POC-CCA rapid test among schoolchildren in the Tulla district of the Sidama region in southern Ethiopia. The prevalence of *S. mansoni* infection was 20.1% (95% CI: 16.8-23.7) using the KK technique. This indicates the need for biannual deworming to schoolchildren in the study area with a moderate prevalence (≥ 10% but < 50%) according to WHO guidelines for preventive chemotherapy, providing a baseline for designing health intervention strategies (4). This result aligns with previous studies from Jimma 20.2% (26), Kersa 19.9% (27) and Mekelle 23.9% (28) in Ethiopia. Furthermore, the prevalence reported in this study is consistent with findings from Egypt 19.1% (29) and Angola 21.2% (30). However, it was higher than rates observed in Jiga at 15.2% (31), Guangua 12.6% (32), and Wondo districts 11.4% (33).

The prevalence in this study is lower than findings from Lake Tana in northwest Ethiopia, which reported 34.9% (34), as well as from Jimma town, an urban area in southwest Ethiopia, with a prevalence of 28.7% (35).

Additionally, the determined prevalence is lower than rates recorded in Maksegnit, Debark, Sanja, and Chuahit districts in northwest Ethiopia at 33.5% (36), Abbey and Didessa valleys 53.9% (37), and the Wolaita zone in southern Ethiopia 81.3% (38). Furthermore, the prevalence noted in this study is also lower than reports from Kenya 53.7% (39), Ituri province in

the northeastern Democratic Republic of Congo 90.2% (40), and northwestern Tanzania 90.6% (41). The differences in the prevalence of *S. mansoni* infection in different regions may be attributed to variations in socio-demographic factors, exposure to water sources, rates of reinfection, diagnostic methods employed, and the expertise of the professionals conducting the examinations.

The moderate infection intensity reported in this study is consistent with findings from various regions in Ethiopia (37,42) and in Tanzania (41). This difference might be due to repeated exposure of schoolchildren to water bodies infested with the infective stage. The intensity of *S. mansoni* infection was found to be slightly higher in male students compared to female students, which is consistent with findings from Kemissie and Wondo Genet (42) and Jimma Town (43) in Ethiopia. The observed difference in proportion might be linked to male students' greater exposure to outdoor activities like fishing, swimming, and irrigation compared to female students.

A significant association was found between *S. mansoni* infection and schoolchildren who engaged in swimming and bathing activities in the lake, with infection risks being 2.4 times higher for swimming and 1.8 times higher for bathing than those who did not partake in these habits. This association between *S. mansoni* infection and swimming habits among schoolchildren aligns with previous studies (22,32) and is further supported by studies linking bathing habits to infection (34).

Additionally, a strong association was noted between *S. mansoni* infection and participation in irrigation activities, with students involved in irrigation being 2.4 times more likely to be infected than those not engaged in such activities, consistent with prior findings (22,32). Notable differences in infection rates were observed among students across the various schools in the

study areas. Children attending Bushulo Primary School were 4.7 times more likely to be infected with Schistosoma than their peers at Tulla Primary School. This finding aligns with previous studies (22,34). This might be associated with students' residence distance variation from lakes and other water bodies.

The study area displayed a moderate prevalence of STH infections among schoolchildren despite them being part of the deworming target group. Deworming programs have successfully decreased the global morbidity attributed to STHs (44), but the risk of re-infection persists in the absence of improved water, sanitation, and hygiene (WASH) practices and health education (45). A systematic review and meta-analysis estimated that three months after deworming, the prevalence of *Ascaris lumbricoides*, *Trichuris trichuria*, and *hookworms* reached 26%, 36%, and 30% of their pre-treatment levels, respectively. By one-year post-treatment, the prevalence rates approached their pre-treatment levels again, with *A. lumbricoides* at 94%, T. trichuria at 82%, and *hookworm* infections at 57% (46). The occurrence of re-infection highlights the importance of a comprehensive approach to controlling STH. This is especially crucial given the increasing interest in ultimately halting STH transmission in specific geographic regions (47). The current WHO guidelines recommend the evaluation of any STH infections in school-age children after five to six years of periodic chemotherapy using the standard Kato-Katz thick smear (48).

The findings of this study also provide critical insights into the socio-economic and environmental determinants associated with STH infections among school children. This research identified several significant factors linked to the prevalence of STH, including the lack of a latrine at home, lack of hand-washing practice following latrine use, educational status of fathers, lack of proper fingernail trimming practices, and certain school attendances. The absence of a latrine at home emerged as a prominent risk factor for STH infection. This finding aligns

with existing literature that emphasizes the role of sanitation facilities in preventing worm infections (49–51). Households without adequate sanitation are often compelled to resort to open defecation, which increases the likelihood of soil contamination with helminth eggs. Hand hygiene practices, particularly the failure to wash hands after using a latrine, were also significantly associated with STH prevalence, which aligns with previous literature (52–54). This underscores the critical role of handwashing in interrupting these parasites' transmission cycle (55). Educational interventions aimed at promoting proper hand hygiene, especially among children, could serve as a vital strategy in reducing STH infections.

Moreover, the educational status of was found to correlate significantly with STH prevalence among children, which aligns with previous studies (56,57). This highlights the importance of targeting educational programs not only for children but also for parents to foster a supportive environment for health-promoting behaviors. Certain school attendance was also significantly associated with STH infection, in line with previous studies (58,59). This might be due to differences in sanitation facilities, hygiene practices, socio-economic status, and health education among students, which can significantly influence the transmission dynamics of STH infections in certain schools.

Finally, our study revealed that the practice of not trimming fingernails was significantly associated with STH infection, which aligns with previous studies (50,60–62). Since, long fingernails can harbor soil and pathogens, serving as a reservoir for helminth eggs that can be ingested or transmitted through hand-to-mouth contact, public health initiatives need to promote personal hygiene practices among school-aged children, including regular nail-trimming and cleanliness.

The current study found a better diagnostic performance of the POC-CCA assay using a single urine sample than duplicate KK thick smears from a single stool sample. Thus, among the 96 KK positive samples, the POC-CCA test identified 10 (10.4%) as negative. Conversely, with KK negative results, POC-CCA classified only 78 (4.9%) as positive, indicating that the KK technique has high specificity. Using the KK technique as the gold standard, the urine-based POC-CCA rapid test showed very low specificity but higher sensitivity. Its higher sensitivity might be due to its ability to detect low levels of circulating antigens in urine, enabling the identification of light infections and broader detection of Schistosoma species. However, it has lower specificity because of potential cross-reactivity with other infections, persistent antigen presence after treatment, variability in individual immune response, and population differences.

Overall, the results indicated a moderate agreement between the two methods, with a kappa value of 0.55, $p < 0.001$, and an agreement rate of 81.5%. These findings are consistent with a study involving five countries, including Ethiopia, which compared the two techniques (20), Similar comparative analyses carried out in Kenya (63), and Egypt (19).

Conclusions

There was a moderate prevalence of Schistosoma mansoni and STH, along with a moderate intensity of schistosomiasis and poor hygiene practices. This situation underscores the necessity for additional control measures, including health education. Furthermore, there is potential to incorporate POC-CCA testing alongside the Kato-Katz method to enhance diagnostic accuracy in areas with low *S. mansoni* infection rates to monitor and improve mass deworming program disease management outcomes.

Limitations

This study has a few limitations that should be acknowledged. Firstly, the cross-sectional design provides a snapshot of *S. mansoni* and STH infection at a single point in time, which may not capture seasonal variations or fluctuations in infection rates over longer periods. Finally, the purposive selection of schools limits the generalizability of the findings to the larger community in the study area.

ACKNOWLEDGMENT

Primarily, we would like to thank Hawassa University, School of Medical Laboratory Sciences, for funding this research. Then our thanks go to the study participants and the school community for their cooperation, as their contributions were crucial to its success. Finally, we would also like to thank Tulla Primary Hospital and Bushulo Mother and Child Health Specialty Center staff, especially laboratory professionals for their dedication and hard work in collecting and conducting experiments.

References

1. Weekly epidemiological record Relevé épidémiologique hebdomadaire. World Heal Organ. 2022;97(48):621–32.
2. Natalie G, Kibira SPS, Ssenyonga R, Id JN, Id AKS, Ssempebwa JC, et al. The prevalence of schistosomiasis in Uganda : A nationally representative population estimate to inform control programs and water and sanitation interventions. PLoS Negl Trop Dis. 2019;13(8):1–21.
3. Hussen S, Assegu D, Tadesse BT, Shimelis T. Prevalence of Schistosoma mansoni infection in Ethiopia : a systematic review and meta-analysis. Trop Dis Travel Med

Vaccines. 2021;7(4):1–12.

4. WHO. WHO GUIDELINE on control and elimination of human schistosomiasis [Internet]. Vol. 3, PLoS Neglected Tropical Diseases. 2022. 1–144 p.

5. Ahmed H, Ahmed H, Hong S tae, Tag A, Bashir E, Mohamed R, et al. Prevalence , risk factors , and clinical manifestations of schistosomiasis among school children in the White Nile River basin , Sudan. Parasit Vectors. 2014;7(1):1–11.

6. Nathan, Bezerra FSM, Colley DG, Fleming FM, Homeida M, Kabatereine N, et al. Review of 2022 WHO guidelines on the control and elimination of schistosomiasis. Lancet Infect Dis. 2022;22(11):e327–35.

7. Dahal A, Francis E, Francis J, Wamtas F. Soil-transmitted helminths and associated risk factors among elementary school pupils in Dadin Kowa, Jos. Niger Med J. 2019;60(4):181.

8. Getaneh M, Hailegebriel T, Munshea A. Prevalence and Associated Risk Factors of Soil-Transmitted Helminth Infections among Schoolchildren around Lake Tana , Northwest Ethiopia. J Parasitol Res. 2022;2022:13.

9. Wale M, Gedefaw S. Prevalence of Intestinal Protozoa and Soil Transmitted Helminths Infections among School Children in Jaragedo Town, South Gondar Zone of Ethiopia. J Trop Med. 2022;2022:11.

10. Beyene B, Vaz Nery S, Lambiyo T, Shimelis T. Schistosoma mansoni and soil-transmitted helminth infections among schoolchildren living along the shore of Lake Hawassa, southern Ethiopia. Parasites and Vectors [Internet]. 2024;17(1):1–14.

11. Pullan RL, Smith JL, Jasrasaria R, Brooker SJ. Global numbers of infection and disease burden of soil transmitted helminth infections in 2010. Parasites and Vectors [Internet].

2014;7(1):1–19.

12. Östan I, Kilimcioğlu AA, Girginkardeşler N, Özyurt BC, Limoncu ME, Ok ÜZ. Health inequities: Lower socio-economic conditions and higher incidences of intestinal parasites. BMC Public Health. 2007;7:1–8.

13. De Silva NR, Brooker S, Hotez PJ, Montresor A, Engels D, Savioli L. Soil-transmitted helminth infections: Updating the global picture. Trends Parasitol. 2003;19(12):547–51.

14. Vercruysse J, Albonico M, Behnke J, Kotze A, Mccarthy J, Prichard R, et al. Monitoring Anthelmintic Efficacy for Soil Transmitted Helminths (STH). World Heal Organ. 2008;1–64.

15. Montresor A, Mupfasoni D, Mikhailov A, Mwinzi P, Lucianez A, Jamsheed M, et al. The global progress of soil-transmitted helminthiases control in 2020 and world health organization targets for 2030. PLoS Negl Trop Dis. 2020;14(8):1–17.

16. Sousa MS, Dam GJ Van, Cristhiany M, Pinheiro C, Dood CJ De, Peralta JM, et al. Performance of an Ultra-Sensitive Assay Targeting the Circulating Anodic Antigen (CAA) for Detection of Schistosoma mansoni Infection in a Low Endemic Area in Brazil. Front Immunol. 2019;10:1–16.

17. Lindholz CG, Favero V, Verissimo CDM, Russo R, Candido F, Souza RP De, et al. Study of diagnostic accuracy of Helmintex , Kato-Katz , and POC-CCA methods for diagnosing intestinal schistosomiasis in Candeal , a low intensity transmission area in northeastern Brazil. PLoS Negl Trop Dis. 2018;12(3):1–16.

18. Mueller A, Fuss A, Ziegler U, Kaatano GM, Mazigo HD. Intestinal schistosomiasis of Ijinga Island , north-western Tanzania : prevalence , intensity of infection , hepatosplenic morbidities and their associated factors. BMC Infect Dis. 2019;19(832):1–12.

19. Haggag AA, Rabiee A, Elaziz KMA, Campbell CH, Colley DG, Ramzy RMR. Thirty-Day Daily Comparisons of Kato – Katz and CCA Assays of 45 Egyptian Children in Areas with Very Low Prevalence of Schistosoma mansoni. Am Soc Trop Med Hyg. 2019;100(3):578–83.

20. Colley DG, Binder S, Campbell C, King CH, Tchuenté LAT, N'Goran EK, et al. A five-country evaluation of a point-of-care circulating cathodic antigen urine assay for the prevalence of Schistosoma mansoni. Am J Trop Med Hyg. 2013;88(3):426–32.

21. Straily A, Kavere EA, Wanja D, Wiegand RE, Montgomery SP, Mwaki A, et al. Evaluation of the Point-of-Care Circulating Cathodic Antigen Assay for Monitoring Mass Drug Administration in a Schistosoma mansoni Control Program in Western Kenya. Am Soc Trop Med Hyg. 2022;106(1):303–11.

22. Tadege B, Shimelis T. Infections with Schistosoma mansoni and geohelminths among school children dwelling along the shore of the Lake Hawassa , southern Ethiopia. PLoS One. 2017;12(7):1–12.

23. WHO. Helminth control in school-age children Helminth control in. A Guid Manag Control Program. 2011;2:1–90.

24. Casacuberta-Partal M, Hoekstra PT, Kornelis D, van Lieshout L, van Dam GJ. An innovative and user-friendly scoring system for standardised quantitative interpretation of the urine-based point-of-care strip test (POC-CCA) for the diagnosis of intestinal schistosomiasis: a proof-of-concept study. Acta Trop [Internet]. 2019;2(17):1–4.

25. Okoyo C, Simiyu E, Njenga SM, Mwandawiro C. Comparing the performance of circulating cathodic antigen and Kato-Katz techniques in evaluating Schistosoma mansoni infection in areas with low prevalence in selected counties of Kenya: A cross-sectional

study. BMC Public Health. 2018;18(1):1–7.
26. Tiruneh A, Zemene E, Abdissa Mizana B, Girma H, Dereje E, Sharew B, et al. Schistosoma mansoni Infections and Morbidities Among School Children in Hotspot Areas of Jimma Town, Southwest Ethiopia: A Cross-Sectional Study. Environ Health Insights. 2023;17:1–8.
27. Aliyi H, Ahmed M, Gobena T, Alemu BM, Adem HA, Usso AA. Prevalence and factors associated with Schistosoma mansoni infection among primary school children in Kersa District, Eastern Ethiopia. PeerJ. 2024;12(6):1–20.
28. Ashenafi T, Tadesse D, Zewdneh T. Infection prevalence of intestinal helminths and associated risk factors among schoolchildren in selected kebeles of Enderta district, Tigray, Northern Ethiopia. J Parasitol Vector Biol. 2014;6(11):166–73.
29. Ghazy RM, Ellakany WI, Badr MM, Taktak NEM, Elhadad H, Abdo SM, et al. Determinants of Schistosoma mansoni transmission in hotspots at the late stage of elimination in Egypt. Infect Dis Poverty. 2022;11(1):1–14.
30. Bartlett AW, Figueiredo JCS, Goor RC Van, Monaghan P, Lancaster W, Mugizi R, et al. Burden and factors associated with schistosomiasis and soil - transmitted helminth infections among school - age children in Huambo , Uige and Zaire provinces , Angola. Infect Dis Poverty [Internet]. 2022;11(73):1–15.
31. Wubet K, Damtie D. Prevalence of Schistosoma mansoni Infection and Associated Risk Factors among School Children in Jiga Town , Northwest-Ethiopia : A Cross-Sectional Study. J Parasitol Res. 2020;2020:1–7.
32. Tazebew B, Temesgen D, Alehegn M, Salew D. Prevalence of S . mansoni Infection and Associated Risk Factors among School Children in Guangua District , Northwest

Ethiopia. J Parasitol Res. 2022;2022:1–9.

33. Ansha MG, Kuti KA, Girma E. Prevalence of Intestinal Schistosomiasis and Associated Factors among School Children in Wondo District , Ethiopia. J Trop Med. 2020;10:1–8.

34. Hailegebriel, Nibret, Munshea A et al. Prevalence , intensity and associated risk factors of Schistosoma mansoni infections among schoolchildren around Lake Tana , northwestern Ethiopia. PLoS Negl Trop Dis [Internet]. 2021;15(10):1–16.

35. Azalech Tefera, Belay B. Epidemiology of Schistosoma mansoni infection and associated risk factors among school children attending primary schools nearby rivers in Jimma town , an urban setting , Southwest Ethiopia. PLoS One [Internet]. 2020;15(2):1–15.

36. Zeleke AJ, Addisu A, Tegegne Y. Prevalence, Intensity, and Associated Factors of Schistosoma mansoni among School Children in Northwest Ethiopia. J Parasitol Res. 2020;2020:1–7.

37. Assefa A, Erko B, Gundersen SG, Medhin G, Berhe N. Current status of Schistosoma mansoni infection among previously treated rural communities in the Abbey and Didessa Valleys , Western Ethiopia : Implications for sustainable control. PLoS One [Internet]. 2021;16(2):1–15.

38. Alemayehu B, Tomass Z. Schistosoma mansoni infection prevalence and associated risk factors among schoolchildren in Demba Girara, Damot Woide District of Wolaita Zone, Southern Ethiopia. Asian Pac J Trop Med [Internet]. 2015;8(6):457–63.

39. Masaku J, Madigu N, Okoyo C, Njenga SM. Current status of Schistosoma mansoni and the factors associated with infection two years following mass drug administration programme among primary school children in Mwea irrigation scheme : A cross- sectional study. BMC Public Health. 2015;15(739):1–9.

40. Nigo MM, Odermatt P, Salieb-Beugelaar GB, Morozov O, Battegay M, Hunziker PR. Epidemiology of schistosoma mansoni infection in Ituri province, North-Eastern democratic republic of the Congo. PLoS Negl Trop Dis [Internet]. 2021;15(12):1–26.

41. Hussein R, Id M, Minzi OS, Kinung SM, Kamuhabwa A, Aklillu E. Prevalence and correlates of intestinal schistosomiasis infection among school-aged children in North-Western Tanzania. PLoS One. 2020;15(2):1–17.

42. Aemero M, Berhe N, Erko B. Status of Schistosoma mansoni prevalence and intensity of infection in geographically apart endemic localities of Ethiopia: a comparison. Ethiop J Health Sci. 2014;24(3):189–94.

43. Bajiro M, Dana D, Levecke B. Prevalence and intensity of schistosoma mansoni infections among schoolchildren attending primary schools in an urban setting in southwest, ethiopia. BMC Res Notes [Internet]. 2017;10(1):1–6.

44. Montresor A, Mupfasoni D, Mikhailov A, Mwinzi P, Lucianez A, Jamsheed M, et al. 12879_2019_Article_4730.pdf. PLoS Negl Trop Dis. 2020;8(8):1–8.

45. Gass K, Addiss DG, Freeman MC. Exploring the Relationship between Access to Water, Sanitation and Hygiene and Soil-Transmitted Helminth Infection: A Demonstration of Two Recursive Partitioning Tools. PLoS Negl Trop Dis. 2014;8(6).

46. Jia TW, Melville S, Utzinger J, King CH, Zhou XN. Soil-transmitted helminth reinfection after drug treatment: A systematic review and meta-analysis. PLoS Negl Trop Dis. 2012;6(5).

47. Brooker SJ, Mwandawiro CS, Halliday KE, Njenga SM, McHaro C, Gichuki PM, et al. Interrupting transmission of soiltransmitted helminths: A study protocol for cluster randomised trials evaluating alternative treatment strategies and delivery systems in

Kenya. BMJ Open. 2015;5(10):1–12.

48. Giardina F, Coffeng LE, Farrell SH, Vegvari C, Werkman M, Truscott JE, et al. Sampling strategies for monitoring and evaluation of morbidity targets for soil-transmitted helminths. PLoS Negl Trop Dis. 2019;13(6):1–17.

49. Fenta A, Mesganaw B, Belachew F, Teshale G, Abebaw G, Tesfa E, et al. Prevalence and risks of soil transmitted helminths among Ethiopian school children: A cross-sectional study. Asian Pac J Trop Med. 2022;15(3):121–7.

50. Samuel F, Demsew A, Alem Y, Hailesilassie Y. Soil transmitted Helminthiasis and associated risk factors among elementary school children in ambo town, western Ethiopia. BMC Public Health. 2017;17(1):1–7.

51. Tolera B, Mohammed J, Umer U, Abamecha J, Tebeje F, Sime A, et al. Prevalence, intensity, and associated factors of intestinal schistosomiasis among primary school children in Nono District, Southwest Ethiopia. Front Trop Dis. 2024;5(February):1–11.

52. Alemu G, Abossie A, Yohannes Z. Current status of intestinal parasitic infections and associated factors among primary school children in Birbir town, Southern Ethiopia. BMC Infect Dis. 2019;19(1):4–11.

53. Zeynudin A, Degefa T, Suleman S, Abamecha A, Hajikelil Z, Wieser A. Prevalence and Determinants of Geohelminthiasis among School-Age Children in Jimma City, Ethiopia. J Trop Med. 2023;2023.

54. Bokicho B, Hailu D, Eshetu B, Matie M, Tadele T. Soil-transmitted helminthiases among school-age children and their association with water, sanitation, and hygiene, Hawassa City, Southern Ethiopia. PLoS Negl Trop Dis [Internet]. 2023;17(7):1–12.

55. Luby SP, Agboatwalla M, Painter J, Altaf A, Billhimer WL, Hoekstra RM. Effect of

Intensive Handwashing Promotion on Childhood Diarrhea in High-Risk Communities in PakistanA Randomized Controlled Trial. JAMA [Internet]. 2004 Jun 2;291(21):2547–54.

56. Feleke DG, Ali A, Bisetegn H, Debash H, Birara W, Andualem A. Soil-transmitted helminthes and Schistosoma mansoni infections among primary school children at Ambasame primary school, North-West Ethiopia: a cross-sectional study. BMC Pediatr [Internet]. 2022;22(1):1–7. Available from: https://doi.org/10.1186/s12887-022-03534-5

57. Forson AO, Arthur I, Ayeh-Kumi PF. The role of family size, employment and education of parents in the prevalence of intestinal parasitic infections in school children in Accra. PLoS One. 2018;13(2):1–10.

58. Kihoro RW, Mulewa D, Okoyo C, Ayaa D, Korir A, Njomo DW, et al. Epidemiology of soil-transmitted helminthiasis among school-aged children in pastoralist communities of Kenya: A cross-sectional study. PLoS One [Internet]. 2024;19(5 MAY):1–20.

59. Debalke S, Worku A, Jahur N, Mekonnen Z. Soil transmitted helminths and associated factors among schoolchildren in government and private primary school in Jimma Town, Southwest Ethiopia. Ethiop J Health Sci. 2013;23(3):237–44.

60. Genet A, Motbainor A, Samuel T, Azage M. Prevalence and associated factors of soil transmitted helminthiasis among school-age children in wetland and non-wetland areas of Blue Nile Basins, northwest Ethiopia: A community-based comparative study. SAGE Open Med. 2021;9.

61. Andargie Y, Alelign A, Tekeste Z. Prevalence and associated risk factors of soil-transmitted helminth infections among schoolchildren in Mekan Eyesus town, northwestern Ethiopia. Parasite Epidemiol Control [Internet]. 2024;27(March):e00379.

62. Novianty S, Dimyati Y, Pasaribu S, Pasaribu AP. Risk Factors for Soil-Transmitted

Helminthiasis in Preschool Children Living in Farmland, North Sumatera, Indonesia. J Trop Med. 2018;2018.

63. Chieng B, Okoyo C, Simiyu E, Gichuki P, Mwatele C, Kepha S, et al. Comparison of quantitative polymerase chain reaction, Kato-Katz and circulating cathodic antigen rapid test for the diagnosis of Schistosoma mansoni infection: A cross-sectional study in Kirinyaga County, Kenya. Curr Res Parasitol Vector-Borne Dis [Internet]. 2021;1(5):100029.

Additional file

Table Legends

Table 4: The distribution of *Schistosoma mansoni* infection by socio-demographic and other factors among schoolchildren in Tulla district, Sidama region, Southern Ethiopia 2024 (n = 477).

Table 3: Univariate and multivariate logistic regression analyses for socio-demographic and other potential risk factors associated with Soil-transmitted helminths (STHs) infections among school children in Tulla district, Sidama region, Southern Ethiopia 2024 (n = 477).

Figure Legends

File 1: Figure 1: Map of the study site. The figure in the left corner depicts Lake Hawassa and the study district, while the upper right corner shows Ethiopia. The figure at the bottom right displays the map of the Sidama region.

File 2: Figure 2: Infection intensity of *S. mansoni* among schoolchildren in Tulla district 2024 (n

= 477).

File 3: Figure 3: The distribution of intestinal helminths among schoolchildren in Tulla district 2024 (n = 477). SM, *S. mansoni*; AL, *Ascaris lumbricoides*; EV, *Enterobius vermicularis*; HW, *Hook worm*; TS, *Taenia* species; TT, *Trichuris trichuria*; HN, *Hymenolepis nana*

Abbreviation

AOR, adjusted odds ratio; CCA, circulating cathodic antigen; CI, Confidence interval; COR, Crude odds ratio; EPG, Eggs per gram; KK, Kato Katz; LR^+, Positive likelihood ratio; LR^-, N likelihood ratio; MDA, Mass drug administration; NPV, Negative predictive value; NTD, neglected tropical disease; POC, Point of care; PPV, Positive predictive value; SOP, Standard operating procedure; STH, Soil-transmitted Helminth; WHO, World Health Organization

Availability of data and materials

All relevant data are within the manuscript and the datasets generated and/or analyzed during the current study are available from the corresponding author upon reasonable request, subject to the approval of the Institutional Review Board of the Hawassa University College of Medicine and Health Sciences. Restrictions apply to the availability of these data, which parents had consented for the collected information to be used for our research study only, and so are not publicly available.

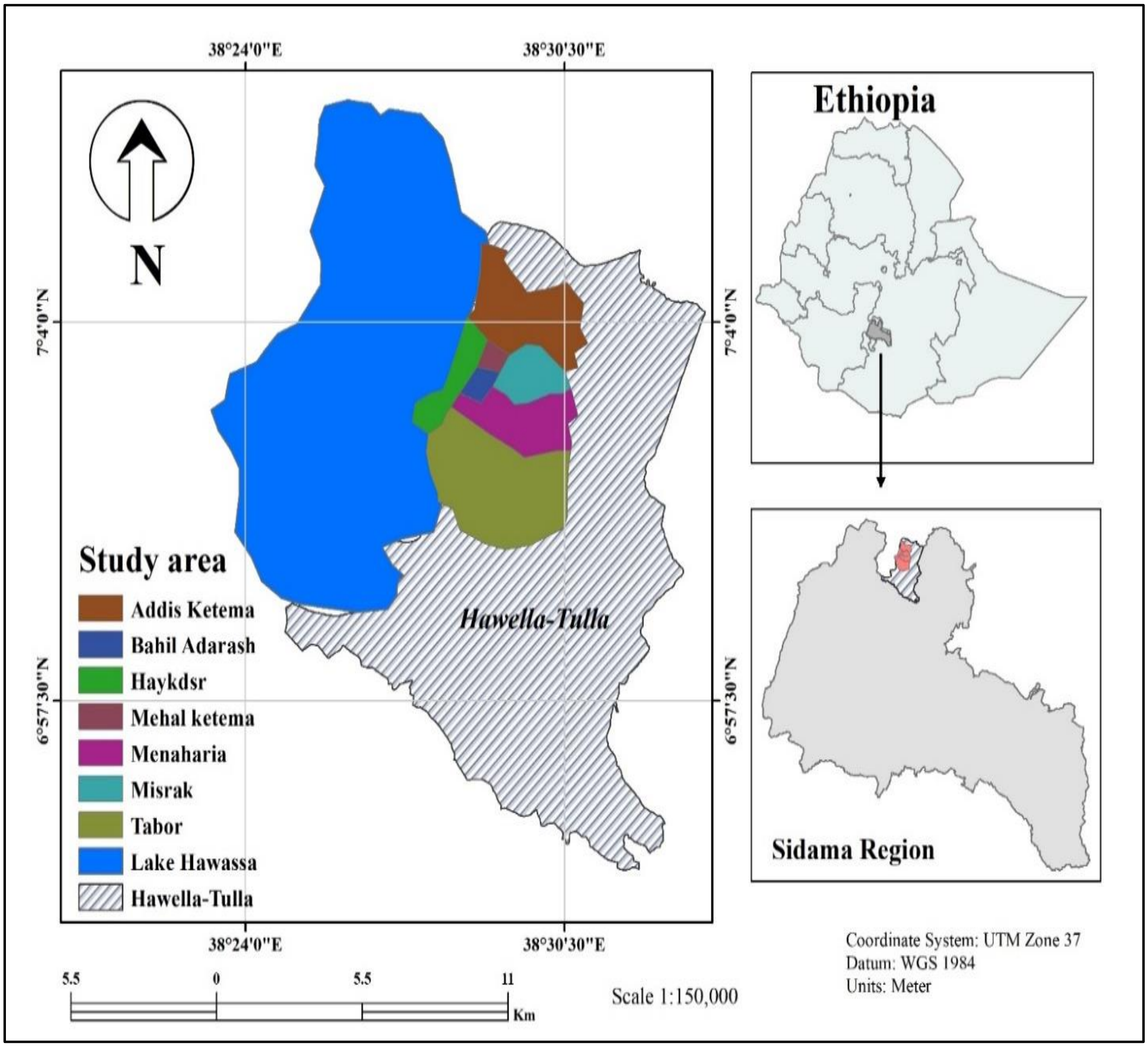

Figure 1: Map of the study site. The figure in the left corner depicts Lake Hawassa and the study district, while the upper right corner shows Ethiopia. The figure at the bottom right displays the map of the Sidama region.

Figure Click here to access/download;Figure;Figure 2 (1).docx

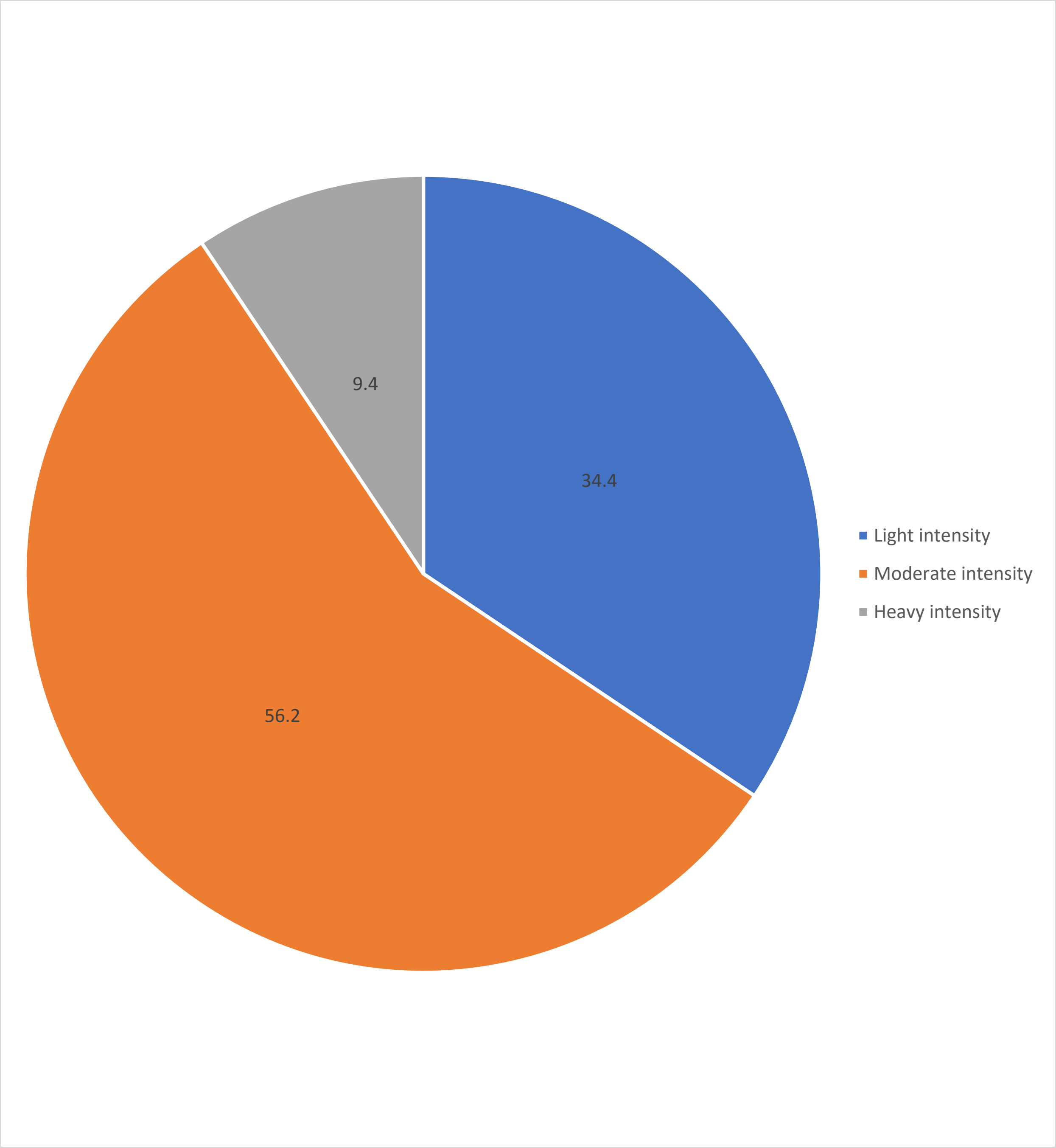

Figure 2: Infection intensity of *S. mansoni* among schoolchildren in Tulla district 2024 (n = 477).

 Click here to access/download;Figure;Figure 3 (1).docx

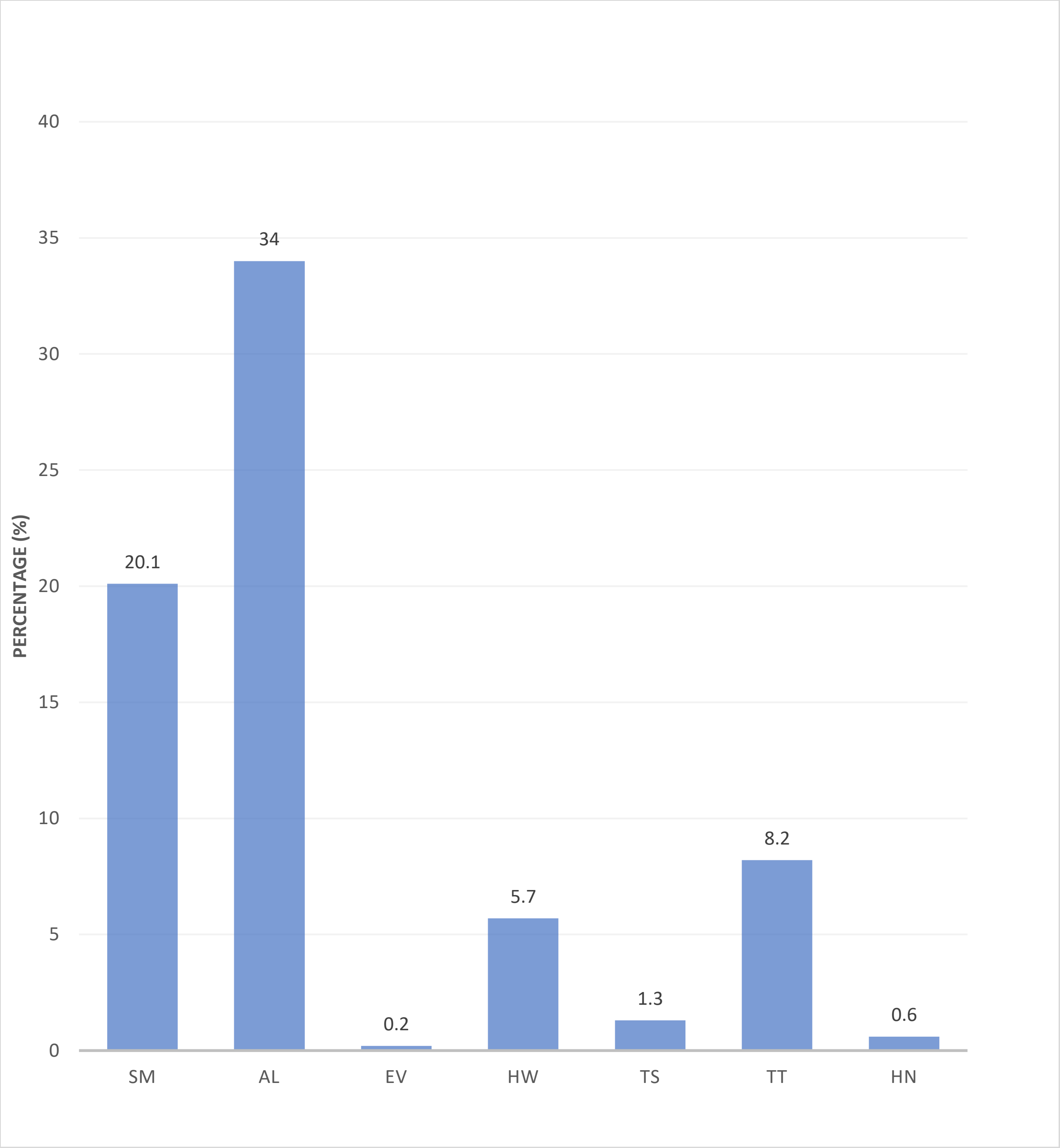

Figure 3: The distribution of intestinal helminths with Kato katz among schoolchildren in Tulla district 2024 (n = 477). SM, *S. mansoni*; AL, *Ascaris lumbricoides*; EV, *Enterobius vermicularis*; HW, *Hook worm*; TS, *Taenia* species; TT, *Trichuris trichuria*; HN, *Hymenolepis nana*

 Click here to access/download;Figure;Table 2.docx

Table 1: The distribution of *Schistosoma mansoni* infection by socio-demographic and other factors among schoolchildren in Tulla district, Sidama region, Southern Ethiopia 2024 (n = 477).

Variables	*S. mansoni*			COR (95% CI)	p-value	AOR (95%CI)	p-value
	Positive %	Negative %	Total %				
Gender							
Male	52(25.0)	156(75.0)	208(43.6)	1		1	
Female	44(16.4)	225(83.6)	269(56.4)	0.587(0.374-0.920)	0.020	0.607(0.359-1.027)	0.063
Age							
5-9	7(9.1)	70(90.9)	77(16.1)	1			
10-14	67(22.4)	232(77.6)	299(62.7)	2.888(1.268-6.578)	0.012	2.118(0.841-5.330)	0.111
15-19	22(21.8)	79(78.2)	101(21.2)	2.785(1.122-6.914)	0.027	1.442(0.495-4.198)	0.502
School							
Bushulo	52(36.4)	91(63.6)	143(30)	6.163(3.234-11.746)	0.001	4.685(1.869-11.742)	**0.001****
Finchawa	13(24.5)	40(75.5)	53(11.1)	3.505(1.526-8.051)	0.003	1.444(0.486-4.297)	0.509
Gemeto	17(14.6)	99(85.4)	116(24.3)	1.852(0.874-3.926)	0.108	1.528(0.662-3.525)	0.320
Tulla	14(8.5)	151(91.5)	165(34.6)	1			
Educational status of the father							
No formal education	17(18.5)	75(81.5)	92(19.3)	1		1	
Primary school	58(26.0)	165(74.0)	223(46.75)	1.551(0.846-2.841)	0.156	1.420(0.690-2.921)	0.341
Educational status of the mother							
No formal education	3(7.0)	40(93.0)	43(9)	1		1	
Primary school	62(23.2)	205(76.8)	267(56)	4.033(1.206-13.485)	0.024	3.391(0.889-12.937)	0.074
Secondary school	25(18.8)	108(81.2)	133(28)	3.086(0.883-10.787)	0.078	2.178(0.535-8.859)	0.277
College and above	6(17.7)	28(82.3)	34(7)	2.857(0.658-12.397)	0.161	2.384(0.414-13.719)	0.331
Distance of residential area							

Within 1km	49(30.6)	111(69.4)	160(33.5)	1		1	
More than 1km	47(14.8)	270(85.2)	317(66.5)	0.394(0.250-0.623)	0.001	1.194(0.615-2.318)	0.601
Bathing in the lake							
Yes	43(28.5)	108(71.5)	151(31.7)	2.051(1.295-3.248)	0.002	1.804(1.048-3.107)	**0.033***
No	53(16.3)	273(83.7)	326(68.3)	1		1	
Swimming habit							
Yes	50(31.9)	107(68,1	157(32.9)	2.783(1.780-4.403)	0.001	2.407(1.430-4.052)	**0.001****
No	46(14.4)	274(85.6)	320(67.1)	1			
Fishing							
Yes	20(35.1)	37(64.9)	57(12)	2.447(1.345-4.449)	0.003	1.135(0.533-2.417)	0.743
No	76(18.1)	344(71.9)	420(88)	1			
Washing clothes along the lake shore							
Yes	19(30.6)	43(69.4)	62(13)	1.940(1.071-3.513)	0.029	2.016(0.934-4.350)	0.074
No	77(17.3)	338(82.7)	415(87)	1			
Involvement in irrigation							
Yes	44(37.0)	75(63.0)	119(25)	3.452(2.148-5.547)	0.001	2.429(1.377-4.287)	**0.002***
No	52(14.5)	306(85.5)	358(75)	1			

AOR, Adjusted Odds Ratio; CI, Confidence interval; COR, Crude Odds Ratio; *, Significant at $p < 0.05$.

Table 3: Univariate and multivariate logistic regression analyses for socio-demographic and other potential risk factors associated with Soil-transmitted helminths (STHs) infections among school children in Tulla district, Sidama region, Southern Ethiopia 2024 (n = 477).

Variables	STH Positive %	Negative %	Total %	COR (95% CI)	p-value	AOR (95%CI)	p-value
Gender							
Male	97(46.6)	111(53.4)	208(43.6)	1			
Female	136(50.6)	133(49.4)	269(56.4)	1.170(0.814-1.681)	0.395		
Age							
5-9	38(49.4)	39(50.6)	77(16.1)	1			
10-14	151(50.5)	148(49.5)	299(62.7)	1.047(0.634-1.728)	0.857		
15-19	44(43.6)	57(56.4)	101(21.2)	0.792(0.437-1.437)	0.443		
School							
Bushulo	76(53.2)	67(46.8)	143(30)	1		1	
Finchawa	28(52.8)	25(47.2)	53(11.1)	0.987(0.525-1.857)	0.969		
Gemeto	54(46.5)	62(53.5)	116(24.3)	0.768(0.470-1.254)	0.292		
Tulla	75(45.5)	90(54.5)	165(34.6)	0.735(0.469-1.151)	0.178	0.469(0.256-0.861)	**0.015***
Grade level							
1-4	118(48.2)	127(51.8)	245(51.4)	1		1	
5-8	126(54.3)	106(45.7)	232(48.6)	1.279(0.893-1.834)	0.180	0.848(0.495-1.453)	0.548
Educational status of father							
No formal education	55(59.8)	37(40.2)	92(19.3)	3.964(1.420-11.066)	0.009	4.161(1.276-13.570)	**0.018***
Primary school	115(51.6)	108(48.4)	223(46.7)	2.840(1.072-7.523)	0.036	2.957(0.991-8.818)	0.052
Secondary education	57(40.7)	83(59.3)	140(29.4)	1.831(1.072-7.523)	0.234	1.616(0.547-4.775)	0.385
College and above	6(27.3)	16(72.7)	22(4.6)	1		1	
Occupation of family							

Civil servant	6(31.6)	13(68.4)	19(4)	1		1	
Merchant	56(41.5)	79(58.5)	135(28.3)	1.536(0.550-4.285)	0.412		
Farmer	63(50.8)	61(49.2)	124(26)	2.238(0.799-6.264)	0.125	0.199(0.013-3.028)	0.245
Private (Daily laborer)	108(54.3)	91(45.7)	199(41.7)	2.571(0.940-7.037)	0.066	0.262(0.017-3.932)	0.332
Latrine availability at home							
Yes	128(42)	177(58)	305(63.9)	1		1	
No	105(61)	67(39)	172(36.1)	0.461(0.315-0.676)	0.001	0.626(0.393-0.996)	**0.048***
Hand washing after latrine							
Yes	6(12.8)	41(87.2)	47(9.9)	1		1	
No	227(52.8)	203(47.2)	430(90.1)	0.131(0.54-0.315)	0.001	0.199(0.072-0.551)	**0.002***
Shoe wearing							
Yes	198(49.25	204(50.75)	402(84.3)	1			
No	35(46.7)	40(53.3)	75(15.7)	0.902(0.550-1.477)	0.681		
Finger nail trimming							
Yes	39(29.3)	94(70.7)	133(27.9)	1		1	
No	194(56.4)	150(43.6)	344(72.1)	0.321(0.209-0.493)	0.001	0.413(0.250-0.682)	**0.001***
Hand washing before meal							
Yes	37(33.6)	73(66.4)	110(23)	1		1	
No	196(53.4)	171(46.6)	367(77)	0.442(0.283-0.890)	0.001	0.585(0.337-1.015)	0.058
Open field defecation							
Yes	24(48)	26(52)	50(10.5)	1.039(0.578-1.867)	0.899		
No	209(48.9)	218(51.1)	427(89.5)	1			

Key - AOR: Adjusted Odds Ratio, CI: Confidence interval, COR: Crude Odds Ratio, *: Significant at $p < 0.05$, STH: Soil-transmitted helminths.
