# Supplementary material for: Assessment of Schistosoma mansoni and soil-transmitted helminth infections and the diagnostic performance of the circulating cathodic antigen test among schoolchildren in Tulla district, Sidama region, Southern Ethiopia
Source: PLoS One. 2025 Dec 12;20(12):e0337919. doi: 10.1371/journal.pone.0337919 (PMC12700372; doi:10.1371/journal.pone.0337919)
Supplement: Attachment — Submitted filename: PONE-D-25-23474_R1 Sept 2025.pdf [file pone.0337919.s006.pdf]

PLOS ONE

Assessment of Schistosoma mansoni and Soil-transmitted Helminth Infections and the Diagnostic Performance of the Circulating Cathodic Antigen Test among Schoolchildren in Tulla District, Sidama Region, Southern Ethiopia.

--Manuscript Draft--

**Manuscript Number:**	PONE-D-25-23474R1
**Article Type:**	Research Article
**Full Title:**	Assessment of Schistosoma mansoni and Soil-transmitted Helminth Infections and the Diagnostic Performance of the Circulating Cathodic Antigen Test among Schoolchildren in Tulla District, Sidama Region, Southern Ethiopia.
**Short Title:**	Schistosoma mansoni Infection and Performance its antigen test
**Corresponding Author:**	Bamlaku Tadege Tadege, PhD<br>Hawassa University College of Medicine and Health Sciences<br>Hawassa, ETHIOPIA
**Keywords:**	Prevalence; Infection intensity; Schistosoma mansoni; Soil-transmitted helminth; Diagnostic performance; Kato-Katz technique; Point-of-care circulating cathodic antigen
**Abstract:**	Background: Soil-transmitted helminths (STH) and Schistosoma mansoni pose significant public health challenges in areas with poor sanitation. While S. mansoni infection leads to chronic illness and mortality, research on its intensity remains limited in the study area. Diagnosis traditionally relies on the Kato-Katz method, though the POC-CCA urine test offers a rapid alternative. This study examined the prevalence, intensity, and risk factors of S. mansoni and Soil-transmitted helminth infections among schoolchildren while evaluating the diagnostic performance of the POC-CCA test.<br>Methods: A school-based cross-sectional study was conducted in the Tulla district, Sidama region, among 477 students (April–June 2024). The Schoolchildren were recruited using a purposive sampling technique. Socio-demographic data were collected using pre-structured questionnaires. A single stool sample was collected from each study participant and processed for the detection of helminth eggs using the Kato-Katz (KK) technique. Additionally, urine samples were tested with the Point of Care Circulating Cathodic Antigen Test (POC-CCA), which detects Schistosoma antigens. Data was analyzed using SPSS and McNemar's chi-square test.<br>Result: The overall prevalence of Schistosoma mansoni was 36.5% (20.1% by KK and 34.4% by POC-CCA). The prevalence of STH was 48.8%. The leading parasite was Ascaris lumbricoides (34%), followed by Trichuris trichuria (8.2%) and hookworm (5.7%). Other helminths included Taenia species 1.3%, Hymenolepis nana 0.6%, and Enterobius vermicularis 0.2%. Infection intensity of S. mansoni was classified as light 34.4%, moderate 56.2%, and heavy 9.4%. Involvement in irrigation, swimming, bathing, and school location were significantly associated with S. mansoni infection. School location, educational status, lack of latrine, hand washing practice and fingernail trimming practice were significantly associated with STH infections. The POC-CCA rapid test had a significantly higher sensitivity of 89.6% (McNemar test χ^2m = 52.3, $p < 0.001$) compared to the Kato-Katz technique.<br>Conclusions: Moderate S. mansoni and STH infection and poor hygiene practice underscore for additional control measures. Incorporating POC-CCA testing alongside the KK method could optimize diagnostic accuracy and improve disease management outcomes.
**Order of Authors:**	Bamlaku Tadege Tadege, PhD
	Fitsum Getachew Getachew, MSC
	Demissie Assegu Fenta, MSC
	Biniyam Kijineh Kijineh, MSC
**Response to Reviewers:**	Response to the PLoS ONE editor and reviewers<br>First and foremost, we sincerely thank you for reviewing our manuscript and offering valuable comments and suggestions. Your insights have been very helpful in

	highlighting areas for improvement, and we have carefully addressed each of the points you raised.<br>1. Please ensure that your manuscript meets PLOS ONE's style requirements, including those for file naming. The PLOS ONE style templates can be found at https://journals.plos.org/plosone/s/file?id=wjVg/PLOSOne_formatting_sample_main_body.pdf and https://journals.plos.org/plosone/s/file?id=ba62/PLOSOne_formatting_sample_title_authors_affiliations.pdf.<br>We followed our manuscript to meet PLOS ONE's style requirements, including those for file naming.<br>2. PLOS requires an ORCID iD for the corresponding author in Editorial Manager on papers submitted after December 6th, 2016. Please ensure that you have an ORCID iD and that it is validated in Editorial Manager. To do this, go to ‘Update my Information’ (in the upper left-hand corner of the main menu), and click on the Fetch/Validate link next to the ORCID field. This will take you to the ORCID site and allow you to create a new iD or authenticate a pre-existing iD in Editorial Manager.<br>The corresponding author's ORCID ID is addressed<br>3. We note that your Data Availability Statement is currently as follows: [data are all contained within the manuscript and/or Supporting Information files, enter the following: All relevant data are within the manuscript and its Supporting Information files.]<br>Please confirm at this time whether or not your submission contains all raw data required to replicate the results of your study. Authors must share the “minimal data set” for their submission. PLOS defines the minimal data set to consist of the data required to replicate all study findings reported in the article, as well as related metadata and methods<br><br><br>We included raw data used for this manuscript results analysis<br><br>Reviewers' comments:<br><br>Reviewer's Responses to Questions<br>Comments to the Author<br><br>1. Is the manuscript technically sound, and do the data support the conclusions?<br><br>The manuscript must describe a technically sound piece of scientific research with data that supports the conclusions. Experiments must have been conducted rigorously, with appropriate controls, replication, and sample sizes. The conclusions must be drawn appropriately based on the data presented.<br>Reviewer #1: Yes<br>Reviewer #2: Yes<br>We thank all reviewers<br>____<br>2. Has the statistical analysis been performed appropriately and rigorously?<br>Reviewer #1: Yes<br>Reviewer #2: Yes<br>We thank all reviewers<br><br>____<br>3. Have the authors made all data underlying the findings in their manuscript fully available?<br><br>The PLOS Data policy requires authors to make all data underlying the findings described in their manuscript fully available without restriction, with rare exception (please refer to the Data Availability Statement in the manuscript PDF file). The data should be provided as part of the manuscript or its supporting information, or deposited to a public repository. For example, in addition to summary statistics, the data points behind means, medians and variance measures should be available. If there are restrictions on publicly sharing data—e.g. participant privacy or use of data from a third party—those must be specified.<br>Reviewer #1: Yes<br>Reviewer #2: No<br>Thank you for your curiosity. We attached raw data and supplementary information, like the questionnaire

4. Is the manuscript presented in an intelligible fashion and written in Standard English?

PLOS ONE does not copyedit accepted manuscripts, so the language in submitted articles must be clear, correct, and unambiguous. Any typographical or grammatical errors should be corrected at revision, so please note any specific errors here.
Reviewer #1: Yes
Reviewer #2: No
We revised our manuscript once again for any topographical and grammatical corrections in the revised manuscript and indicated on the marked revised manuscript

5. Review Comments to the Author

Please use the space provided to explain your answers to the questions above. You may also include additional comments for the author, including concerns about dual publication, research ethics, or publication ethics. (Please upload your review as an attachment if it exceeds 20,000 characters)
Reviewer #1: The manuscript is well written, the methodology, results, statistical analysis and discussion were well presented. The manuscript is technically sound, and data provided in the manuscript. The statistical analysis is appropriate. All findings were outlined in the manuscript. The manuscript is presented in Standard English.

The authors should address the comments made on the manuscript.
Reviewer #2: General comments
The manuscript presents a well-conducted cross-sectional study on the prevalence, intensity, and risk factors of Schistosoma mansoni and soil-transmitted helminth (STH) infections among schoolchildren in Ethiopia, alongside an evaluation of the diagnostic performance of the Point-of-Care Circulating Cathodic Antigen (POC-CCA) test. The study addresses an important public health issue in a region where these infections are endemic. The methodology is robust, and the findings are relevant to ongoing control efforts. However, there are several areas where the manuscript could be improved to enhance clarity, accuracy, and impact.
1. Ethical considerations: The manuscript states that ethical clearance was obtained from Hawassa University, but the reference number provided (IRB/172/16) appears outdated (2016). Given that the study was conducted in 2024, clarification or updated ethical approval documentation is necessary. Again, the consent process is described, but it would be beneficial to explicitly state whether assent was obtained from all children, including those under 12, or only those aged 12 and above.
We want to clarify that the ethical clearance reference number is not outdated; it is based on the Ethiopian calendar (E.C.). The reference number: IRB/172/16 E.C. corresponds to the Gregorian calendar date of 22/03/2024, which is indicated on the ethical clearance letter. We add E.C. to indicate the calendar, see the correction on line number 242 in the revised manuscript. Additionally, we acknowledge that our initial manuscript did not mention that assent was obtained from students aged ≥12 years following parent/guardian consent. This has now been addressed and appropriately revised in the manuscript. See the revised one from line number 245 – 247
2. Data availability: The manuscript claims that "all relevant data are within the manuscript and its Supporting Information files," but no supplementary files are provided. The authors should either upload the supporting data or clarify where it can be accessed.
Regarding the comment on the supplementary files, we have separately supplementary information. We also attached raw data
3. Diagnostic performance analysis: The comparison between POC-CCA and Kato-Katz is a key contribution, but the specificity of POC-CCA (79.6%) is notably low. The authors should discuss potential reasons for this (e.g., cross-reactivity, persistent antigens post-treatment) and implications for field use. Again, the McNemar's test result ($\chi^2 = 52.3$, $p < 0.001$) indicates significant disagreement between the two tests, but the discussion does not adequately address this. The authors should elaborate on the practical implications of this discrepancy.
Regarding the specificity of the POC-CCA test (79.6%), which is notably low, we were asked to discuss potential reasons and its implications for field use. We have addressed this from line numbers 497 – 521in the revised manuscript as follows: "However, the specificity of the POC-CCA test was found to be 79.6%, which is

consistent with previously reported ranges but remains relatively low (20). This reduced specificity may be attributed to cross-reactivity with other helminth infections, the subjective interpretation of trace results as positive, and the continued excretion of CCA following recent treatment or in the absence of active infection (64). Such limitations can result in false positives, particularly in low-endemic or post-treatment settings, potentially leading to an overestimation of prevalence. Despite this, the POC-CCA test remains a practical diagnostic tool in field settings due to its operational simplicity, rapid results, and high sensitivity. However, its limited specificity underscores the importance of confirmatory testing, especially in surveillance, program evaluation, and low-transmission areas where diagnostic accuracy is critical."
Regarding the McNemar's test result ($\chi^2 = 52.3$, $p < 0.001$), we were informed that the discussion did not adequately address the observed discrepancy or elaborate on its practical implications. Therefore, we have addressed the issue as follows: "Importantly, McNemar's test revealed a statistically significant discordance between the two methods ($\chi^2 = 52.3$, $p < 0.001$), driven primarily by the high number of POC-CCA positive but KK negative cases. This lack of alignment between the two diagnostic tools underscores their fundamentally different targets: POC-CCA detects active infection through CCA in urine, whereas KK relies on the detection of Schistosoma eggs in stool, which are subject to day-to-day variation and intra-sample inconsistencies (17). Therefore, in field settings, particularly in low-endemic or post-treatment areas, the POC-CCA test may identify infections that KK misses, making it a more sensitive tool for surveillance and monitoring of control programs."
4. Risk factor analysis: The association between STH infections and factors like latrine availability and handwashing is well-documented in the literature. The authors should contextualize their findings more critically, discussing how their results align with or diverge from previous studies in similar settings. Again, the multivariate analysis for S. mansoni risk factors is clear, but the odds ratios for some variables (e.g., swimming, irrigation) are high. The authors should consider potential confounding factors (e.g., frequency of water contact) that were not adjusted for.
As we were asked to critically analyse the association between STH infections and factors like latrine availability and hand washing, and to discuss how our results compare or differ from previous studies in similar settings, we addressed this in lines 445–472 of the revised manuscript as follows:

The lack of a latrine at home was identified as a significant risk factor for soil-transmitted helminth (STH) infections in this study. This finding highlights the strong connection between poor sanitation and the transmission of helminths. It aligns with extensive research showing that limited access to sanitation facilities, especially at the household level, plays a major role in driving STH infections (49–51). Without access to latrines, many individuals are forced to practice open defecation, resulting in the direct release of helminth eggs into the environment and promoting continued transmission. Having access to household latrines significantly reduces the risk of exposure to infectious helminth stages in soil and water by minimizing the dispersion of feces in the surrounding area. Notably, This finding aligns with previous research in the same region, which also identified open field defecation as a key factor contributing to the prevalence of soil-transmitted helminths (STH) (22). This study reinforces existing evidence by examining sanitation status at the household level instead of relying on broader community-level indicators. By doing so, it provides a deeper understanding of how individual risks are influenced by domestic infrastructure. This highlights the need for targeted public health interventions that extend beyond general sanitation promotion. It is essential to prioritize equal access to latrines and ensure their sustained use at the household level, especially in high-transmission areas where environmental contamination continues to pose a significant challenge.
"Hand hygiene practices, especially the failure to wash hands after using the latrine, were significantly associated with the prevalence of STH, which aligns with prior global studies (52–54). This highlights the importance of handwashing as a key factor in preventing the transmission of STH (55). In contrast to previous research in the same area, hand hygiene showed no significant effect (22). This divergence may be due to variations in study design, changes in hygiene behaviors over time, or previous underestimations of individual practices. Furthermore, recent improvements in sanitation infrastructure, in the absence of corresponding behavioral changes, could increase the relative impact of hand hygiene on the risk of infection. These findings underscore the complex interaction between environmental and behavioral factors that influence the transmission of soil-transmitted helminths (STH) and emphasize the

Powered by Editorial Manager® and ProduXion Manager® from Aries Systems Corporation

importance of ongoing local assessments.
We were advised to consider potential confounding factors, such as frequency of water contact; however, this variable was not included in the final analysis as it was removed after the pretest due to participants' poor recall.

5. Prevalence and intensity: The reported prevalence of S. mansoni (20.1% by Kato-Katz) is described as "moderate," but the WHO threshold for moderate prevalence is ≥10% and <50%. The authors should explicitly state how their findings align with WHO guidelines for mass drug administration (MDA). Again, the intensity classification (light, moderate, heavy) is based on WHO criteria, but the geometric mean egg count (139.39 EPG) suggests a skewed distribution. The authors should discuss whether this affects the interpretation of intensity.
As we were asked to explicitly state how the 20.1% prevalence of S. mansoni aligns with WHO guidelines for MDA, we note that this has already been addressed in the discussion section of the previously submitted manuscript. We were also asked whether the interpretation of infection intensity was affected by the skewed distribution when using arithmetic and geometric means. we addressed this in lines 401–404 of the revised manuscript as follows: "The noticeable discrepancy between the arithmetic and geometric means reflects the presence of a right-skewed distribution of egg counts. This pattern is consistent with the typical epidemiology of schistosomiasis, where a minority of individuals harbor high worm burdens, while the majority exhibit low to moderate infection levels (42).
6. Limitations: The cross-sectional design and purposive school selection are noted as limitations, but the authors should also address potential biases (e.g., seasonal variations in transmission, non-random sampling). Again, the use of a single stool sample for Kato-Katz may underestimate prevalence due to day-to-day variation in egg excretion. This should be discussed as a limitation.
Regarding the comments on the limitations section of the manuscript, we addressed this in lines 532–535 in the revised manuscript as follows: "This study has a few limitations: (i), the cross-sectional design provides a snapshot of S. mansoni and STH infection at a single point in time, which may not capture seasonal variations or fluctuations in infection rates over longer periods. (ii), the use of a single stool sample per participant for the KK technique may have led to underestimation of infection prevalence and intensity, given the known day-to-day variability in helminth egg excretion. (iii), the purposive selection of schools limits the generalizability of the findings to the larger community in the study area.
7. Minor Comments: (i) Abstract: The abstract is comprehensive but could be more concise. The methods section, for example, includes unnecessary details (e.g., SPSS version). Again, the conclusion should avoid redundancy (e.g., "necessity for additional control measures" is repeated). (ii) Tables and Figures: Table 1 (infection intensity) is clear but could be merged with Table 2 (socio-demographic factors) to streamline the presentation. Again, Figure 3 (helminth distribution) is informative but would benefit from a clearer legend (e.g., full parasite names instead of abbreviations). (iii) Writing and Clarity: Some sentences are overly long or awkwardly phrased (e.g., "The absence of a latrine at home emerged as a prominent risk factor for STH infection"). Editing for conciseness would improve readability. Again, the abbreviation "STH" is used throughout but should be defined at first mention in the abstract and main text. (iv) References: References are generally appropriate, but some are outdated (e.g., WHO guidelines from 2011). The authors should cite more recent WHO publications (e.g., 2022 guidelines). Again, a few references are incomplete (e.g., "Weekly epidemiological record Relevé épidémiologique hebdomadaire" lacks volume/issue details).
The minor comments have all been addressed in the revised manuscript, except for merging the tables, which we chose not to do because the current format is clearer and merging would make the presentation more complicated.
(i)Abstract: Data were processed in SPSS v26 using logistic regression, with p<0.05 considered statistically significant paraphrased to Data was analysed using SPSS and McNemar's chi-square test (line numbers 53 – 54). In conclusion, part necessity is deleted
(ii)We prefer to retain Table 1, which has been changed to Table 2 in the revised manuscript, as it aligns with the above explanation.
Figure 3 for helminthic distribution. We revised the figure and wrote the full name of the parasite rather than using the legend. See the revised Fig 3
(iii)Writing and Clarity: Once again, we revised the whole manuscript document for

Powered by Editorial Manager® and ProduXion Manager® from Aries Systems Corporation

	clarity, abbreviation consistency, and scientific parasite naming<br>(iv)We also revised the reference and made necessary corrections in the revised manuscript (line numbers 545 – 547)<br>____<br><br>While revising your submission, please upload your figure files to the Preflight Analysis and Conversion Engine (PACE) digital diagnostic tool, https://pacev2.apexcovantage.com/. PACE helps ensure that figures meet PLOS requirements. To use PACE, you must first register as a user. Registration is free. Then, login and navigate to the UPLOAD tab, where you will find detailed instructions on how to use the tool. If you encounter any issues or have any questions when using PACE, please email PLOS at figures@plos.org. Please note that Supporting Information files do not need this step.
**Additional Information:**	
**Question**	**Response**
**Financial Disclosure**<br><br>Enter a financial disclosure statement that describes the sources of funding for the work included in this submission. Review the submission guidelines for detailed requirements. View published research articles from *PLOS ONE* for specific examples.<br><br>This statement is required for submission and **will appear in the published article** if the submission is accepted. Please make sure it is accurate.<br><br>**Funded studies**<br>Enter a statement with the following details:<br>• Initials of the authors who received each award<br>• Grant numbers awarded to each author<br>• The full name of each funder<br>• URL of each funder website<br>• Did the sponsors or funders play any role in the study design, data collection and analysis, decision to publish, or preparation of the manuscript?<br><br>Did you receive funding for this work?	The author(s) received no specific funding for this work.
**Competing Interests**<br><br>Use the instructions below to enter a competing interest statement for this submission. On behalf of all authors, disclose any competing interests that could be perceived to bias this	There is no any competing interest for this research. All authors dead and agreed to submit this article to PLoS ONE journal. We read also the journal guideline

Powered by Editorial Manager® and ProduXion Manager® from Aries Systems Corporation

work—acknowledging all financial support and any other relevant financial or non-financial competing interests.<br><br>This statement is required for submission and **will appear in the published article** if the submission is accepted. Please make sure it is accurate and that any funding sources listed in your Funding Information later in the submission form are also declared in your Financial Disclosure statement.<br><br>View published research articles from *PLOS ONE* for specific examples.<br><br>**NO authors have competing interests**<br><br>Enter: *The authors have declared that no competing interests exist.*<br><br>**Authors with competing interests**<br><br>Enter competing interest details beginning with this statement:<br><br>*I have read the journal's policy and the authors of this manuscript have the following competing interests: [insert competing interests here]*<br><br>* typeset	
**Ethics Statement**<br><br>Enter an ethics statement for this submission. This statement is required if the study involved:<br><br>• Human participants<br>• Human specimens or tissue<br>• Vertebrate animals or cephalopods<br>• Vertebrate embryos or tissues<br>• Field research<br><br>Write "N/A" if the submission does not require an ethics statement.	Ethical clearance was obtained from Hawassa University, College of Medicine, and other Health Sciences Internal Review Board (IRB) with a reference number: IRB/172/16 E.C. A letter of support from Hawassa University School of Medical Laboratory was provided to the Sidama Regional State Health Bureau, as well as to the health and education offices in Tulla district and the directors of the selected schools. Participation was fully voluntary, with written informed consent obtained from the parents/guardians of the children enrolled. Assent was obtained from students aged ≥ 12 years, following parent/guardian consent. Schoolchildren who tested positive for any pathogenic intestinal parasites received appropriate treatment administered by qualified public health professionals. All data collected during the study were kept strictly confidential and used exclusively for research purposes. The study procedures were conducted in full compliance with relevant ethical guidelines and regulatory standards

General guidance is provided below. Consult the submission guidelines for detailed instructions. **Make sure that all information entered here is included in the Methods section of the manuscript.**<br><br>**Format for specific study types**<br><br>**Human Subject Research (involving human participants and/or tissue)**<br>• Give the name of the institutional review board or ethics committee that approved the study<br>• Include the approval number and/or a statement indicating approval of this research<br>• Indicate the form of consent obtained (written/oral) or the reason that consent was not obtained (e.g. the data were analyzed anonymously)<br><br>**Animal Research (involving vertebrate animals, embryos or tissues)**<br>• Provide the name of the Institutional Animal Care and Use Committee (IACUC) or other relevant ethics board that reviewed the study protocol, and indicate whether they approved this research or granted a formal waiver of ethical approval<br>• Include an approval number if one was obtained<br>• If the study involved *non-human primates*, add *additional details* about animal welfare and steps taken to ameliorate suffering<br>• If anesthesia, euthanasia, or any kind of animal sacrifice is part of the study, include briefly which substances and/or methods were applied<br><br>**Field Research**<br><br>Include the following details if this study involves the collection of plant, animal, or other materials from a natural setting:<br>• Field permit number<br>• Name of the institution or relevant body that granted permission	
**Data Availability**<br><br>Authors are required to make all data underlying the findings described fully	Yes - all data are fully available without restriction

available, without restriction, and from the time of publication. PLOS allows rare exceptions to address legal and ethical concerns. See the PLOS Data Policy and FAQ for detailed information.<br><br>A Data Availability Statement describing where the data can be found is required at submission. Your answers to this question constitute the Data Availability Statement and **will be published in the article**, if accepted.<br><br>**Important:** Stating 'data available on request from the author' is not sufficient. If your data are only available upon request, select 'No' for the first question and explain your exceptional situation in the text box.<br><br>Do the authors confirm that all data underlying the findings described in their manuscript are fully available without restriction?	
**Describe where the data may be found in full sentences. If you are copying our sample text, replace any instances of XXX with the appropriate details.**<br><br>• If the data are **held or will be held in a public repository**, include URLs, accession numbers or DOIs. If this information will only be available after acceptance, indicate this by ticking the box below. For example: *All XXX files are available from the XXX database (accession number(s) XXX, XXX.*).<br>• If the data are all contained **within the manuscript and/or Supporting Information files**, enter the following: *All relevant data are within the manuscript and its Supporting Information files.*<br>• If neither of these applies but you are able to provide **details of access elsewhere**, with or without limitations, please do so. For example:<br><br>*Data cannot be shared publicly because of [XXX]. Data are available from the XXX Institutional Data Access / Ethics*	data are all contained within the manuscript and/or Supporting Information files, enter the following: All relevant data are within the manuscript and its Supporting Information files.

Committee (contact via XXX) for researchers who meet the criteria for access to confidential data.<br><br>*The data underlying the results presented in the study are available from (include the name of the third party and contact information or URL).*<br>• This text is appropriate if the data are owned by a third party and authors do not have permission to share the data.<br><br>* typeset	
Additional data availability information:	

Powered by Editorial Manager® and ProduXion Manager® from Aries Systems Corporation

Manuscript
Click here to access/download;Manuscript;Un marked Manuscript.docx

Assessment of *Schistosoma mansoni* and Soil-transmitted Helminth Infections and the Diagnostic Performance of the Circulating Cathodic Antigen Test among Schoolchildren in Tulla District, Sidama Region, Southern Ethiopia.

Short title: *Schistosoma mansoni* Infection and Performance of its antigen test

Fitsum Getachew[1,2*], Demissie Assegu[1], Biniyam Kijineh[3] and Bamlaku Tadege[1*]

[1] School of Medical Laboratory Sciences, Hawassa University, Hawassa, Ethiopia.

[2] School of Medical Laboratory Sciences, Wolaita Sodo University, Wolaita, Ethiopia.

[3] School of Medical Laboratory Sciences, Wachemo University, Ethiopia, Hossana, Ethiopia.

*E-mail of corresponding author: bamlaku22tadege@gmail.com

Consent for publication

All authors have read the manuscript and consented to submit the manuscript.

Competing interests

All authors declare that they have no competing interests.

Authors' contributions

FG: conceptualization, data curation, formal analysis, investigation, methodology, writing – original draft; DA & BK: writing – review & editing, BT: conceptualization, supervision, writing – review & editing, data curation, and formal analysis.

Authors' addresses

fitsumgetachew6@gmail.com (FG)

bamlaku22tadege@gmail.com (BT)

demisissieasegu@yahoo.com (DA)

kijinehb@gmail.com (BK)

Funding

This study was funded by Hawassa University

Abstract

Background: Soil-transmitted helminths (STH) and *Schistosoma mansoni* pose significant public health challenges in areas with poor sanitation. While *S. mansoni* infection leads to chronic illness and mortality, research on its intensity remains limited in the study area. Diagnosis traditionally relies on the Kato-Katz method, though the POC-CCA urine test offers a rapid alternative. This study examined the prevalence, intensity, and risk factors of *S. mansoni* and Soil-transmitted helminth infections among schoolchildren while evaluating the diagnostic performance of the POC-CCA test.

Methods: A school-based cross-sectional study was conducted in the Tulla district, Sidama region, among 477 students (April–June 2024). The Schoolchildren were recruited using a purposive sampling technique. Socio-demographic data were collected using pre-structured questionnaires. A single stool sample was collected from each study participant and processed for the detection of helminth eggs using the Kato-Katz (KK) technique. Additionally, urine samples were tested with the Point of Care Circulating Cathodic Antigen Test (POC-CCA), which detects Schistosoma antigens.

Result: The overall prevalence of *Schistosoma mansoni* was 36.5% (20.1% by KK and 34.4% by POC-CCA). The prevalence of STH was 48.8%. The leading parasite was *Ascaris lumbricoides (*34%*),* followed by *Trichuris trichuria* (8.2%) and hookworm (5.7%). Other helminths included Taenia species 1.3%, *Hymenolepis nana* 0.6%, and *Enterobius vermicularis* 0.2%. Infection intensity of *S. mansoni* was classified as light 34.4%, moderate 56.2%, and heavy 9.4%. Involvement in irrigation, swimming, bathing, and school location were significantly associated with *S. mansoni* infection. School location, educational status, lack of

latrine, hand washing practice and fingernail trimming practice were significantly associated with STH infections. The POC-CCA rapid test had a significantly higher sensitivity of 89.6% (McNemar test $\chi^2 m = 52.3$, $p < 0.001$) compared to the Kato-Katz technique.

Conclusions: Moderate *S. mansoni* and STH infection and poor hygiene practice underscore for additional control measures. Incorporating POC-CCA testing alongside the KK method could optimize diagnostic accuracy and improve disease management outcomes.

Keywords: Prevalence, Infection intensity, *Schistosoma mansoni,* Soil-transmitted helminth, Diagnostic performance, Kato-Katz technique, Point-of-care circulating cathodic antigen.

Introduction

Background

Schistosoma and soil-transmitted helminth (STH) are worm infections that belong to the group of neglected tropical diseases (NTDs) (1). Schistosomiasis is a water-based, vector-borne parasite transmitted indirectly through freshwater snails (2). People who live in areas with inadequate water and sanitation systems are susceptible to this most common and deadly infection (3). Globally, schistosomiasis is recognized as endemic in 78 low and middle-income countries in tropical and subtropical areas, estimated to affect >251 million people (4). Furthermore, 243 million people require preventative chemotherapy, including 111 million school-age children, of which 226 million are in Africa (5). It causes significant morbidity, with an estimated 1.4-3.3 million disability-adjusted life years (DALYs) lost annually due to its health impacts (6).

STHs are intestinal parasitic worms transmitted to humans through fecally contaminated soil (7). The main STHs that affect humans are *Ascaris lumbricoides* (A. *lumbricoides*), *Trichuris trichuria (*T. *trichuria), Necator americanus, and Ancylostoma duodenale* (8). These parasites cause gastrointestinal infections and are most commonly found in tropical and subtropical regions, particularly impacting impoverished nations where schoolchildren are especially vulnerable (9).

Globally, an estimated 1.5 billion people are infected with STHs, including 654 million school-aged children living in areas where these parasites are prevalent (10). The Burden of *Schistosoma* and STHs leads to various health issues, including gastrointestinal bleeding, nutrient malabsorption, loss of appetite, anemia due to the depletion of iron and other essential proteins, growth retardation, impaired cognitive development, school absenteeism, and disability-adjusted life-years lost (11–13).

To control *Schistosoma* and STH-attributable morbidity, WHO recommends three interventions, including periodical administration of anthelmintic drugs to kill the worms, health education, and improved water, hygiene, and sanitation (WASH) to prevent worm re-infection. Today, mass drug administration (MDA) programs remain the mainstream strategy. During these programs, a single oral dose of albendazole (400 mg) or mebendazole (500 mg) for STH and praziquantel for

S*chistosoma* are periodically administered to the at-risk groups (14). Although these deworming programs have been successful in reducing global *Schistosoma* and STH-attributable morbidity, re-infection perpetuates in the absence of improved WASH and health education (15).

Intestinal *Schistosoma* is diagnosed by microscopic detection of parasite eggs in stool using the Kato-Katz (KK) technique, which is widely used in epidemiological surveys (16). However, its diagnosis is challenging in low-endemic areas where prevalence and worm burden are reduced, due to factors like uneven egg distribution in stool samples, daily variations in egg excretion, and random distribution effects (17,18). On the other hand, the point-of-care (POC) cassette test is a relatively new diagnostic tool that detects circulating cathodic antigens (CCA) in urine samples, offering higher sensitivity and operational benefits (18,19). The study found that POC-CCA was more sensitive (86% vs. 62%) but less specific (72% vs. ~100%) compared to multiple KK smears from a single stool sample. Despite this, the study noted that only 1% of POC-CCA tests in non-endemic areas were false positives, indicating that the specificity of POC-CCA might be underestimated. Multivariable modeling also suggested that POC-CCA was significantly more sensitive than KK for detecting low infection intensities (<100 eggs/gram of stool) (20). However, before the POC-CCA assay can be fully utilized for monitoring and evaluating control programs, it is essential to assess its performance in endemic settings (21).

Therefore, this study aimed to evaluate the prevalence of *Schistosoma mansoni* (*S. mansoni*) and STH infections and intensity of *S. mansoni* in elementary school-aged children, as well as the associated risk factors to evaluate ongoing control intervention. Additionally, it sought to assess the performance of the POC-CCA test compared to the KK method for diagnosing *S. mansoni*.

Materials and Methods

Study design and setting

A school-based cross-sectional study was conducted between April and June 2024 among four governmental elementary school children in the Tulla district. The study was conducted in four elementary schools in the Tulla district, Sidama region in Southern Ethiopia. The district is situated along the shore of Lake Hawassa and the Tikur Wuha River, approximately 289 kilometers south of Addis Ababa, the capital city of Ethiopia. The altitude is 1800 meters above

sea level, with an annual rainfall of 1123 mm and temperatures ranging from 13 to 27°C. Lake Hawassa is the primary water source for the residents of Hawella Tulla, catering to their domestic, agricultural, fishing, and other needs. According to ECSA, the district has a total population of 129,507, comprising 65,018 males and 64,499 females. The district has various social service institutions, including 27 health facilities and 69 schools. The health facilities consist of 1 primary hospital, 6 health centers, 18 rural health posts, 2 urban health posts, and 1 non-governmental health center. In terms of education, the district includes 21 primary schools, 5 secondary schools, 6 non-governmental primary schools, and 37 non-governmental kindergarten schools (Fig 1).

Fig 1: Map of the study site. The figure in the left corner depicts Lake Hawassa and the study district, while the upper right corner shows Ethiopia. The figure at the bottom right displays the map of the Sidama region 2024 (The map is prepared using ArcGIS 10.8 (ESRI, Redlands, CA USA) after obtaining country shape files from the humdata.org website (http://data.humdata.org/).

Population and enrollment criteria

The source population was all schoolchildren in the Tulla district, Sidama region, southern Ethiopia. The study population included schoolchildren who were enrolled in the selected schools during the study period and met the established inclusion criteria (who had both parental or guardian informed consent and their assent to participate) and exclusion criteria (who had received anti-helminthic treatment within one month before the recruitment period, or who were unable to provide stool and urine samples were excluded from the study).

Sample size and sampling technique

To determine the sample size, a single population proportion formula was used based on the following assumptions: a prevalence of *S. mansoni* of 31% (22), with a 5% margin of error and a 95% confidence level. As a result, the calculated sample size was 329. Considering the design or cluster effect between schools, the determined number was multiplied by 1.5, and then 5% was added for the non-response rate, giving the final sample size of 484.

To recruit participants for this study, we utilized a purposive sampling technique. In the initial stage, Bushulo and Finchawa, located near the coasts of Lake Hawassa in the Tulla sub-city, were selected alongside two primary schools farther from the lake, Gemeto and Tulla, with student populations of 1,820, 686, 2,050, and 1,439, respectively. Then, the predetermined sample size of 484 was proportionally allocated to the schools, resulting in 147 participants from Bushulo, 55 from Finchawa, 116 from Gemeto, and 166 from Tulla. The students' registration lists served as the sampling frame, and a systematic random sampling technique was employed to select the study subjects from each school. The k-value (sampling interval) was determined by dividing the total number of study participants in each school by the number of students to be selected. The students were then systematically selected at regular intervals (every 12th student), stratified by age (5–19 years) and grade levels (1–4 and 5–8) until the predetermined sample size was achieved. In cases where the selected student was absent from school, three repeated visits were made. When a student missed after three visits, he/she considered non-respondents.

Dependent and independent variables

The dependent variables were prevalence, infection intensity, and diagnostic performance of *Schistosoma mansoni* infection. The independent variables consisted of socio-demographic characteristics such as gender, age, and educational status of both parents and family's occupation. Additionally, grade level and the schools attended were considered. The distance of the home from the lake and practice-related factors were also explored, focusing on habits such as swimming, fishing, shoe-wearing, washing clothes, hand washing before meals and after latrine, open-field defecation, trimming finger nail, bathing, and involvement in irrigation activities. Environmental factors considered included the availability of a latrine at home. Clinical symptoms of the disease were also assessed among study participants.

Data collection methods

Interview

The data was collected using a structured questionnaire. Two trained nurses conducted home-to-home visits to interview the parents or guardians of the children, utilizing the KOBO Collect digital tool. A senior expert and the principal investigator supervised the data collection process, and

regular meetings were held to address any challenges and ensure adherence to established protocols and standards.

Laboratory investigation

Fecal and urine samples were collected in a screw-capped, dry, and clean container pre-labeled with the participant's unique identification number. The collected stool samples were transported within an hour of collection in suitable cool boxes at temperatures between 4°C and 6°C for subsequent examination. Samples from Gemeto and Tulla Elementary School were transported to Tulla Hospital, while samples from Bushulo and Finchawa Elementary School were transported to the Bushulo Mother and Child Health Specialty Center. Sample collection and transportation quality were supervised by the investigator to ensure adherence to procedures and the accuracy of the data results.

Stool sample processing using the Kato-Katz technique

The stool samples were examined using the KK technique to detect parasite eggs. A portion of the sample was processed using a calibrated template holding 41.7 mg of stool. Double slides were prepared for each stool sample. The number of eggs was counted from the double KK microscopic slides, and the average count was converted to eggs per gram of feces (EPG) using a correction factor of 24. Based on WHO cut-off values, the infection intensity was categorized as light (EPG 1-99), moderate (EPG 100-399), or heavy (EPG >400) (23).

Urine sample processing using POC-CCA rapid test

Each urine sample was processed immediately at the schools using the POC-CCA test, following the manufacturer's instructions (24). This on-site processing allowed for prompt results and minimized potential delays or degradation of the samples. 100μL of urine was transferred to the circular well of the test cassette and allowed to absorb completely into the specimen pad. The test results were read after 20 minutes of assay development. In cases of discrepancies, the tests were re-examined to ensure accuracy. The POC-CCA tests were scored as negative or positive based on the formation and intensity of the control band. Tests were considered invalid if an internal

control band did not appear or if results were not read after more than 25 minutes. In such cases, samples were re-run with a new cassette and scored as necessary.

Data quality assurance

The questionnaire was initially developed in English and then translated into Amharic and Sidamu Afoo, the local language of the participants. It was then translated back into English to ensure consistency. A pre-test was conducted on 5% of the sample size at Tullo Primary School, which was not part of the selected schools for the final study. The questionnaire was subsequently modified based on the findings from the pre-test. The completeness of data collection was overseen daily by the assigned supervisor.

The senior laboratory technologist provided refresher training to laboratory professionals. Standard operating procedures (SOPs) for pre-analytical, analytical, and post-analytical procedures were implemented throughout sample analysis. To guarantee quality control as an internal quality control measure, 10% of the slides were randomly selected and re-examined by a senior laboratory technologist at Tulla Hospital and Bushulo Mother and Child Health Specialty Centre and by another experienced technologist at HUCSH as part of an external laboratory evaluation. Both stool and urine samples were collected between 09:00-12:00 a.m. and 03:00-05:00 a.m. local time each day of the survey.

Data analysis

Data was entered into the KOBO Collect digital tool and analyzed using SPSS version 26 software. The prevalence of *S. mansoni* and STH infection was presented as a percentage, while infection intensity was classified as low, moderate, or heavy according to WHO guidelines (4). The mean egg count was reported as a geometric mean. A logistic regression model was employed to identify potential determinants of *S. mansoni* and STH infections and assess the strength of associations. Crude odds ratios (COR) from bivariate analysis were calculated to identify significant variables associated with the outcome. To control for confounding effects,

factors with a p-value ≤ 0.25 in bivariate analysis were included in multivariable logistic regression, allowing for the determination of adjusted odds ratios (AOR). A p-value < 0.05 was considered statistically significant. The diagnostic performance of the POC-CCA was assessed by calculating sensitivity, specificity, and other relevant metrics, with these tests regarded as reference standards (25). Kappa statistics were utilized to quantify agreement between the two diagnostic methods, and the concordance rate was calculated as the proportion of cases yielding identical results. McNemar's chi-square test was applied to analyze paired proportions of positive and negative results to investigate detection rate differences further.

Ethical consideration

Ethical clearance was obtained from Hawassa University, College of Medicine, and other Health Sciences Internal Review Board (IRB) with a reference number: IRB/172/16 E.C. A letter of support from Hawassa University School of Medical Laboratory was provided to the Sidama Regional State Health Bureau, as well as to the health and education offices in Tulla district and the directors of the selected schools. Participation was fully voluntary, with written informed consent obtained from the parents/guardians of the children enrolled. Assent was obtained from students aged ≥ 12 years, following parent/guardian consent. Schoolchildren who tested positive for any pathogenic intestinal parasites received appropriate treatment administered by qualified public health professionals. All data collected during the study were kept strictly confidential and used exclusively for research purposes. The study procedures were conducted in full compliance with relevant ethical guidelines and regulatory standards

Result

Socio-demographic characteristics of the study participants

A total of 484 students were invited to participate in the study, of whom 477 met the inclusion criteria and agreed to take part, resulting in a response rate of 98.5%. The study sample included 208 (43.6%) males and 269 (56.4%) females. Participants' ages ranged from 5 to 19 years, with a mean age of 11.94 years and a standard deviation of 2.76 years. Most of the study participants (62.7%) were 10-14 years old. The majority of students reside within 1 km of a lake (66.5%) and are under the care of their private (daily laborer) parents or guardians (41.7%). The students were selected from 4 primary schools: Bushulo 143 (30%), Finchawa 53 (11.1%), Gemeto 116 (24.3%), and Tulla 165 (34.6%) **(Table 1).**

Table 1: The distribution of *Schistosoma mansoni* infection by socio-demographic and other factors among schoolchildren in Tulla district, Sidama region, Southern Ethiopia 2024 (n = 477).

Variables	*S. mansoni* Positive %	Negative %	Total %	COR (95% CI)	p-value	AOR (95%CI)	p-value
**Gender**							
Male	52(25.0)	156(75.0)	208(43.6)	1		1	
Female	44(16.4)	225(83.6)	269(56.4)	0.587(0.374-0.920)	0.020	0.607(0.359-1.027)	0.063
Age							
5-9	7(9.1)	70(90.9)	77(16.1)	1			
10-14	67(22.4)	232(77.6)	299(62.7)	2.888(1.268-6.578)	0.012	2.118(0.841-5.330)	0.111
15-19	22(21.8)	79(78.2)	101(21.2)	2.785(1.122-6.914)	0.027	1.442(0.495-4.198)	0.502
Name of School							
Bushulo	52(36.4)	91(63.6)	143(30)	6.163(3.234-11.746)	0.001	4.685(1.869-11.742)	**0.001****
Finchawa	13(24.5)	40(75.5)	53(11.1)	3.505(1.526-8.051)	0.003	1.444(0.486-4.297)	0.509
Gemeto	17(14.6)	99(85.4)	116(24.3)	1.852(0.874-3.926)	0.108	1.528(0.662-3.525)	0.320
Tulla	14(8.5)	151(91.5)	165(34.6)	1			
Educational status of the father							
No formal education	17(18.5)	75(81.5)	92(19.3)	1		1	
Primary school	58(26.0)	165(74.0)	223(46.75)	1.551(0.846-2.841)	0.156	1.420(0.690-2.921)	0.341
Educational status of the mother							
No formal education	3(7.0)	40(93.0)	43(9)	1		1	
Primary school	62(23.2)	205(76.8)	267(56)	4.033(1.206-13.485)	0.024	3.391(0.889-12.937)	0.074
Secondary school	25(18.8)	108(81.2)	133(28)	3.086(0.883-10.787)	0.078	2.178(0.535-8.859)	0.277
College and above	6(17.7)	28(82.3)	34(7)	2.857(0.658-12.397)	0.161	2.384(0.414-13.719)	0.331
Distance of residential area							
Within 1km	49(30.6)	111(69.4)	160(33.5)	1		1	
More than 1km	47(14.8)	270(85.2)	317(66.5)	0.394(0.250-0.623)	0.001	1.194(0.615-2.318)	0.601
Bathing in the lake							
Yes	43(28.5)	108(71.5)	151(31.7)	2.051(1.295-3.248)	0.002	1.804(1.048-3.107)	**0.033***
No	53(16.3)	273(83.7)	326(68.3)	1		1	

Swimming habit							
Yes	50(31.9)	107(68,1	157(32.9)	2.783(1.780-4.403)	0.001	2.407(1.430-4.052)	**0.001****
No	46(14.4)	274(85.6)	320(67.1)	1			
Fishing							
Yes	20(35.1)	37(64.9)	57(12)	2.447(1.345-4.449)	0.003	1.135(0.533-2.417)	0.743
No	76(18.1)	344(71.9)	420(88)	1			
Washing clothes along the lake shore							
Yes	19(30.6)	43(69.4)	62(13)	1.940(1.071-3.513)	0.029	2.016(0.934-4.350)	0.074
No	77(17.3)	338(82.7)	415(87)	1			
Involvement in irrigation							
Yes	44(37.0)	75(63.0)	119(25)	3.452(2.148-5.547)	0.001	2.429(1.377-4.287)	**0.002***
No	52(14.5)	306(85.5)	358(75)	1			

AOR, Adjusted Odds Ratio; CI, Confidence interval; COR, Crude Odds Ratio; *, Significant at $p < 0.05$.

Prevalence of *Schistosoma mansoni* infection

The combined results of the 2 diagnostic methods showed an overall prevalence of *S. mansoni* to be 36.5%, with 20.1% (96/477) detected using the KK technique and 34.4% (164/477) detected using the POC-CCA test. The highest prevalence of *S. mansoni* infection, 30.5%, was observed among students attending schools near the lake (Bushulo and Finchawa). Overall, the prevalence of *S. mansoni* infection was highest among students aged 10 - 14 years (Table 1).

Infection intensity of *Schistosoma mansoni* infection

Out of 174 *S. mansoni* infected study participants, 96 tested positive by the KK technique using a standard amount of stool that enables the quantification of ova per gram of stool. The minimum and maximum eggs per gram of stool were 24 and 672, respectively. Likewise, the arithmetic and geometric mean values were 189.75 and 139.39, respectively. According to WHO classification, among 96 students, 33 (34.4%) had light intensity, 54 (56.2%) had moderate intensity, and 9 (9.4%) had a heavy intensity of *S. mansoni* infection (Fig 2).

Fig 2: The prevalence of *S. mansoni* infection intensity across 477 schoolchildren in Tulla district Sidama Region, southern Ethiopia, 2024

As shown in Table 2, moderate infection with *S. mansoni* among males accounted for 53.9%, followed by 34.6% with light infection and 11.5% with heavy infection. Among individuals in the 15 to 19 age group, most infections were of moderate intensity (72.7%), followed by light intensity (18.2%) and heavy intensity (9.1%). When comparing different schools, more than half of the infections reported by students from Bushulo Primary School were of moderate intensity (61.5%), while 27% were classified as light intensity and 11.5% as heavy intensity. In contrast, the majority of infections among students from Tulla Primary School were of light intensity (64.3%) and moderate intensity (35.7%) **(Table 2)**

306 Table 2: Gender, age, and school-based variation in S. mansoni infection intensity among schoolchildren in Tulla District Sidama Region southern Ethiopia 2024 (n = 477).

Variable	N	Intensity of infection n (%)		
		Light	Moderate	Heavy
**Gender**				
Male	52	18(34.6)	28(53.9)	6(11.5)
Female	44	15(34.1)	26(59.1)	3(6.8)
**Age**				
5-9	7	2(28.6)	5(71.4)	0(0)
10-14	67	27(40.3)	33(49.25)	7(10.45)
15-19	22	4(18.2)	16(72.7)	2(9.1)
**Schoolchildren**				
Bushulo	52	14(27)	32(61.5)	6(11.5)
Finchawa	13	4(31)	7(53.8)	2(15.2)
Gemeto	17	6(35.3)	10(58.8)	1(5.9)
Tulla	14	9(64.3)	5(35.7)	0(0)

Key - N: refers to the total number of infected; whereas, n: refers to the number of infected according to intensity classes.

Potential associated risk factors of *S. mansoni* infections.

In the bivariate logistic regression analysis, activities such as washing clothes 13% (62/477), bathing 31.7% (151/477), swimming 32.9% (157/477), fishing 11.9% (57/477), and engaging in irrigation activities 24.9% (119/477), were identified as potential risk factors of *S. mansoni* infection with a p-value ≤ 0.25. Socio-demographic factors such as gender, age, school (Bushulo), educational status of the father (primary school), educational status of the mother (primary school), and distance of residential area from the lake were also potential predictors of *S. mansoni* infection (Table 1).

Multivariate logistic regression analysis was applied to avoid confounding factors and only students engaged in irrigation were 2.4 times more likely to be infected than those not involved (AOR = 2.43; 95% CI: 1.38-4.29, P = 0.002), schoolchildren with swimming habits exhibited a 2.4-fold increase in the likelihood of infection (AOR = 2.41; 95% CI: 1.43-4.34, P = 0.001) and participants who bathed in the lake were 1.8 times more likely to be infected than those who did not engage in this activity (AOR = 1.80; 95% CI: 1.05-3.12, P = 0.033). A school-wide analysis revealed that students attending Bushulo primary school were 4.7 times more likely to contract *S. mansoni* infection compared to their peers at Tulla primary school (AOR = 4.68; 95% CI: 1.87-11.74, P = 0.001) (Table 1).

Prevalence of soil transmitted helminthic infections

Aside from *S. mansoni*, the prevalence of intestinal helminths among the schoolchildren was 233 (48.8%). The most common STH was *Ascaris lumbricoides* 162 (34%), followed by *Trichuris trichuria* 39 (8.2%) and hookworm 27 (5.7%). Other helminth parasites included Taenia species 6 (1.3%), *Hymenolepis nana* 3 (0.6%), and *Enterobius vermicularis* 1 (0.2%) **(Fig 3).**

Fig 3: The prevalence of intestinal helminthic infections across 477 school children in Tulla district Sidama Region, southern Ethiopia, 2024

Potential associated risk factors of Soil transmitted helminthic infections.

In the bivariate logistic regression analysis, both hygiene-related practices and socio-demographic factors were identified as potential risk factors for intestinal helminthic infections. Household latrine availability (61%, 105/477), hand washing after latrine use (52.8%, 227/477), fingernail trimming (56.4%, 194/477), and hand washing before meals (53.4%, 196/477) were all associated with STH infections, with a p-value ≤ 0.25. Additionally, socio-demographic factors, including attending Tulla Elementary School, grade level, father's educational status, and family occupation (farmer), emerged as candidate predictors **(Table 3).**

Table 3: Univariate and multivariate logistic regression analyses for socio-demographic and other potential risk factors associated with soil transmitted helminthic infections among school children in Tulla district, Sidama region, Southern Ethiopia 2024 (n = 477).

Variables	STH Positive %	Negative %	Total %	COR (95% CI)	p-value	AOR (95%CI)	p-value
Gender							
Male	97(46.6)	111(53.4)	208(43.6)	1			
Female	136(50.6)	133(49.4)	269(56.4)	1.170(0.814-1.681)	0.395		
Age							
5-9	38(49.4)	39(50.6)	77(16.1)	1			
10-14	151(50.5)	148(49.5)	299(62.7)	1.047(0.634-1.728)	0.857		
15-19	44(43.6)	57(56.4)	101(21.2)	0.792(0.437-1.437)	0.443		
School							
Bushulo	76(53.2)	67(46.8)	143(30)	1		1	
Finchawa	28(52.8)	25(47.2)	53(11.1)	0.987(0.525-1.857)	0.969		
Gemeto	54(46.5)	62(53.5)	116(24.3)	0.768(0.470-1.254)	0.292		
Tulla	75(45.5)	90(54.5)	165(34.6)	0.735(0.469-1.151)	0.178	0.469(0.256-0.861)	**0.015***
Grade level							
1-4	118(48.2)	127(51.8)	245(51.4)	1		1	
5-8	126(54.3)	106(45.7)	232(48.6)	1.279(0.893-1.834)	0.180	0.848(0.495-1.453)	0.548
Educational status of father							
No formal education	55(59.8)	37(40.2)	92(19.3)	3.964(1.420-11.066)	0.009	4.161(1.276-13.570)	**0.018***
Primary school	115(51.6)	108(48.4)	223(46.7)	2.840(1.072-7.523)	0.036	2.957(0.991-8.818)	0.052
Secondary education	57(40.7)	83(59.3)	140(29.4)	1.831(1.072-7.523)	0.234	1.616(0.547-4.775)	0.385
College and above	6(27.3)	16(72.7)	22(4.6)	1		1	
Occupation of family							
Civil servant	6(31.6)	13(68.4)	19(4)	1		1	
Merchant	56(41.5)	79(58.5)	135(28.3)	1.536(0.550-4.285)	0.412		
Farmer	63(50.8)	61(49.2)	124(26)	2.238(0.799-6.264)	0.125	0.199(0.013-3.028)	0.245
Private (Daily laborer)	108(54.3)	91(45.7)	199(41.7)	2.571(0.940-7.037)	0.066	0.262(0.017-3.932)	0.332
Latrine availability at home							
Yes	128(42)	177(58)	305(63.9)	1		1	
No	105(61)	67(39)	172(36.1)	0.461(0.315-0.676)	0.001	0.626(0.393-0.996)	**0.048***

Hand washing after latrine							
Yes	6(12.8)	41(87.2)	47(9.9)	1		1	
No	227(52.8)	203(47.2)	430(90.1)	0.131(0.54-0.315)	0.001	0.199(0.072-0.551)	**0.002***
Shoe wearing							
Yes	198(49.25	204(50.75)	402(84.3)	1			
No	35(46.7)	40(53.3)	75(15.7)	0.902(0.550-1.477)	0.681		
Finger nail trimming							
Yes	39(29.3)	94(70.7)	133(27.9)	1		1	
No	194(56.4)	150(43.6)	344(72.1)	0.321(0.209-0.493)	0.001	0.413(0.250-0.682)	**0.001***
Hand washing before meal							
Yes	37(33.6)	73(66.4)	110(23)	1		1	
No	196(53.4)	171(46.6)	367(77)	0.442(0.283-0.890)	0.001	0.585(0.337-1.015)	0.058
Open field defecation							
Yes	24(48)	26(52)	50(10.5)	1.039(0.578-1.867)	0.899		
No	209(48.9)	218(51.1)	427(89.5)	1			

Key - AOR: Adjusted Odds Ratio, CI: Confidence interval, COR: Crude Odds Ratio, *: Significant at $p < 0.05$, STH: Soil-transmitted helminths.

In the multivariate logistic regression analysis, several socio-demographic and behavioral factors were found to significantly influence the prevalence of STH infections among schoolchildren. Students from Tulla Elementary School had 0.47 times lower odds of STH infection compared to their counterparts at Bushulo Elementary School (AOR = 0.469; 95% CI: 0.256-0.861, P = 0.015). Specifically, children whose fathers had no formal education were found to be 4.2 times more likely to be infected than those whose fathers had completed college or higher education (Adjusted Odds Ratio = 4.161; 95% Confidence Interval: 1.276-13.570, P = 0.018).

The presence of latrines at home also significantly influenced infection rates, with children having access to latrines exhibiting 0.63 times lower odds of infection than those without such facilities (AOR = 0.626; 95% CI: 0.393-0.996, P = 0.048). Furthermore, hygiene practices emerged as critical determinants, as children who washed their hands after using the latrine had a remarkable 0.2 times lower odds of contracting STH infections compared to those who did not (AOR = 0.199; 95% CI: 0.072-0.551, P = 0.002). Similarly, regular nail trimming was associated with a significant reduction in infection risk, with children who trimmed their nails having 0.4 times lower odds of STH infection than those who did not (AOR = 0.413; 95% CI: 0.250-0.682, P = 0.001) (Table 3).

Detection and performance evaluation of the POC-CCA test

Out of 477 samples examined, the POC-CCA rapid test identified 86 (18.0%) true positives, 303 (63.5%) true negatives, and 10 (2.1%) discrepancies (false negatives) compared to the KK technique. Using KK as the gold standard or ‘reference test’ (25), the POC-CCA sensitivity was 89.6% (95% CI: 83.5-95.7) and specificity 79.6% (95% CI: 75.6-83.6) **(Table 4)**

Table 4: Performance of POC-CCA test using Kato-Katz as a gold standard across 477 school children in Tulla district Sidama Region souther Ethiopia 2024

**Method**	**Result**	**KK (Gold standard)**		**Sensitivity % (95% CI)**	**Specificity % (95% CI)**	**NPV % (95[illegible] CI)**	**PPV % (95[illegible] CI)**	**LR^{-} % (95[illegible] CI)**	**LR^{+} % (95[illegible] CI)**
		**Pos**	**Neg**						
**POC-CCA**	**Pos**	**86**	**78**	**89.6 (80-99.2)**	**79.6 (73.9-85.[illegible]**	**96.8 (90.7-100)**	**52.4 (45.2-59.6)**	**0.131 (0.05[illegible] 0.197)**	**4.39 (3.93-5.10)**
	**Neg**	**10**	**303**						

Key - KK: Kato-Katz POC-CCA: Point of Care Circulating Cathodic Antigen, NPV: Negative Predictive value, PPV: Positive Predictive value, NLR^{-}: Negative Likely hood ratio, LR^{+}: Positive Likely hood ratio, Pos: Positive, Neg: Negative.

The agreement between the two diagnostic methods was quantified using Cohen's Kappa statistics which yielded a kappa value of 0.55. Furthermore, the overall concordance rate between the KK and POC-CCA was 81.5%. However, to assess the statistical significance of the differences in results between the two tests, we employed the McNemar Chi-square test. The result revealed a value of 52.3

Discussion

This study aimed to assess the prevalence, intensity, and associated risk factors of *S. mansoni* and to evaluate the diagnostic performance of the POC-CCA rapid test among schoolchildren in the Tulla district of the Sidama region in southern Ethiopia. The prevalence of *S. mansoni* infection was 20.1% (95% CI: 16.8-23.7) using the KK technique. This indicates the need for biannual deworming of schoolchildren in the study area with a moderate prevalence (≥ 10% but < 50%) according to WHO guidelines (4). This result aligns with previous studies from Jimma, 20.2% (26), Kersa, 19.9% (27), and Mekelle, 23.9% (28) in Ethiopia. Furthermore, the prevalence reported in this study is consistent with findings from Egypt, 19.1% (29), and Angola, 21.2% (30). However, it was higher than rates observed in Jiga at 15.2% (31), Guangua 12.6% (32), and Wondo districts 11.4% (33).

The prevalence in this study is lower than findings from Lake Tana in northwest Ethiopia, 34.9% (34), Jimma town, Ethiopia, 28.7% (35). The prevalence of this study is lower than rates reported in Maksegnit, Debark, Sanja, and Chuahit districts in northwest Ethiopia at 33.5% (36), Abbey and Didessa valleys 53.9% (37), and Wolaita southern Ethiopia 81.3% (38), Kenya, 53.7% (39), Congo, 90.2% (40), and Tanzania, 90.6% (41). The variations in the prevalence of S. mansoni infection across different regions may be attributed to several factors related to human behavior towards contaminated water sources, socioeconomic conditions and the rate of reinfection. These conditions include living environments, education levels, occupations, and the immune status of the individuals being studied. Furthermore, climate change may also influence human exposure to schistosomiasis. Environmental factors may also impact disease transmission variation across different regions include the proximity to snail habitats, the construction of dams, land cover

(particularly elevation), rainfall patterns, seasonal land surface temperatures, and the presence of flooded agricultural areas. Additionally, it is crucial to consider differences in sample sizes, study designs and diagnostic methods applied.

In the present study, out of 174 participants infected with *S. mansoni*, 96 were confirmed positive using the KK technique, a standard diagnostic method valued for its ability to quantify EPG of stool. The intensity of infection ranged from 24 to 672 EPG, with an arithmetic mean of 189.75 and a geometric mean of 139.39. The noticeable discrepancy between the arithmetic and geometric means reflects the presence of a right-skewed distribution of egg counts. This pattern is consistent with the typical epidemiology of schistosomiasis, where a minority of individuals harbor high worm burdens, while the majority exhibit low to moderate infection levels (42).

The moderate infection intensity reported in this study is consistent with findings from various regions in Ethiopia (37,43) and in Tanzania (41). This difference might be due to repeated exposure of schoolchildren to water bodies infested with the infective stage. The intensity of *S. mansoni* infection was found to be slightly higher in male students compared to female students, which is consistent with findings from Kemissie and Wondo Genet (43) and Jimma Town (44) in Ethiopia. The observed difference in proportion might be linked to male students' greater exposure to outdoor activities like fishing, swimming, and irrigation compared to female students.

A significant association was found between *S. mansoni* infection and schoolchildren who engaged in swimming and bathing activities in the lake, with infection risks being 2.4 times higher for swimming and 1.8 times higher for bathing than those who did not partake in these habits. This association between *S. mansoni* infection and swimming habits among

schoolchildren aligns with previous studies (22,32) and is further supported by studies linking bathing habits to infection (34).

Additionally, a strong association was noted between *S. mansoni* infection and participation in irrigation activities, with students involved in irrigation being 2.4 times more likely to be infected than those not engaged in such activities, consistent with prior findings (22,32). Notable differences in infection rates were observed among students across the various schools in the study areas. Children attending Bushulo Primary School were 4.7 times more likely to be infected with Schistosoma than their peers at Tulla Primary School. This finding aligns with previous studies (22,34). This might be associated with students' residence distance variation from lakes and other water bodies.

The study area displayed a moderate prevalence of STH infections among schoolchildren despite them being part of the deworming target group. Deworming programs have successfully decreased the global morbidity attributed to STHs (45), but the risk of re-infection persists in the absence of improved water, sanitation, and hygiene WASH practices and health education (46). A systematic review and meta-analysis estimated that three months after deworming, the prevalence of *Ascaris lumbricoides*, *Trichuris trichuria*, and *hookworms* reached 26%, 36%, and 30% of their pre-treatment levels, respectively. By one-year post-treatment, the prevalence rates approached their pre-treatment levels again, with *A. lumbricoides* at 94%, T. trichuria at 82%, and *hookworm* infections at 57% (47). The occurrence of re-infection highlights the importance of a comprehensive approach to controlling STH. This is especially crucial given the increasing interest in ultimately halting STH transmission in specific geographic regions (48). The current WHO guidelines recommend the evaluation of any STH infections in school-age children after five to six years of periodic chemotherapy using the standard Kato-Katz thick smear (49).

The findings of this study also provide critical insights into the socio-demographic and environmental determinants associated with STH infections among school children. This research identified several significant factors linked to the prevalence of STH, including the lack of a latrine at home, inadequate hand-washing practices following latrine use, the educational status of fathers, improper fingernail trimming practices, and certain school attendance patterns. The lack of a latrine at home was identified as a significant risk factor for STH infections in this study. This finding highlights the strong connection between poor sanitation and the transmission of helminths. It aligns with extensive research showing that limited access to sanitation facilities, especially at the household level, plays a major role in driving STH infections (50–52). Without access to latrines, many individuals are forced to practice open defecation, resulting in the direct release of helminth eggs into the environment and promoting continued transmission. Having access to household latrines significantly reduces the risk of exposure to infectious helminth stages in soil and water by minimizing the dispersion of feces in the surrounding area. Notably, This finding aligns with previous research in the same region, which also identified open field defecation as a key factor contributing to the prevalence of STH (22). This study reinforces existing evidence by examining sanitation status at the household level instead of relying on broader community-level indicators. By doing so, it provides a deeper understanding of how individual risks are influenced by domestic infrastructure. This highlights the need for targeted public health interventions that extend beyond general sanitation promotion. It is essential to prioritize equal access to latrines and ensure their sustained use at the household level, especially in high-transmission areas where environmental contamination continues to pose a significant challenge.

Hand hygiene practices, especially the failure to wash hands after using the latrine, were significantly associated with the prevalence of STH, which aligns with prior global studies (53–55). This highlights the importance of hand washing as a key factor in preventing the transmission of STH (56). In contrast to previous research in the same area, hand hygiene showed no significant effect (22). This divergence may be due to variations in study design, changes in hygiene behaviors over time, or previous underestimations of individual practices. Furthermore, recent improvements in sanitation infrastructure, in the absence of corresponding behavioral changes, could increase the relative impact of hand hygiene on the risk of infection. These findings underscore the complex interaction between environmental and behavioral factors that influence the transmission of STH and emphasize the importance of ongoing local assessments.

Moreover, the educational status of was found to correlate significantly with STH prevalence among children, which aligns with previous studies (57,58). This highlights the importance of targeting educational programs not only for children but also for parents to foster a supportive environment for health-promoting behaviors. Certain school attendance was also significantly associated with STH infection, in line with previous studies (59,60). This might be due to differences in sanitation facilities, hygiene practices, socio-economic status, and health education among students, which can significantly influence the transmission dynamics of STH infections in certain schools.

This study also revealed that the practice of not trimming fingernails was significantly associated with STH infection, which aligns with previous studies (51,61–63). Since long fingernails can harbor soil and pathogens, serving as a reservoir for helminth eggs that can be ingested or

transmitted through hand-to-mouth contact, public health initiatives need to promote personal hygiene practices among school-aged children, including regular nail-trimming and cleanliness.

The current study found a better diagnostic performance of the POC-CCA assay using a single urine sample than duplicate KK thick smears from a single stool sample. Thus, among the 96 KK positive samples, the POC-CCA test identified 10 (10.4%) as negative. Conversely, with KK negative results, POC-CCA classified only 78 (4.9%) as positive, indicating that the KK technique has high specificity.

In this study, using the KK technique as the gold standard, the POC-CCA test demonstrated higher sensitivity but notably lower specificity. The improved sensitivity (particularly in detecting low-intensity infections) might be attributed to the POC-CCA test's ability to detect trace levels of circulating antigens in urine, allowing for broader detection of Schistosoma infections, including cases that KK might miss due to the known variability in egg excretion and the limited sensitivity of KK, especially in low-endemic settings (64). However, the specificity of the POC-CCA test was found to be 79.6%, which is consistent with previously reported ranges but remains relatively low (20). This reduced specificity may be attributed to cross-reactivity with other helminth infections, the subjective interpretation of trace results as positive, and the continued excretion of CCA following recent treatment or in the absence of active infection (65). Such limitations can result in false positives, particularly in low-endemic or post-treatment settings, potentially leading to an overestimation of prevalence. Despite its limitations, the POC-CCA test is a valuable diagnostic tool in field settings because of its ease of use, rapid results, and high sensitivity. However, its limited specificity highlights the necessity for confirmatory testing, especially in situations involving surveillance, program evaluation, and low-transmission areas where diagnostic accuracy is crucial.

The overall agreement between KK and POC-CCA was moderate, as reflected by a kappa statistic of 0.55 ($p < 0.001$) and an observed agreement rate of 81.5%. While this level of concordance suggests a reasonable overlap between the two diagnostic tools, it must be interpreted cautiously given the limitations of κ statistics, which are sensitive to disease prevalence and imbalanced marginal totals (66). McNemar's test revealed a statistically significant discordance between the two diagnostic methods, with a chi-square value of 52.3 and $p < 0.001$. This discrepancy was primarily driven by the high number of cases that were positive by the point-of-care circulating cathodic antigen (POC-CCA) test but negative by the Kato-Katz (KK) method. This lack of alignment underscores the fundamentally different targets of the two diagnostic tools: the POC-CCA test detects active infections by identifying CCA in urine, while the KK method relies on detecting *Schistosoma* eggs in stool, which can vary from day to day and show inconsistencies within the same sample (17). Therefore, in field settings, especially in low-endemic or post-treatment areas, the POC-CCA test may identify infections that the KK method misses, making it a more sensitive tool for surveillance and monitoring of control programs.

Conclusions

There was a moderate prevalence of *S. mansoni* and STH, along with a moderate intensity of schistosomiasis and poor hygiene practices. This situation underscores for additional control measures, including health education. Furthermore, there is potential to incorporate POC-CCA testing alongside the KK method to enhance diagnostic accuracy in areas with low *S. mansoni* infection rates to monitor and improve mass deworming program disease management outcomes.

Limitations

This study has a few limitations that should be acknowledged. (i), the cross-sectional design provides a snapshot of *S. mansoni* and STH infection at a single point in time, which may not capture seasonal variations or fluctuations in infection rates over longer periods. (i), the use of a single stool sample per participant for the KK technique may have led to underestimation of infection prevalence and intensity, given the known day-to-day variability in helminth egg excretion. (iii), the purposive selection of schools limits the generalizability of the findings to the larger community in the study area.

Acknowledgement

Primarily, we would like to thank Hawassa University, School of Medical Laboratory Sciences, for funding this research. Then our thanks go to the study participants and the school community for their cooperation, as their contributions were crucial to its success. Finally, we would also like to thank Tulla Primary Hospital and Bushulo Mother and Child Health Specialty Center staff, especially laboratory professionals, for their dedication and hard work in collecting and conducting experiments.

References

1. Schistosomiasis and soil- transmitted helminthiases: progress report 2021. Weekly epidemiological record Relevé épidémiologique hebdomadaire. Wkly Epidemiol Rec. 2022;97(48):621–32.
2. Natalie G, Kibira SPS, Ssenyonga R, Id JN, Id AKS, Ssempebwa JC, et al. The prevalence of schistosomiasis in Uganda : A nationally representative population estimate to inform

control programs and water and sanitation interventions. PLoS Negl Trop Dis. 2019;13(8):1–21.

3. Hussen S, Assegu D, Tadesse BT, Shimelis T. Prevalence of Schistosoma mansoni infection in Ethiopia : a systematic review and meta-analysis. Trop Dis Travel Med Vaccines. 2021;7(4):1–12.

4. WHO. WHO Guidelines on control and elimination of human schistosomiasis [Internet]. Vol. 3, PLoS Neglected Tropical Diseases. 2022. 1–144 p.

5. Ahmed H, Ahmed H, Hong S tae, Tag A, Bashir E, Mohamed R, et al. Prevalence , risk factors , and clinical manifestations of schistosomiasis among school children in the White Nile River basin , Sudan. Parasit Vectors. 2014;7(1):1–11.

6. Nathan, Bezerra FSM, Colley DG, Fleming FM, Homeida M, Kabatereine N, et al. Review of 2022 WHO guidelines on the control and elimination of schistosomiasis. Lancet Infect Dis. 2022;22(11):e327–35.

7. Dahal A, Francis E, Francis J, Wamtas F. Soil-transmitted helminths and associated risk factors among elementary school pupils in Dadin Kowa, Jos. Niger Med J. 2019;60(4):181.

8. Getaneh M, Hailegebriel T, Munshea A. Prevalence and Associated Risk Factors of Soil-Transmitted Helminth Infections among Schoolchildren around Lake Tana , Northwest Ethiopia. J Parasitol Res. 2022;2022:13.

9. Wale M, Gedefaw S. Prevalence of Intestinal Protozoa and Soil Transmitted Helminths Infections among School Children in Jaragedo Town, South Gondar Zone of Ethiopia. J Trop Med. 2022;2022:11.

10. Beyene B, Vaz Nery S, Lambiyo T, Shimelis T. Schistosoma mansoni and soil-transmitted

helminth infections among schoolchildren living along the shore of Lake Hawassa, southern Ethiopia. Parasites and Vectors [Internet]. 2024;17(1):1–14.

11. Pullan RL, Smith JL, Jasrasaria R, Brooker SJ. Global numbers of infection and disease burden of soil transmitted helminth infections in 2010. Parasites and Vectors [Internet]. 2014;7(1):1–19.

12. Östan I, Kilimcioğlu AA, Girginkardeşler N, Özyurt BC, Limoncu ME, Ok ÜZ. Health inequities: Lower socio-economic conditions and higher incidences of intestinal parasites. BMC Public Health. 2007;7:1–8.

13. De Silva NR, Brooker S, Hotez PJ, Montresor A, Engels D, Savioli L. Soil-transmitted helminth infections: Updating the global picture. Trends Parasitol. 2003;19(12):547–51.

14. Vercruysse J, Albonico M, Behnke J, Kotze A, McCarthy J, Prichard R, et al. Monitoring Anthelmintic Efficacy for Soil Transmitted Helminths (STH). World Health Organ. 2008;1–64.

15. Montresor A, Mupfasoni D, Mikhailov A, Mwinzi P, Lucianez A, Jamsheed M, et al. The global progress of soil-transmitted helminthiasis control in 2020 and World Health Organization's targets for 2030. PLoS Negl Trop Dis. 2020;14(8):1–17.

16. Sousa MS, Dam GJ Van, Cristhiany M, Pinheiro C, Dood CJ De, Peralta JM, et al. Performance of an Ultra-Sensitive Assay Targeting the Circulating Anodic Antigen (CAA) for Detection of Schistosoma mansoni Infection in a Low-Endemic Area in Brazil. Front Immunol. 2019;10:1–16.

17. Lindholz CG, Favero V, Verissimo CDM, Russo R, Candido F, Souza RP De, et al. Study of diagnostic accuracy of Helmintex , Kato-Katz , and POC-CCA methods for diagnosing intestinal schistosomiasis in Candeal , a low intensity transmission area in northeastern

Brazil. PLoS Negl Trop Dis. 2018;12(3):1–16.

18. Mueller A, Fuss A, Ziegler U, Kaatano GM, Mazigo HD. Intestinal schistosomiasis of Ijinga Island , north-western Tanzania : prevalence , intensity of infection , hepatosplenic morbidities and their associated factors. BMC Infect Dis. 2019;19(832):1–12.

19. Haggag AA, Rabiee A, Elaziz KMA, Campbell CH, Colley DG, Ramzy RMR. Thirty-Day Daily Comparisons of Kato – Katz and CCA Assays of 45 Egyptian Children in Areas with Very Low Prevalence of Schistosoma mansoni. Am Soc Trop Med Hyg. 2019;100(3):578–83.

20. Colley DG, Binder S, Campbell C, King CH, Tchuenté LAT, N'Goran EK, et al. A five-country evaluation of a point-of-care circulating cathodic antigen urine assay for the prevalence of Schistosoma mansoni. Am J Trop Med Hyg. 2013;88(3):426–32.

21. Straily A, Kavere EA, Wanja D, Wiegand RE, Montgomery SP, Mwaki A, et al. Evaluation of the Point-of-Care Circulating Cathodic Antigen Assay for Monitoring Mass Drug Administration in a Schistosoma mansoni Control Program in Western Kenya. Am Soc Trop Med Hyg. 2022;106(1):303–11.

22. Tadege B, Shimelis T. Infections with Schistosoma mansoni and geohelminths among school children dwelling along the shore of the Lake Hawassa, southern Ethiopia. PLoS One. 2017;12(7):1–12.

23. Bugssa G, Teklehaymanot T, Medhin G, Berhe N. Prevalence and intensity of Schistosoma mansoni infection, and contributing factors in Alamata district of Tigray Region, Northern Ethiopia. PLoS Negl Trop Dis [Internet]. 2024;2024-Novem(11):1–18.

24. Casacuberta-Partal M, Hoekstra PT, Kornelis D, van Lieshout L, van Dam GJ. An innovative and user-friendly scoring system for standardised quantitative interpretation of

the urine-based point-of-care strip test (POC-CCA) for the diagnosis of intestinal schistosomiasis: a proof-of-concept study. Acta Trop [Internet]. 2019;2(17):1–4.

25. Okoyo C, Simiyu E, Njenga SM, Mwandawiro C. Comparing the performance of circulating cathodic antigen and Kato-Katz techniques in evaluating Schistosoma mansoni infection in areas with low prevalence in selected counties of Kenya: A cross-sectional study. BMC Public Health. 2018;18(1):1–7.

26. Tiruneh A, Zemene E, Abdissa Mizana B, Girma H, Dereje E, Sharew B, et al. Schistosoma mansoni Infections and Morbidities Among School Children in Hotspot Areas of Jimma Town, Southwest Ethiopia: A Cross-Sectional Study. Environ Health Insights. 2023;17:1–8.

27. Aliyi H, Ahmed M, Gobena T, Alemu BM, Adem HA, Usso AA. Prevalence and factors associated with Schistosoma mansoni infection among primary school children in Kersa District, Eastern Ethiopia. PeerJ. 2024;12(6):1–20.

28. Ashenafi T, Tadesse D, Zewdneh T. Infection prevalence of intestinal helminths and associated risk factors among schoolchildren in selected kebeles of Enderta district, Tigray, Northern Ethiopia. J Parasitol Vector Biol. 2014;6(11):166–73.

29. Ghazy RM, Ellakany WI, Badr MM, Taktak NEM, Elhadad H, Abdo SM, et al. Determinants of Schistosoma mansoni transmission in hotspots at the late stage of elimination in Egypt. Infect Dis Poverty. 2022;11(1):1–14.

30. Bartlett AW, Figueiredo JCS, Goor RC Van, Monaghan P, Lancaster W, Mugizi R, et al. Burden and factors associated with schistosomiasis and soil - transmitted helminth infections among school-age children in Huambo, Uige, and Zaire provinces, Angola. Infect Dis Poverty [Internet]. 2022;11(73):1–15.

31. Wubet K, Damtie D. Prevalence of Schistosoma mansoni Infection and Associated Risk Factors among School Children in Jiga Town, Northwest-Ethiopia : A Cross-Sectional Study. J Parasitol Res. 2020;2020:1–7.

32. Tazebew B, Temesgen D, Alehegn M, Salew D. Prevalence of S mansoni Infection and Associated Risk Factors among School Children in Guangua District, Northwest Ethiopia. J Parasitol Res. 2022;2022:1–9.

33. Ansha MG, Kuti KA, Girma E. Prevalence of Intestinal Schistosomiasis and Associated Factors among School Children in Wondo District, Ethiopia. J Trop Med. 2020;10:1–8.

34. Hailegebriel, Nibret, Munshea A et al. Prevalence, intensity, and associated risk factors of Schistosoma mansoni infections among schoolchildren around Lake Tana, northwestern Ethiopia. PLoS Negl Trop Dis [Internet]. 2021;15(10):1–16.

35. Azalech Tefera, Belay B. Epidemiology of Schistosoma mansoni infection and associated risk factors among school children attending primary schools near rivers in Jimma town, an urban setting, Southwest Ethiopia. PLoS One [Internet]. 2020;15(2):1–15.

36. Zeleke AJ, Addisu A, Tegegne Y. Prevalence, Intensity, and Associated Factors of Schistosoma mansoni among School Children in Northwest Ethiopia. J Parasitol Res. 2020;2020:1–7.

37. Assefa A, Erko B, Gundersen SG, Medhin G, Berhe N. Current status of Schistosoma mansoni infection among previously treated rural communities in the Abbey and Didessa Valleys , Western Ethiopia : Implications for sustainable control. PLoS One [Internet]. 2021;16(2):1–15.

38. Alemayehu B, Tomass Z. Schistosoma mansoni infection prevalence and associated risk factors among schoolchildren in Demba Girara, Damot Woide District of Wolaita Zone,

Southern Ethiopia. Asian Pac J Trop Med [Internet]. 2015;8(6):457–63.

39. Masaku J, Madigu N, Okoyo C, Njenga SM. Current status of Schistosoma mansoni and the factors associated with infection two years following mass drug administration programme among primary school children in Mwea irrigation scheme : A cross-sectional study. BMC Public Health. 2015;15(739):1–9.

40. Nigo MM, Odermatt P, Salieb-Beugelaar GB, Morozov O, Battegay M, Hunziker PR. Epidemiology of Schistosoma mansoni infection in Ituri province, North-Eastern democratic republic of the Congo. PLoS Negl Trop Dis [Internet]. 2021;15(12):1–26.

41. Hussein R, Id M, Minzi OS, Kinung SM, Kamuhabwa A, Aklillu E. Prevalence and correlates of intestinal schistosomiasis infection among school-aged children in North-Western Tanzania. PLoS One. 2020;15(2):1–17.

42. Maddren R, Phillips A, Ower A, Landeryou T, Mengistu B, Anjulo U, et al. Soil-transmitted helminths and schistosome infections in Ethiopia: a systematic review of progress in their control over the past 20 years [Internet]. Vol. 14, Parasites and Vectors. BioMed Central; 2021. p. 1–15.

43. Aemero M, Berhe N, Erko B. Status of Schistosoma mansoni prevalence and intensity of infection in geographically apart endemic localities of Ethiopia: a comparison. Ethiop J Health Sci. 2014;24(3):189–94.

44. Bajiro M, Dana D, Levecke B. Prevalence and intensity of Schistosoma mansoni infections among schoolchildren attending primary schools in an urban setting in southwest, ethiopia. BMC Res Notes [Internet]. 2017;10(1):1–6.

45. Montresor A, Mupfasoni D, Mikhailov A, Mwinzi P, Lucianez A, Jamsheed M, et al. 12879_2019_Article_4730.pdf. PLoS Negl Trop Dis. 2020;8(8):1–8.

46. Gass K, Addiss DG, Freeman MC. Exploring the Relationship between Access to Water, Sanitation and Hygiene and Soil-Transmitted Helminth Infection: A Demonstration of Two Recursive Partitioning Tools. PLoS Negl Trop Dis. 2014;8(6).

47. Jia TW, Melville S, Utzinger J, King CH, Zhou XN. Soil-transmitted helminth reinfection after drug treatment: A systematic review and meta-analysis. PLoS Negl Trop Dis. 2012;6(5).

48. Brooker SJ, Mwandawiro CS, Halliday KE, Njenga SM, McHaro C, Gichuki PM, et al. Interrupting transmission of soiltransmitted helminths: A study protocol for cluster randomised trials evaluating alternative treatment strategies and delivery systems in Kenya. BMJ Open. 2015;5(10):1–12.

49. Giardina F, Coffeng LE, Farrell SH, Vegvari C, Werkman M, Truscott JE, et al. Sampling strategies for monitoring and evaluation of morbidity targets for soil-transmitted helminths. PLoS Negl Trop Dis. 2019;13(6):1–17.

50. Fenta A, Mesganaw B, Belachew F, Teshale G, Abebaw G, Tesfa E, et al. Prevalence and risks of soil transmitted helminths among Ethiopian school children: A cross-sectional study. Asian Pac J Trop Med. 2022;15(3):121–7.

51. Samuel F, Demsew A, Alem Y, Hailesilassie Y. Soil transmitted Helminthiasis and associated risk factors among elementary school children in ambo town, western Ethiopia. BMC Public Health. 2017;17(1):1–7.

52. Tolera B, Mohammed J, Umer U, Abamecha J, Tebeje F, Sime A, et al. Prevalence, intensity, and associated factors of intestinal schistosomiasis among primary school children in Nono District, Southwest Ethiopia. Front Trop Dis. 2024;5(February):1–11.

53. Alemu G, Abossie A, Yohannes Z. Current status of intestinal parasitic infections and

associated factors among primary school children in Birbir town, Southern Ethiopia. BMC Infect Dis. 2019;19(1):4–11.

54. Zeynudin A, Degefa T, Suleman S, Abamecha A, Hajikelil Z, Wieser A. Prevalence and Determinants of Geohelminthiasis among School-Age Children in Jimma City, Ethiopia. J Trop Med. 2023;2023.

55. Bokicho B, Hailu D, Eshetu B, Matie M, Tadele T. Soil-transmitted helminthiases among school-age children and their association with water, sanitation, and hygiene, Hawassa City, Southern Ethiopia. PLoS Negl Trop Dis [Internet]. 2023;17(7):1–12.

56. Luby SP, Agboatwalla M, Painter J, Altaf A, Billhimer WL, Hoekstra RM. Effect of Intensive Handwashing Promotion on Childhood Diarrhea in High-Risk Communities in Pakistan Randomized Controlled Trial. JAMA. 2004 Jun;291(21):2547–54.

57. Feleke DG, Ali A, Bisetegn H, Debash H, Birara W, Andualem A. Soil-transmitted helminthes and Schistosoma mansoni infections among primary school children at Ambasame primary school, North-West Ethiopia: a cross-sectional study. BMC Pediatr. 2022;22(1):1–7.

58. Forson AO, Arthur I, Ayeh-Kumi PF. The role of family size, employment, and education of parents in the prevalence of intestinal parasitic infections in school children in Accra. PLoS One. 2018;13(2):1–10.

59. Kihoro RW, Mulewa D, Okoyo C, Ayaa D, Korir A, Njomo DW, et al. Epidemiology of soil-transmitted helminthiasis among school-aged children in pastoralist communities of Kenya: A cross-sectional study. PLoS One. 2024;19(5 MAY):1–20.

60. Debalke S, Worku A, Jahur N, Mekonnen Z. Soil transmitted helminths and associated factors among schoolchildren in government and private primary school in Jimma Town,

Southwest Ethiopia. Ethiop J Health Sci. 2013;23(3):237–44.

61. Genet A, Motbainor A, Samuel T, Azage M. Prevalence and associated factors of soil transmitted helminthiasis among school-age children in wetland and non-wetland areas of Blue Nile Basins, northwest Ethiopia: A community-based comparative study. SAGE Open Med. 2021;9.

62. Andargie Y, Alelign A, Tekeste Z. Prevalence and associated risk factors of soil-transmitted helminth infections among schoolchildren in Mekan Eyesus town, northwestern Ethiopia. Parasite Epidemiol Control. 2024;27(March):e00379.

63. Novianty S, Dimyati Y, Pasaribu S, Pasaribu AP. Risk Factors for Soil-Transmitted Helminthiasis in Preschool Children Living in Farmland, North Sumatera, Indonesia. J Trop Med. 2018;2018.

64. Graeff-Teixeira C, Favero V, Pascoal VF, de Souza RP, Rigo F de V, Agnese LHD, et al. Low specificity of point-of-care circulating cathodic antigen (POC[sbnd]CCA) diagnostic test in a non-endemic area for schistosomiasis mansoni in Brazil. Acta Trop. 2021;217:7.

65. Homsana A, Odermatt P, Southisavath P, Yajima A, Sayasone S. Cross-reaction of POC-CCA urine test for detection of Schistosoma mekongi in Lao PDR: A cross-sectional study. Infect Dis Poverty. 2020;9(1):1–9.

66. Casagrande A, Fabris F, Girometti R. Beyond kappa: an informational index for diagnostic agreement in dichotomous and multivalue ordered-categorical ratings. Med Biol Eng Comput. 2020;58(12):3089–99.

Abbreviation

AOR, adjusted odds ratio; CCA, circulating cathodic antigen; CI, Confidence interval; COR, Crude odds ratio; EPG, Eggs per gram; KK, Kato Katz; LR^+, Positive likelihood ratio; LR^-

, N likelihood ratio; MDA, Mass drug administration; NPV, Negative predictive value; NTD, neglected tropical disease; POC, Point of care; PPV, Positive predictive value; SOP, Standard operating procedure; STH, Soil-transmitted Helminth; WHO, World Health Organization

Availability of data and materials

All relevant data are within the manuscript, and the datasets generated and/or analyzed during the current study are submitted, subject to the approval of the Institutional Review Board of the Hawassa University College of Medicine and Health Sciences. Restrictions apply to the availability of these data, which parents had consented for the collected information to be used for our research study only, and so are not publicly available.

 Click here to access/download;Figure;Fig 1.tif

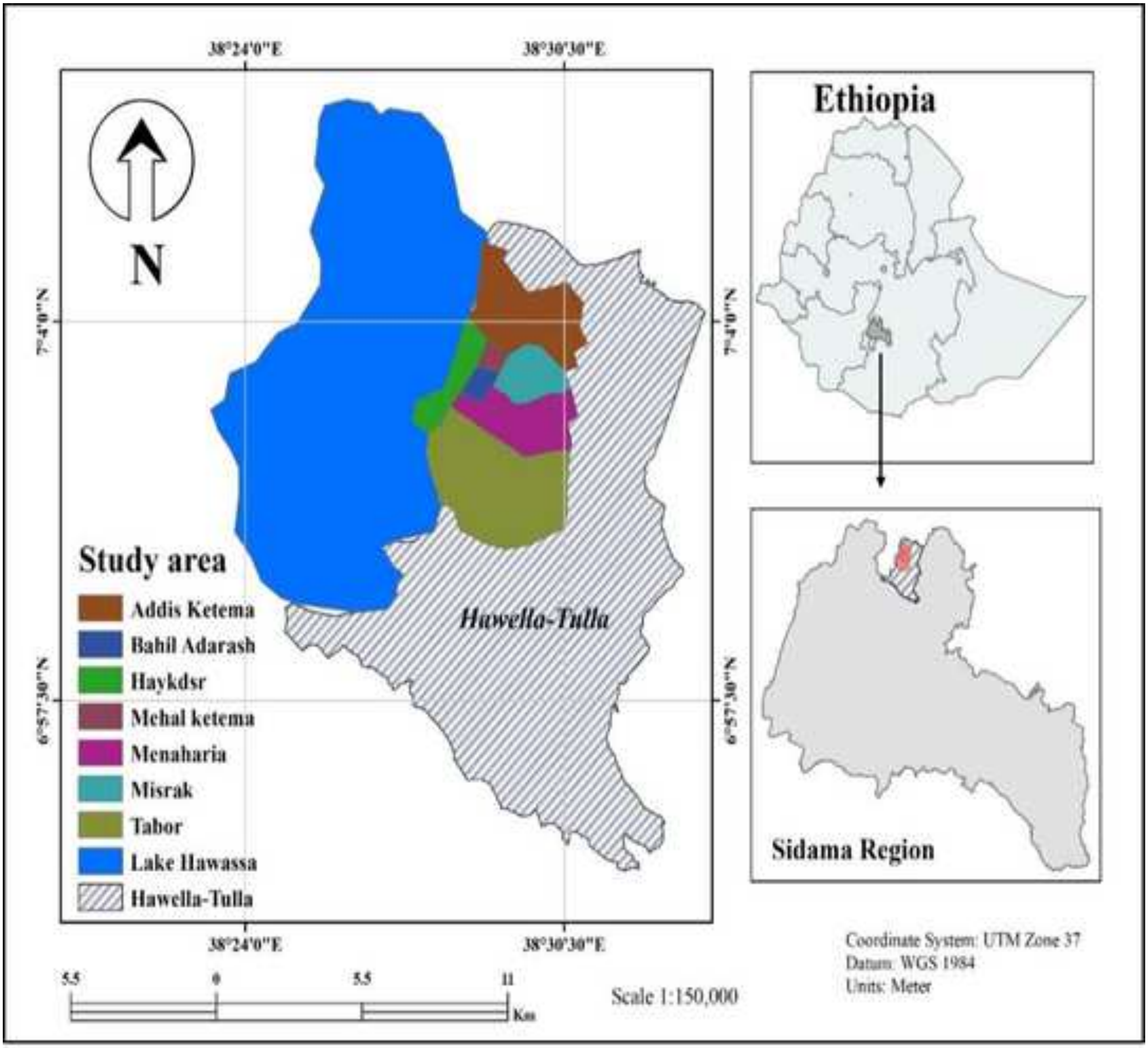

Fig 1: The figure in the left corner depicts Lake Hawassa and the study district, while the upper right corner shows Ethiopia and the bottom right shows the Sidama Region, 2024. The map is prepared using ArcGIS 10.8 (ESRI, Redlands, CA, USA) after obtaining country shape files from the humdata.org website (http://data.humdata.org/).

Click here to access/download;Figure;Fig 2..tif

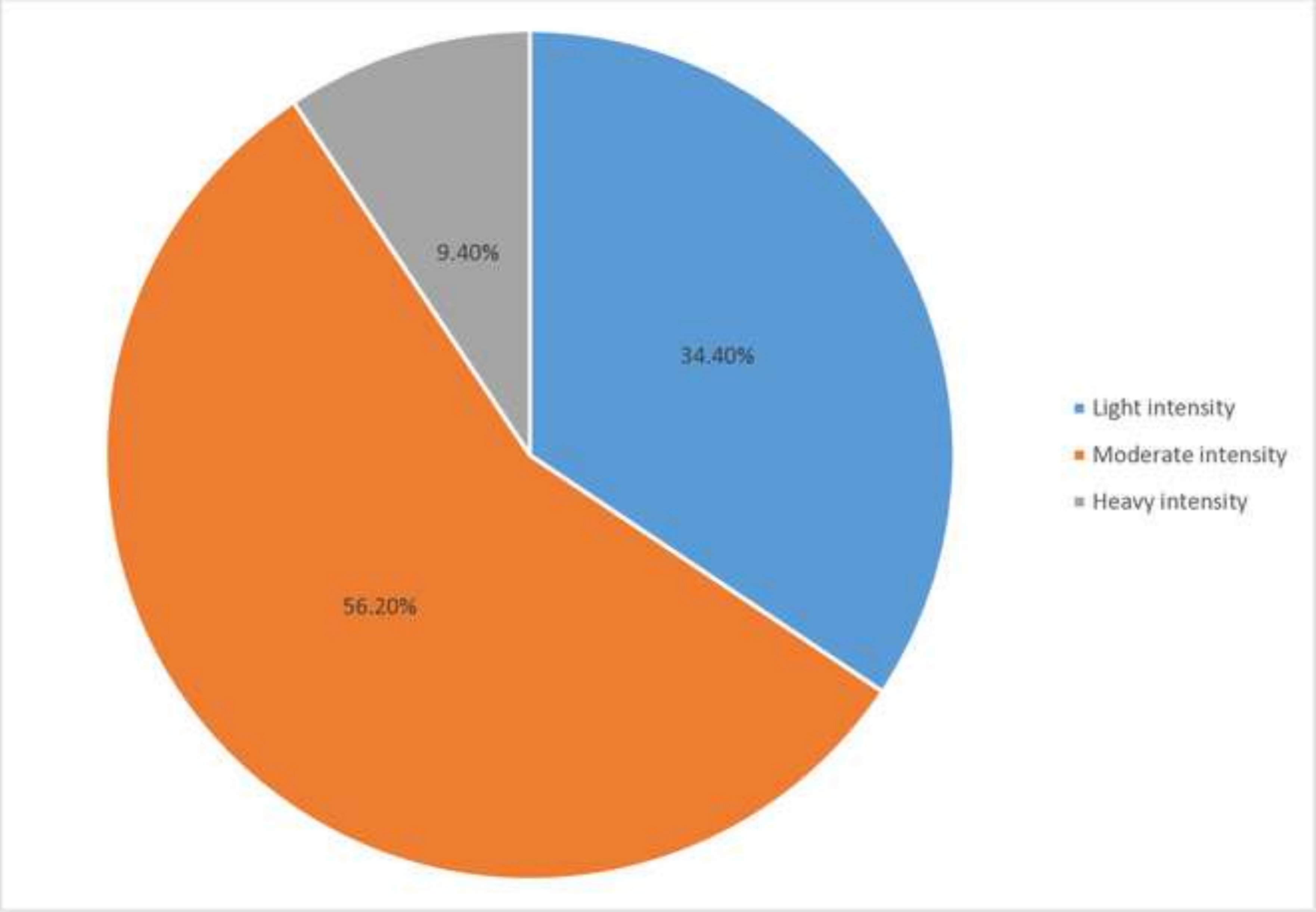

Fig 2: The prevalence of *S. mansoni* infection intensity in stool across 477 school children in Tulla district, Sidama Region, southern Ethiopia, 2024

Figure

Click here to access/download;Figure;Fig 3.tif

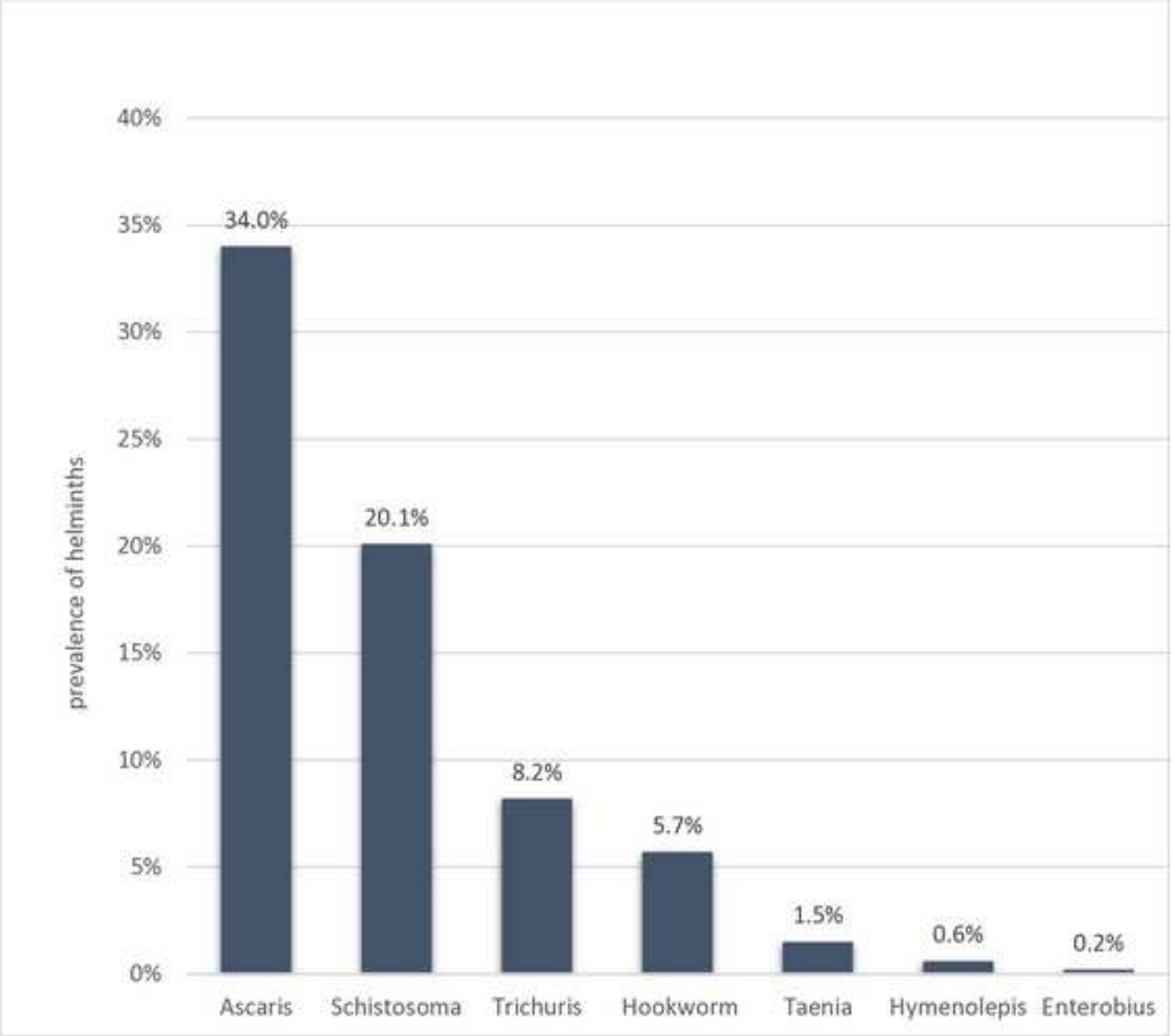

Figure 3: The prevalence of intestinal helminthic infection in stool across 477 school children in Tulla district, Sidama Region, southern Ethiopia, 2024

Click here to access/download
Supporting Information
S1 Table questionnaire.docx

Click here to access/download
Supporting Information
SPSS raw data.sav

Assessment of *Schistosoma mansoni* and Soil-transmitted Helminth Infections and the Diagnostic Performance of the Circulating Cathodic Antigen Test among Schoolchildren in Tulla District, Sidama Region, Southern Ethiopia.

Short title: *Schistosoma mansoni* Infection and Performance of its antigen test

Fitsum Getachew[1,2*], Demissie Assegu[1], Biniyam Kijineh[3] and Bamlaku Tadege[1*]

[1] School of Medical Laboratory Sciences, Hawassa University, Hawassa, Ethiopia.

[2] School of Medical Laboratory Sciences, Wolaita Sodo University, Wolaita, Ethiopia.

[3] School of Medical Laboratory Sciences, Wachemo University, Ethiopia, Hossana, Ethiopia.

*E-mail of corresponding author: bamlaku22tadege@gmail.com

Consent for publication

All authors have read the manuscript and consented to submit the manuscript.

Competing interests

All authors declare that they have no competing interests.

Authors' contributions

FG: conceptualization, data curation, formal analysis, investigation, methodology, writing – original draft; DA & BK: writing – review & editing, BT: conceptualization, supervision, writing – review & editing, data curation, and formal analysis.

Authors' addresses

fitsumgetachew6@gmail.com (FG)

bamlaku22tadege@gmail.com **(**BT**)**

demisissieasegu@yahoo.com (DA)

kijinehb@gmail.com (BK)

Funding

This study was funded by Hawassa University

Abstract

Background: Soil-transmitted helminths (STH) and *Schistosoma mansoni* pose significant public health challenges in areas with poor sanitation. While *S. mansoni* infection leads to chronic illness and mortality, research on its intensity remains limited in the study area. Diagnosis traditionally relies on the Kato-Katz method, though the POC-CCA urine test offers a rapid alternative. This study examined the prevalence, intensity, and risk factors of *S. mansoni* and Soil-transmitted helminth infections among schoolchildren while evaluating the diagnostic performance of the POC-CCA test.

Methods: A school-based cross-sectional study was conducted in the Tulla district, Sidama region, among 477 students (April–June 2024). The Schoolchildren were recruited using a purposive sampling technique. Socio-demographic data were collected using pre-structured questionnaires. A single stool sample was collected from each study participant and processed for the detection of helminth eggs using the Kato-Katz (KK) technique. Additionally, urine samples were tested with the Point of Care Circulating Cathodic Antigen Test (POC-CCA), which detects Schistosoma antigens. -Data was analyzed using SPSS and McNemar's chi-square test.

Commented [B1]: Data were processed in SPSS v26 using logistic regression, with p<0.05 considered statistically significant. Diagnostic accuracy was evaluated using 2×2 contingency tables and McNemar's chi-square test is repaced by Data was analyzed using SPSS and McNemar's chi-square test.

Result: The overall prevalence of *Schistosoma mansoni* was 36.5% (20.1% by KK and 34.4% by POC-CCA). The prevalence of STH was 48.8%. The leading parasite was *Ascaris lumbricoides (*34%*),* followed by *Trichuris trichuria* (8.2%) and hookworm (5.7%). Other helminths included Taenia species 1.3%, *Hymenolepis nana* 0.6%, and *Enterobius vermicularis* 0.2%. Infection intensity of *S. mansoni* was classified as light 34.4%, moderate 56.2%, and heavy 9.4%. Involvement in irrigation, swimming, bathing, and school location were

significantly associated with *S. mansoni* infection. School location, educational status, lack of latrine, hand washing practice and fingernail trimming practice were significantly associated with STH infections. The POC-CCA rapid test had a significantly higher sensitivity of 89.6% (McNemar test $\chi^2 m = 52.3$, $p < 0.001$) compared to the Kato-Katz technique.

Conclusions: Moderate *S. mansoni* and STH infection and poor hygiene practice underscore ~~the necessity~~ for additional control measures. Incorporating POC-CCA testing alongside the KK method could optimize diagnostic accuracy and improve disease management outcomes.

Keywords: Prevalence, Infection intensity, *Schistosoma mansoni,* Soil-transmitted helminth, Diagnostic performance, Kato-Katz technique, Point-of-care circulating cathodic antigen.

Introduction

Background

Schistosoma and soil-transmitted helminthiases (STH) are worm infections that belong to the group of neglected tropical diseases (NTDs) (1). Schistosomiasis is a water-based, vector-borne parasite transmitted indirectly through freshwater snails (2). People who live in areas with inadequate water and sanitation systems are susceptible to this most common and deadly infection (3). Globally, schistosomiasis is recognized as endemic in 78 low and middle-income countries in tropical and subtropical areas, estimated to affect >251 million people (4). Furthermore, 243 million people require preventative chemotherapy, including 111 million school-age children, of which 226 million are in Africa (5). It causes significant morbidity, with an estimated 1.4-3.3 million disability-adjusted life years (DALYs) lost annually due to its health impacts (6).

STHs are intestinal parasitic worms transmitted to humans through fecally contaminated soil (7). The main STHs that affect humans are *Ascaris lumbricoides* (A. *lumbricoides*), *Trichuris trichuria (*T. *trichuria), Necator americanus, and Ancylostoma duodenale* (8). These parasites cause gastrointestinal infections and are most commonly found in tropical and subtropical regions, particularly impacting impoverished nations where schoolchildren are especially vulnerable (9).

Commented [B2]: Parasite name changed to italic

Commented [B3R2]:

Globally, an estimated 1.5 billion people are infected with STHs, including 654 million school-aged children living in areas where these parasites are prevalent (10). The Burden of *Schistosoma* and STHs leads to various health issues, including gastrointestinal bleeding, nutrient malabsorption, loss of appetite, anemia due to the depletion of iron and other essential proteins, growth retardation, impaired cognitive development, school absenteeism, and disability-adjusted life-years lost (11–13).

Commented [B4]: Italic

Commented [B5R4]:

To control *Schistosoma* and STH-attributable morbidity, WHO recommends three interventions, including periodical administration of anthelmintic drugs to kill the worms, health education, and improved water, hygiene, and sanitation (WASH) to prevent worm re-infection. Today, mass drug administration (MDA) programs remain the mainstream strategy. During these programs, a

Commented [B6]: Italic

Commented [B7R6]:

single oral dose of albendazole (400 mg) or mebendazole (500 mg) for STH and praziquantel for S*chistosoma* are periodically administered to the at-risk groups (14). Although these deworming programs have been successful in reducing global *Schistosoma* and STH-attributable morbidity, re-infection perpetuates in the absence of improved WASH and health education (15).

Intestinal *Schistosoma* is diagnosed by microscopic detection of parasite eggs in stool using the Kato-Katz (KK) technique, which is widely used in epidemiological surveys (16). However, its diagnosis is challenging in low-endemic areas where prevalence and worm burden are reduced, due to factors like uneven egg distribution in stool samples, daily variations in egg excretion, and random distribution effects (17,18). On the other hand, the point-of-care (POC) cassette test is a relatively new diagnostic tool that detects circulating cathodic antigens (CCA) in urine samples, offering higher sensitivity and operational benefits (18,19). The study found that POC-CCA was more sensitive (86% vs. 62%) but less specific (72% vs. ~100%) compared to multiple KK smears from a single stool sample. Despite this, the study noted that only 1% of POC-CCA tests in non-endemic areas were false positives, indicating that the specificity of POC-CCA might be underestimated. Multivariable modeling also suggested that POC-CCA was significantly more sensitive than KK for detecting low infection intensities (<100 eggs/gram of stool) (20). However, before the POC-CCA assay can be fully utilized for monitoring and evaluating control programs, it is essential to assess its performance in endemic settings (21).

Therefore, this study aimed to evaluate the prevalence of *Schistosoma mansoni* (*S. mansoni*) and STH infections and intensity of *S. mansoni* in elementary school-aged children, as well as the associated risk factors to evaluate ongoing control intervention. Additionally, it sought to assess the performance of the POC-CCA test compared to the KK method for diagnosing *S. mansoni*.

Materials and Methods

Study design and setting

A school-based cross-sectional study was conducted between April and June 2024 among four governmental elementary school children in the Tulla district. The study was conducted in four elementary schools in the Tulla district, Sidama region in Southern Ethiopia. The district is situated along the shore of Lake Hawassa and the Tikur Wuha River, approximately 289

Commented [B8]: Italic
Commented [B9R8]:
Commented [B10]: Italic
Commented [B11R10]:
Commented [B12]: Italic
Commented [B13R12]:

kilometers south of Addis Ababa, the capital city of Ethiopia. The altitude is 1800 meters above sea level, with an annual rainfall of 1123 mm and temperatures ranging from 13 to 27°C. Lake Hawassa is the primary water source for the residents of Hawella Tulla, catering to their domestic, agricultural, fishing, and other needs. According to ECSA, the district has a total population of 129,507, comprising 65,018 males and 64,499 females. The district has various social service institutions, including 27 health facilities and 69 schools. The health facilities consist of 1 primary hospital, 6 health centers, 18 rural health posts, 2 urban health posts, and 1 non-governmental health center. In terms of education, the district includes 21 primary schools, 5 secondary schools, 6 non-governmental primary schools, and 37 non-governmental kindergarten schools (Fig 1).

Figure 1: Map of the study site. The figure in the left corner depicts Lake Hawassa and the study district, while the upper right corner shows Ethiopia. The figure at the bottom right displays the map of the Sidama region 2924 (The map is prepared using ArcGIS 10.8 (ESRI, Redlands, CA USA) after obtaining country shape files from the humdata.org website (http://data.humdata.org/).

Population and enrollment criteria

The source population was all schoolchildren in the Tulla district, Sidama region, southern Ethiopia. The study population included schoolchildren who were enrolled in the selected schools during the study period and met the established inclusion criteria (who had both parental or guardian informed consent and their assent to participate) and exclusion criteria (who had received anti-helminthic treatment within one month before the recruitment period, or who were unable to provide stool and urine samples were excluded from the study).

Sample size and sampling technique

To determine the sample size, a single population proportion formula was used based on the following assumptions: a prevalence of *S. mansoni* of 31% (22), with a 5% margin of error and a 95% confidence level. As a result, the calculated sample size was 329. Considering the design or cluster effect between schools, the determined number was multiplied by 1.5, and then 5% was added for the non-response rate, giving the final sample size of 484.

To recruit participants for this study, we utilized a purposive sampling technique. In the initial stage, Bushulo and Finchawa, located near the coasts of Lake Hawassa in the Tulla sub-city, were selected alongside two primary schools farther from the lake, Gemeto and Tulla, with student populations of 1,820, 686, 2,050, and 1,439, respectively. Then, the predetermined sample size of 484 was proportionally allocated to the schools, resulting in 147 participants from Bushulo, 55 from Finchawa, 116 from Gemeto, and 166 from Tulla. The students' registration lists served as the sampling frame, and a systematic random sampling technique was employed to select the study subjects from each school. The k-value (sampling interval) was determined by dividing the total number of study participants in each school by the number of students to be selected. The students were then systematically selected at regular intervals (every 12th student), stratified by age (5–19 years) and grade levels (1–4 and 5–8) until the predetermined sample size was achieved. In cases where the selected student was absent from school, three repeated visits were made. When a student missed after three visits, he/she considered non-respondents.

Dependent and independent variables

The dependent variables were prevalence, infection intensity, and diagnostic performance of *Schistosoma mansoni* infection. The independent variables include socio-demographic characteristics (gender, age, grade level, school attended, educational status of the father and mother, and family occupation). The independent variables consisted of socio-demographic characteristics such as gender, age, and educational status of both parents and family's occupation. Additionally, grade level and the schools attended were considered. The distance of the home from the lake and practice-related factors were also explored, focusing on habits such as swimming, fishing, shoe-wearing, washing clothes, hand washing before meals and after latrine, open-field defecation, trimming finger nail, bathing, and involvement in irrigation activities. Environmental factors considered included the availability of a latrine at home. Clinical symptoms of the disease were also assessed among study participants.

Commented [B14]: This one is deleted and replaced with The independent variables consisted of socio-demographic characteristics such as gender, age, and educational status of both parents and family's occupation. Additionally, grade level and the schools attended were considered.

Commented [B15R14]:

Data collection methods

Interview

The data was collected using a structured questionnaire. Two trained nurses conducted home-to-home visits to interview the parents or guardians of the children, utilizing the KOBO Collect digital tool. A senior expert and the principal investigator supervised the data collection process, and regular meetings were held to address any challenges and ensure adherence to established protocols and standards.

Laboratory investigation

Fecal and urine samples were collected in a screw-capped, dry, and clean container pre-labeled with the participant's unique identification number. The collected stool samples were transported within an hour of collection in suitable cool boxes at temperatures between 4°C and 6°C for subsequent examination. Samples from Gemeto and Tulla Elementary School were transported to Tulla Hospital, while samples from Bushulo and Finchawa Elementary School were transported to the Bushulo Mother and Child Health Specialty Center. Sample collection and transportation quality were supervised by the investigator to ensure adherence to procedures and the accuracy of the data results.

Stool sample processing using the Kato-Katz technique

The stool samples were examined using the KK technique to detect parasite eggs. A portion of the sample was processed using a calibrated template holding 41.7 mg of stool. Double slides were prepared for each stool sample. The number of eggs was counted from the double KK microscopic slides, and the average count was converted to eggs per gram of feces (EPG) using a correction factor of 24. Based on WHO cut-off values, the infection intensity was categorized as light (EPG 1-99), moderate (EPG 100-399), or heavy (EPG >400) (23).

Urine sample processing using POC-CCA rapid test

Each urine sample was processed immediately at the schools using the POC-CCA test, following the manufacturer's instructions (24). This on-site processing allowed for prompt results and minimized potential delays or degradation of the samples. 100µL of urine was transferred to the

circular well of the test cassette and allowed to absorb completely into the specimen pad. The test results were read after 20 minutes of assay development. In cases of discrepancies, the tests were re-examined to ensure accuracy. The POC-CCA tests were scored as negative or positive based on the formation and intensity of the control band. Tests were considered invalid if an internal control band did not appear or if results were not read after more than 25 minutes. In such cases, samples were re-run with a new cassette and scored as necessary.

Data quality assurance

The questionnaire was initially developed in English and then translated into Amharic and Sidamu Afoo, the local language of the participants. It was then translated back into English to ensure consistency. A pre-test was conducted on 5% of the sample size at Tullo Primary School, which was not part of the selected schools for the final study. The questionnaire was subsequently modified based on the findings from the pre-test. The completeness of data collection was overseen daily by the assigned supervisor.

The senior laboratory technologist provided refresher training to laboratory professionals. Standard operating procedures (SOPs) for pre-analytical, analytical, and post-analytical procedures were implemented throughout sample analysis. To guarantee quality control as an internal quality control measure, 10% of the slides were randomly selected and re-examined by a senior laboratory technologist at Tulla Hospital and Bushulo Mother and Child Health Specialty Centre and by another experienced technologist at HUCSH as part of an external laboratory evaluation. Both stool and urine samples were collected between 09:00-12:00 a.m. and 03:00-05:00 a.m. local time each day of the survey.

Data analysis

Data was entered into the KOBO Collect digital tool and analyzed using SPSS version 26 software. The prevalence of *S. mansoni* and STH infection was presented as a percentage, while infection intensity was classified as low, moderate, or heavy according to WHO guidelines (4).

The mean egg count was reported as a geometric mean. A logistic regression model was employed to identify potential determinants of *S. mansoni* and STH infections and assess the strength of associations. Crude odds ratios (COR) from bivariate analysis were calculated to identify significant variables associated with the outcome. To control for confounding effects, factors with a p-value ≤ 0.25 in bivariate analysis were included in multivariable logistic regression, allowing for the determination of adjusted odds ratios (AOR). A p-value < 0.05 was considered statistically significant. The diagnostic performance of the POC-CCA was assessed by calculating sensitivity, specificity, and other relevant metrics, with these tests regarded as reference standards (25). Kappa statistics were utilized to quantify agreement between the two diagnostic methods, and the concordance rate was calculated as the proportion of cases yielding identical results. McNemar's chi-square test was applied to analyze paired proportions of positive and negative results to investigate detection rate differences further.

Ethical consideration

Ethical clearance was obtained from Hawassa University, College of Medicine, and other Health Sciences Internal Review Board (IRB) with a reference number: IRB/172/16 E.C. A letter of support from Hawassa University School of Medical Laboratory was provided to the Sidama Regional State Health Bureau, as well as to the health and education offices in Tulla district and the directors of the selected schools. Participation was fully voluntary, with written informed consent obtained from the parents/guardians of the children enrolled. Assent was obtained from students aged ≥ 12 years, following parent/guardian consent. Schoolchildren who tested positive for any pathogenic intestinal parasites received appropriate treatment administered by qualified public health professionals. All data collected during the study were kept strictly confidential and used exclusively for research purposes. The study procedures were conducted in full compliance with relevant ethical guidelines and regulatory standards

Commented [B16]: This highlight is to indicate the Ethiopian calendar 2016 E.C is equivalent to 2024 G.C

Commented [B17R16]:

Commented [B18]: This is the revised one for consent and assent of study participants

Commented [B19R18]:

Result

Socio-demographic characteristics of the study participants

A total of 484 students were invited to participate in the study, of which 477 met the inclusion criteria and agreed to take part, yielding a response rate of 98.5%. The study sample included 208 (43.6%) males and 269 (56.4%) females. Participants' ages ranged from 5 to 19 years, with a mean age of 11.94 years and a standard deviation of 2.76 years. Most of the study participants (62.7%) were 10-14 years old. The majority of students reside within 1 km of a lake (66.5%) and are under the care of their private (daily laborer) parents or guardians (41.7%). The students were selected from 4 primary schools: Bushulo 143 (30%), Finchawa 53 (11.1%), Gemeto 116 (24.3%), and Tulla 165 (34.6%) (Table 1).

Table 1: The prevalence of *Schistosoma mansoni* infection by socio-demographic and other factors among schoolchildren in Tulla district, Sidama region, Southern Ethiopia 2024 (n = 477).

Variables	*S. mansoni*			COR (95% CI)	p-value	AOR (95%CI)	p-value
	Positive %	Negative %	Total %				
Gender							
Male	52(25.0)	156(75.0)	208(43.6)	1		1	
Female	44(16.4)	225(83.6)	269(56.4)	0.587(0.374-0.920)	0.020	0.607(0.359-1.027)	0.063
Age							
5-9	7(9.1)	70(90.9)	77(16.1)	1			
10-14	67(22.4)	232(77.6)	299(62.7)	2.888(1.268-6.578)	0.012	2.118(0.841-5.330)	0.111
15-19	22(21.8)	79(78.2)	101(21.2)	2.785(1.122-6.914)	0.027	1.442(0.495-4.198)	0.502
School							
Bushulo	52(36.4)	91(63.6)	143(30)	6.163(3.234-11.746)	0.001	4.685(1.869-11.742)	**0.001****
Finchawa	13(24.5)	40(75.5)	53(11.1)	3.505(1.526-8.051)	0.003	1.444(0.486-4.297)	0.509

Gemeto	17(14.6)	99(85.4)	116(24.3)	1.852(0.874-3.926)	0.108	1.528(0.662-3.525)	0.320
Tulla	14(8.5)	151(91.5)	165(34.6)	1			
Educational status of the father							
No formal education	17(18.5)	75(81.5)	92(19.3)	1		1	
Primary school	58(26.0)	165(74.0)	223(46.75)	1.551(0.846-2.841)	0.156	1.420(0.690-2.921)	0.341
Educational status of the mother							
No formal education	3(7.0)	40(93.0)	43(9)	1		1	
Primary school	62(23.2)	205(76.8)	267(56)	4.033(1.206-13.485)	0.024	3.391(0.889-12.937)	0.074
Secondary school	25(18.8)	108(81.2)	133(28)	3.086(0.883-10.787)	0.078	2.178(0.535-8.859)	0.277
College and above	6(17.7)	28(82.3)	34(7)	2.857(0.658-12.397)	0.161	2.384(0.414-13.719)	0.331
Distance of residential area							
Within 1km	49(30.6)	111(69.4)	160(33.5)	1		1	
More than 1km	47(14.8)	270(85.2)	317(66.5)	0.394(0.250-0.623)	0.001	1.194(0.615-2.318)	0.601
Bathing in the lake							
Yes	43(28.5)	108(71.5)	151(31.7)	2.051(1.295-3.248)	0.002	1.804(1.048-3.107)	**0.033***
No	53(16.3)	273(83.7)	326(68.3)	1		1	
Swimming habit							
Yes	50(31.9)	107(68,1	157(32.9)	2.783(1.780-4.403)	0.001	2.407(1.430-4.052)	**0.001****
No	46(14.4)	274(85.6)	320(67.1)	1			

Fishing							
Yes	20(35.1)	37(64.9)	57(12)	2.447(1.345-4.449)	0.003	1.135(0.533-2.417)	0.743
No	76(18.1)	344(71.9)	420(88)	1			
Washing clothes along the lake shore							
Yes	19(30.6)	43(69.4)	62(13)	1.940(1.071-3.513)	0.029	2.016(0.934-4.350)	0.074
No	77(17.3)	338(82.7)	415(87)	1			
Involvement in irrigation							
Yes	44(37.0)	75(63.0)	119(25)	3.452(2.148-5.547)	0.001	2.429(1.377-4.287)	**0.002***
No	52(14.5)	306(85.5)	358(75)	1			

AOR, Adjusted Odds Ratio; CI, Confidence interval; COR, Crude Odds Ratio; *, Significant at $p < 0.05$.

Prevalence of *Schistosoma mansoni* infection

The combined results of the 2 diagnostic methods showed an overall prevalence of *S. mansoni* to be 36.5%, with 20.1% (96/477) detected using the KK technique and 34.4% (164/477) detected using the POC-CCA test. The highest prevalence of *S. mansoni* infection, 30.5%, was observed among students attending schools near the lake (Bushulo and Finchawa). Overall, the prevalence of *S. mansoni* infection was highest among students aged 10 - 14 years (Table 1).

Infection intensity of *Schistosoma mansoni* infection

Out of 174 *S. mansoni* infected study participants, 96 tested positive by the KK technique which use using a standard amount of stool and that enables the quantification of ova per gram of stool. The minimum and maximum eggs per gram of stool were 24 and 672, respectively. Likewise, the arithmetic and geometric mean values were 189.75 and 139.39, respectively. According to WHO classification, among 96 students, 33 (34.4%) had light intensity, 54 (56.2%) had moderate intensity, and 9 (9.4%) had a heavy intensity of *S. mansoni* infection (Fig 2).

Commented [B20]: Changed to using

Commented [B21R20]:

Commented [B22]: Changed to that

Commented [B23R22]:

Fig 2: The prevalence of *S. mansoni* infection intensity across 477 schoolchildren in Tulla district Sidama Region, southern Ethiopia, 2024

Formatted: Font: Not Italic

As shown in Table 2, moderate infection with *S. mansoni* among males accounted for 53.9%, followed by 34.6% with light infection and 11.5% with heavy infection. Similarly, among the 15 – 19 years age group, most infections with were of moderate intensity (72.7%) followed by 18.2% with light and 9.1% with heavy intensity. When comparing schools, more than half of the infections with among respondents from Bushulo primary school were of moderate intensity at 61.5%, followed by 27% with light and 11.5% with heavy. However, most infections among respondents from Tulla primary school were of light intensity (64.3%) and moderate intensity (35.7%).

Table 2: Gender, age, and school-based variation in S. mansoni infection intensity among schoolchildren in Tulla District Sidama Region southern Ethiopia 2024 (n = 477).

Variable	N	Intensity of infection n (%)		
		Light	Moderate	Heavy
**Gender**				
Male	52	18(34.6)	28(53.9)	6(11.5)
Female	44	15(34.1)	26(59.1)	3(6.8)
**Age**				
5-9	7	2(28.6)	5(71.4)	0(0)
10-14	67	27(40.3)	33(49.25)	7(10.45)
15-19	22	4(18.2)	16(72.7)	2(9.1)
**Schoolchildren**				
Bushulo	52	14(27)	32(61.5)	6(11.5)
Finchawa	13	4(31)	7(53.8)	2(15.2)
Gemeto	17	6(35.3)	10(58.8)	1(5.9)
Tulla	14	9(64.3)	5(35.7)	0(0)

Key - N: refers to the total number of infected; whereas, n: refers to the number of infected according to intensity classes.

Potential associated risk factors of *S. mansoni* infections.

In the bivariate logistic regression analysis, activities such as washing clothes 13% (62/477), bathing 31.7% (151/477), swimming 32.9% (157/477), fishing 11.9% (57/477), and engaging in irrigation activities 24.9% (119/477), were identified as candidate potential risk factors of *S. mansoni* infection with a p-value ≤ 0.25. Socio-demographic factors such as gender, age, school (Bushulo), educational status of the father (primary school), educational status of the mother (primary school), and distance of residential area from the lake were also candidate potential predictors of *S. mansoni* infection (Table 1).

Commented [B24]: This one is deleted and replaced by potential risk

Commented [B25R24]:

Commented [B26]: This one is deleted and replaced by potential

Commented [B27R26]:

Multivariate logistic regression analysis was applied to avoid confounding factors and only students engaged in irrigation were 2.4 times more likely to be infected than those not involved (AOR = 2.43; 95% CI: 1.38-4.29, P = 0.002), schoolchildren with swimming habits exhibited a 2.4-fold increase in the likelihood of infection (AOR = 2.41; 95% CI: 1.43-4.34, P = 0.001) and participants who bathed in the lake were 1.8 times more likely to be infected than those who did not engage in this activity (AOR = 1.80; 95% CI: 1.05-3.12, P = 0.033). A school-wide analysis revealed that students attending Bushulo primary school were 4.7 times more likely to contract *S. mansoni* infection compared to their peers at Tulla primary school (AOR = 4.68; 95% CI: 1.87-11.74, P = 0.001) (Table 1).

Prevalence of STH infections

Aside from *S. mansoni*, the prevalence of intestinal helminths among the schoolchildren was 233 (48.8%). The most common STH was *Ascaris lumbricoides* 162 (34%), followed by *Trichuris trichuria* 39 (8.2%) and Hookworm 27 (5.7%). Other helminth parasites included Taenia species 6 (1.3%), *Hymenolepis nana* 3 (0.6%), and *Enterobius vermicularis* 1 (0.2%) (Fig 3).

Fig 3: The prevalence of intestinal helminthic infection across 477 schoolchildren in Tulla district Sidama Region, southern Ethiopia, 2024

Formatted: Font: Not Italic

Potential associated risk factors of STH infections.

In the bivariate logistic regression analysis, both hygiene-related practices and socio-demographic factors were identified as potential risk factors for STH infections. Household latrine availability (61%, 105/477), handwashing after latrine use (52.8%, 227/477), fingernail trimming (56.4%, 194/477), and handwashing before meals (53.4%, 196/477) were all associated with STH infections, with a p-value ≤ 0.25. Additionally, socio-demographic factors, including attending Tulla Elementary School, grade level, father's educational status, and family occupation (farmer), emerged as candidate predictors (Table 3).

Table 3: Univariate and multivariate logistic regression analyses for socio-demographic and other potential risk factors associated with Soil-transmitted helminths (STHs) infections among school children in Tulla district, Sidama region, Southern Ethiopia 2024 (n = 477).

Variables	STH			COR (95% CI)	p-value	AOR (95%CI)	p-value
	Positive %	Negative %	Total %				
Gender							
Male	97(46.6)	111(53.4)	208(43.6)	1			
Female	136(50.6)	133(49.4)	269(56.4)	1.170(0.814-1.681)	0.395		
Age							
5-9	38(49.4)	39(50.6)	77(16.1)	1			
10-14	151(50.5)	148(49.5)	299(62.7)	1.047(0.634-1.728)	0.857		

15-19	44(43.6)	57(56.4)	101(21.2)	0.792(0.437-1.437)	0.443		
School							
Bushulo	76(53.2)	67(46.8)	143(30)	1		1	
Finchawa	28(52.8)	25(47.2)	53(11.1)	0.987(0.525-1.857)	0.969		
Gemeto	54(46.5)	62(53.5)	116(24.3)	0.768(0.470-1.254)	0.292		
Tulla	75(45.5)	90(54.5)	165(34.6)	0.735(0.469-1.151)	0.178	0.469(0.256-0.861)	**0.015***
Grade level							
1-4	118(48.2)	127(51.8)	245(51.4)	1		1	
5-8	126(54.3)	106(45.7)	232(48.6)	1.279(0.893-1.834)	0.180	0.848(0.495-1.453)	0.548
Educational status of father							
No formal education	55(59.8)	37(40.2)	92(19.3)	3.964(1.420-11.066)	0.009	4.161(1.276-13.570)	**0.018***
Primary schoo	115(51.6)	108(48.4)	223(46.7)	2.840(1.072-7.523)	0.036	2.957(0.991-8.818)	0.052
Secondary education	57(40.7)	83(59.3)	140(29.4)	1.831(1.072-7.523)	0.234	1.616(0.547-4.775)	0.385
College and above	6(27.3)	16(72.7)	22(4.6)	1		1	
Occupation of family							
Civil servant	6(31.6)	13(68.4)	19(4)	1		1	
Merchant	56(41.5)	79(58.5)	135(28.3)	1.536(0.550-4.285)	0.412		
Farmer	63(50.8)	61(49.2)	124(26)	2.238(0.799-6.264)	0.125	0.199(0.013-3.028)	0.245
Private (Daily laborer)	108(54.3)	91(45.7)	199(41.7)	2.571(0.940-7.037)	0.066	0.262(0.017-3.932)	0.332

Latrine availability at home							
Yes	128(42)	177(58)	305(63.9)	1		1	
No	105(61)	67(39)	172(36.1)	0.461(0.315-0.676)	0.001	0.626(0.393-0.996)	**0.048***
Hand washing after latrine							
Yes	6(12.8)	41(87.2)	47(9.9)	1		1	
No	227(52.8)	203(47.2)	430(90.1)	0.131(0.54-0.315)	0.001	0.199(0.072-0.551)	**0.002***
Shoe wearing							
Yes	198(49.25	204(50.75)	402(84.3)	1			
No	35(46.7)	40(53.3)	75(15.7)	0.902(0.550-1.477)	0.681		
Finger nail trimming							
Yes	39(29.3)	94(70.7)	133(27.9)	1		1	
No	194(56.4)	150(43.6)	344(72.1)	0.321(0.209-0.493)	0.001	0.413(0.250-0.682)	**0.001***
Hand washing before meal							
Yes	37(33.6)	73(66.4)	110(23)	1		1	
No	196(53.4)	171(46.6)	367(77)	0.442(0.283-0.890)	0.001	0.585(0.337-1.015)	0.058
Open field defecation							
Yes	24(48)	26(52)	50(10.5)	1.039(0.578-1.867)	0.899		
No	209(48.9)	218(51.1)	427(89.5)	1			

Key - AOR: Adjusted Odds Ratio, CI: Confidence interval, COR: Crude Odds Ratio, *: Significant at $p < 0.05$, STH: Soil-transmitted helminths.

In the multivariate logistic regression analysis, several socio-demographic and behavioral factors were found to significantly influence the prevalence of STH infections among schoolchildren. Students from Tulla Elementary School had 0.47 times lower odds of STH infection compared to

their counterparts at Bushulo Elementary School (AOR = 0.469; 95% CI: 0.256-0.861, P = 0.015).). Additionally, the educational background of parents played a crucial role, as children whose fathers had no formal education were found to be 4.2 times more likely to be infected with STH compared to those whose fathers had attained a college education or higher (AOR = 4.161; 95% CI: 1.276-13.570, P = 0.018). Specifically, children whose fathers had no formal education were found to be 4.2 times more likely to be infected than those whose fathers had completed college or higher education (Adjusted Odds Ratio = 4.161; 95% Confidence Interval: 1.276-13.570, P = 0.018).

Commented [B28]: This one is deleted and replaced by Specifically, children whose fathers had no formal education were found to be 4.2 times more likely to be infected than those whose fathers had completed college or higher education (Adjusted Odds Ratio = 4.161; 95% Confidence Interval: 1.276-13.570, P = 0.018).

The presence of latrines at home also significantly influenced infection rates, with children having access to latrines exhibiting 0.63 times lower odds of infection than those without such facilities (AOR = 0.626; 95% CI: 0.393-0.996, P = 0.048). Furthermore, hygiene practices emerged as critical determinants, as children who washed their hands after using the latrine had a remarkable 0.2 times lower odds of contracting STH infections compared to those who did not (AOR = 0.199; 95% CI: 0.072-0.551, P = 0.002). Similarly, regular nail trimming was associated with a significant reduction in infection risk, with children who trimmed their nails having 0.4 times lower odds of STH infection than those who did not (AOR = 0.413; 95% CI: 0.250-0.682, P = 0.001) (Table 3).

Detection and performance evaluation of the POC-CCA test

Out of 477 samples examined, the POC-CCA rapid test identified 86 (18.0%) true positives, 303 (63.5%) true negatives, and 10 (2.1%) discrepancies (false negatives) compared to the KK technique. Using KK as the gold standard or ‘reference test’ (25), the POC-CCA sensitivity was 89.6% (95% CI: 83.5-95.7) and specificity 79.6% (95% CI: 75.6-83.6) (Table 4).

Table 4: Performance of POC-CCA test using Kato-Katz as a gold standard among schoolchildren in Tulla district Sidama Region, souther Ethiopia 2024 (n = 477).

Method	Result	KK (Gold standard) Pos	KK (Gold standard) Neg	Sensitivity % (95% CI)	Specificity % (95% CI)	NPV % (95% CI)	PPV % (95% CI)	LR^- % (95% CI)	LR^+ % (95% CI)
POC-CCA	Pos	86	78	89.6 (80-99.2)	79.6 (73.9-85.3)	96.8 (90.7-100)	52.4 (45.2-59.6)	0.131 (0.057-0.197)	4.39 (3.93-5.10)
	Neg	10	303						

Key - KK: Kato-Katz POC-CCA: Point of Care Circulating Cathodic Antigen, NPV: Negative Predictive value, PPV: Positive Predictive value, NLR^-: Negative Likely hood ratio, LR^+: Positive Likely hood ratio, Pos: Positive, Neg: Negative.

The agreement between the two diagnostic methods was quantified using Cohen's Kappa statistics which yielded a kappa value of 0.55. Furthermore, the overall concordance rate between the KK and POC-CCA was 81.5%. However, to assess the statistical significance of the differences in results between the two tests, we employed the McNemar Chi-square test. The result revealed a value of 52.3.

Discussion

This study aimed to assess the prevalence, intensity, and associated risk factors of *S. mansoni* and to evaluate the diagnostic performance of the POC-CCA rapid test among schoolchildren in the Tulla district of the Sidama region in southern Ethiopia. The prevalence of *S. mansoni* infection

was 20.1% (95% CI: 16.8-23.7) using the KK technique. This indicates the need for biannual deworming of schoolchildren in the study area with a moderate prevalence (≥ 10% but < 50%) according to WHO guidelines (4). This result aligns with previous studies from Jimma, 20.2% (26), Kersa, 19.9% (27), and Mekelle, 23.9% (28) in Ethiopia. Furthermore, the prevalence reported in this study is consistent with findings from Egypt, 19.1% (29), and Angola, 21.2% (30). However, it was higher than rates observed in Jiga at 15.2% (31), Guangua 12.6% (32), and Wondo districts 11.4% (33).

The prevalence in this study is lower than findings from Lake Tana in northwest Ethiopia, 34.9% (34), Jimma town, Ethiopia, 28.7% (35). The prevalence of this study is lower than rates reported in Maksegnit, Debark, Sanja, and Chuahit districts in northwest Ethiopia at 33.5% (36), Abbey and Didessa valleys 53.9% (37), and Wolaita southern Ethiopia 81.3% (38), Kenya, 53.7% (39), Congo, 90.2% (40), and Tanzania, 90.6% (41). The differences in the prevalence of *S. mansoni* infection in different regions may be attributed to variations in socio-demographic factors, exposure to water sources, rates of reinfection, diagnostic methods employed, and the expertise of the professionals conducting the examinations. The variations in the prevalence of S. mansoni infection across different regions may be attributed to several factors related to human behavior towards contaminated water sources, socioeconomic conditions and the rate of reinfection. These conditions include living environments, education levels, occupations, and the immune status of the individuals being studied. Furthermore, climate change may also influence human exposure to schistosomiasis. Environmental factors may also impact disease transmission variation across different regions include the proximity to snail habitats, the construction of dams, land cover (particularly elevation), rainfall patterns, seasonal land surface temperatures, and the presence of

Commented [B29]: Deleted and replaced by The variations in the prevalence of S. mansoni infection across different regions may be attributed to several factors related to human behavior towards contaminated water sources, socioeconomic conditions and the rate of reinfection. These conditions include living environments, education levels, occupations, and the immune status of the individuals being studied. Furthermore, climate change may also influence human exposure to schistosomiasis. Environmental factors may also impact disease transmission variation across different regions include the proximity to snail habitats, the construction of dams, land cover (particularly elevation), rainfall patterns, seasonal land surface temperatures, and the presence of flooded agricultural areas. Additionally, it is crucial to consider differences in sample sizes, study designs and diagnostic methods applied.

Commented [B30R29]:

flooded agricultural areas. Additionally, it is crucial to consider differences in sample sizes, study designs and diagnostic methods applied.

In the present study, out of 174 participants infected with *S. mansoni*, 96 were confirmed positive using the KK technique, a standard diagnostic method valued for its ability to quantify EPG of stool. The intensity of infection ranged from 24 to 672 EPG, with an arithmetic mean of 189.75 and a geometric mean of 139.39. The noticeable discrepancy between the arithmetic and geometric means reflects the presence of a right-skewed distribution of egg counts. This pattern is consistent with the typical epidemiology of schistosomiasis, where a minority of individuals harbor high worm burdens, while the majority exhibit low to moderate infection levels (42).

Commented [B31]: This is justification given for question asked whether the interpretation of infection intensity was affected by the skewed distribution when using arithmetic and geometric means

Commented [B32R31]:

The moderate infection intensity reported in this study is consistent with findings from various regions in Ethiopia (37,43) and in Tanzania (41). This difference might be due to repeated exposure of schoolchildren to water bodies infested with the infective stage. The intensity of *S. mansoni* infection was found to be slightly higher in male students compared to female students, which is consistent with findings from Kemissie and Wondo Genet (43) and Jimma Town (44) in Ethiopia. The observed difference in proportion might be linked to male students' greater exposure to outdoor activities like fishing, swimming, and irrigation compared to female students.

A significant association was found between *S. mansoni* infection and schoolchildren who engaged in swimming and bathing activities in the lake, with infection risks being 2.4 times higher for swimming and 1.8 times higher for bathing than those who did not partake in these habits. This association between *S. mansoni* infection and swimming habits among schoolchildren aligns with previous studies (22,32) and is further supported by studies linking bathing habits to infection (34).

Additionally, a strong association was noted between *S. mansoni* infection and participation in irrigation activities, with students involved in irrigation being 2.4 times more likely to be infected than those not engaged in such activities, consistent with prior findings (22,32). Notable differences in infection rates were observed among students across the various schools in the study areas. Children attending Bushulo Primary School were 4.7 times more likely to be infected with Schistosoma than their peers at Tulla Primary School. This finding aligns with previous studies (22,34). This might be associated with students' residence distance variation from lakes and other water bodies.

The study area displayed a moderate prevalence of STH infections among schoolchildren despite them being part of the deworming target group. Deworming programs have successfully decreased the global morbidity attributed to STHs (45), but the risk of re-infection persists in the absence of improved water, sanitation, and hygiene WASH practices and health education (46). A systematic review and meta-analysis estimated that three months after deworming, the prevalence of *Ascaris lumbricoides*, *Trichuris trichuria*, and *hookworms* reached 26%, 36%, and 30% of their pre-treatment levels, respectively. By one-year post-treatment, the prevalence rates approached their pre-treatment levels again, with *A. lumbricoides* at 94%, T. trichuria at 82%, and *hookworm* infections at 57% (47). The occurrence of re-infection highlights the importance of a comprehensive approach to controlling STH. This is especially crucial given the increasing interest in ultimately halting STH transmission in specific geographic regions (48). The current WHO guidelines recommend the evaluation of any STH infections in school-age children after five to six years of periodic chemotherapy using the standard Kato-Katz thick smear (49).

The findings of this study also provide critical insights into the socio-demographic and environmental determinants associated with STH infections among school children. This

research identified several significant factors linked to the prevalence of STH, including the lack of a latrine at home, inadequate hand-washing practices following latrine use, the educational status of fathers, improper fingernail trimming practices, and certain school attendance patterns. The lack of a latrine at home was identified as a significant risk factor for STH infections in this study. This finding highlights the strong connection between poor sanitation and the transmission of helminths. It aligns with extensive research showing that limited access to sanitation facilities, especially at the household level, plays a major role in driving STH infections (50–52). Without access to latrines, many individuals are forced to practice open defecation, resulting in the direct release of helminth eggs into the environment and promoting continued transmission. Having access to household latrines significantly reduces the risk of exposure to infectious helminth stages in soil and water by minimizing the dispersion of feces in the surrounding area. Notably, This finding aligns with previous research in the same region, which also identified open field defecation as a key factor contributing to the prevalence of STH (22). This study reinforces existing evidence by examining sanitation status at the household level instead of relying on broader community-level indicators. By doing so, it provides a deeper understanding of how individual risks are influenced by domestic infrastructure. This highlights the need for targeted public health interventions that extend beyond general sanitation promotion. It is essential to prioritize equal access to latrines and ensure their sustained use at the household level, especially in high-transmission areas where environmental contamination continues to pose a significant challenge.

Hand hygiene practices, especially the failure to wash hands after using the latrine, were significantly associated with the prevalence of STH, which aligns with prior global studies (53–55). This highlights the importance of hand washing as a key factor in preventing the

transmission of STH (56). In contrast to previous research in the same area, hand hygiene showed no significant effect (22). This divergence may be due to variations in study design, changes in hygiene behaviors over time, or previous underestimations of individual practices. Furthermore, recent improvements in sanitation infrastructure, in the absence of corresponding behavioral changes, could increase the relative impact of hand hygiene on the risk of infection. These findings underscore the complex interaction between environmental and behavioral factors that influence the transmission of STH and emphasize the importance of ongoing local assessments.

Commented [B33]: This is the discussion for questions raised to contextualize The association between STH infections and factors like latrine availability and hand washing   results align with or diverge from previous studies in similar settings.

Commented [B34R33]:

Moreover, the educational status of was found to correlate significantly with STH prevalence among children, which aligns with previous studies (57,58). This highlights the importance of targeting educational programs not only for children but also for parents to foster a supportive environment for health-promoting behaviors. Certain school attendance was also significantly associated with STH infection, in line with previous studies (59,60). This might be due to differences in sanitation facilities, hygiene practices, socio-economic status, and health education among students, which can significantly influence the transmission dynamics of STH infections in certain schools.

This study also revealed that the practice of not trimming fingernails was significantly associated with STH infection, which aligns with previous studies (51,61–63). Since long fingernails can harbor soil and pathogens, serving as a reservoir for helminth eggs that can be ingested or transmitted through hand-to-mouth contact, public health initiatives need to promote personal hygiene practices among school-aged children, including regular nail-trimming and cleanliness.

The current study found a better diagnostic performance of the POC-CCA assay using a single urine sample than duplicate KK thick smears from a single stool sample. Thus, among the 96 KK positive samples, the POC-CCA test identified 10 (10.4%) as negative. Conversely, with KK negative results, POC-CCA classified only 78 (4.9%) as positive, indicating that the KK technique has high specificity.

In this study, using the KK technique as the gold standard, the POC-CCA test demonstrated higher sensitivity but notably lower specificity. The improved sensitivity (particularly in detecting low-intensity infections) might be attributed to the POC-CCA test's ability to detect trace levels of circulating antigens in urine, allowing for broader detection of Schistosoma infections, including cases that KK might miss due to the known variability in egg excretion and the limited sensitivity of KK, especially in low-endemic settings (64). However, the specificity of the POC-CCA test was found to be 79.6%, which is consistent with previously reported ranges but remains relatively low (20). This reduced specificity may be attributed to cross-reactivity with other helminth infections, the subjective interpretation of trace results as positive, and the continued excretion of CCA following recent treatment or in the absence of active infection (65). Such limitations can result in false positives, particularly in low-endemic or post-treatment settings, potentially leading to an overestimation of prevalence. Despite its limitations, the POC-CCA test is a valuable diagnostic tool in field settings because of its ease of use, rapid results, and high sensitivity. However, its limited specificity highlights the necessity for confirmatory testing, especially in situations involving surveillance, program evaluation, and low-transmission areas where diagnostic accuracy is crucial.

The overall agreement between KK and POC-CCA was moderate, as reflected by a kappa statistic of 0.55 ($p < 0.001$) and an observed agreement rate of 81.5%. While this level of

concordance suggests a reasonable overlap between the two diagnostic tools, it must be interpreted cautiously given the limitations of κ statistics, which are sensitive to disease prevalence and imbalanced marginal totals (66). McNemar's test revealed a statistically significant discordance between the two diagnostic methods, with a chi-square value of 52.3 and $p < 0.001$. This discrepancy was primarily driven by the high number of cases that were positive by the point-of-care circulating cathodic antigen (POC-CCA) test but negative by the Kato-Katz (KK) method. This lack of alignment underscores the fundamentally different targets of the two diagnostic tools: the POC-CCA test detects active infections by identifying CCA in urine, while the KK method relies on detecting *Schistosoma* eggs in stool, which can vary from day to day and show inconsistencies within the same sample (17). Therefore, in field settings, especially in low-endemic or post-treatment areas, the POC-CCA test may identify infections that the KK method misses, making it a more sensitive tool for surveillance and monitoring of control programs.

Commented [B35]: Explanation for Diagnostic performance analysis

Commented [B36R35]:

Conclusions

There was a moderate prevalence of *S. mansoni* and STH, along with a moderate intensity of schistosomiasis and poor hygiene practices. This situation underscores ~~the necessity~~ for additional control measures, including health education. Furthermore, there is potential to incorporate POC-CCA testing alongside the KK method to enhance diagnostic accuracy in areas with low *S. mansoni* infection rates to monitor and improve mass deworming program disease management outcomes.

Limitations

This study has a few limitations that should be acknowledged. (i), the cross-sectional design provides a snapshot of *S. mansoni* and STH infection at a single point in time, which may not capture seasonal variations or fluctuations in infection rates over longer periods. (i), the use of a single stool sample per participant for the KK technique may have led to underestimation of infection prevalence and intensity, given the known day-to-day variability in helminth egg excretion. (iii), the purposive selection of schools limits the generalizability of the findings to the larger community in the study area.

Commented [B37]: Additional justification to the limitation of this study

Commented [B38R37]:

Acknowledgement

Primarily, we would like to thank Hawassa University, School of Medical Laboratory Sciences, for funding this research. Then our thanks go to the study participants and the school community for their cooperation, as their contributions were crucial to its success. Finally, we would also like to thank Tulla Primary Hospital and Bushulo Mother and Child Health Specialty Center staff, especially laboratory professionals, for their dedication and hard work in collecting and conducting experiments.

References

Commented [B39]: Reference number 1 and 4 are revised

Formatted: Highlight

1. Schistosomiasis and soil- transmitted helminthiases: progress report 2021. Weekly epidemiological record Relevé épidémiologique hebdomadaire. Wkly Epidemiol Rec. 2022;97(48):621–32.
2. Natalie G, Kibira SPS, Ssenyonga R, Id JN, Id AKS, Ssempebwa JC, et al. The prevalence of schistosomiasis in Uganda : A nationally representative population estimate to inform

control programs and water and sanitation interventions. PLoS Negl Trop Dis. 2019;13(8):1–21.

3. Hussen S, Assegu D, Tadesse BT, Shimelis T. Prevalence of Schistosoma mansoni infection in Ethiopia : a systematic review and meta-analysis. Trop Dis Travel Med Vaccines. 2021;7(4):1–12.

4. WHO. WHO Guidelines on control and elimination of human schistosomiasis [Internet]. Vol. 3, PLoS Neglected Tropical Diseases. 2022. 1–144 p.

5. Ahmed H, Ahmed H, Hong S tae, Tag A, Bashir E, Mohamed R, et al. Prevalence , risk factors , and clinical manifestations of schistosomiasis among school children in the White Nile River basin , Sudan. Parasit Vectors. 2014;7(1):1–11.

6. Nathan, Bezerra FSM, Colley DG, Fleming FM, Homeida M, Kabatereine N, et al. Review of 2022 WHO guidelines on the control and elimination of schistosomiasis. Lancet Infect Dis. 2022;22(11):e327–35.

7. Dahal A, Francis E, Francis J, Wamtas F. Soil-transmitted helminths and associated risk factors among elementary school pupils in Dadin Kowa, Jos. Niger Med J. 2019;60(4):181.

8. Getaneh M, Hailegebriel T, Munshea A. Prevalence and Associated Risk Factors of Soil-Transmitted Helminth Infections among Schoolchildren around Lake Tana , Northwest Ethiopia. J Parasitol Res. 2022;2022:13.

9. Wale M, Gedefaw S. Prevalence of Intestinal Protozoa and Soil Transmitted Helminths Infections among School Children in Jaragedo Town, South Gondar Zone of Ethiopia. J Trop Med. 2022;2022:11.

10. Beyene B, Vaz Nery S, Lambiyo T, Shimelis T. Schistosoma mansoni and soil-transmitted

Formatted: Highlight

helminth infections among schoolchildren living along the shore of Lake Hawassa, southern Ethiopia. Parasites and Vectors [Internet]. 2024;17(1):1–14.

11. Pullan RL, Smith JL, Jasrasaria R, Brooker SJ. Global numbers of infection and disease burden of soil transmitted helminth infections in 2010. Parasites and Vectors [Internet]. 2014;7(1):1–19.

12. Östan I, Kilimcioğlu AA, Girginkardeşler N, Özyurt BC, Limoncu ME, Ok ÜZ. Health inequities: Lower socio-economic conditions and higher incidences of intestinal parasites. BMC Public Health. 2007;7:1–8.

13. De Silva NR, Brooker S, Hotez PJ, Montresor A, Engels D, Savioli L. Soil-transmitted helminth infections: Updating the global picture. Trends Parasitol. 2003;19(12):547–51.

14. Vercruysse J, Albonico M, Behnke J, Kotze A, McCarthy J, Prichard R, et al. Monitoring Anthelmintic Efficacy for Soil Transmitted Helminths (STH). World Health Organ. 2008;1–64.

15. Montresor A, Mupfasoni D, Mikhailov A, Mwinzi P, Lucianez A, Jamsheed M, et al. The global progress of soil-transmitted helminthiasis control in 2020 and World Health Organization's targets for 2030. PLoS Negl Trop Dis. 2020;14(8):1–17.

16. Sousa MS, Dam GJ Van, Cristhiany M, Pinheiro C, Dood CJ De, Peralta JM, et al. Performance of an Ultra-Sensitive Assay Targeting the Circulating Anodic Antigen (CAA) for Detection of Schistosoma mansoni Infection in a Low-Endemic Area in Brazil. Front Immunol. 2019;10:1–16.

17. Lindholz CG, Favero V, Verissimo CDM, Russo R, Candido F, Souza RP De, et al. Study of diagnostic accuracy of Helmintex , Kato-Katz , and POC-CCA methods for diagnosing intestinal schistosomiasis in Candeal , a low intensity transmission area in northeastern

Brazil. PLoS Negl Trop Dis. 2018;12(3):1–16.

18. Mueller A, Fuss A, Ziegler U, Kaatano GM, Mazigo HD. Intestinal schistosomiasis of Ijinga Island , north-western Tanzania : prevalence , intensity of infection , hepatosplenic morbidities and their associated factors. BMC Infect Dis. 2019;19(832):1–12.

19. Haggag AA, Rabiee A, Elaziz KMA, Campbell CH, Colley DG, Ramzy RMR. Thirty-Day Daily Comparisons of Kato – Katz and CCA Assays of 45 Egyptian Children in Areas with Very Low Prevalence of Schistosoma mansoni. Am Soc Trop Med Hyg. 2019;100(3):578–83.

20. Colley DG, Binder S, Campbell C, King CH, Tchuenté LAT, N'Goran EK, et al. A five-country evaluation of a point-of-care circulating cathodic antigen urine assay for the prevalence of Schistosoma mansoni. Am J Trop Med Hyg. 2013;88(3):426–32.

21. Straily A, Kavere EA, Wanja D, Wiegand RE, Montgomery SP, Mwaki A, et al. Evaluation of the Point-of-Care Circulating Cathodic Antigen Assay for Monitoring Mass Drug Administration in a Schistosoma mansoni Control Program in Western Kenya. Am Soc Trop Med Hyg. 2022;106(1):303–11.

22. Tadege B, Shimelis T. Infections with Schistosoma mansoni and geohelminths among school children dwelling along the shore of the Lake Hawassa, southern Ethiopia. PLoS One. 2017;12(7):1–12.

23. Bugssa G, Teklehaymanot T, Medhin G, Berhe N. Prevalence and intensity of Schistosoma mansoni infection, and contributing factors in Alamata district of Tigray Region, Northern Ethiopia. PLoS Negl Trop Dis [Internet]. 2024;2024-Novem(11):1–18.

24. Casacuberta-Partal M, Hoekstra PT, Kornelis D, van Lieshout L, van Dam GJ. An innovative and user-friendly scoring system for standardised quantitative interpretation of

the urine-based point-of-care strip test (POC-CCA) for the diagnosis of intestinal schistosomiasis: a proof-of-concept study. Acta Trop [Internet]. 2019;2(17):1–4.

25. Okoyo C, Simiyu E, Njenga SM, Mwandawiro C. Comparing the performance of circulating cathodic antigen and Kato-Katz techniques in evaluating Schistosoma mansoni infection in areas with low prevalence in selected counties of Kenya: A cross-sectional study. BMC Public Health. 2018;18(1):1–7.

26. Tiruneh A, Zemene E, Abdissa Mizana B, Girma H, Dereje E, Sharew B, et al. Schistosoma mansoni Infections and Morbidities Among School Children in Hotspot Areas of Jimma Town, Southwest Ethiopia: A Cross-Sectional Study. Environ Health Insights. 2023;17:1–8.

27. Aliyi H, Ahmed M, Gobena T, Alemu BM, Adem HA, Usso AA. Prevalence and factors associated with Schistosoma mansoni infection among primary school children in Kersa District, Eastern Ethiopia. PeerJ. 2024;12(6):1–20.

28. Ashenafi T, Tadesse D, Zewdneh T. Infection prevalence of intestinal helminths and associated risk factors among schoolchildren in selected kebeles of Enderta district, Tigray, Northern Ethiopia. J Parasitol Vector Biol. 2014;6(11):166–73.

29. Ghazy RM, Ellakany WI, Badr MM, Taktak NEM, Elhadad H, Abdo SM, et al. Determinants of Schistosoma mansoni transmission in hotspots at the late stage of elimination in Egypt. Infect Dis Poverty. 2022;11(1):1–14.

30. Bartlett AW, Figueiredo JCS, Goor RC Van, Monaghan P, Lancaster W, Mugizi R, et al. Burden and factors associated with schistosomiasis and soil - transmitted helminth infections among school-age children in Huambo, Uige, and Zaire provinces, Angola. Infect Dis Poverty [Internet]. 2022;11(73):1–15.

31. Wubet K, Damtie D. Prevalence of Schistosoma mansoni Infection and Associated Risk Factors among School Children in Jiga Town, Northwest-Ethiopia : A Cross-Sectional Study. J Parasitol Res. 2020;2020:1–7.

32. Tazebew B, Temesgen D, Alehegn M, Salew D. Prevalence of S mansoni Infection and Associated Risk Factors among School Children in Guangua District, Northwest Ethiopia. J Parasitol Res. 2022;2022:1–9.

33. Ansha MG, Kuti KA, Girma E. Prevalence of Intestinal Schistosomiasis and Associated Factors among School Children in Wondo District, Ethiopia. J Trop Med. 2020;10:1–8.

34. Hailegebriel, Nibret, Munshea A et al. Prevalence, intensity, and associated risk factors of Schistosoma mansoni infections among schoolchildren around Lake Tana, northwestern Ethiopia. PLoS Negl Trop Dis [Internet]. 2021;15(10):1–16.

35. Azalech Tefera, Belay B. Epidemiology of Schistosoma mansoni infection and associated risk factors among school children attending primary schools near rivers in Jimma town, an urban setting, Southwest Ethiopia. PLoS One [Internet]. 2020;15(2):1–15.

36. Zeleke AJ, Addisu A, Tegegne Y. Prevalence, Intensity, and Associated Factors of Schistosoma mansoni among School Children in Northwest Ethiopia. J Parasitol Res. 2020;2020:1–7.

37. Assefa A, Erko B, Gundersen SG, Medhin G, Berhe N. Current status of Schistosoma mansoni infection among previously treated rural communities in the Abbey and Didessa Valleys , Western Ethiopia : Implications for sustainable control. PLoS One [Internet]. 2021;16(2):1–15.

38. Alemayehu B, Tomass Z. Schistosoma mansoni infection prevalence and associated risk factors among schoolchildren in Demba Girara, Damot Woide District of Wolaita Zone,

Southern Ethiopia. Asian Pac J Trop Med [Internet]. 2015;8(6):457–63.

39. Masaku J, Madigu N, Okoyo C, Njenga SM. Current status of Schistosoma mansoni and the factors associated with infection two years following mass drug administration programme among primary school children in Mwea irrigation scheme : A cross-sectional study. BMC Public Health. 2015;15(739):1–9.

40. Nigo MM, Odermatt P, Salieb-Beugelaar GB, Morozov O, Battegay M, Hunziker PR. Epidemiology of Schistosoma mansoni infection in Ituri province, North-Eastern democratic republic of the Congo. PLoS Negl Trop Dis [Internet]. 2021;15(12):1–26.

41. Hussein R, Id M, Minzi OS, Kinung SM, Kamuhabwa A, Aklillu E. Prevalence and correlates of intestinal schistosomiasis infection among school-aged children in North-Western Tanzania. PLoS One. 2020;15(2):1–17.

42. Maddren R, Phillips A, Ower A, Landeryou T, Mengistu B, Anjulo U, et al. Soil-transmitted helminths and schistosome infections in Ethiopia: a systematic review of progress in their control over the past 20 years [Internet]. Vol. 14, Parasites and Vectors. BioMed Central; 2021. p. 1–15.

43. Aemero M, Berhe N, Erko B. Status of Schistosoma mansoni prevalence and intensity of infection in geographically apart endemic localities of Ethiopia: a comparison. Ethiop J Health Sci. 2014;24(3):189–94.

44. Bajiro M, Dana D, Levecke B. Prevalence and intensity of Schistosoma mansoni infections among schoolchildren attending primary schools in an urban setting in southwest, ethiopia. BMC Res Notes [Internet]. 2017;10(1):1–6.

45. Montresor A, Mupfasoni D, Mikhailov A, Mwinzi P, Lucianez A, Jamsheed M, et al. 12879_2019_Article_4730.pdf. PLoS Negl Trop Dis. 2020;8(8):1–8.

46. Gass K, Addiss DG, Freeman MC. Exploring the Relationship between Access to Water, Sanitation and Hygiene and Soil-Transmitted Helminth Infection: A Demonstration of Two Recursive Partitioning Tools. PLoS Negl Trop Dis. 2014;8(6).

47. Jia TW, Melville S, Utzinger J, King CH, Zhou XN. Soil-transmitted helminth reinfection after drug treatment: A systematic review and meta-analysis. PLoS Negl Trop Dis. 2012;6(5).

48. Brooker SJ, Mwandawiro CS, Halliday KE, Njenga SM, McHaro C, Gichuki PM, et al. Interrupting transmission of soiltransmitted helminths: A study protocol for cluster randomised trials evaluating alternative treatment strategies and delivery systems in Kenya. BMJ Open. 2015;5(10):1–12.

49. Giardina F, Coffeng LE, Farrell SH, Vegvari C, Werkman M, Truscott JE, et al. Sampling strategies for monitoring and evaluation of morbidity targets for soil-transmitted helminths. PLoS Negl Trop Dis. 2019;13(6):1–17.

50. Fenta A, Mesganaw B, Belachew F, Teshale G, Abebaw G, Tesfa E, et al. Prevalence and risks of soil transmitted helminths among Ethiopian school children: A cross-sectional study. Asian Pac J Trop Med. 2022;15(3):121–7.

51. Samuel F, Demsew A, Alem Y, Hailesilassie Y. Soil transmitted Helminthiasis and associated risk factors among elementary school children in ambo town, western Ethiopia. BMC Public Health. 2017;17(1):1–7.

52. Tolera B, Mohammed J, Umer U, Abamecha J, Tebeje F, Sime A, et al. Prevalence, intensity, and associated factors of intestinal schistosomiasis among primary school children in Nono District, Southwest Ethiopia. Front Trop Dis. 2024;5(February):1–11.

53. Alemu G, Abossie A, Yohannes Z. Current status of intestinal parasitic infections and

associated factors among primary school children in Birbir town, Southern Ethiopia. BMC Infect Dis. 2019;19(1):4–11.

54. Zeynudin A, Degefa T, Suleman S, Abamecha A, Hajikelil Z, Wieser A. Prevalence and Determinants of Geohelminthiasis among School-Age Children in Jimma City, Ethiopia. J Trop Med. 2023;2023.

55. Bokicho B, Hailu D, Eshetu B, Matie M, Tadele T. Soil-transmitted helminthiases among school-age children and their association with water, sanitation, and hygiene, Hawassa City, Southern Ethiopia. PLoS Negl Trop Dis [Internet]. 2023;17(7):1–12.

56. Luby SP, Agboatwalla M, Painter J, Altaf A, Billhimer WL, Hoekstra RM. Effect of Intensive Handwashing Promotion on Childhood Diarrhea in High-Risk Communities in Pakistan Randomized Controlled Trial. JAMA. 2004 Jun;291(21):2547–54.

57. Feleke DG, Ali A, Bisetegn H, Debash H, Birara W, Andualem A. Soil-transmitted helminthes and Schistosoma mansoni infections among primary school children at Ambasame primary school, North-West Ethiopia: a cross-sectional study. BMC Pediatr. 2022;22(1):1–7.

58. Forson AO, Arthur I, Ayeh-Kumi PF. The role of family size, employment, and education of parents in the prevalence of intestinal parasitic infections in school children in Accra. PLoS One. 2018;13(2):1–10.

59. Kihoro RW, Mulewa D, Okoyo C, Ayaa D, Korir A, Njomo DW, et al. Epidemiology of soil-transmitted helminthiasis among school-aged children in pastoralist communities of Kenya: A cross-sectional study. PLoS One. 2024;19(5 MAY):1–20.

60. Debalke S, Worku A, Jahur N, Mekonnen Z. Soil transmitted helminths and associated factors among schoolchildren in government and private primary school in Jimma Town,

Southwest Ethiopia. Ethiop J Health Sci. 2013;23(3):237–44.

61. Genet A, Motbainor A, Samuel T, Azage M. Prevalence and associated factors of soil transmitted helminthiasis among school-age children in wetland and non-wetland areas of Blue Nile Basins, northwest Ethiopia: A community-based comparative study. SAGE Open Med. 2021;9.

62. Andargie Y, Alelign A, Tekeste Z. Prevalence and associated risk factors of soil-transmitted helminth infections among schoolchildren in Mekan Eyesus town, northwestern Ethiopia. Parasite Epidemiol Control. 2024;27(March):e00379.

63. Novianty S, Dimyati Y, Pasaribu S, Pasaribu AP. Risk Factors for Soil-Transmitted Helminthiasis in Preschool Children Living in Farmland, North Sumatera, Indonesia. J Trop Med. 2018;2018.

64. Graeff-Teixeira C, Favero V, Pascoal VF, de Souza RP, Rigo F de V, Agnese LHD, et al. Low specificity of point-of-care circulating cathodic antigen (POC[sbnd]CCA) diagnostic test in a non-endemic area for schistosomiasis mansoni in Brazil. Acta Trop. 2021;217:7.

65. Homsana A, Odermatt P, Southisavath P, Yajima A, Sayasone S. Cross-reaction of POC-CCA urine test for detection of Schistosoma mekongi in Lao PDR: A cross-sectional study. Infect Dis Poverty. 2020;9(1):1–9.

66. Casagrande A, Fabris F, Girometti R. Beyond kappa: an informational index for diagnostic agreement in dichotomous and multivalue ordered-categorical ratings. Med Biol Eng Comput. 2020;58(12):3089–99.

Abbreviation

AOR, adjusted odds ratio; CCA, circulating cathodic antigen; CI, Confidence interval; COR, Crude odds ratio; EPG, Eggs per gram; KK, Kato Katz; LR^+, Positive likelihood ratio; LR^-

, N likelihood ratio; MDA, Mass drug administration; NPV, Negative predictive value; NTD, neglected tropical disease; POC, Point of care; PPV, Positive predictive value; SOP, Standard operating procedure; STH, Soil-transmitted Helminth; WHO, World Health Organization

Availability of data and materials

All relevant data are within the manuscript, and the datasets generated and/or analyzed during the current study are and submitted with revised manuscript. ~~available from the corresponding author upon reasonable request, subject to the approval of the Institutional Review Board of the Hawassa University College of Medicine and Health Sciences.~~ Restrictions apply to the availability of these data, which parents had consented for the collected information to be used for our research study only, and so are not publicly available.

Response to the PLoS ONE editor and reviewers

First and foremost, we sincerely thank you for reviewing our manuscript and offering valuable comments and suggestions. Your insights have been very helpful in highlighting areas for improvement, and we have carefully addressed each of the points you raised.

1. Please ensure that your manuscript meets PLOS ONE's style requirements, including those for file naming. The PLOS ONE style templates can be found at

https://journals.plos.org/plosone/s/file?id=wjVg/PLOSOne_formatting_sample_main_body.pdf and

https://journals.plos.org/plosone/s/file?id=ba62/PLOSOne_formatting_sample_title_authors_affiliations.pdf.

We followed our manuscript to meet PLOS ONE's style requirements, including those for file naming.

2. PLOS requires an ORCID iD for the corresponding author in Editorial Manager on papers submitted after December 6th, 2016. Please ensure that you have an ORCID iD and that it is validated in Editorial Manager. To do this, go to 'Update my Information' (in the upper left-hand corner of the main menu), and click on the Fetch/Validate link next to the ORCID field. This will take you to the ORCID site and allow you to create a new iD or authenticate a pre-existing iD in Editorial Manager.

The corresponding author's ORCID ID is addressed

3. We note that your Data Availability Statement is currently as follows: [data are all contained within the manuscript and/or Supporting Information files, enter the following: All relevant data are within the manuscript and its Supporting Information files.]

Please confirm at this time whether or not your submission contains all raw data required to replicate the results of your study. Authors must share the "minimal data set" for their submission. PLOS defines the minimal data set to consist of the data required to replicate all study findings reported in the article, as well as related metadata and methods

We included raw data used for this manuscript results analysis

Reviewers' comments:

Reviewer's Responses to Questions

Comments to the Author

1. Is the manuscript technically sound, and do the data support the conclusions?

The manuscript must describe a technically sound piece of scientific research with data that supports the conclusions. Experiments must have been conducted rigorously, with appropriate controls, replication, and sample sizes. The conclusions must be drawn appropriately based on the data presented.

Reviewer #1: Yes

Reviewer #2: Yes

We thank all reviewers

---

2. Has the statistical analysis been performed appropriately and rigorously?

Reviewer #1: Yes

Reviewer #2: Yes

We thank all reviewers

---

3. Have the authors made all data underlying the findings in their manuscript fully available?

The PLOS Data policy requires authors to make all data underlying the findings described in their manuscript fully available without restriction, with rare exception (please refer to the Data Availability Statement in the manuscript

PDF file). The data should be provided as part of the manuscript or its supporting information, or deposited to a public repository. For example, in addition to summary statistics, the data points behind means, medians and variance measures should be available. If there are restrictions on publicly sharing data—e.g. participant privacy or use of data from a third party—those must be specified.

Reviewer #1: Yes

Reviewer #2: No

Thank you for your curiosity. We attached raw data and supplementary information, like the questionnaire

---

4. Is the manuscript presented in an intelligible fashion and written in Standard English?

PLOS ONE does not copyedit accepted manuscripts, so the language in submitted articles must be clear, correct, and unambiguous. Any typographical or grammatical errors should be corrected at revision, so please note any specific errors here.

Reviewer #1: Yes

Reviewer #2: No

We revised our manuscript once again for any topographical and grammatical corrections in the revised manuscript and indicated on the marked revised manuscript

---

5. Review Comments to the Author

Please use the space provided to explain your answers to the questions above. You may also include additional comments for the author, including concerns about dual publication, research ethics, or publication ethics. (Please upload your review as an attachment if it exceeds 20,000 characters)

Reviewer #1: The manuscript is well written, the methodology, results, statistical analysis and discussion were well presented. The manuscript is technically sound, and data provided in the manuscript. The statistical analysis

is appropriate. All findings were outlined in the manuscript. The manuscript is presented in Standard English.

The authors should address the comments made on the manuscript.

Reviewer #2: General comments
The manuscript presents a well-conducted cross-sectional study on the prevalence, intensity, and risk factors of Schistosoma mansoni and soil-transmitted helminth (STH) infections among schoolchildren in Ethiopia, alongside an evaluation of the diagnostic performance of the Point-of-Care Circulating Cathodic Antigen (POC-CCA) test. The study addresses an important public health issue in a region where these infections are endemic. The methodology is robust, and the findings are relevant to ongoing control efforts. However, there are several areas where the manuscript could be improved to enhance clarity, accuracy, and impact.
1. Ethical considerations: The manuscript states that ethical clearance was obtained from Hawassa University, but the reference number provided (IRB/172/16) appears outdated (2016). Given that the study was conducted in 2024, clarification or updated ethical approval documentation is necessary. Again, the consent process is described, but it would be beneficial to explicitly state whether assent was obtained from all children, including those under 12, or only those aged 12 and above.

We want to clarify that the ethical clearance reference number is not outdated; it is based on the Ethiopian calendar (E.C.). The reference number: IRB/172/16 E.C. corresponds to the Gregorian calendar date of 22/03/2024, which is indicated on the ethical clearance letter. We add E.C. to indicate the calendar, see the correction on line number 247 in the revised manuscript. Additionally, we acknowledge that our initial manuscript did not mention that assent was obtained from students aged ≥12 years following parent/guardian consent. This has now been addressed and appropriately revised in the manuscript. See the revised one from line number 250 – 252

2. Data availability: The manuscript claims that "all relevant data are within the manuscript and its Supporting Information files," but no supplementary files are provided. The authors should either upload the supporting data or clarify where it can be accessed.

Regarding the comment on the supplementary files, we have separately supplementary information. We also attached raw data

3. Diagnostic performance analysis: The comparison between POC-CCA and Kato-Katz is a key contribution, but the specificity of POC-CCA (79.6%) is notably low. The authors should discuss potential reasons for this (e.g., cross-reactivity, persistent antigens post-treatment) and implications for field use. Again, the McNemar's test result ($\chi^2 = 52.3$, $p < 0.001$) indicates significant disagreement between the two tests, but the discussion does not adequately address this. The authors should elaborate on the practical implications of this discrepancy.

Regarding the specificity of the POC-CCA test (79.6%), which is notably low, we were asked to discuss potential reasons and its implications for field use. We have addressed this from line numbers 516 – 540 in the revised manuscript as follows: **"However, the specificity of the POC-CCA test was found to be 79.6%, which is consistent with previously reported ranges but remains relatively low (20). This reduced specificity may be attributed to cross-reactivity with other helminth infections, the subjective interpretation of trace results as positive, and the continued excretion of CCA following recent treatment or in the absence of active infection (64). Such limitations can result in false positives, particularly in low-endemic or post-treatment settings, potentially leading to an overestimation of prevalence. Despite this, the POC-CCA test remains a practical diagnostic tool in field settings due to its operational simplicity, rapid results, and high sensitivity. However, its limited specificity underscores the importance of confirmatory testing, especially in surveillance, program evaluation, and low-transmission areas where diagnostic accuracy is critical."** Regarding the McNemar's test result ($\chi^2 = 52.3$, $p < 0.001$), we were informed that the discussion did not adequately address the observed discrepancy or elaborate on its practical implications. Therefore, we have addressed the issue as follows: **"Importantly, McNemar's**

test revealed a statistically significant discordance between the two methods ($\chi^2 = 52.3$, $p < 0.001$), driven primarily by the high number of POC-CCA positive but KK negative cases. This lack of alignment between the two diagnostic tools underscores their fundamentally different targets: POC-CCA detects active infection through CCA in urine, whereas KK relies on the detection of Schistosoma eggs in stool, which are subject to day-to-day variation and intra-sample inconsistencies (17). Therefore, in field settings, particularly in low-endemic or post-treatment areas, the POC-CCA test may identify infections that KK misses, making it a more sensitive tool for surveillance and monitoring of control programs."

4. Risk factor analysis: The association between STH infections and factors like latrine availability and handwashing is well-documented in the literature. The authors should contextualize their findings more critically, discussing how their results align with or diverge from previous studies in similar settings. Again, the multivariate analysis for S. mansoni risk factors is clear, but the odds ratios for some variables (e.g., swimming, irrigation) are high. The authors should consider potential confounding factors (e.g., frequency of water contact) that were not adjusted for.

As we were asked to critically analyse the association between STH infections and factors like latrine availability and hand washing, and to discuss how our results compare or differ from previous studies in similar settings, we addressed this in lines 464 –491 of the revised manuscript as follows:

- **The lack of a latrine at home was identified as a significant risk factor for soil-transmitted helminth (STH) infections in this study. This finding highlights the strong connection between poor sanitation and the transmission of helminths. It aligns with extensive research showing that limited access to sanitation facilities, especially at the household level,**

plays a major role in driving STH infections (49–51). Without access to latrines, many individuals are forced to practice open defecation, resulting in the direct release of helminth eggs into the environment and promoting continued transmission. Having access to household latrines significantly reduces the risk of exposure to infectious helminth stages in soil and water by minimizing the dispersion of feces in the surrounding area. Notably, This finding aligns with previous research in the same region, which also identified open field defecation as a key factor contributing to the prevalence of soil-transmitted helminths (STH) (22). This study reinforces existing evidence by examining sanitation status at the household level instead of relying on broader community-level indicators. By doing so, it provides a deeper understanding of how individual risks are influenced by domestic infrastructure. This highlights the need for targeted public health interventions that extend beyond general sanitation promotion. It is essential to prioritize equal access to latrines and ensure their sustained use at the household level, especially in high-transmission areas where environmental contamination continues to pose a significant challenge.

- **"Hand hygiene practices, especially the failure to wash hands after using the latrine, were significantly associated with the prevalence of STH, which aligns with prior global studies (52–54). This highlights the importance of hand washing as a key factor in preventing the transmission of STH (55). In contrast to previous research in the same area, hand hygiene showed no significant effect (22). This divergence may be due to variations in study design, changes in hygiene behaviors over time, or previous underestimations of individual practices. Furthermore, recent improvements in sanitation infrastructure, in the absence of corresponding behavioral changes, could increase the relative impact of hand hygiene on the risk of infection. These findings underscore the**

complex interaction between environmental and behavioral factors that influence the transmission of soil-transmitted helminths (STH) and emphasize the importance of ongoing local assessments.

- We were advised to consider potential confounding factors, such as frequency of water contact; however, this variable was not included in the final analysis as it was removed after the pretest due to participants' poor recall.

5. Prevalence and intensity: The reported prevalence of S. mansoni (20.1% by Kato-Katz) is described as "moderate," but the WHO threshold for moderate prevalence is ≥10% and <50%. The authors should explicitly state how their findings align with WHO guidelines for mass drug administration (MDA). Again, the intensity classification (light, moderate, heavy) is based on WHO criteria, but the geometric mean egg count (139.39 EPG) suggests a skewed distribution. The authors should discuss whether this affects the interpretation of intensity.

As we were asked to explicitly state how the 20.1% prevalence of S. mansoni aligns with WHO guidelines for MDA, we note that this has already been addressed in the discussion section of the previously submitted manuscript. We were also asked whether the interpretation of infection intensity was affected by the skewed distribution when using arithmetic and geometric means. we addressed this in lines 420 –423 of the revised manuscript as follows: "**The noticeable discrepancy between the arithmetic and geometric means reflects the presence of a right-skewed distribution of egg counts. This pattern is consistent with the typical epidemiology of schistosomiasis, where a minority of individuals harbor high worm burdens, while the majority exhibit low to moderate infection levels** (42)**.**

6. Limitations: The cross-sectional design and purposive school selection are noted as limitations, but the authors should also address potential biases (e.g.,

seasonal variations in transmission, non-random sampling). Again, the use of a single stool sample for Kato-Katz may underestimate prevalence due to day-to-day variation in egg excretion. This should be discussed as a limitation.

Regarding the comments on the limitations section of the manuscript, we addressed this in lines 551–554 in the revised manuscript as follows: "**This study has a few limitations: (i), the cross-sectional design provides a snapshot of *S. mansoni* and STH infection at a single point in time, which may not capture seasonal variations or fluctuations in infection rates over longer periods. (ii), the use of a single stool sample per participant for the KK technique may have led to underestimation of infection prevalence and intensity, given the known day-to-day variability in helminth egg excretion. (iii), the purposive selection of schools limits the generalizability of the findings to the larger community in the study area.**

7. Minor Comments: (i) Abstract: The abstract is comprehensive but could be more concise. The methods section, for example, includes unnecessary details (e.g., SPSS version). Again, the conclusion should avoid redundancy (e.g., "necessity for additional control measures" is repeated). (ii) Tables and Figures: Table 1 (infection intensity) is clear but could be merged with Table 2 (socio-demographic factors) to streamline the presentation. Again, Figure 3 (helminth distribution) is informative but would benefit from a clearer legend (e.g., full parasite names instead of abbreviations). (iii) Writing and Clarity: Some sentences are overly long or awkwardly phrased (e.g., "The absence of a latrine at home emerged as a prominent risk factor for STH infection"). Editing for conciseness would improve readability. Again, the abbreviation "STH" is used throughout but should be defined at first mention in the abstract and main text. (iv) References: References are generally appropriate, but some are outdated (e.g., WHO guidelines from 2011). The authors should cite more recent WHO

publications (e.g., 2022 guidelines). Again, a few references are incomplete (e.g., "Weekly epidemiological record Relevé épidémiologique hebdomadaire" lacks volume/issue details).

The minor comments have all been addressed in the revised manuscript, except for merging the tables, which we chose not to do because the current format is clearer and merging would make the presentation more complicated.

(i) Abstract: Data were processed in SPSS v26 using logistic regression, with p<0.05 considered statistically significant paraphrased to Data was analysed using SPSS and McNemar's chi-square test (line numbers 53 – 54). In conclusion, part necessity is deleted

(ii) We prefer to retain Table 1, which has been changed to Table 2 in the revised manuscript, as it aligns with the above explanation.
Figure 3 for helminthic distribution. We revised the figure and wrote the full name of the parasite rather than using the legend. See the revised Fig 3

(iii) Writing and Clarity: Once again, we revised the whole manuscript document for clarity, abbreviation consistency, and scientific parasite naming

(iv) We also revised the reference and made necessary corrections in the revised manuscript (line numbers 545 – 547)

While revising your submission, please upload your figure files to the Preflight Analysis and Conversion Engine (PACE) digital diagnostic tool, https://pacev2.apexcovantage.com/. PACE helps ensure that figures meet PLOS requirements. To use PACE, you must first register as a user. Registration is free. Then, login and navigate to the UPLOAD tab, where you will find detailed instructions on how to use the tool. If you encounter any issues or have any questions when using PACE, please email PLOS at figures@plos.org. Please note that Supporting Information files do not need this step.
