# Supplementary material for: Assessment of Schistosoma mansoni and soil-transmitted helminth infections and the diagnostic performance of the circulating cathodic antigen test among schoolchildren in Tulla district, Sidama region, Southern Ethiopia
Source: PLoS One. 2025 Dec 12;20(12):e0337919. doi: 10.1371/journal.pone.0337919 (PMC12700372; doi:10.1371/journal.pone.0337919)
Supplement: Attachment — Submitted filename: PONE-D-25-23474_R2.pdf [file pone.0337919.s008.pdf]

PLOS ONE

Assessment of Schistosoma mansoni and Soil-transmitted Helminth Infections and the Diagnostic Performance of the Circulating Cathodic Antigen Test among Schoolchildren in Tulla District, Sidama Region, Southern Ethiopia.

--Manuscript Draft--

**Manuscript Number:**	PONE-D-25-23474R2
**Article Type:**	Research Article
**Full Title:**	Assessment of Schistosoma mansoni and Soil-transmitted Helminth Infections and the Diagnostic Performance of the Circulating Cathodic Antigen Test among Schoolchildren in Tulla District, Sidama Region, Southern Ethiopia.
**Short Title:**	Schistosoma mansoni Infection and Performance its antigen test
**Corresponding Author:**	Bamlaku Tadege Tadege, PhD<br>Hawassa University College of Medicine and Health Sciences<br>Hawassa, ETHIOPIA
**Keywords:**	Prevalence; Infection intensity; risk factors. Schistosoma mansoni; Soil-Transmitted Helminth; Diagnostic performance; Kato-Katz technique; Point-of-care circulating cathodic antigen, Tulla district
**Abstract:**	Background: Soil-transmitted helminths (STH) and Schistosoma mansoni pose significant public health challenges in areas with poor sanitation. While S. mansoni infection leads to chronic illness and mortality, research on its intensity remains limited in the study area. Diagnosis traditionally relies on the Kato-Katz method, though the POC-CCA urine test offers a rapid alternative. This study examined the prevalence, intensity, and risk factors of S. mansoni and Soil-transmitted helminth infections among schoolchildren while evaluating the diagnostic performance of the POC-CCA test.<br>Methods: A school-based cross-sectional study was conducted in the Tulla district, Sidama region, among 477 students (April–June 2024). The Schoolchildren were recruited using a purposive sampling technique. Socio-demographic data were collected using pre-structured questionnaires. A single stool sample was collected from each study participant and processed for the detection of helminth eggs using the Kato-Katz (KK) technique. Additionally, urine samples were tested with the Point of Care Circulating Cathodic Antigen Test (POC-CCA), which detects Schistosoma antigens. Data was analyzed using SPSS and McNemar's chi-square test.<br>Result: The overall prevalence of Schistosoma mansoni was 36.5% (20.1% by KK and 34.4% by POC-CCA). The prevalence of STH was 48.8%. The leading parasite was Ascaris lumbricoides (34%), followed by Trichuris trichuria (8.2%) and hookworm (5.7%). Other helminths included Taenia species 1.3%, Hymenolepis nana 0.6%, and Enterobius vermicularis 0.2%. Infection intensity of S. mansoni was classified as light 34.4%, moderate 56.2%, and heavy 9.4%. Involvement in irrigation, swimming, bathing, and school location were significantly associated with S. mansoni infection. School location, educational status, lack of latrine, hand washing practice and fingernail trimming practice were significantly associated with STH infections. The POC-CCA rapid test had a significantly higher sensitivity of 89.6% (McNemar test χ^2m = 52.3, $p < 0.001$) compared to the Kato-Katz technique.<br>Conclusions: Moderate S. mansoni and STH infection and poor hygiene practice underscore for additional control measures. Incorporating POC-CCA testing alongside the KK method could optimize diagnostic accuracy and improve disease management outcomes.
**Order of Authors:**	Bamlaku Tadege Tadege, PhD
	Fitsum Getachew Getachew, MSC
	Demissie Assegu Fenta, MSC
	Biniyam Kijineh Kijineh, MSC
**Response to Reviewers:**	PONE-D-25-23474R1<br>Assessment of Schistosoma mansoni and Soil-transmitted Helminth Infections and the Diagnostic Performance of the Circulating Cathodic Antigen Test among

Powered by Editorial Manager® and ProduXion Manager® from Aries Systems Corporation

Schoolchildren in Tulla District, Sidama Region, Southern Ethiopia. PLOS ONE

Response to the PLoS ONE editor and reviewers' comments and questions
First and foremost, we sincerely thank you for reviewing our manuscript and offering valuable comments and suggestions. Your insights have been very helpful in highlighting areas for improvement, and we have carefully addressed each of the points you raised. We respond to each comment, suggestion and question raised by reviewers, and these responses are highlighted with green colour.
We thank you for your scientific contribution
Journal Requirements:
If the reviewer comments include a recommendation to cite specific previously published works, please review and evaluate these publications to determine whether they are relevant and should be cited.
Recommendation given by reviewer I to state background information if the two organisms are problems in the area under study. Information on ongoing interventions (in terms of number of rounds of treatment, Vector WASH services) on these infections should be mentioned and implications for the prevalence recorded in the present study should be investigated and reported.
We include the current global controlling strategies and background information on the prevalence of STH and Schistosoma in Ethiopia and in the current study area and control intervention measures at the national level and in the region of our study area in the introduction part from line number 118 to 147 which is highlighted in revised marked manuscript and we cite new references used for background information (reference numbers 14 – 20) in the reference section
Please review your reference list to ensure that it is complete and correct. If you have cited papers that have been retracted, please include the rationale for doing so in the manuscript text, or remove these references and replace them with relevant current references. Any changes to the reference list should be mentioned in the rebuttal letter that accompanies your revised manuscript. If you need to cite a retracted article, indicate the article's retracted status in the References list and also include a citation and full reference for the retraction notice.
We cited references which are relevant to our manuscript. All references cited in this manuscript are revised to ensure its completeness and correctness to fit the PLOS ONE reference citation procedure; for this, it is highlighted in the revised marked references section

1. If the authors have adequately addressed your comments raised in a previous round of review and you feel that this manuscript is now acceptable for publication, you may indicate that here to bypass the "Comments to the Author" section, enter your conflict of interest statement in the "Confidential to Editor" section, and submit your "Accept" recommendation.
Reviewer #1: All comments have been addressed
Reviewer #3: (No Response)
Reviewer #4: (No Response)
We revised the manuscript to address the constructive comments and suggestions provided by the reviewers. Thank you for your time and dedication

____

2. Is the manuscript technically sound, and do the data support the conclusions?

The manuscript must describe a technically sound piece of scientific research with data that supports the conclusions. Experiments must have been conducted rigorously, with appropriate controls, replication, and sample sizes. The conclusions must be drawn appropriately based on the data presented.
Reviewer #1: Yes
Reviewer #3: Yes
Reviewer #4: Partly
We revised the comments raised by the reviewer concerning this part and conclusion part is revised in advance

____

3. Has the statistical analysis been performed appropriately and rigorously?
Reviewer #1: Yes
Reviewer #3: No
Reviewer #4: Yes

We thank you all reviewers
In the revised manuscript, we clearly explain why we apply McNemar's test analysis and its purpose. We also explain the purpose of using logistic regression analysis which is indicated in data analysis section

____
4. Have the authors made all data underlying the findings in their manuscript fully available?

The PLOS Data policy requires authors to make all data underlying the findings described in their manuscript fully available without restriction, with rare exception (please refer to the Data Availability Statement in the manuscript PDF file). The data should be provided as part of the manuscript or its supporting information, or deposited to a public repository. For example, in addition to summary statistics, the data points behind means, medians and variance measures should be available. If there are restrictions on publicly sharing data—e.g. participant privacy or use of data from a third party—those must be specified.
Reviewer #1: Yes
Reviewer #3: Yes
Reviewer #4: Yes
We thank you all reviewers
____
5. Is the manuscript presented in an intelligible fashion and written in Standard English?

PLOS ONE does not copyedit accepted manuscripts, so the language in submitted articles must be clear, correct, and unambiguous. Any typographical or grammatical errors should be corrected at revision, so please note any specific errors here.
Reviewer #1: Yes
Reviewer #3: Yes
Reviewer #4: Yes
We thank all reviewers
6. Review Comments to the Author

Please use the space provided to explain your answers to the questions above. You may also include additional comments for the author, including concerns about dual publication, research ethics, or publication ethics. (Please upload your review as an attachment if it exceeds 20,000 characters)
Reviewer #1: The authors have adequately addressed the comments raised previously; however there few comments that need to be clarified in the current review. I found the manuscript technically sound and have value for policy and public health practice.
Thank you, Reviewer. We include the current global controlling strategies and background information on the prevalence of STH and Schistosoma in Ethiopia and in the current study area and control intervention measures at the national level and in the region of our study area in the introduction part from line number 118 to 147 which is highlighted in revised marked manuscript and we cited new references used for background information (reference numbers 14 – 20) in the reference section. We also added additional keywords significantly related to the study findings. We agreed to keep the research title as it is. ____
Reviewer #3: I advise the author(s) to take into account the following issues even if they made revisions to the text in response to the feedback and recommendations of earlier reviewers:
1. Uncorrected or Inaccurate Responses to Reviewers:
Ethical approval reference still unclear despite E.C. explanation; conversion not standardly recognized without explicit note.
Thank you, Reviewer, for your time and concern
In our initial submission, we uploaded the ethical clearance approval letter provided by the IRB of Hwassa University College of Medicine and Health Science, which was issued in 2024. So, I missed uploading the ethical clearance approval letter in the second submission. In the current revised manuscript submission, I have uploaded approval letter to the other files. The reference number provided on the ethical clearance application letter is not related to the calendar. Instead, they are unique code number assigned to the ethical approval letter by the IRB. If you require confirmation,

	you can contact the IRB office at this email address: embialle@hu.ede.et<br>Data availability statement contradictory: claims data in SI, but no files uploaded; "available upon request" violates PLOS policy.<br>Raw data not publicly accessible; IRB restriction cited but not justified sufficiently for non-public sharing.<br>No supplementary files provided despite mention of questionnaire and raw data.<br>All data used for this manuscript have been submitted. We provided the raw data and questionnaire in a supplementary file during the second manuscript submission and we have no remaining data to submit. We have resubmitted the raw data and questionnaire in the supplementary files in the current revised manuscript submission.<br>Use of McNemar's test inappropriate for described analysis; original use of logistic regression more suitable, change unexplained.<br>We used SPSS regression analysis for descriptive analysis and for the significant association between risk factors and infection, whereas the McNemar chi-square test was employed to compare the statistically significant differences in sensitivity and specificity between the two diagnostic methods, focusing on cases where the tests disagree. This is clearly described in the data analysis section of this revised manuscript.<br><br>2. New Comments/Questions to Improve Quality:<br>Correct inconsistent verb tense usage (e.g., "data was analysed" → "data were analysed").<br>Subject-verb agreement is corrected in the revised manuscript<br><br>Clarify statistical methods: justify the switch from logistic regression to McNemar's test.<br>This one is described above; We used SPSS regression analysis for descriptive analysis and for the significant association between risk factors and infection, whereas the McNemar chi-square test was employed to compare the statistically significant differences in sensitivity and specificity between the two diagnostic methods, focusing on cases where the tests disagree. This is clearly described in the data analysis part of this revised manuscript.<br><br>Fix subject-verb agreement errors (e.g., "the results shows" → "the results show").<br>Subject-verb agreement is corrected in the revised manuscript<br><br>Provide DOI or repository link for raw data to comply with PLOS policy.<br>Our college is planning to develop a repository link; for this, we have submitted raw data in a supplementary file in this revised manuscript.<br>Include full ethical approval document with date conversion proof as supplementary.<br>This is described above. We uploaded the ethical clearance approval letter provided by the IRB of Hwassa University College of Medicine and Health Science, which was issued in 2024 in this revised manuscript. We address this issue in the ethical part.<br>Ethical clearance was obtained from Hawassa University, College of Medicine, and the other Health Sciences Internal Review Board (IRB) with a reference number: IRB/172/16 in 2024. If you require confirmation, you can contact the IRB office at this email address.<br>Define the assent process clearly for children under 12 in methods<br>We explain the assent process clearly for children under 12 in methods as follow:<br>Explicit written consent was not obtained for children under 12 years, as they may lack the developmental capacity to understand this concept. However, these children were verbally informed about the study in simple language, and their willingness or unwillingness to participate was respected. This is included in the ethical clearance part<br>Update all WHO references to 2022 guidelines and complete missing citation details.<br>We revised not only the WHO references but all other references following the Vancouver citation style, following PLOS ONE guidelines, which are highlighted in marked revised manuscript.<br>WHO updated citation references include reference numbers 1 and 4 in the revised manuscript<br><br>Reviewer #4: Major Comments<br>1. What is your main objective? This might result in major revision of the whole MS contents.<br>a. To assess the prevalence of S. mansoni and STHs (POC-CCA is additional)

Powered by Editorial Manager® and ProduXion Manager® from Aries Systems Corporation

b. To evaluate the diagnostic performance of KK vs POC-CCA (STH is additional)
c. ???
The objectives of this study are:
To assess the prevalence of S. mansoni and STH infections.
To assess risk factors for STH and S. manson infections
To evaluate the intensity of S. mansoni infection
To compare the diagnostic performance of POC-CCA test to that of KK.
We clearly address these objective in the abstract and last paragraph of introduction section

2. Your result part is highly fragmented or not well organised: needs reshuffling.
We revised the results section and reorganized it to make the information clearer and more attractive for the reader.
3. What is the relevance of your study
Our study relevant to current WHO NTD control program as regional and local information are important assess ongoing controlling stratigies for the following reasons:
1.WHO has launched deworming programs for Schistosomiasis and soil-transmitted helminths targeting vulnerable school-age children? Although these deworming efforts have successfully reduced morbidity caused by Schistosoma and STH, re-infection continues in the absence of complementary measures to sustain deworming. This study is important for providing valuable supplementary interventions in the regional study area.
2.Intestinal schistosomiasis and soil-transmitted helminth infections are diagnosed by detecting parasite eggs in stool samples through microscopic methods, primarily using the Kato-Katz (KK) technique. Recommended by the WHO, this method is commonly used in epidemiological surveys. However, the Kato-Katz technique has low sensitivity, making diagnosis difficult in areas with low prevalence and decreased worm loads. This difficulty results from several factors, including uneven egg distribution in stool samples, daily variations in egg excretion, and the random nature of egg dispersal. A more sensitive diagnostic method, called the POC-CCA test, has been developed to detect S. mansoni antigens in urine samples, especially useful in low-endemic areas, either as a replacement or supplement to the Kato-Katz technique. Nonetheless, the diagnostic accuracy of this method has not been thoroughly evaluated across different regions. This study is relevant to address this gap.

4. Your discussion is precise and not attractive to your readers.
We revised the discussion section and provided detailed descriptions of specific subtopics, which are highlighted in the marked revised manuscript. Additionally, we added subsections to improve readability (see from number line 563 – 606 in the marked revised manuscript)
Minor comments
Title
1. What do you mean by short title?
Short title is requested by PLoS ONE submission guidelines, which means writing the research title in a short sentence, keeping the main idea of the original research title
2. Check affiliation 3
We check, and it is correct
Abstract
1. A sentence but two different ideas. While S. mansoni infection leads to chronic illness and mortality, research on its intensity remains limited in the study area.’
We revised it and cleared the sentences in the current revised manuscript in the abstract part
2. Write acronyms of Point of Care Circulating Cathodic Antigen Test (POC-CCA) in its first appearance (in the background section)
We corrected acronyms
3. Modify your language throughout your entire document (e.g. a school-based cross-sectional study was conducted among 477 school children from April to June 2024 in Tulla district, Sidama Region, Southern Ethiopia).
Once again, we revised our manuscript throughout the document and marked the changes in the revised document.
4. Modify: Urine samples were also collected from the study participants and tested using POC-CCA for the detection of Schistosoma antigens.
We changed to urine samples were analyzed with POC-CCA technique.

Powered by Editorial Manager® and ProduXion Manager® from Aries Systems Corporation

	5. Other helminths included … seams meaningless??<br>Yes, it may not be clear to all readers, so we removed others in the abstract part and replaced them with the name of the parasite species in the revised manuscript section<br>6. Avoid vague sentences: e.g. 'Moderate S. mansoni and STH infection and poor hygiene practice underscore for additional control measures.<br>Yes, we revised and cleared the sentence in the revised manuscript, which is highlighted in the marked manuscript<br>7. Your conclusion is not attractive.<br>We revised and rewrote the conclusion section (from the number line 747 – 760 in the revised marked manuscript<br>8. Reconsider your keywords (read about how to select keywords).<br>We considered and revised keywords in the revised manuscript<br>9. General comment: Critical language problems<br>We revised the manuscript once again to clear grammar errors and language issues, to eliminate specific errors and ambiguity. Any typographical or grammatical mistakes have been corrected during the revision<br>Introduction<br>Background<br>We deleted the word background and kept the Introduction in the revised manuscript<br>1. Put references in [..].<br>We put the reference number in [ ] in a manuscript word document<br>2. Paragraph 5 (line 106-111): It looks like you are talking about your own study<br>Thank you, we revised the paragraph (from line number 162 – 166) in the marked revised manuscript<br>3. The last paragraph: It is the main objective of your study, but not well structured, and I recommend re-write. Are you comfortable with 'to evaluate the prevalence…'? Also check your language (S. mansoni, S. mansoni).<br>Method<br>1. … among four governmental elementary school children (schools)<br>Corrected as: A school-based cross-sectional study was conducted from April to June 2024 among children in four governmental elementary schools in the Tulla district.<br><br>2Add a sample collection portion and clearly indicate how you collected stool and urine samples from your study participants.<br>Stool and urine sample collection procedures are described in the manuscript from lines 244 – 253. Subtitle is given as Stool and Urine Sample Collection, in the revised marked manuscript<br><br><br>3. Infection intensities: You did categories (light, moderate and heavy) only for S. mansoni?<br>Yes, our research mainly focused on the prevalence, intensity, and performance of two diagnostic methods on S. mansoni. We only assess prevalence and associated risk factors of STH. Due to time constraints and resource limitations, we did not assess the intensity of STH<br>4. What is the relationship between quality and sample collection time?<br>Quality in a laboratory refers to its capacity to consistently produce accurate, reliable, and timely test results. The timing of sample collection is a crucial aspect of this quality, as it directly impacts the accuracy and reliability of the test outcomes. Furthermore, it is an important factor that is monitored throughout the entire testing process, from collection to interpretation. For instance, in our research context, maintaining proper timing for urine sample collection enhances the quality of the laboratory results.<br>5. Data analysis: indicate your reference test and give details about your analysis.<br>The data analysis and the reference test used for this study (the KK) are explained in the revised manuscript and highlighted in the marked manuscript from line number 287 to 315 in the analysis section<br><br>Results<br>1. I prefer if you put your results of diagnostics for S. mansoni and then to risk factors, STH,…<br>The authors agreed to put the order as follows: economic information, prevalence of S. mansoni, risk factors of S. mansoni, Intensity of S. mansoni, Prevalence of STH, risk factors of STH and finally, comparison of two diagnostic methods on S. mansoni detection

Powered by Editorial Manager® and ProduXion Manager® from Aries Systems Corporation

	2. Table 1: move it under the risk factors sub-title.<br>We put Table 1 under risk factors<br>3. What is the importance of Table 2?<br>The importance of Table 2 is to show intensity variations across sex, age, and children's school attendance location
Additional Information:	
**Question**	**Response**
**Financial Disclosure**<br><br>Enter a financial disclosure statement that describes the sources of funding for the work included in this submission. Review the submission guidelines for detailed requirements. View published research articles from *PLOS ONE* for specific examples.<br><br>This statement is required for submission and **will appear in the published article** if the submission is accepted. Please make sure it is accurate.<br><br>**Funded studies**<br>Enter a statement with the following details:<br>• Initials of the authors who received each award<br>• Grant numbers awarded to each author<br>• The full name of each funder<br>• URL of each funder website<br>• Did the sponsors or funders play any role in the study design, data collection and analysis, decision to publish, or preparation of the manuscript?<br><br>Did you receive funding for this work?	The author(s) received no specific funding for this work.
**Competing Interests**<br><br>Use the instructions below to enter a competing interest statement for this submission. On behalf of all authors, disclose any competing interests that could be perceived to bias this work—acknowledging all financial support and any other relevant financial or non-financial competing interests.	There is no any competing interest for this research. All authors dead and agreed to submit this article to PLoS ONE journal. We read also the journal guideline

This statement is required for submission and **will appear in the published article** if the submission is accepted. Please make sure it is accurate and that any funding sources listed in your Funding Information later in the submission form are also declared in your Financial Disclosure statement.<br><br>View published research articles from *PLOS ONE* for specific examples.<br><br>**NO authors have competing interests**<br><br>Enter: *The authors have declared that no competing interests exist.*<br><br>**Authors with competing interests**<br><br>Enter competing interest details beginning with this statement:<br><br>*I have read the journal's policy and the authors of this manuscript have the following competing interests: [insert competing interests here]*<br><br>* typeset	
**Ethics Statement**<br><br>Enter an ethics statement for this submission. This statement is required if the study involved:<br><br>• Human participants<br>• Human specimens or tissue<br>• Vertebrate animals or cephalopods<br>• Vertebrate embryos or tissues<br>• Field research<br><br>Write "N/A" if the submission does not require an ethics statement.<br><br>General guidance is provided below. Consult the submission guidelines for detailed instructions. **Make sure that all information entered here is included in the Methods section of the manuscript.**	Ethical clearance was obtained from Hawassa University, College of Medicine, and the other Health Sciences Internal Review Board (IRB) with a reference number: IRB/172/16 in 2024. A letter of support from Hawassa University School of Medical Laboratory was provided to the Sidama Regional State Health Bureau, as well as to the health and education offices in Tulla district and the directors of the selected schools. Participation was fully voluntary, with written informed consent obtained from the parents/guardians of the children enrolled. An additional separate written assent was secured from children older than 12 years. Explicit written assent was not obtained for children under 12 years, as they may lack the developmental capacity to understand this concept. However, these children were verbally informed about the study in simple language, and their willingness or unwillingness to participate was respected.<br>Schoolchildren who tested positive for any pathogenic intestinal parasites received appropriate treatment administered by qualified public health professionals. All data collected during the study were kept strictly confidential and used exclusively for research purposes. The study procedures were conducted in full compliance with relevant ethical guidelines and regulatory standards

Powered by Editorial Manager® and ProduXion Manager® from Aries Systems Corporation

**Format for specific study types**<br><br>**Human Subject Research (involving human participants and/or tissue)**<br>• Give the name of the institutional review board or ethics committee that approved the study<br>• Include the approval number and/or a statement indicating approval of this research<br>• Indicate the form of consent obtained (written/oral) or the reason that consent was not obtained (e.g. the data were analyzed anonymously)<br><br>**Animal Research (involving vertebrate animals, embryos or tissues)**<br>• Provide the name of the Institutional Animal Care and Use Committee (IACUC) or other relevant ethics board that reviewed the study protocol, and indicate whether they approved this research or granted a formal waiver of ethical approval<br>• Include an approval number if one was obtained<br>• If the study involved *non-human primates*, add *additional details* about animal welfare and steps taken to ameliorate suffering<br>• If anesthesia, euthanasia, or any kind of animal sacrifice is part of the study, include briefly which substances and/or methods were applied<br><br>**Field Research**<br><br>Include the following details if this study involves the collection of plant, animal, or other materials from a natural setting:<br>• Field permit number<br>• Name of the institution or relevant body that granted permission	
**Data Availability**<br><br>Authors are required to make all data underlying the findings described fully available, without restriction, and from the time of publication. PLOS allows rare exceptions to address legal and ethical concerns. See the PLOS Data Policy and FAQ for detailed information.	Yes - all data are fully available without restriction

Powered by Editorial Manager® and ProduXion Manager® from Aries Systems Corporation

A Data Availability Statement describing where the data can be found is required at submission. Your answers to this question constitute the Data Availability Statement and **will be published in the article**, if accepted.<br><br>**Important:** Stating 'data available on request from the author' is not sufficient. If your data are only available upon request, select 'No' for the first question and explain your exceptional situation in the text box.<br><br>Do the authors confirm that all data underlying the findings described in their manuscript are fully available without restriction?	
**Describe where the data may be found in full sentences. If you are copying our sample text, replace any instances of XXX with the appropriate details.**<br><br>• If the data are **held or will be held in a public repository**, include URLs, accession numbers or DOIs. If this information will only be available after acceptance, indicate this by ticking the box below. For example: *All XXX files are available from the XXX database (accession number(s) XXX, XXX.*).<br>• If the data are all contained **within the manuscript and/or Supporting Information files**, enter the following: *All relevant data are within the manuscript and its Supporting Information files.*<br>• If neither of these applies but you are able to provide **details of access elsewhere**, with or without limitations, please do so. For example:<br><br>*Data cannot be shared publicly because of [XXX]. Data are available from the XXX Institutional Data Access / Ethics Committee (contact via XXX) for researchers who meet the criteria for access to confidential data.*<br><br>*The data underlying the results presented in the study are available*	All relevant data are within the paper and its Supporting Information files

from (include the name of the third party and contact information or URL).<br>• This text is appropriate if the data are owned by a third party and authors do not have permission to share the data.<br><br>* typeset	
Additional data availability information:	Tick here if your circumstances are not covered by the questions above and you need the journal’s help to make your data available.

Powered by Editorial Manager® and ProduXion Manager® from Aries Systems Corporation

Manuscript
Click here to access/download;Manuscript;Unmarked revised manuscript final.docx

Assessment of *Schistosoma mansoni* and Soil-transmitted Helminth Infections and the Diagnostic Performance of the Circulating Cathodic Antigen Test among Schoolchildren in Tulla District, Sidama Region, Southern Ethiopia.

Short title: *Schistosoma mansoni* Infection and Performance of its antigen test

Fitsum Getachew[1,2*], Demissie Assegu[1], Biniyam Kijineh[3] and Bamlaku Tadege[1*]

[1] School of Medical Laboratory Sciences, Hawassa University, Hawassa, Ethiopia.

[2] School of Medical Laboratory Sciences, Wolaita Sodo University, Wolaita, Ethiopia.

[3] School of Medical Laboratory Sciences, Wachemo University, Ethiopia, Hossana, Ethiopia.

*E-mail of corresponding author: bamlaku22tadege@gmail.com

Consent for publication

All authors have read the manuscript and consented to submit the manuscript.

Competing interests

All authors declare that they have no competing interests.

Authors' contributions

FG: conceptualization, data curation, formal analysis, investigation, methodology, writing – original draft; DA & BK: writing – review & editing, BT: conceptualization, supervision, writing – review & editing, data curation, and formal analysis.

Authors' addresses

fitsumgetachew6@gmail.com (FG)

bamlaku22tadege@gmail.com (BT)

demisissieasegu@yahoo.com (DA)

kijinehb@gmail.com (BK)

Funding

This study was funded by Hawassa University

Abstract

Background: STH and *S. mansoni* pose significant public health challenges in regions with inadequate sanitation. Existing research on *S. mansoni* intensity remains limited in the study area. *S. mansoni* diagnosis traditionally relies on the KK method, though the POC-CCA urine test offers a rapid alternative with high sensitivity. This study aimed to assess the prevalence and risk factors of *S. mansoni* and STH infections. It also evaluated the intensity of *S. mansoni* and compared the performance of the POC-CCA test to that of KK.

Methods: A cross-sectional study was conducted among schoolchildren in the Tulla district, Sidama region, from April to June 2024, using a purposive sampling approach. Data were collected through pre-structured questionnaires, and stool samples were analyzed using the KK method, while urine samples were analyzed with the POC-CCA technique. A logistic regression model was employed to examine potential associations between infections and risk factors, the Kappa statistic assessed agreement between tests, and the McNemar chi-square test compared the sensitivity and specificity of the diagnostic methods.

Result: The prevalence of *S. mansoni* with two combined tests was 36.5% (20.1% by KK and 34.4% by POC-CCA). Activities like irrigation, swimming, and bathing showed significant associations with *S. mansoni* infection. The prevalence of STH was 48.8%, with A. lumbricoides (34%) as the most common, followed by *T. trichuria* (8.2%), hookworm (5.7%), *Taenia* species (1.3%), *H. nana* (0.6%), and *E.vermicularis* (0.2%). Factors like hand washing, fingernail trimming, lack of latrines, and educational status were significantly linked to STH infections. The POC-CCA test demonstrated higher sensitivity (89.6%) than the KK technique (McNemar test χ^2m = 52.3, $p < 0.001$).

Conclusions: The moderate prevalence of *S. mansoni* and STH infections, coupled with socio-demographic factors, behaviours, hygiene practices, and sanitation issues associated with these infections, highlights the need for additional control measures beyond deworming. Implementing a highly sensitive POC- CCA test alongside the KK method in low-endemic areas could improve diagnostic accuracy and enhance disease management outcomes.

Keywords: Prevalence, intensity, risk factor, *Schistosoma mansoni,* Soil-transmitted helminth, Kato-Katz, Point-of-care circulating cathodic antigen, Tulla district, Ethiopia

Introduction

Schistosoma and soil-transmitted helminth (STH) are worm infections that belong to the group of neglected tropical diseases (NTDs) [1]. *Schistosoma mansoni* (*S. mansoni*) is a water-based, vector-borne parasite transmitted indirectly through freshwater snails [2]. People living in areas with inadequate water and sanitation systems are particularly susceptible to this most common and deadly infection [3]. Globally, schistosomiasis is recognized as endemic in 78 low- and middle-income countries in tropical and subtropical areas, estimated to affect more than 251 million people [4]. Furthermore, 243 million people require preventative chemotherapy, including 111 million school-age children, of which 226 million are in Africa [5]. It causes significant morbidity, with an estimated 1.4-3.3 million disability-adjusted life years (DALYs) lost annually due to its health impacts [6].

STHs are intestinal parasitic worms transmitted to humans through fecally contaminated soil [7]. The main STHs that affect humans are *Ascaris lumbricoides* (A. *lumbricoides*), *Trichuris trichuria (*T. *trichuria), Necator americanus, and Ancylostoma duodenale* [8]. These parasites cause gastrointestinal infections and are most commonly found in tropical and subtropical regions, particularly impacting impoverished nations where schoolchildren are especially vulnerable [9].

Globally, an estimated 1.5 billion people are infected with STHs, including 654 million school-aged children living in areas where these parasites are prevalent [10]. The Burden of *Schistosoma*

and STHs leads to various health issues, including gastrointestinal bleeding, nutrient malabsorption, loss of appetite, anemia due to the depletion of iron and other essential proteins, growth retardation, impaired cognitive development, school absenteeism, and disability-adjusted life-years lost [11], [12], [13].

The World Health Organization (WHO) recommends integrated strategies for controlling and eliminating schistosomiasis and STH. These strategies include preventive chemotherapy (PC), which consists of periodic administration of anthelmintic medicines praziquantel for schistosomiasis and albendazole or mebendazole for STH. Additionally, improvements in water, sanitation, and hygiene (WASH), behavior change, snail control, and environmental management are essential, as deworming alone cannot prevent re-infection [14], [15].

In Ethiopia, an estimated 79 million people live in areas endemic for STHs, and 37.3 million live in regions affected by *S. mansoni*, with the majority being school-aged children—specifically, 25.3 million and 12.3 million children, respectively [16]. National mapping surveys conducted between 2013 and 2015 across Ethiopia's regional states revealed a prevalence of 21.7% for STHs and 3.5% for *S. mansoni* infections among school-aged children [17]. In 2015, Ethiopia launched a national control program aimed at eliminating *S. mansoni* and STHs as serious public health concerns by 2020, with the goal of breaking transmission by 2025 [18]. The program aims to treat at least 75% of school-aged children and to extend treatment to adolescents and adults. It also seeks to integrate initiatives for neglected tropical diseases and strengthen collaboration with mass drug administration efforts. Furthermore, it aims to enhance cooperation with WASH programs [16]. However, the WASH program has limited coverage, particularly in rural areas. This issue is primarily attributed to insufficient collaboration and the high financial costs associated with running the program in Ethiopia.

Results from systematic reviews in Ethiopia have shown that helminth control interventions have significantly reduced the prevalence of STH and *S. mansoni* infections over the years in Ethiopia[3], [18]. Additionally, a survey conducted among schoolchildren in our study area revealed a notable decline in the prevalence of STHs (52.4%) and *S. mansoni* (31%) in 2015 compared to the rates in 2007, which were 67.3% for STHs and 76.5% for *S. mansoni* [19], [20]. There is annual deworming programme for school children in our study area to control STH and

S. mansoni. However, the current status of infection intensity in the study area, following the increase in helminth control interventions, remains unknown.

Intestinal *Schistosoma* is diagnosed by microscopic detection of parasite eggs in stool using the Kato-Katz (KK) technique, which is widely used in epidemiological surveys [21]. However, its diagnosis is challenging in low-endemic areas where prevalence and worm burden are reduced, due to factors like uneven egg distribution in stool samples, daily variations in egg excretion, and random distribution effects [22], [23]. The point-of-care (POC) cassette test is a relatively new diagnostic tool that detects circulating cathodic antigens (CCA) in urine samples, offering higher sensitivity and operational benefits [23], [24]. A past study found that POC-CCA was more sensitive (86% vs. 62%) but less specific (72% vs. ~100%) compared to multiple KK smears from a single stool sample. A multivariable modelling study suggested that POC-CCA was significantly more sensitive than KK for detecting low infection intensities (<100 eggs/gram of stool) [25]. A previous study recommended assessing the POC-CCA assay before fully utilizing it for monitoring and evaluating control programs [26].

Therefore, this study aimed to assess the prevalence and risk factors of *S. mansoni* and STH infections among schoolchildren. It also aimed to assess the intensity of *S. mansoni* and compare the diagnostic performance of the POC-CCA test with KK.

Materials and Methods

Study design and setting

A school-based cross-sectional study was conducted from April to June 2024 among children in four governmental elementary schools in the Tulla district. The study was conducted in four elementary schools in the Tulla district, Sidama region in Southern Ethiopia. The district is situated along the shore of Lake Hawassa and the Tikur Wuha River, approximately 289 kilometers south of Addis Ababa, the capital city of Ethiopia. The altitude is 1800 meters above sea level, with an annual rainfall of 1123 mm and temperatures ranging from 13 to 27°C. Lake Hawassa is the primary water source for the residents of Hawella Tulla, catering to their domestic, agricultural, fishing, and other needs. According to ECSA, the district has a total population of 129,507, comprising 65,018 males and 64,499 females. The district has various

social service institutions, including 27 health facilities and 69 schools. The health facilities consist of 1 primary hospital, 6 health centers, 18 rural health posts, 2 urban health posts, and 1 non-governmental health center. In terms of education, the district includes 21 primary schools, 5 secondary schools, 6 non-governmental primary schools, and 37 non-governmental kindergarten schools (Fig 1).

Fig 1: Map of the study site. The figure in the left corner depicts Lake Hawassa and the study district, while the upper right corner shows Ethiopia. The figure at the bottom right displays the map of the Sidama region 2024 (The map is prepared using ArcGIS 10.8 (ESRI, Redlands, CA USA) after obtaining country shape files from the humdata.org website (http://data.humdata.org/).

Population and enrollment criteria

The source population was all schoolchildren in the Tulla district, Sidama region, southern Ethiopia. The study population included schoolchildren who were enrolled in the selected schools during the study period and met the established inclusion criteria (who had both parental or guardian informed consent and their assent to participate) and exclusion criteria (who had received anti-helminthic treatment within one month before the recruitment period, or who were unable to provide stool and urine samples were excluded from the study).

Sample size and sampling technique

To determine the sample size, a single population proportion formula was used based on the following assumptions: a prevalence of *S. mansoni* of 31% [20], with a 5% margin of error and a 95% confidence level. As a result, the calculated sample size was 329. Considering the design or cluster effect between schools, the determined number was multiplied by 1.5, and then 5% was added for the non-response rate, giving the final sample size of 484.

To recruit participants for this study, we utilized a purposive sampling technique. In the initial stage, Bushulo and Finchawa, located near the coasts of Lake Hawassa in the Tulla sub-city, were selected alongside two primary schools farther from the lake, Gemeto and Tulla, with student populations of 1,820, 686, 2,050, and 1,439, respectively. Then, the predetermined sample size of 484 was proportionally allocated to the schools, resulting in 147 participants from

Bushulo, 55 from Finchawa, 116 from Gemeto, and 166 from Tulla. The students' registration lists served as the sampling frame, and a systematic random sampling technique was employed to select the study subjects from each school. The k-value (sampling interval) was determined by dividing the total number of study participants in each school by the number of students to be selected. The students were then systematically selected at regular intervals (every 12th student), stratified by age (5–19 years) and grade levels (1–4 and 5–8) until the predetermined sample size was achieved. In cases where the selected student was absent from school, three repeated visits were made. When a student missed after three visits, he/she considered non-respondents.

Dependent and independent variables

The dependent variables were prevalence, infection intensity, and diagnostic performance of *S. mansoni* infection. The independent variables included socio-demographic characteristics such as gender, age, and educational status of both parents, as well as family occupation. Additionally, grade level and the schools attended were taken into account. The distance from home to the lake and practice-related factors were also examined, emphasizing habits such as swimming, fishing, wearing shoes, washing clothes, hand washing before meals and after using the latrine, open-field defecation, trimming fingernails, bathing, and involvement in irrigation activities. Environmental factors considered encompassed the availability of a latrine at home. Clinical symptoms of the disease were also evaluated among study participants.

Data collection methods

Interview

The data was collected using a structured questionnaire. Two trained nurses conducted home-to-home visits to interview the parents or guardians of the children, utilizing the KOBO Collect digital tool. A senior expert and the principal investigator supervised the data collection process, and regular meetings were held to address any challenges and ensure adherence to established protocols and standards.

Stool and Urine sample collection

Fecal and urine samples were collected in a separate screw-capped, dry, and clean container pre-labelled with the participant's unique identification number. The stool samples were transported within an hour of collection in suitable cool boxes at temperatures between 4°C and 6°C for subsequent examination. Samples from Gemeto and Tulla Elementary Schools were transported to Tulla Hospital, while samples from Bushulo and Finchawa Elementary Schools were sent to the Bushulo Mother and Child Health Specialty Center. The investigator supervised the collection and transportation of samples to ensure compliance with standard operating procedures.

Stool sample processing using the Kato-Katz technique

The stool samples were examined using the KK technique to detect parasite eggs. A portion of the sample was processed using a calibrated template holding 41.7 mg of stool. Double slides were prepared for each stool sample. The number of eggs was counted from the double KK microscopic slides, and the average count was converted to eggs per gram of feces (EPG) using a correction factor of 24. Based on WHO cut-off values, the infection intensity was categorized as light (EPG 1-99), moderate (EPG 100-399), or heavy (EPG >400) [27].

Urine sample processing using POC-CCA rapid test

Each urine sample was processed immediately at the schools using the POC-CCA test, following the manufacturer's instructions [28]. This on-site processing enabled prompt results and minimized potential delays or degradation of the samples. 100μL of urine was transferred to the circular well of the test cassette and allowed to absorb completely into the specimen pad. The test results were read after 20 minutes of assay development. In cases of discrepancies, the tests were re-examined to ensure accuracy. The POC-CCA tests were scored as negative or positive based on the formation and intensity of the control band. Tests were considered invalid if an internal control band did not appear or if results were not read after 25 minutes or more. In such cases, samples were re-run with a new cassette and scored as necessary.

Data quality assurance

The questionnaire was initially developed in English and then translated into Amharic and Sidamu Afoo, the local language of the participants. It was then translated back into English to ensure consistency. A pre-test was conducted on 5% of the sample size at Tullo Primary School, which was not part of the selected schools for the final study. The questionnaire was subsequently modified based on the findings from the pre-test. The completeness of data collection was overseen daily by the assigned supervisor.

The senior laboratory technologist provided refresher training to laboratory professionals. Standard operating procedures (SOPs) for pre-analytical, analytical, and post-analytical procedures were implemented throughout sample analysis. To ensure internal quality control, 10% of the slides were randomly selected and re-examined by a senior laboratory technologist at Tulla Hospital and Bushulo Mother and Child Health Specialty Centre. Additionally, another experienced technologist at HUCSH conducted an external laboratory evaluation. Both stool and urine samples were collected daily between 09:00-12:00 AM and 03:00-05:00 AM local time throughout the survey.

Data analysis

Data were entered into the KOBO Collect digital tool and analyzed using IBM SPSS Statistics Version 26. Descriptive statistics, including frequency, percentage, mean, and standard deviation were calculated. Arithmetic mean infection intensity of *S. mansoni* was also amalyzed and reported as low, moderate, or heavy according to WHO guidelines[4]. The mean egg count was reported as a geometric mean. A logistic regression model was used to identify potential determinants of *S. mansoni* and STH infections. Initially, a bivariate analysis was conducted to calculate the crude odds ratios (COR) for each variable and to determine which variables were significantly associated with the outcome. To control for confounding variables, factors with a p-value of ≤ 0.25 from the bivariate analysis were included in the multivariable logistic regression

analysis, allowing for the calculation of adjusted odds ratios (AOR). A p-value of less than 0.05 was considered statistically significant. The diagnostic performance of the POC-CCA test was assessed using metrics like sensitivity and specificity, often using the KK as a reference standard [29]. The KK method is the most commonly used diagnostic approach for helminths, epidemiology studies, and treatment evaluation which shows good performance, particularly at detecting helminth infections of moderate and heavy intensity.

Kappa statistic was employed to quantify the agreement between the two diagnostic methods, and the concordance rate was determined as the proportion of cases that yielded identical results. While the McNemar chi-square test was employed to compare the statistically significant differences in sensitivity and specificity between the two diagnostic methods, focusing on cases where the tests disagree.

Ethical consideration

Ethical clearance was obtained from Hawassa University, College of Medicine, and the other Health Sciences Internal Review Board (IRB) with a reference number: IRB/172/16 in 2024. A letter of support from Hawassa University School of Medical Laboratory was provided to the Sidama Regional State Health Bureau, as well as to the health and education offices in Tulla district and the directors of the selected schools. Participation was fully voluntary, with written informed consent obtained from the parents/guardians of the children enrolled. An additional separate written assent was secured from children older than 12 years. Explicit written assent was not obtained for children under 12 years, as they may lack the developmental capacity to understand this concept. However, these children were verbally informed about the study in simple language, and their willingness or unwillingness to participate was respected.

Schoolchildren who tested positive for any pathogenic intestinal parasites received appropriate treatment administered by qualified public health professionals. All data collected during the study were kept strictly confidential and used exclusively for research purposes. The study

procedures were conducted in full compliance with relevant ethical guidelines and regulatory standards

Result

Socio-demographic characteristics of the study participants

A total of 484 students were invited to participate in the study, of whom 477 met the inclusion criteria and agreed to take part, resulting in a response rate of 98.5%. The study sample comprised 208 males (43.6%) and 269 females (56.4%). Participants' ages ranged from 5 to 19 years, with a mean age of 11.94 years and a standard deviation of 2.76 years. Most of the study participants (62.7%) were 10 - 14 years old. The majority of students reside within 1 km of a lake (66.5%) and are under the care of their private (daily laborer) parents or guardians (41.7%). The students were selected from 4 primary schools: Bushulo 143 (30%), Finchawa 53 (11.1%), Gemeto 116 (24.3%), and Tulla 165 (34.6%) **(Table 1).**

Prevalence of *Schistosoma mansoni* infection

The prevalence of *S. mansoni* among school children, as determined by the two combined diagnostic methods, was found to be 36.5%. Specifically, 20.1% (96 out of 477 samples) were detected using the KK technique, while 34.4% (164 out of 477 samples) were identified through the POC-CCA test. Compared to sex for *S. mansoni*, boys were more infected than girls (25.0% *vs*. 16.4%). Children aged 10 – 14 years were more infected (22.4%) than those aged 5– 9 years (9.1%). There was a significant variation in the prevalence of *S. mansoni* across different schools (Table 1). The highest prevalence of *S. mansoni* infection was observed among students attending Bushulo Elementary School (36.4%), followed by Finchawa (24.5%), Gemeto (14.6%), and Tulla (8.5%). Children living within 1 km of the lake had a higher *S. mansoni* infection rate (39.6%) compared to those residing more than 1 km away, who had an infection rate of 14.8%. Additionally, children who had greater contact with the contaminated lake exhibited a higher prevalence of *S. mansoni* infection compared to those who had no exposure. Specifically, the infection rate among children who engaged in fishing was 35.1%, notably higher than the 18.1% infection rate among those who did not participate in fishing activities. Table 1 shows detailed information about various activities at the lake and their associated infection rates.

Potential associated risk factors of *S. mansoni* infections.

In the bivariate logistic regression analysis, several activities were identified as potential risk factors for *S. mansoni* infection. These included washing clothes (13%, 62/477), bathing (31.7%, 151/477), swimming (32.9%, 157/477), fishing (11.9%, 57/477), and engaging in irrigation activities (24.9%, 119/ 477), all with a p-value of ≤ 0.25. Additionally, socio-demographic factors such as gender, age, the parents' educational status, attending Bushulo School and the distance of the residential area from the lake were also identified as potential predictors of *S. mansoni* infection (Table 1). Multivariate logistic regression analysis was applied to avoid confounding factors and only students engaged in irrigation were 2.4 times more likely to be infected than those not involved (AOR = 2.43; 95% CI: 1.38-4.29, P = 0.002), schoolchildren with swimming habits exhibited a 2.4-fold increase in the likelihood of infection (AOR = 2.41; 95% CI: 1.43-4.34, P = 0.001) and participants who bathed in the lake were 1.8 times more likely to be infected than those who did not engage in this activity (AOR = 1.80; 95% CI: 1.05-3.12, P = 0.033). A school-based analysis revealed that students attending Bushulo primary school were 4.7 times more likely to contract *S. mansoni* infection compared to their peers at Tulla primary school (AOR = 4.68; 95% CI: 1.87-11.74, P = 0.001) (Table 1).

326 Table 1: The distribution of *Schistosoma mansoni* infection by socio-demographic and other factors among schoolchildren in Tulla
327 district, Sidama region, Southern Ethiopia 2024 (n = 477).

Variables	*S. mansoni* Positive %	Negative %	Total %	COR (95% CI)	p-value	AOR (95%CI)	p-value
**Gender**							
Male	52(25.0)	156(75.0)	208(43.6)	1		1	
Female	44(16.4)	225(83.6)	269(56.4)	0.587(0.374-0.920)	0.020	0.607(0.359-1.027)	0.063
Age							
5-9	7(9.1)	70(90.9)	77(16.1)	1			
10-14	67(22.4)	232(77.6)	299(62.7)	2.888(1.268-6.578)	0.012	2.118(0.841-5.330)	0.111
15-19	22(21.8)	79(78.2)	101(21.2)	2.785(1.122-6.914)	0.027	1.442(0.495-4.198)	0.502
Name of School							
Bushulo	52(36.4)	91(63.6)	143(30)	6.163(3.234-11.746)	0.001	4.685(1.869-11.742)	**0.001****
Finchawa	13(24.5)	40(75.5)	53(11.1)	3.505(1.526-8.051)	0.003	1.444(0.486-4.297)	0.509
Gemeto	17(14.6)	99(85.4)	116(24.3)	1.852(0.874-3.926)	0.108	1.528(0.662-3.525)	0.320
Tulla	14(8.5)	151(91.5)	165(34.6)	1			
Educational status of the father							
No formal education	17(18.5)	75(81.5)	92(19.3)	1		1	
Primary school	58(26.0)	165(74.0)	223(46.75)	1.551(0.846-2.841)	0.156	1.420(0.690-2.921)	0.341
Educational status of the mother							
No formal education	3(7.0)	40(93.0)	43(9)	1		1	
Primary school	62(23.2)	205(76.8)	267(56)	4.033(1.206-13.485)	0.024	3.391(0.889-12.937)	0.074
Secondary school	25(18.8)	108(81.2)	133(28)	3.086(0.883-10.787)	0.078	2.178(0.535-8.859)	0.277
College and above	6(17.7)	28(82.3)	34(7)	2.857(0.658-12.397)	0.161	2.384(0.414-13.719)	0.331
Distance from residence area							
Within 1km	49(30.6)	111(69.4)	160(33.5)	1		1	
More than 1km	47(14.8)	270(85.2)	317(66.5)	0.394(0.250-0.623)	0.001	1.194(0.615-2.318)	0.601
Bathing in the lake							
Yes	43(28.5)	108(71.5)	151(31.7)	2.051(1.295-3.248)	0.002	1.804(1.048-3.107)	**0.033***
No	53(16.3)	273(83.7)	326(68.3)	1		1	
Swimming habit							
Yes	50(31.9)	107(68,1	157(32.9)	2.783(1.780-4.403)	0.001	2.407(1.430-4.052)	**0.001****

No	46(14.4)	274(85.6)	320(67.1)	1			
Fishing							
Yes	20(35.1)	37(64.9)	57(12)	2.447(1.345-4.449)	0.003	1.135(0.533-2.417)	0.743
No	76(18.1)	344(71.9)	420(88)	1			
Washing clothes along the lake shore							
Yes	19(30.6)	43(69.4)	62(13)	1.940(1.071-3.513)	0.029	2.016(0.934-4.350)	0.074
No	77(17.3)	338(82.7)	415(87)	1			
Involvement in irrigation							
Yes	44(37.0)	75(63.0)	119(25)	3.452(2.148-5.547)	0.001	2.429(1.377-4.287)	**0.002***
No	52(14.5)	306(85.5)	358(75)	1			

AOR, Adjusted Odds Ratio; CI, Confidence interval; COR, Crude Odds Ratio; *, Significant at $p < 0.05$.

Infection intensity of *Schistosoma mansoni* infection

Out of 174 children infected with *S. mansoni*, 96 tested positive by the KK technique using a standard volume of stool that enables the quantification of ova per gram of stool. The minimum and maximum eggs per gram of stool were 24 and 672, respectively. Likewise, the arithmetic and geometric mean values were 189.75 and 139.39, respectively. According to the WHO classification, among 96 students infected with *S. mansoni*, 33 (34.4%) had light intensity, 54 (56.2%) had moderate intensity, and 9 (9.4%) had heavy intensity (Fig. 2).

Fig 2: The prevalence of *S. mansoni* infection intensity across 477 schoolchildren in Tulla district, Sidama Region, southern Ethiopia, 2024

There was no significant variation in the intensity of infection across age groups. However, the intensity of infection across age groups varies, with the younger age groups, 10 – 14 years, having less moderate intensity (49.25%) compared to older age groups, 15 – 19 years (72.7%) and the younger age group, 5 – 9 years (71.4%). The highest light intensity of *S. mansoni* infection (64.3%) and the lowest moderate intensity (35.7%) were observed among students at Tulla Elementary School, with no cases of heavy intensity when compared to students at other elementary schools **(Table 2)**

Table 2: Gender, age, and school-based variation in *S. mansoni* infection intensity among schoolchildren in Tulla District Sidama Region southern Ethiopia 2024 (n = 477).

**Variable**	**N**	**Intensity of infection n (%)**		
		**Light**	**Moderate**	**Heavy**
**Gender**				
Male	52	18(34.6)	28(53.9)	6(11.5)
Female	44	15(34.1)	26(59.1)	3(6.8)
**Age**				
5-9	7	2(28.6)	5(71.4)	0(0)
10-14	67	27(40.3)	33(49.25)	7(10.45)
15-19	22	4(18.2)	16(72.7)	2(9.1)
**Schoolchildren**				
Bushulo	52	14(27)	32(61.5)	6(11.5)
Finchawa	13	4(31)	7(53.8)	2(15.2)
Gemeto	17	6(35.3)	10(58.8)	1(5.9)
Tulla	14	9(64.3)	5(35.7)	0(0)

Key - N: refers to the total number of infected; whereas, n: refers to the number of infected according to intensity classes.

Prevalence of soil transmitted helminthic infections

Aside from *S. mansoni*, the prevalence of intestinal helminths among the schoolchildren was 233 (48.8%). The most common STHs were *A. lumbricoides* 162 (34%), followed by *T. trichuria* 39 (8.2%) and hookworm 27 (5.7%). There were also intestinal helminth parasites reported in this study including *Taenia* species 6 (1.3%), *Hymenolepis nana* (*H.nana*) 3 (0.6%), and *Enterobius vermicularis* (*E. vermicularis*) 1 (0.2%) **(Fig. 3).** The younger age group of 5–9 years was more infected (49.4%) than those aged 15–19 years (43.6%). The data revealed that children whose parents had no formal education were more likely to be infected with STH (59.4%) compared to those whose parents had a college education (27.3%). Additionally, higher STH infection rates were found among children from families where the parents worked as daily labourers (54.5%) or farmers (50.8%). Children who did not wash their hands after using the latrine exhibited a significantly higher STH infection rate of 52.8%, while those who practised hand washing had a much lower rate of 12.8%. Similarly, children who neglected to wash their hands before meals had an infection rate of 53.4%, compared to 33.6% for those who did wash their hands before eating. Furthermore, children who did not trim their nails had an increased risk of contracting STH infections, with an infection rate of 54.7% compared to 32.7% for those who regularly trimmed their nails, as shown in (**Table 3)**.

Fig 3: The prevalence of intestinal helminthic infections across 477 school children in Tulla district, Sidama Region, southern Ethiopia, 2024

Potential associated risk factors of STH infections

In the bivariate logistic regression analysis, both hygiene-related practices and socio-demographic factors were identified as potential risk factors for soil-transmitted helminthic infections. STH infection was associated with latrine use, hand washing practices, fingernail trimming habits, educational level, and occupation, with a p-value of ≤ 0.25 **(Table 3).**

Finally, the multivariate regression analysis revealed that latrine use, school location, educational status, nail trimming practices, and hand washing after using the latrine were significantly associated with STH infection, with a p-value of ≤ 0.05 as summarised in Table 3. Students from Tula Elementary School had 0.47 times lower odds of STH infection compared to their counterparts at Bushulo Elementary School (AOR = 0.469; 95% CI: 0.256-0.861, P = 0.015). Specifically, children whose fathers had no formal education were found to be 4.2 times more likely to be infected than those whose fathers had completed college or higher education (Adjusted Odds Ratio = 4.161; 95% Confidence Interval: 1.276-13.570, P = 0.018).

The presence of latrines at home also significantly influenced infection rates, with children having access to latrines exhibiting 0.63 times lower odds of infection than those without such facilities (AOR = 0.626; 95% CI: 0.393-0.996, P = 0.048). Furthermore, hygiene practices emerged as critical determinants, as children who washed their hands after using the latrine had a remarkable 0.2 times lower odds of contracting STH infections compared to those who did not (AOR = 0.199; 95% CI: 0.072-0.551, P = 0.002). Similarly, regular nail trimming was associated with a significant reduction in infection risk, with children who trimmed their nails having 0.4 times lower odds of STH infection than those who did not (AOR = 0.413; 95% CI: 0.250-0.682, P = 0.001) (Table 3).

Table 3: Univariate and multivariate logistic regression analyses for socio-demographic and other potential risk factors associated with soil transmitted helminthic infections among school children in Tulla district, Sidama region, Southern Ethiopia 2024 (n = 477).

Variables	STH Positive %	Negative %	Total %	COR (95% CI)	p-value	AOR (95%CI)	p-value
Gender							
Male	97(46.6)	111(53.4)	208(43.6)	1			
Female	136(50.6)	133(49.4)	269(56.4)	1.170(0.814-1.681)	0.395		
Age							
5-9	38(49.4)	39(50.6)	77(16.1)	1			
10-14	151(50.5)	148(49.5)	299(62.7)	1.047(0.634-1.728)	0.857		
15-19	44(43.6)	57(56.4)	101(21.2)	0.792(0.437-1.437)	0.443		
School							
Bushulo	76(53.2)	67(46.8)	143(30)	1		1	
Finchawa	28(52.8)	25(47.2)	53(11.1)	0.987(0.525-1.857)	0.969		
Gemeto	54(46.5)	62(53.5)	116(24.3)	0.768(0.470-1.254)	0.292		
Tulla	75(45.5)	90(54.5)	165(34.6)	0.735(0.469-1.151)	0.178	0.469(0.256-0.861)	**0.015***
Grade level							
1-4	118(48.2)	127(51.8)	245(51.4)	1		1	
5-8	126(54.3)	106(45.7)	232(48.6)	1.279(0.893-1.834)	0.180	0.848(0.495-1.453)	0.548
Educational status of father							
No formal education	55(59.8)	37(40.2)	92(19.3)	3.964(1.420-11.066)	0.009	4.161(1.276-13.570)	**0.018***
Primary school	115(51.6)	108(48.4)	223(46.7)	2.840(1.072-7.523)	0.036	2.957(0.991-8.818)	0.052
Secondary education	57(40.7)	83(59.3)	140(29.4)	1.831(1.072-7.523)	0.234	1.616(0.547-4.775)	0.385
College and above	6(27.3)	16(72.7)	22(4.6)	1		1	
Occupation of family							
Civil servant	6(31.6)	13(68.4)	19(4)	1		1	
Merchant	56(41.5)	79(58.5)	135(28.3)	1.536(0.550-4.285)	0.412		
Farmer	63(50.8)	61(49.2)	124(26)	2.238(0.799-6.264)	0.125	0.199(0.013-3.028)	0.245
Private (Daily laborer)	108(54.3)	91(45.7)	199(41.7)	2.571(0.940-7.037)	0.066	0.262(0.017-3.932)	0.332
Latrine availability at home							
Yes	128(42)	177(58)	305(63.9)	1		1	
No	105(61)	67(39)	172(36.1)	0.461(0.315-0.676)	0.001	0.626(0.393-0.996)	**0.048***

Hand washing after latrine							
Yes	6(12.8)	41(87.2)	47(9.9)	1		1	
No	227(52.8)	203(47.2)	430(90.1)	0.131(0.54-0.315)	0.001	0.199(0.072-0.551)	**0.002***
Shoe wearing							
Yes	198(49.25	204(50.75)	402(84.3)	1			
No	35(46.7)	40(53.3)	75(15.7)	0.902(0.550-1.477)	0.681		
Finger nail trimming							
Yes	39(29.3)	94(70.7)	133(27.9)	1		1	
No	194(56.4)	150(43.6)	344(72.1)	0.321(0.209-0.493)	0.001	0.413(0.250-0.682)	**0.001***
Hand washing before meal							
Yes	37(33.6)	73(66.4)	110(23)	1		1	
No	196(53.4)	171(46.6)	367(77)	0.442(0.283-0.890)	0.001	0.585(0.337-1.015)	0.058
Open field defecation							
Yes	24(48)	26(52)	50(10.5)	1.039(0.578-1.867)	0.899		
No	209(48.9)	218(51.1)	427(89.5)	1			

Key - AOR: Adjusted Odds Ratio, CI: Confidence interval, COR: Crude Odds Ratio, *: Significant at p < 0.05, STH: Soil-transmitted helminths.

Detection and performance evaluation of the POC-CCA test

Out of 477 samples examined, the POC-CCA rapid test identified 86 (18.0%) true positives, 303 (63.5%) true negatives, and 10 (2.1%) discrepancies (false negatives) compared to the KK technique. Using KK as the gold standard or 'reference test' [29], the POC-CCA sensitivity was 89.6% (95% CI: 82.3 - 94.4), specificity 79.5% (95% CI: 75.2-83.3), NPV 96.8% (95% CI: 94.1–98.2), PPV 52.4% (95% CI: 44.6 – 60.1), Negative LR 0.13 (95% CI: 0.07–0.23) and Positive LR 4.3 (95% CI: 3.38–5.63) **(Table 4)**

Table 4: Performance of POC-CCA test using Kato-Katz as a gold standard across 477 school children in Tulla district Sidama Region souther Ethiopia 2024

**Method**	**Result**	**KK (Gold standard)**		**Sensitivity % (95% CI)**	**Specificity % (95% CI)**	**NPV % (95% CI)**	**PPV % (95 CI)**	**LR^- % (95% CI)**	**LR^+ % (95% CI)**
		**Pos**	**Neg**						
**POC-CC**	**Pos**	**86**	**78**	**89.6 (82.3-94.4**	**79.5 (75.2-83.3)**	**96.8 (94.1-98.2**	**52.4 (44.6-60.1)**	**0.13 (0.07-0.23)**	**4.3 (3.38-5.63**
	**Neg**	**10**	**303**						

Key - KK: Kato-Katz POC-CCA: Point of Care Circulating Cathodic Antigen, NPV: Negative Predictive value, PPV: Positive Predictive value, NLR$^-$: Negative Likely hood ratio, LR$^+$: Positive Likely hood ratio, Pos: Positive, Neg: Negative.

The agreement between the two diagnostic methods was quantified using Cohen's Kappa statistics which yielded a kappa value of 0.55. Furthermore, the overall concordance rate between the KK and POC-CCA was 81.5%. However, to assess the statistical significance of the differences in results between the two tests, we employed the McNemar Chi-square test. The result revealed a value of 52.3

Discussion

Although large-scale deworming programmes targeting specific groups have been successful in reducing morbidity associated with *S. mansoni* and STH infections, the absence of a sensitive diagnostic method for detecting low infection intensities and reinfection without additional intervention measures remains unavoidable. This study assessed the prevalence and related risk factors of these parasites, and evaluated the intensity and diagnostic performance of the POC-CCA rapid test for *S. mansoni* among schoolchildren in the Tulla district of the Sidama region, Ethiopia.

Prevalence of *Schistosoma mansoni* infection

The prevalence of *S. mansoni* infection was 20.1% (95% CI: 16.8-23.7) using the KK technique. This finding highlights the need for annual deworming of schoolchildren in the study area, as the prevalence falls within the moderate range (≥ 10% but < 50%) according to WHO guidelines [4]. This result is consistent with previous studies conducted in the region, which reported prevalence rates as follows: Jimma. 20.2% [30], Kersa, 19.9% [31], Mekelle, 23.9% [32], Egypt, 19.1% [33], and Angola, 21.2% [34]. However, it is higher than the prevalence observed in Jiga, 15.2% [35], Guangua, 12.6% [36], and Wondo districts, 11.4% [37]. In contrast, the prevalence found in this study is lower than that reported in Lake Tana, 34.9% [38], Jimma town, 28.7% [39], Maksegnit, Debark, Sanja, and Chuahit districts, 33.5% [40], Abbey and Didessa valleys 53.9% [41], and Wolaita, 81.3% [42], Kenya, 53.7% [43], Congo, 90.2% [44], and Tanzania, 90.6% [45]. The variations in the prevalence of *S. mansoni* infection across different regions may be attributed to differences in temperature and rainfall patterns that influence snail reproduction and water availability, which in turn affect the local transmission rates of the parasite. Differences in

human behavior towards contaminated water sources include swimming or crossing infested water, as well as performing domestic chores in contaminated water bodies. Inadequate access to safe drinking water and the lack of proper sanitation facilities, leading to open defecation near water sources, influence the transmission cycle of the parasite.

Socioeconomic factors such as increased population density and migration to new localities contribute to the spread of schistosomiasis by increasing the potential for exposure to infested water. Altered agricultural practices and the use of untreated water for irrigation can enhance opportunities for human-water contact and snail proliferation that may also favour the rate of infection. Environmental factors may also impact disease transmission variation across different regions, including the proximity to snail habitats, the construction of dams, land cover (particularly elevation), rainfall patterns, seasonal land surface temperatures, and the presence of flooded agricultural areas. Additionally, it is crucial to consider differences in sample sizes, study designs and diagnostic methods applied.

Potential associated risk factors of *S. mansoni* infections.

A significant association was found between *S. mansoni* infection and schoolchildren who engaged in swimming and bathing activities in the lake, with infection risks being 2.4 times higher for swimming and 1.8 times higher for bathing than those who did not partake in these habits. This association between *S. mansoni* infection and swimming habits among schoolchildren aligns with previous studies [20], [36] and is further supported by studies linking bathing habits to infection [38]. Additionally, a strong association was noted between *S. mansoni* infection and participation in irrigation activities, with students involved in irrigation being 2.4 times more likely to be infected than those not engaged in such activities, consistent with prior findings [20], [36]. Notable differences in infection rates were observed among students across

the various schools in the study areas. Children attending Bushulo Primary School were 4.7 times more likely to be infected with Schistosoma than their peers at Tulla Primary School. This finding aligns with previous studies [20], [38]. This might be associated with students' residence distance variation from lakes and other water bodies.

Intensity of *S. mansoni* infection

In the present study, out of 174 participants infected with *S. mansoni*, 96 were confirmed positive using the KK technique, a standard diagnostic method valued for its ability to quantify EPG of stool. The intensity of infection ranged from 24 to 672 EPG, with an arithmetic mean of 189.75 and a geometric mean of 139.39. The noticeable discrepancy between the arithmetic and geometric means reflects the presence of a right-skewed distribution of egg counts. This pattern is consistent with the typical epidemiology of schistosomiasis, where a minority of individuals harbor high worm burdens, while the majority exhibit low to moderate infection levels [18].

The moderate infection intensity reported in this study is consistent with findings from various regions in Ethiopia [41], [46] and in Tanzania [45]. This difference might be due to repeated exposure of schoolchildren to water bodies infested with the infective stage. The intensity of *S. mansoni* infection was found to be slightly higher in male students compared to female students, which is consistent with findings from Kemissie and Wondo Genet [46] and Jimma Town [47] in Ethiopia. The observed difference in proportion might be linked to male students' greater exposure to outdoor activities like fishing, swimming, and irrigation compared to female students.

Prevalence of soil transmitted helminthic infections

The study area displayed a moderate prevalence of STH infections among schoolchildren despite them being part of the deworming target group. Deworming programs have successfully decreased the global morbidity attributed to STHs [48], but the risk of re-infection persists in the absence of improved water, sanitation, and hygiene WASH practices and health education [49]. A systematic review and meta-analysis estimated that three months after deworming, the prevalence of *A. lumbricoides*, *T. trichuria*, and *hookworms* reached 26%, 36%, and 30% of their pre-treatment levels, respectively. By one-year post-treatment, the prevalence rates approached their pre-treatment levels again, with *A. lumbricoides* at 94%, *T. trichuria* at 82%, and *hookworm* infections at 57% [50]. The occurrence of re-infection highlights the importance of a comprehensive approach to controlling STH. This is especially crucial given the increasing interest in ultimately halting STH transmission in specific geographic regions [51]. The current WHO guidelines recommend the evaluation of any STH infections in school-age children after five to six years of periodic chemotherapy using the standard Kato-Katz thick smear [52].

Potential associated risk factors of STH infections

The results of this study also provide critical insights into the socio-demographic and environmental determinants associated with STH infections among school children. This research identified several significant factors linked to STH infection, including the lack of a latrine at home, inadequate hand-washing practices following latrine use, the educational status of fathers, improper fingernail trimming practices, and certain school attendance patterns. The lack of a latrine at home was identified as a significant risk factor for STH infections in this study. This finding emphasises the strong association between poor sanitation and the spread of

helminths. It agrees with extensive research showing that limited access to sanitation facilities, especially at the household level, significantly contributes to STH infections [53], [54], [55]. Without access to latrines, many individuals are forced to practice open defecation, resulting in the direct release of helminth eggs into the environment and promoting continued transmission. Having access to household latrines significantly reduces the risk of exposure to infectious helminth stages in soil and water by minimizing the dispersion of feces in the surrounding area. Notably, This finding aligns with previous research in the same region, which also identified open field defecation as a key factor contributing to STH infection [20]. This study strengthens existing evidence by focusing on sanitation conditions at the household level rather than using broader community-level indicators. This approach provides a deeper understanding of how individual risks are affected by domestic infrastructure, emphasizing the need for targeted public health interventions that go beyond general sanitation promotion. It is essential to prioritize equal access to latrines and ensure their sustained use at the household level, especially in high-transmission areas where environmental contamination continues to pose a significant challenge.

Hand hygiene practices, especially the failure to wash hands after using the latrine, were significantly associated with the prevalence of STH, which aligns with prior global studies [56], [57], [58]. This highlights the importance of hand washing as a key factor in preventing the transmission of STH [59]. In contrast to previous research in the same area, hand hygiene showed no significant effect [20]. This divergence might be due to variations in study design, changes in hygiene behaviors over time, or previous underestimations of individual practices. Furthermore, recent improvements in sanitation infrastructure, in the absence of corresponding behavioral changes, could increase the relative impact of hand hygiene on the risk of infection. These findings indicate the complex interaction between environmental and behavioral factors

that influence the transmission of STH and emphasize the importance of ongoing local assessments.

Moreover, the educational status was found to be significantly associated with STH prevalence among children, which aligns with previous studies [60], [61]. This highlights the importance of targeting educational programs not only for children but also for parents to foster a supportive environment for health-promoting behaviors. Certain school attendance was also significantly associated with STH infection, in line with previous studies [62], [63]. This might be due to differences in sanitation facilities, hygiene practices, socio-economic status, and health education among students, which can significantly influence the transmission dynamics of STH infections in certain schools.

This study also revealed that the practice of not trimming fingernails was significantly associated with STH infection, which aligns with previous studies [54], [64], [65], [66]. Since long fingernails can harbor soil and pathogens, serving as a reservoir for helminth eggs that can be ingested or transmitted through hand-to-mouth contact, public health initiatives need to promote personal hygiene practices among school-aged children, including regular nail-trimming and cleanliness.

Comparison of the POC-CCA test performance with KK on *S. mansoni*

The current study found a better diagnostic performance of the POC-CCA assay using a single urine sample than duplicate KK thick smears from a single stool sample. From the 96 KK positive samples, the POC-CCA test identified 10 (10.4%) as negative. Conversely, with KK negative results, POC-CCA classified only 78 (4.9%) as positive, indicating that the KK technique has higher specificity.

In this study, using the KK technique as the gold standard, the POC-CCA test demonstrated higher sensitivity but notably lower specificity. The improved sensitivity (particularly in detecting low-intensity infections) might be attributed to the POC-CCA test's ability to detect trace levels of circulating antigens in urine, allowing for broader detection of *Schistosoma* infection, including cases that KK might miss due to the known variability in egg excretion and the limited sensitivity of KK, especially in low-endemic settings [67]. However, the specificity of the POC-CCA test was found to be 79.6%, which is consistent with previously reported ranges but remains relatively low [25]. This reduced specificity may be attributed to cross-reactivity with other helminth infections, the subjective interpretation of trace results as positive, and the continued excretion of CCA following recent treatment or in the absence of active infection [68]. Such limitations can result in false positives, particularly in low-endemic or post-treatment settings, potentially leading to an overestimation of prevalence. Despite its limitations, the POC-CCA test is a valuable diagnostic tool in field settings because of its ease of use, rapid results, and high sensitivity. However, its limited specificity highlights the necessity for confirmatory testing, especially in situations involving surveillance, program evaluation, and low-transmission areas where diagnostic accuracy is crucial.

The overall agreement between KK and POC-CCA was moderate, as reflected by a kappa statistic of 0.55 ($p < 0.001$) and an observed agreement rate of 81.5%. While this level of concordance suggests a reasonable overlap between the two diagnostic tools, it must be interpreted cautiously given the limitations of κ statistics, which are sensitive to disease prevalence and imbalanced marginal totals [69]. McNemar's test revealed a statistically significant discordance between the two diagnostic methods, with a chi-square value of 52.3 and $p < 0.001$. This discrepancy was primarily driven by the high number of cases that were positive

by the point-of-care circulating cathodic antigen (POC-CCA) test but negative by the KK method. This lack of alignment underscores the fundamentally different targets of the two diagnostic tools: the POC-CCA test detects active infections by identifying CCA in urine, while the KK method relies on detecting *Schistosoma* eggs in stool, which can vary from day to day and show inconsistencies within the same sample [22]. Therefore, in field settings, especially in low-endemic or post-treatment areas, the POC-CCA test may identify infections that the KK method misses, making it a more sensitive tool for surveillance and monitoring of control programs.

Conclusions

The existence of moderate prevalence of *S. mansoni* and STH infections among school children emphasises the need of annual deworming for school-aged children in the study area. The findings from this study also provide valuable insights into socio-demographic factors, behavioural factors, personal hygiene practices and environmental sanitation association to STH and Schistosoma infections in these children. This highlights the need for additional control measures to supplement deworming programmes, including health education focused on personal hygiene and environmental sanitation. Furthermore, this study shows that KK has low sensitivity and may underestimate the accurate prevalence. In contrast, the POC-CCA test demonstrates higher sensitivity in the study area, making it a more reliable option for case detection and large-scale screening. Therefore, we recommend adopting the POC-CCA test in school-based *S. mansoni* infection control and elimination programmes alongside the KK method, as this could enhance diagnostic accuracy and improve management outcomes. However, to optimise the consistency and utility of this test, further research to standardise the scoring system is strongly recommended.

Limitations

This study has a few limitations that should be acknowledged. (i), the cross-sectional design provides a snapshot of *S. mansoni* and STH infection at a single point in time, which may not capture seasonal variations or fluctuations in infection rates over longer periods. (ii), the use of a single stool sample per participant for the KK technique may have led to underestimation of infection prevalence and intensity, given the known day-to-day variability in helminth egg excretion. (iii), the purposive selection of schools limits the generalizability of the findings to the larger community in the study area.

Acknowledgement

Primarily, we would like to thank Hawassa University, School of Medical Laboratory Sciences, for funding this research. Then our thanks go to the study participants and the school community for their cooperation, as their contributions were crucial to its success. Finally, we would also like to thank Tulla Primary Hospital and Bushulo Mother and Child Health Specialty Center staff, especially laboratory professionals, for their dedication and hard work in collecting and conducting experiments.

References

1 World Health Organization. Schistosomiasis and soil-transmitted helminthiases: progress report, 2023. Wkly Epidemiol Rec. 2024;99:707-17.

2 Exum NG, Kibira SP, Ssenyonga R, Nobili J, Shannon AK, Ssempebwa JC et al. The prevalence of schistosomiasis in Uganda: A nationally representative population estimate to inform control programs and water and sanitation interventions. PLoS neglected

tropical diseases. 2019 Aug 14;13(8):e0007617.

3 Hussen S, Assegu D, Tadesse BT, Shimelis T. Prevalence of Schistosoma mansoni infection in Ethiopia: a systematic review and meta-analysis. Tropical diseases, travel medicine and vaccines. 2021 Feb 1;7(1):4.

4 World Health Organization. WHO guideline on control and elimination of human schistosomiasis. World Health Organization; 2022 Feb 14. https://www.who.int/publications/i/item/9789240041608

5 Ismail HA, Hong ST, Babiker AT, Hassan RM, Sulaiman MA, Jeong HG et al. Prevalence, risk factors, and clinical manifestations of schistosomiasis among school children in the White Nile River basin, Sudan. Parasites & vectors. 2014 Oct 15;7(1):478.

6 Lo NC, Bezerra FS, Colley DG, Fleming FM, Homeida M, Kabatereine N, Kabole FM, King CH, Mafe MA, Midzi N, Mutapi F. Review of 2022 WHO guidelines on the control and elimination of schistosomiasis. The Lancet Infectious Diseases. 2022 Nov 1;22(11):e327-35.

7 Dahal AS, Francis EO, Francis JE, Wamtas FI. Soil-transmitted helminths and associated risk factors among elementary school pupils in Dadin Kowa, Jos. Nigerian Medical Journal. 2019 Jul 1;60(4):181-5. doi: 10.4103/nmj.nmj_62_19.

8 Getaneh M, Hailegebriel T, Munshea A, Nibret E. Prevalence and Associated Risk Factors of Soil-Transmitted Helminth Infections Among Schoolchildren Around Lake Tana, Northwest Ethiopia. Journal of Parasitology Research. 2022;2022(1):4603638.

9 Wale M, Gedefaw S. Prevalence of intestinal Protozoa and soil transmitted helminths infections among school children in Jaragedo town, South Gondar zone of Ethiopia. Journal of Tropical Medicine. 2022;2022(1):5747978.

10 Beyene B, Vaz Nery S, Lambiyo T, Shimelis T. Schistosoma mansoni and soil-transmitted helminth infections among schoolchildren living along the shore of Lake Hawassa, southern Ethiopia. Parasites & Vectors. 2024 Dec 3;17(1):498.

11 Pullan RL, Smith JL, Jasrasaria R, Brooker SJ. Global numbers of infection and disease burden of soil transmitted helminth infections in 2010. Parasites & vectors. 2014 Jan 21;7(1):37, doi: 10.1186/1756-3305-7-37.

12 Östan İ, Kilimcioğlu AA, Girginkardeşler N, Özyurt BC, Limoncu ME, Ok ÜZ. Health inequities: lower socio-economic conditions and higher incidences of intestinal parasites. BMC public health. 2007 Nov 27;7(1):342 , doi: 10.1186/1471-2458-7-342.

13 N. R. De Silva, S. Brooker, P. J. Hotez, A. Montresor, D. Engels, and L. Savioli,. Soil-transmitted helminth infections: Updating the global picture. *Trends Parasitol.*, vol. 19, no. 12, pp. 547–551, 2003, doi: 10.1016/j.pt.2003.10.002.

14 World Health Organization. Schistosomiasis and soil-transmitted helminthiases: progress report, 2021. Wkly Epidemiol Rec. 2022;97(48):621-32.

15 Rayment Gomez S, Maddren R, Liyew EF, Chernet M, Anjulo U, Tamiru A et al. Predisposition to soil-transmitted helminth reinfection after four rounds of mass drug administration: results from a longitudinal cohort in the Geshiyaro project, a transmission elimination feasibility study in the Wolaita zone of southern Ethiopia. Transactions of The Royal Society of Tropical Medicine and Hygiene. 2023 Jul;117(7):514-21.

16 Negussu N, Mengistu B, Kebede B, Deribe K, Ejigu E, Tadesse G, Mekete K, Sileshi M. Ethiopia schistosomiasis and soil-transmitted helminthes control programme: progress and prospects. Ethiopian medical journal. 2017;55(Suppl 1):75.

17 Leta GT, Mekete K, Wuletaw Y, Gebretsadik A, Sime H, Mekasha S, Woyessa A, Shafi O,

Vercruysse J, Grimes JE, Gardiner I. National mapping of soil-transmitted helminth and schistosome infections in Ethiopia. Parasites & vectors. 2020 Sep 1;13(1):437.

18 Maddren R, Phillips A, Ower A, Landeryou T, Mengistu B, Anjulo U, Firdawek E, Negussu N, Anderson R. Soil-transmitted helminths and schistosome infections in Ethiopia: a systematic review of progress in their control over the past 20 years. Parasites & vectors. 2021 Feb 5;14(1):97.

19 Terefe A, Shimelis T, Mengistu M, Hailu A, Erko B. Schistosomiasis mansoni and soil-transmitted helminthiasis in Bushulo village, southern Ethiopia. Ethiopian Journal of Health Development. 2011;25(1):46-50.

20 Tadege B, Shimelis T. Infections with Schistosoma mansoni and geohelminths among school children dwelling along the shore of the Lake Hawassa, southern Ethiopia. PloS one. 2017 Jul 18;12(7):e0181547.

21 Sousa MS, Van Dam GJ, Pinheiro MC, De Dood CJ, Peralta JM, Peralta RH, et al.. Performance of an ultra-sensitive assay targeting the circulating anodic antigen (CAA) for detection of Schistosoma mansoni infection in a low endemic area in Brazil. Frontiers in immunology. 2019 Apr 4;10:682.

22 Lindholz CG, Favero V, Verissimo CD, Candido RR, de Souza RP, Dos Santos RR, et al. Study of diagnostic accuracy of Helmintex, Kato-Katz, and POC-CCA methods for diagnosing intestinal schistosomiasis in Candeal, a low intensity transmission area in northeastern Brazil. PLoS Neglected Tropical Diseases. 2018 Mar 8;12(3):e0006274.

23 Mueller A, Fuss A, Ziegler U, Kaatano GM, Mazigo HD. Intestinal schistosomiasis of Ijinga Island, north-western Tanzania: prevalence, intensity of infection, hepatosplenic morbidities and their associated factors. BMC infectious diseases. 2019 Oct 7;19(1):832.

24 Haggag AA, Rabiee A, Abd Elaziz KM, Campbell Jr CH, Colley DG, Ramzy RM. Thirty-day daily comparisons of Kato–Katz and CCA assays of 45 Egyptian children in areas with very low prevalence of Schistosoma mansoni. The American journal of tropical medicine and hygiene. 2019 Jan 2;100(3):578.

25 Colley DG, Binder S, Campbell C, King CH, Tchuenté LA, N'Goran EK et al. A five-country evaluation of a point-of-care circulating cathodic antigen urine assay for the prevalence of Schistosoma mansoni. The American journal of tropical medicine and hygiene. 2013 Mar 6;88(3):426.

26 Straily A, Kavere EA, Wanja D, Wiegand RE, Montgomery SP, Mwaki A, et al. Evaluation of the point-of-care circulating cathodic antigen assay for monitoring mass drug administration in a Schistosoma mansoni control program in Western Kenya. The American Journal of Tropical Medicine and Hygiene. 2021 Nov 8;106(1):303.

27 Bugssa G, Teklehaymanot T, Medhin G, Berhe N. Prevalence and intensity of Schistosoma mansoni infection, and contributing factors in Alamata district of Tigray Region, Northern Ethiopia. PLOS Neglected Tropical Diseases. 2024 Nov 25;18(11):e0012691.

28 Casacuberta-Partal M, Hoekstra PT, Kornelis D, van Lieshout L, van Dam GJ. An innovative and user-friendly scoring system for standardised quantitative interpretation of the urine-based point-of-care strip test (POC-CCA) for the diagnosis of intestinal schistosomiasis: a proof-of-concept study. Acta Tropica. 2019 Nov 1;199:105150.

29 Okoyo C, Simiyu E, Njenga SM, Mwandawiro C. Comparing the performance of circulating cathodic antigen and Kato-Katz techniques in evaluating Schistosoma mansoni

infection in areas with low prevalence in selected counties of Kenya: a cross-sectional study. BMC Public Health. 2018 Apr 11;18(1):478.

30 Tiruneh A, Zemene E, Abdissa Mizana B, Girma H, Dereje E, Sharew B et al. Schistosoma mansoni infections and morbidities among School Children in Hotspot areas of Jimma Town, Southwest Ethiopia: a cross-sectional study. Environmental Health Insights. 2023 Mar;17:11786302231161047.

31 Aliyi H, Ahmed M, Gobena T, Alemu BM, Adem HA, Usso AA. Prevalence and factors associated with Schistosoma mansoni infection among primary school children in Kersa District, Eastern Ethiopia. PeerJ. 2024 Jun 14;12:e17439.

32 Teklemariam A, Dejenie T, Tomass Z. Infection prevalence of intestinal helminths and associated risk factors among schoolchildren in selected kebeles of Enderta district, Tigray, Northern Ethiopia. J Parasitol Vector Biol. 2014;6(11):166-73.

[33] Ghazy RM, Ellakany WI, Badr MM, Taktak NE, Elhadad H, Abdo SM et al. Determinants of Schistosoma mansoni transmission in hotspots at the late stage of elimination in Egypt. Infectious Diseases of Poverty. 2022 Sep 23;11(1):102.

34 Bartlett AW, Sousa-Figueiredo JC, van Goor RC, Monaghan P, Lancaster W, Mugizi R, et al. Burden and factors associated with schistosomiasis and soil-transmitted helminth infections among school-age children in Huambo, Uige and Zaire provinces, Angola. Infectious Diseases of Poverty. 2022 Jun 25;11(1):73.

35 Wubet K, Damtie D. Prevalence of Schistosoma mansoni Infection and Associated Risk Factors among School Children in Jiga Town, Northwest-Ethiopia: A Cross-Sectional Study. Journal of Parasitology Research. 2020;2020(1):6903912.

36 Tazebew B, Temesgen D, Alehegn M, Salew D, Tarekegn M. Prevalence of S. mansoni

infection and associated risk factors among school children in Guangua District, Northwest Ethiopia. Journal of Parasitology Research. 2022;2022(1):1005637.

37 Ansha MG, Kuti KA, Girma E. Prevalence of intestinal schistosomiasis and associated factors among school children in Wondo District, Ethiopia. Journal of tropical medicine. 2020;2020(1):9813743.

38 Hailegebriel T, Nibret E, Munshea A, Ameha Z. Prevalence, intensity and associated risk factors of Schistosoma mansoni infections among schoolchildren around Lake Tana, northwestern Ethiopia. PLoS Neglected Tropical Diseases. 2021 Oct 27;15(10):e0009861.

39 Tefera A, Belay T, Bajiro M. Epidemiology of Schistosoma mansoni infection and associated risk factors among school children attending primary schools nearby rivers in Jimma town, an urban setting, Southwest Ethiopia. PloS one. 2020 Feb 27;15(2):e0228007.

40 Zeleke AJ, Addisu A, Tegegne Y. Prevalence, intensity, and associated factors of Schistosoma mansoni among school children in Northwest Ethiopia. Journal of Parasitology Research. 2020;2020(1):8820222

41 Assefa A, Erko B, Gundersen SG, Medhin G, Berhe N. Current status of Schistosoma mansoni infection among previously treated rural communities in the Abbey and Didessa Valleys, Western Ethiopia: Implications for sustainable control. Plos one. 2021 Feb 25;16(2):e0247312.

42 Alemayehu B, Tomass Z. Schistosoma mansoni infection prevalence and associated risk factors among schoolchildren in Demba Girara, Damot Woide District of Wolaita Zone, Southern Ethiopia. Asian Pacific journal of tropical medicine. 2015 Jun 1;8(6):457-63.

43 Masaku J, Madigu N, Okoyo C, Njenga SM. Current status of Schistosoma mansoni and

the factors associated with infection two years following mass drug administration programme among primary school children in Mwea irrigation scheme: A cross-sectional study. BMC Public Health. 2015 Aug 1;15(1):739.

44 Nigo MM, Odermatt P, Salieb–Beugelaar GB, Morozov O, Battegay M, Hunziker PR. Epidemiology of Schistosoma mansoni infection in Ituri Province, north-eastern Democratic Republic of the Congo. PLoS Neglected Tropical Diseases. 2021 Dec 2;15(12):e0009486.

45 Mnkugwe RH, Minzi OS, Kinung'hi SM, Kamuhabwa AA, Aklillu E. Prevalence and correlates of intestinal schistosomiasis infection among school-aged children in North-Western Tanzania. PloS one. 2020 Feb 5;15(2):e0228770.

46 Aemero M, Berhe N, Erko B. Status of Schistosoma mansoni prevalence and intensity of infection in geographically apart endemic localities of Ethiopia: a comparison. Ethiopian journal of health sciences. 2014 Jul 31;24(3):189-94.

47 Bajiro M, Dana D, Levecke B. Prevalence and intensity of Schistosoma mansoni infections among schoolchildren attending primary schools in an urban setting in Southwest, Ethiopia. BMC research notes. 2017 Dec 4;10(1):677.

48 Montresor A, Mupfasoni D, Mikhailov A, Mwinzi P, Lucianez A, Jamsheed M, et al. The global progress of soil-transmitted helminthiases control in 2020 and World Health Organization targets for 2030. PLoS neglected tropical diseases. 2020 Aug 10;14(8):e0008505.

49 Gass K, Addiss DG, Freeman MC. Exploring the relationship between access to water, sanitation and hygiene and soil-transmitted helminth infection: a demonstration of two recursive partitioning tools. PLoS neglected tropical diseases. 2014 Jun 12;8(6):e2945.

50 Jia TW, Melville S, Utzinger J, King CH, Zhou XN. Soil-transmitted helminth reinfection after drug treatment: a systematic review and meta-analysis. PLoS neglected tropical diseases. 2012 May 8;6(5):e1621.

51 Brooker SJ, Mwandawiro CS, Halliday KE, Njenga SM, Mcharo C, Gichuki PM, Wasunna B, Kihara JH, Njomo D, Alusala D, Chiguzo A. Interrupting transmission of soil-transmitted helminths: a study protocol for cluster randomised trials evaluating alternative treatment strategies and delivery systems in Kenya. BMJ open. 2015 Oct 1;5(10):e008950.

52 Giardina F, Coffeng LE, Farrell SH, Vegvari C, Werkman M, Truscott JE et al. Sampling strategies for monitoring and evaluation of morbidity targets for soil-transmitted helminths. PLoS neglected tropical diseases. 2019 Jun 26;13(6):e0007514.

53 Fenta A, Mesganaw B, Belachew F, Teshale G, Abebaw G, Tesfa E, Alemayehu G. Prevalence and risks of soil transmitted helminths among Ethiopian school children: A cross-sectional study. Asian Pacific Journal of Tropical Medicine. 2022 Mar 1;15(3):121-7.

54 Samuel F, Demsew A, Alem Y, Hailesilassie Y. Soil transmitted Helminthiasis and associated risk factors among elementary school children in ambo town, western Ethiopia. BMC Public Health. 2017 Oct 10;17(1):791.

55 Tolera B, Mohammed J, Umer U, Abamecha J, Tebeje F, Sime A, Weldegebreal F. Prevalence, intensity, and associated factors of intestinal schistosomiasis among primary school children in Nono District, Southwest Ethiopia. Frontiers in Tropical Diseases. 2025 Feb 18;5:1483164.

56 Alemu G, Abossie A, Yohannes Z. Current status of intestinal parasitic infections and

associated factors among primary school children in Birbir town, Southern Ethiopia. BMC infectious diseases. 2019 Mar 19;19(1):270.

57 Zeynudin A, Degefa T, Suleman S, Abamecha A, Hajikelil Z, Wieser A. Prevalence and Determinants of Geohelminthiasis among School-Age Children in Jimma City, Ethiopia. Journal of Tropical Medicine. 2023;2023(1):8811795.

58 Bokicho B, Hailu D, Eshetu B, Matie M, Tadele T. Soil-transmitted helminthiases among school-age children and their association with water, sanitation, and hygiene, Hawassa City, Southern Ethiopia. PLoS Neglected Tropical Diseases. 2023 Jul 28;17(7):e0011484.

59 Luby SP, Agboatwalla M, Painter J, Altaf A, Billhimer WL, Hoekstra RM. Effect of intensive handwashing promotion on childhood diarrhea in high-risk communities in Pakistan: a randomized controlled trial. Jama. 2004 Jun 2;291(21):2547-54.

60 Feleke DG, Ali A, Bisetegn H, Debash H, Birara W, Andualem A. Soil-transmitted helminthes and Schistosoma mansoni infections among primary school children at Ambasame primary school, North-West Ethiopia: a cross-sectional study. BMC pediatrics. 2022 Aug 5;22(1):477.

61 Forson AO, Arthur I, Ayeh-Kumi PF. The role of family size, employment and education of parents in the prevalence of intestinal parasitic infections in school children in Accra. PloS one. 2018 Feb 7;13(2):e0192303.

62 Kihoro RW, Mulewa D, Okoyo C, Ayaa D, Korir A, Njomo DW, Mwandawiro C, Masaku J. Epidemiology of soil-transmitted helminthiasis among school-aged children in pastoralist communities of Kenya: A cross-sectional study. Plos one. 2024 May 23;19(5):e0304266.

63 Debalke S, Worku A, Jahur N, Mekonnen Z. Soil transmitted helminths and associated

factors among schoolchildren in government and private primary school in Jimma Town, Southwest Ethiopia. Ethiopian journal of health sciences. 2013 Nov 11;23(3):237-44.

64 Genet A, Motbainor A, Samuel T, Azage M. Prevalence and associated factors of soil transmitted helminthiasis among school-age children in wetland and non-wetland areas of Blue Nile Basins, northwest Ethiopia: A community-based comparative study. SAGE Open Medicine. 2021 Dec;9:20503121211063354.

65 Andargie Y, Alelign A, Tekeste Z. Prevalence and associated risk factors of soil-transmitted helminth infections among schoolchildren in Mekan Eyesus town, Northwestern Ethiopia. Parasite Epidemiology and Control. 2024 Nov 1;27:e00379.

66 Novianty S, Dimyati Y, Pasaribu S, Pasaribu AP. Risk Factors for Soil-Transmitted Helminthiasis in Preschool Children Living in Farmland, North Sumatera, Indonesia. Journal of tropical medicine. 2018;2018(1):6706413.

67 Graeff-Teixeira C, Favero V, Pascoal VF, de Souza RP, de Vargas Rigo F, Agnese LH, et al. Low specificity of point-of-care circulating cathodic antigen (POCCCA) diagnostic test in a non-endemic area for schistosomiasis mansoni in Brazil. Acta tropica. 2021 May 1;217:105863.

68 Homsana A, Odermatt P, Southisavath P, Yajima A, Sayasone S. Cross-reaction of POC-CCA urine test for detection of Schistosoma mekongi in Lao PDR: a cross-sectional study. Infectious diseases of poverty. 2020 Aug 1;9(04):66-74.

69 Casagrande A, Fabris F, Girometti R. Beyond kappa: an informational index for diagnostic agreement in dichotomous and multivalue ordered-categorical ratings. Medical & Biological Engineering & Computing. 2020 Dec;58(12):3089-99.02261-2.

Abbreviation

AOR, adjusted odds ratio; CCA, circulating cathodic antigen; CI, Confidence interval; COR, Crude odds ratio; EPG, Eggs per gram; KK, Kato Katz; LR^+, Positive likelihood ratio; LR^-, N likelihood ratio; MDA, Mass drug administration; NPV, Negative predictive value; NTD, neglected tropical disease; POC, Point of care; PPV, Positive predictive value; SOP, Standard operating procedure; STH, Soil-transmitted Helminth; WHO, World Health Organization, WASH, Water Sanitation and Hygiene, PC, preventive chemotherapy

Availability of data and materials

All relevant data are within the manuscript, and the datasets generated and/or analyzed during the current study are submitted. Restrictions apply to the availability of these data, which parents had consented for the collected information to be used for our research study only, and so are not publicly available.

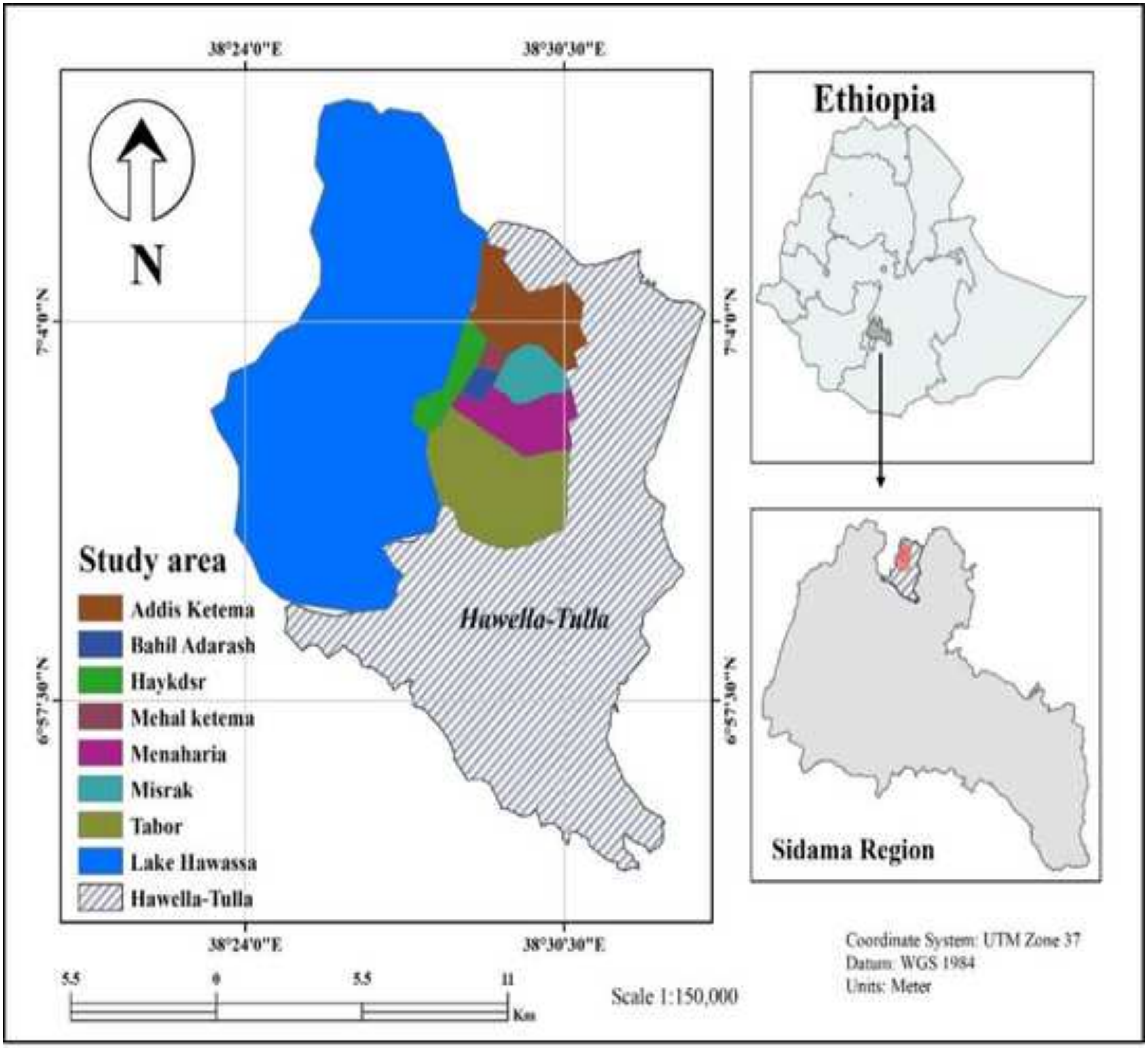

Fig 1: The figure in the left corner depicts Lake Hawassa and the study district, while the upper right corner shows Ethiopia and the bottom right shows the Sidama Region, 2024. The map is prepared using ArcGIS 10.8 (ESRI, Redlands, CA, USA) after obtaining country shape files from the humdata.org website (http://data.humdata.org/).

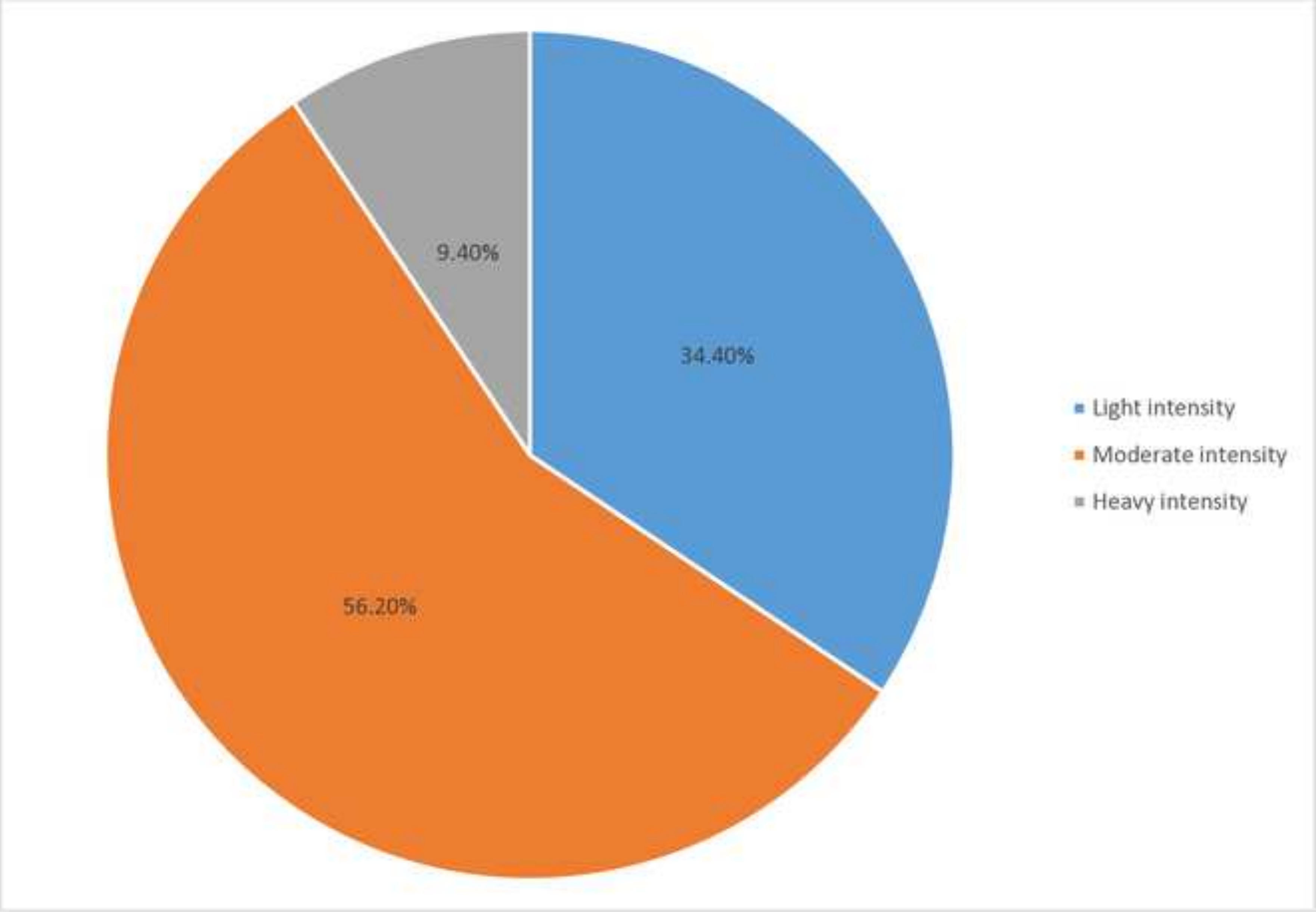

Fig 2: The prevalence of *S. mansoni* infection intensity in stool across 477 school children in Tulla district, Sidama Region, southern Ethiopia, 2024

 Click here to access/download;Figure;Fig 3.tif

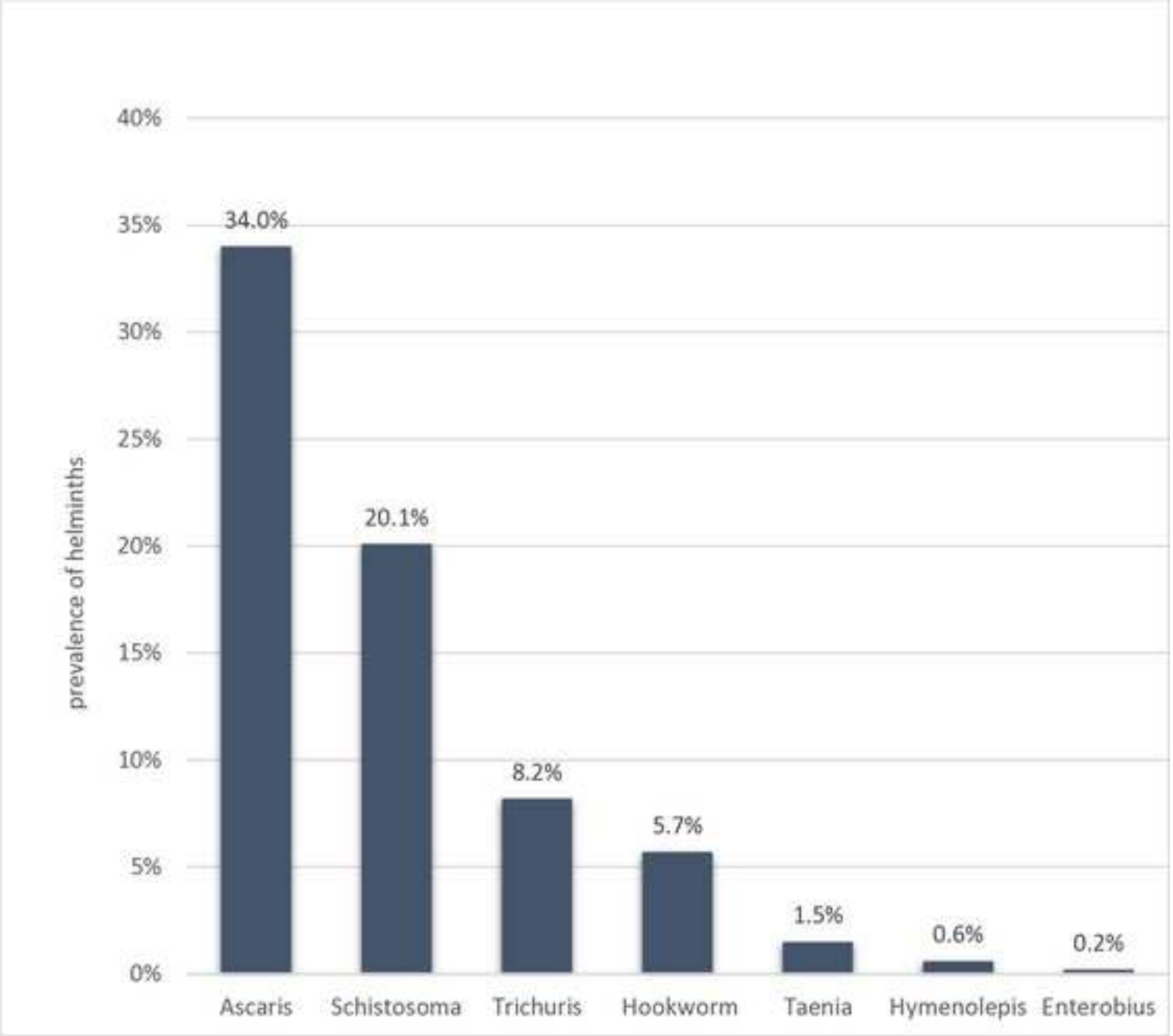

Figure 3: The prevalence of intestinal helminthic infection in stool across 477 school children in Tulla district, Sidama Region, southern Ethiopia, 2024

Click here to access/download
Supporting Information
SPSS raw data.sav

Click here to access/download
Supporting Information
S1 Table questionnaire.docx

Assessment of *Schistosoma mansoni* and Soil-transmitted Helminth Infections and the Diagnostic Performance of the Circulating Cathodic Antigen Test among Schoolchildren in Tulla District, Sidama Region, Southern Ethiopia.

Short title: *Schistosoma mansoni* Infection and Performance of its antigen test

Fitsum Getachew[1,2*], Demissie Assegu[1], Biniyam Kijineh[3] and Bamlaku Tadege[1*]

[1] School of Medical Laboratory Sciences, Hawassa University, Hawassa, Ethiopia.

[2] School of Medical Laboratory Sciences, Wolaita Sodo University, Wolaita, Ethiopia.

[3] School of Medical Laboratory Sciences, Wachemo University, Ethiopia, Hossana, Ethiopia.

*E-mail of corresponding author: bamlaku22tadege@gmail.com

Consent for publication

All authors have read the manuscript and consented to submit the manuscript.

Competing interests

All authors declare that they have no competing interests.

Authors' contributions

FG: conceptualization, data curation, formal analysis, investigation, methodology, writing – original draft; DA & BK: writing – review & editing, BT: conceptualization, supervision, writing – review & editing, data curation, and formal analysis.

Authors' addresses

fitsumgetachew6@gmail.com (FG)

bamlaku22tadege@gmail.com **(**BT**)**

demisissieasegu@yahoo.com (DA)

kijinehb@gmail.com (BK)

Funding

This study was funded by Hawassa University

Abstract

Background: STH and *S. mansoni* pose significant public health challenges in areas with poor sanitation. Research on *S. mansoni* intensity remains limited in study area. *S. mansoni* diagnosis traditionally relies on Kato-Katz method, though POC-CCA urine test offers a rapid alternative with high sensitivity. This study aimed to assess prevalence and risk factors of *S. mansoni* and STH infections. It also evaluated intensity of *S. mansoni* and compared performance of POC-CCA test to that of KK.

~~Soil-transmitted helminths (STH) and *Schistosoma mansoni* pose significant public health challenges in areas with poor sanitation~~. ~~While *S. mansoni* infection leads to chronic illness and mortality, research on its intensity remains limited in the study area. Diagnosis traditionally relies on the Kato-Katz method, though the POC-CCA urine test offers a rapid alternative. This study examined the prevalence, intensity, and risk factors of *S. mansoni* and Soil-transmitted helminth infections among schoolchildren while evaluating the diagnostic performance of the POC-CCA test.~~

Methods: A cross-sectional study was conducted among schoolchildren in Tulla district, Sidama region, from April to June 2024, using purposive sampling. Data were collected through pre-structured questionnaires, and stool samples were analyzed using KK method, while urine samples were analyzed with POC-CCA technique. A logistic regression model was employed to examine potential associations between infections and risk factors, the Kappa statistic assessed agreement between tests, and the McNemar chi-square test compared the sensitivity and specificity of the diagnostic methods.

~~A school-based cross-sectional study was conducted in the Tulla district, Sidama region, among 477 students (April–June 2024). The Schoolchildren were recruited using a purposive sampling~~

~~technique. Socio-demographic data were collected using pre-structured questionnaires. A single stool sample was collected from each study participant and processed for the detection of helminth eggs using the Kato-Katz (KK) technique. Additionally, urine samples were tested with the Point of Care Circulating Cathodic Antigen Test (POC-CCA), which detects Schistosoma antigens.~~

Result: The prevalence of *S. mansoni* with two combined tests was 36.5% (20.1% by KK and 34.4% by POC-CCA). Activities like irrigation, swimming and bathing showed significant associations with *S. mansoni* infection. The prevalence of STH was 48.8%, with A. lumbricoides (34%) as the most common, followed by *T. trichuria* (8.2%), hookworm (5.7%), *Taenia* species (1.3%), *H. nana* (0.6%) and *E.vermicularis* (0.2%). Factors like hand washing, fingernail trimming, lack of latrines, and educational status were significantly linked to STH infections. The POC-CCA test demonstrated higher sensitivity (89.6%) than KK technique (McNemar test χ^2m = 52.3, $p < 0.001$).~~The overall prevalence of *Schistosoma mansoni* was 36.5% (20.1% by KK and 34.4% by POC-CCA). The prevalence of STH was 48.8%. The leading parasite was *Ascaris lumbricoides* (34%), followed by *Trichuris trichuria* (8.2%) and hookworm (5.7%). Other helminths included Taenia species 1.3%, *Hymenolepis nana* 0.6%, and *Enterobius vermicularis* 0.2%. Infection intensity of *S. mansoni* was classified as light 34.4%, moderate 56.2%, and heavy 9.4%. Involvement in irrigation, swimming, bathing, and school location were significantly associated with *S. mansoni* infection. School location, educational status, lack of latrine, hand washing practice and fingernail trimming practice were significantly associated with STH infections. The POC-CCA rapid test had a significantly higher sensitivity of 89.6% (McNemar test χ^2m = 52.3, $p < 0.001$) compared to the Kato-Katz technique.~~

Formatted: English (United Kingdom)

Conclusions: The moderate prevalence of *S. mansoni* and STH infections, coupled with socio-demographic factors, behaviours, hygiene practices, and sanitation issues associated with these infections, highlights the need for additional control measures beyond deworming. Implementing a highly sensitive POC- CCA test alongside KK method in low-endemic areas could improve diagnostic accuracy and enhance disease management outcomes.~~Moderate *S. mansoni* and STH infection and poor hygiene practice underscore for additional control measures. Incorporating POC-CCA testing alongside the KK method could optimize diagnostic accuracy and improve disease management outcomes.~~

Formatted: English (United Kingdom)

Keywords: ~~Prevalence, Infection intensity,~~ Prevalence, intensity, risk factor, *Schistosoma mansoni,* Soil-transmitted helminth, ~~Diagnostic performance,~~ Kato-Katz technique, Point-of-care circulating cathodic antigen, Tulla district.

Introduction

~~Background~~

Formatted: Heading 1

Schistosoma and soil-transmitted helminth (STH) are worm infections that belong to the group of neglected tropical diseases (NTDs) [1]. Schistosomiasis is a water-based, vector-borne parasite transmitted indirectly through freshwater snails [2]. People who live in areas with inadequate water and sanitation systems are susceptible to this most common and deadly infection [3]. Globally, schistosomiasis is recognized as endemic in 78 low and middle-income countries in tropical and subtropical areas, estimated to affect >251 million people [4]. Furthermore, 243 million people require preventative chemotherapy, including 111 million school-age children, of which 226 million are in Africa [5]. It causes significant morbidity, with an estimated 1.4-3.3 million disability-adjusted life years (DALYs) lost annually due to its health impacts [6].

STHs are intestinal parasitic worms transmitted to humans through fecally contaminated soil [7]. The main STHs that affect humans are *Ascaris lumbricoides* (A. *lumbricoides*), *Trichuris trichuria (*T. *trichuria), Necator americanus, and Ancylostoma duodenale* [8]. These parasites cause gastrointestinal infections and are most commonly found in tropical and subtropical regions, particularly impacting impoverished nations where schoolchildren are especially vulnerable [9].

Globally, an estimated 1.5 billion people are infected with STHs, including 654 million school-aged children living in areas where these parasites are prevalent [10]. The Burden of *Schistosoma* and STHs leads to various health issues, including gastrointestinal bleeding, nutrient malabsorption, loss of appetite, anemia due to the depletion of iron and other essential proteins, growth retardation, impaired cognitive development, school absenteeism, and disability-adjusted life-years lost [11], [12], [13].

The World Health Organization (WHO) recommends integrated strategies for controlling and eliminating schistosomiasis and STH. These strategies include preventive chemotherapy (PC), which consists of periodic administration of anthelmintic medicines—praziquantel for schistosomiasis and albendazole or mebendazole for STH. Additionally, improvements in water,

sanitation, and hygiene (WASH), behavior change, snail control, and environmental management are essential, as deworming alone cannot prevent re-infection [14], [15].

In Ethiopia, an estimated 79 million people live in areas endemic for STHs, and 37.3 million live in regions affected by *S. mansoni*, with the majority being school-aged children—specifically, 25.3 million and 12.3 million children, respectively [16~~14~~]. National mapping surveys conducted between 2013 and 2015 across Ethiopia's regional states revealed a prevalence of 21.7% for STHs and 3.5% for *S. mansoni* infections among school-aged children [17~~5~~]. In 2015, Ethiopia launched a national control program aimed at eliminating *S. mansoni* and STHs as serious public health concerns by 2020, ~~to break~~with the goal of breaking transmission by 2025 [18~~6~~]. The program aims to treat at least 75% of school-aged children and to extend treatment to adolescents and adults. It also seeks to integrate initiatives for neglected tropical diseases and strengthen collaboration with mass drug administration efforts. Furthermore, it aims to enhance cooperation with WASH programs ~~The program's objectives include treating at least 75% of school-aged children, extending treatment to adolescents and adults, integrating neglected tropical disease initiatives, and strengthening collaboration between mass drug administration and WASH initiatives~~ [164]. However, the WASH program has limited coverage, particularly in rural areas. This issue is primarily attributed to insufficient collaboration and the high financial costs associated with running the program in Ethiopia.

Results from systematic reviews in Ethiopia have shown that helminth control interventions have significantly reduced the prevalence of STH and *S. mansoni* infections over the years in Ethiopia [3], [18~~6~~]. Additionally, a survey conducted among schoolchildren in our study area revealed a notable decline in the prevalence of STHs (52.4%) and *S. mansoni* (31%) in 2015 compared to the rates in 2007, which were 67.3% for STHs and 76.5% for *S. mansoni* [19~~7~~], [20~~18~~]. There is an annual deworming programme for school children in our study area to control STH and *S. mansoni*. However, the current status of infection intensity in the study area, following the increase in helminth control interventions, remains unknown.

~~To control *Schistosoma* and STH-attributable morbidity, WHO recommends three interventions, including periodical administration of anthelmintic drugs to kill the worms, health education, and improved water, hygiene, and sanitation (WASH) to prevent worm re-infection. Today, mass drug administration (MDA) programs remain the mainstream strategy. During these programs, a single oral dose of albendazole (400 mg) or mebendazole (500 mg) for STH and praziquantel for *Schistosoma* are periodically administered to the at-risk groups [19]. Although these deworming programs have been successful in reducing global *Schistosoma* and STH-attributable morbidity, re-infection perpetuates in the absence of improved WASH and health education [20].~~

Intestinal *Schistosoma* is diagnosed by microscopic detection of parasite eggs in stool using the Kato-Katz (KK) technique, which is widely used in epidemiological surveys [21]. However, its diagnosis is challenging in low-endemic areas where prevalence and worm burden are reduced, due to factors like uneven egg distribution in stool samples, daily variations in egg excretion, and random distribution effects [22], [23]. ~~On the other hand,~~T~~t~~he point-of-care (POC) cassette test is a relatively new diagnostic tool that detects circulating cathodic antigens (CCA) in urine samples, offering higher sensitivity and operational benefits [23], [24]. The past study found that POC-CCA was more sensitive (86% vs. 62%) but less specific (72% vs. ~100%) compared to multiple KK smears from a single stool sample. ~~Despite this, the study noted that only 1% of POC-CCA tests in non-endemic areas were false positives, indicating that the specificity of POC-CCA might be underestimated.~~ Multivariable modeling also suggested that POC-CCA was significantly more sensitive than KK for detecting low infection intensities (<100 eggs/gram of stool) [25]. However, before the POC-CCA assay can be fully utilized for monitoring and evaluating control programs, it is essential to assess its performance in endemic settings [26].

Therefore, this study aimed to assess the prevalence and risk factors of *S. mansoni* and STH infections among schoolchildren. It also aimed to assess the intensity of *S. mansoni* and compare the diagnostic performance of the POC-CCA test with KK.

~~Therefore, this study aimed to evaluate the prevalence of *Schistosoma mansoni* (*S. mansoni*) and STH infections and intensity of *S. mansoni* in elementary school-aged children, as well as the associated risk factors to evaluate ongoing control intervention. Additionally, it sought to assess the performance of the POC-CCA test compared to the KK method for diagnosing *S. mansoni*.~~

Materials and Methods

Study design and setting

A school-based cross-sectional study was conducted between April and June 2024 among four governmental elementary school children in the Tulla district. The study was conducted in four elementary schools in the Tulla district, Sidama region in Southern Ethiopia. The district is situated along the shore of Lake Hawassa and the Tikur Wuha River, approximately 289 kilometers south of Addis Ababa, the capital city of Ethiopia. The altitude is 1800 meters above sea level, with an annual rainfall of 1123 mm and temperatures ranging from 13 to 27°C. Lake Hawassa is the primary water source for the residents of Hawella Tulla, catering to their domestic, agricultural, fishing, and other needs. According to ECSA, the district has a total population of 129,507, comprising 65,018 males and 64,499 females. The district has various social service institutions, including 27 health facilities and 69 schools. The health facilities consist of 1 primary hospital, 6 health centers, 18 rural health posts, 2 urban health posts, and 1 non-governmental health center. In terms of education, the district includes 21 primary schools, 5 secondary schools, 6 non-governmental primary schools, and 37 non-governmental kindergarten schools (Fig 1).

Fig 1: Map of the study site. The figure in the left corner depicts Lake Hawassa and the study district, while the upper right corner shows Ethiopia. The figure at the bottom right displays the map of the Sidama region 2024 (The map is prepared using ArcGIS 10.8 (ESRI, Redlands, CA USA) after obtaining country shape files from the humdata.org website (http://data.humdata.org/).

Population and enrollment criteria

The source population was all schoolchildren in the Tulla district, Sidama region, southern Ethiopia. The study population included schoolchildren who were enrolled in the selected schools during the study period and met the established inclusion criteria (who had both parental or guardian informed consent and their assent to participate) and exclusion criteria (who had received anti-helminthic treatment within one month before the recruitment period, or who were unable to provide stool and urine samples were excluded from the study).

Sample size and sampling technique

To determine the sample size, a single population proportion formula was used based on the following assumptions: a prevalence of *S. mansoni* of 31% [18], with a 5% margin of error and a 95% confidence level. As a result, the calculated sample size was 329. Considering the design or cluster effect between schools, the determined number was multiplied by 1.5, and then 5% was added for the non-response rate, giving the final sample size of 484.

To recruit participants for this study, we utilized a purposive sampling technique. In the initial stage, Bushulo and Finchawa, located near the coasts of Lake Hawassa in the Tulla sub-city, were selected alongside two primary schools farther from the lake, Gemeto and Tulla, with student populations of 1,820, 686, 2,050, and 1,439, respectively. Then, the predetermined sample size of 484 was proportionally allocated to the schools, resulting in 147 participants from Bushulo, 55 from Finchawa, 116 from Gemeto, and 166 from Tulla. The students' registration lists served as the sampling frame, and a systematic random sampling technique was employed to select the study subjects from each school. The k-value (sampling interval) was determined by dividing the total number of study participants in each school by the number of students to be selected. The students were then systematically selected at regular intervals (every 12th student), stratified by age (5–19 years) and grade levels (1–4 and 5–8) until the predetermined sample size was achieved. In cases where the selected student was absent from school, three repeated visits were made. When a student missed after three visits, he/she considered non-respondents.

Dependent and independent variables

The dependent variables were prevalence, infection intensity, and diagnostic performance of *Schistosoma mansoni* infection. The independent variables consisted of socio-demographic characteristics such as gender, age, and educational status of both parents and family's occupation. Additionally, grade level and the schools attended were considered. The distance of the home from the lake and practice-related factors were also explored, focusing on habits such as swimming, fishing, shoe-wearing, washing clothes, hand washing before meals and after latrine, open-field defecation, trimming finger nail, bathing, and involvement in irrigation activities. Environmental

factors considered included the availability of a latrine at home. Clinical symptoms of the disease were also assessed among study participants.

Data collection methods

Interview

The data was collected using a structured questionnaire. Two trained nurses conducted home-to-home visits to interview the parents or guardians of the children, utilizing the KOBO Collect digital tool. A senior expert and the principal investigator supervised the data collection process, and regular meetings were held to address any challenges and ensure adherence to established protocols and standards.

Stool and Urine sample collection

~~Laboratory investigation~~

Fecal and urine samples were collected in a screw-capped, dry, and clean container ~~pre-labeled~~pre-labelled with the participant's unique identification number. The collected stool samples were transported within an hour of collection in suitable cool boxes at temperatures between 4°C and 6°C for subsequent examination. Samples from Gemeto and Tulla Elementary Schools were transported to Tulla Hospital, while samples from Bushulo and Finchawa Elementary School were ~~transported~~ sent to the Bushulo Mother and Child Health Specialty Center. Sample collection and transportation quality were supervised by the investigator to ensure adherence to procedures and the accuracy of the data results.

Stool sample processing using the Kato-Katz technique

The stool samples were examined using the KK technique to detect parasite eggs. A portion of the sample was processed using a calibrated template holding 41.7 mg of stool. Double slides were prepared for each stool sample. The number of eggs was counted from the double KK microscopic slides, and the average count was converted to eggs per gram of feces (EPG) using a

correction factor of 24. Based on WHO cut-off values, the infection intensity was categorized as light (EPG 1-99), moderate (EPG 100-399), or heavy (EPG >400) [27].

Urine sample processing using POC-CCA rapid test

Each urine sample was processed immediately at the schools using the POC-CCA test, following the manufacturer's instructions [28]. This on-site processing allowed for prompt results and minimized potential delays or degradation of the samples. 100µL of urine was transferred to the circular well of the test cassette and allowed to absorb completely into the specimen pad. The test results were read after 20 minutes of assay development. In cases of discrepancies, the tests were re-examined to ensure accuracy. The POC-CCA tests were scored as negative or positive based on the formation and intensity of the control band. Tests were considered invalid if an internal control band did not appear or if results were not read after more than 25 minutes. In such cases, samples were re-run with a new cassette and scored as necessary.

Data quality assurance

The questionnaire was initially developed in English and then translated into Amharic and Sidamu Afoo, the local language of the participants. It was then translated back into English to ensure consistency. A pre-test was conducted on 5% of the sample size at Tullo Primary School, which was not part of the selected schools for the final study. The questionnaire was subsequently modified based on the findings from the pre-test. The completeness of data collection was overseen daily by the assigned supervisor.

The senior laboratory technologist provided refresher training to laboratory professionals. Standard operating procedures (SOPs) for pre-analytical, analytical, and post-analytical procedures were implemented throughout sample analysis. To guarantee quality control as an internal quality control measure, 10% of the slides were randomly selected and re-examined by a senior laboratory technologist at Tulla Hospital and Bushulo Mother and Child Health Specialty Centre and by another experienced technologist at HUCSH as part of an external laboratory

evaluation. Both stool and urine samples were collected between 09:00-12:00 A~~a~~.M~~m~~. and 03:00-05:00 A~~a~~.M~~m~~. local time each day of the survey.

Data analysis

Data were~~as~~ entered into the KOBO Collect digital tool and analyzed using IBM SPSS Statistics Version 26. ~~using SPSS version 26 software.~~ Descriptive statistics, including frequency, percentage, mean, standard deviation, and geometric mean were calculated. ~~The prevalence of *S. mansoni* and STH infection was presented as a percentage,~~ I~~while i~~nfection intensity of *S. mansoni* was also amalyzed and reported ~~classified~~ as low, moderate, or heavy according to WHO guidelines [4]. The mean egg count was reported as a geometric mean. A logistic regression model was used to identify potential determinants of *S. mansoni* and STH infections. Initially, a bivariate analysis was conducted to calculate the crude odds ratios (COR) for each variable and to determine which variables were significantly associated with the outcome. To control for confounding variables, factors with a p-value of ≤ 0.25 from the bivariate analysis were included in the multivariable logistic regression analysis, allowing for the calculation of adjusted odds ratios (AOR). A p-value of less than 0.05 was considered statistically significant.

~~A logistic regression model was employed to identify potential determinants of *S. mansoni* and STH infections and assess the strength of associations. Crude odds ratios (COR) from bivariate analysis were calculated to identify significant variables associated with the outcome. To control for confounding effects, factors with a p-value ≤ 0.25 in bivariate analysis were included in multivariable logistic regression, allowing for the determination of adjusted odds ratios (AOR). A p-value < 0.05 was considered statistically significant.~~ The diagnostic performance of the POC-CCA test was assessed using metrics like sensitivity and specificity, often using the KK as a reference standard.~~The diagnostic performance of the POC-CCA was assessed by calculating sensitivity, specificity, and other relevant metrics, with these tests regarded as reference standards~~ [29]. The KK method is the most commonly used diagnostic approach for helminths,

Formatted: Font: Italic

Formatted: Font: Italic

epidemiology studies, and treatment evaluation which shows good performance, particularly at detecting helminth infections of moderate and heavy intensity.

Kappa statistic was employed to quantify the agreement between the two diagnostic methods, and the concordance rate was determined as the proportion of cases that yielded identical results. While the McNemar chi-square test was employed to compare the statistically significant differences in sensitivity and specificity between the two diagnostic methods, focusing on cases where the tests disagree.

~~Kappa statistics were utilized to quantify agreement between the two diagnostic methods, and the concordance rate was calculated as the proportion of cases yielding identical results. McNemar's chi-square test was applied to analyze paired proportions of positive and negative results to investigate detection rate differences further.~~

Ethical consideration

Ethical clearance was obtained from Hawassa University, College of Medicine, and other Health Sciences Internal Review Board (IRB) with a reference number: IRB/172/16 ~~E.C~~ in 2024. A letter of support from Hawassa University School of Medical Laboratory was provided to the Sidama Regional State Health Bureau, as well as to the health and education offices in Tulla district and the directors of the selected schools. Participation was fully voluntary, with written informed consent obtained from the parents/guardians of the children enrolled. Assent was obtained from students aged ≥ 12 years, following parent/guardian consent. Explicit written assent was not obtained for children under 12, as they may lack the developmental capacity to understand this concept. However, these children were verbally informed about the study in simple language, and their willingness or unwillingness to participate was respected. Schoolchildren who tested positive for any pathogenic intestinal parasites received appropriate treatment administered by qualified public health professionals. All data collected during the study were kept strictly confidential and used exclusively for research purposes. The study

procedures were conducted in full compliance with relevant ethical guidelines and regulatory standards

Result

Socio-demographic characteristics of the study participants

A total of 484 students were invited to participate in the study, of whom 477 met the inclusion criteria and agreed to take part, resulting in a response rate of 98.5%. The study sample ~~included~~ comprised 208 (43.6%) males and 269 (56.4%) females. Participants' ages ranged from 5 to 19 years, with a mean age of 11.94 years and a standard deviation of 2.76 years. Most of the study participants (62.7%) were 10-14 years old. The majority of students reside within 1 km of a lake (66.5%) and are under the care of their private (daily laborer) parents or guardians (41.7%). The students were selected from 4 primary schools: Bushulo 143 (30%), Finchawa 53 (11.1%), Gemeto 116 (24.3%), and Tulla 165 (34.6%) **(Table 1).**

Prevalence of *Schistosoma mansoni* infection

The prevalence of *S. mansoni* among school children, as determined by the two combined diagnostic methods, was found to be 36.5%. Specifically, ~~The combined results of the 2 diagnostic methods showed an overall prevalence of *S. mansoni* to be 36.5%,~~ ~~with~~ 20.1% (96/477) detected using the KK technique and 34.4% (164/477) detected using the POC-CCA test. Compared to sex for *S. mansoni*, boys were more infected than girls (25.0% *vs*. 16.4%). Children aged 10 – 14 years were more infected (22.4%) than those aged 5– 9 years (9.1%). There was a significant variation in the prevalence of *S. mansoni* across different schools (Table 1). The highest prevalence of *S. mansoni* infection was observed among students attending Bushulo Elementary School (36.4%), followed by Finchawa (24.5%), Gemeto (14.6%), and Tulla (8.5%). Children living within 1 km of the lake had a higher *S. mansoni* infection rate (39.6%) compared to those residing more than 1 kilometre away, who had an infection rate of 14.8%. Additionally, children who had greater contact with the contaminated lake exhibited a higher prevalence of *S. mansoni* infection compared to those who had no exposure. Specifically, the infection rate among children who engaged in fishing was 35.1%, notably higher than the 18.1% infection rate among those who did not participate in fishing activities. Table 1 shows detailed information about various activities at the lake and their associated infection rates~~The highest prevalence of *S. mansoni* infection, 30.5%, was observed among students attending schools near the lake (Bushulo and Finchawa). Overall, the prevalence of *S. mansoni* infection was highest among students aged 10 – 14 years~~ (Table 1).

Potential associated risk factors of *S. mansoni* infections.

In the bivariate logistic regression analysis, several activities were identified as potential risk factors for *S. mansoni* infection. These included washing clothes (13%, 62/477), bathing (31.7%, 151/477), swimming (32.9%, 157/477), fishing (11.9%, 57/477), and engaging in irrigation activities (24.9%, 119/ 477), all with a p-value of ≤ 0.25. Additionally, socio-demographic factors such as gender, age, the parents' educational status, attending Bushulo School and the distance of the residential area

Formatted: Font: Italic

Formatted: Font: Italic

Formatted: Font: Italic

Formatted: Font: Italic

Formatted: Font: Italic

from the lake were also identified as potential predictors of *S. mansoni* infection ~~In the bivariate logistic regression analysis, activities such as washing clothes 13% (62/477), bathing 31.7% (151/477), swimming 32.9% (157/477), fishing 11.9% (57/477), and engaging in irrigation activities 24.9% (119/477), were identified as potential risk factors of *S. mansoni* infection with a p-value ≤ 0.25. Socio-demographic factors such as gender, age, school (Bushulo), educational status of the father (primary school), educational status of the mother (primary school), and distance of residential area from the lake were also potential predictors of *S. mansoni* infection~~ (Table 1). Multivariate logistic regression analysis was applied to avoid confounding factors and only students engaged in irrigation were 2.4 times more likely to be infected than those not involved (AOR = 2.43; 95% CI: 1.38-4.29, P = 0.002), schoolchildren with swimming habits exhibited a 2.4-fold increase in the likelihood of infection (AOR = 2.41; 95% CI: 1.43-4.34, P = 0.001) and participants who bathed in the lake were 1.8 times more likely to be infected than those who did not engage in this activity (AOR = 1.80; 95% CI: 1.05-3.12, P = 0.033). A school-wide analysis revealed that students attending Bushulo primary school were 4.7 times more likely to contract *S. mansoni* infection compared to their peers at Tulla primary school (AOR = 4.68; 95% CI: 1.87-11.74, P = 0.001) (Table 1).

Table 1: The distribution of *Schistosoma mansoni* infection by socio-demographic and other factors among schoolchildren in Tulla district, Sidama region, Southern Ethiopia 2024 (n = 477).

Variables	*S. mansoni* Positive %	Negative %	Total %	COR (95% CI)	p-value	AOR (95%CI)	p-value
**Gender**							
Male	52(25.0)	156(75.0)	208(43.6)	1		1	
Female	44(16.4)	225(83.6)	269(56.4)	0.587(0.374-0.920)	0.020	0.607(0.359-1.027)	0.063
Age							
5-9	7(9.1)	70(90.9)	77(16.1)	1			
10-14	67(22.4)	232(77.6)	299(62.7)	2.888(1.268-6.578)	0.012	2.118(0.841-5.330)	0.111
15-19	22(21.8)	79(78.2)	101(21.2)	2.785(1.122-6.914)	0.027	1.442(0.495-4.198)	0.502
Name of School							
Bushulo	52(36.4)	91(63.6)	143(30)	6.163(3.234-11.746)	0.001	4.685(1.869-11.742)	**0.001****
Finchawa	13(24.5)	40(75.5)	53(11.1)	3.505(1.526-8.051)	0.003	1.444(0.486-4.297)	0.509
Gemeto	17(14.6)	99(85.4)	116(24.3)	1.852(0.874-3.926)	0.108	1.528(0.662-3.525)	0.320
Tulla	14(8.5)	151(91.5)	165(34.6)	1			
Educational status of the father							
No formal education	17(18.5)	75(81.5)	92(19.3)	1		1	
Primary school	58(26.0)	165(74.0)	223(46.75)	1.551(0.846-2.841)	0.156	1.420(0.690-2.921)	0.341
Educational status of the mother							
No formal education	3(7.0)	40(93.0)	43(9)	1		1	
Primary school	62(23.2)	205(76.8)	267(56)	4.033(1.206-13.485)	0.024	3.391(0.889-12.937)	0.074
Secondary school	25(18.8)	108(81.2)	133(28)	3.086(0.883-10.787)	0.078	2.178(0.535-8.859)	0.277
College and above	6(17.7)	28(82.3)	34(7)	2.857(0.658-12.397)	0.161	2.384(0.414-13.719)	0.331
Distance of residential area							
Within 1km	49(30.6)	111(69.4)	160(33.5)	1		1	
More than 1km	47(14.8)	270(85.2)	317(66.5)	0.394(0.250-0.623)	0.001	1.194(0.615-2.318)	0.601
Bathing in the lake							
Yes	43(28.5)	108(71.5)	151(31.7)	2.051(1.295-3.248)	0.002	1.804(1.048-3.107)	**0.033***
No	53(16.3)	273(83.7)	326(68.3)	1		1	
Swimming habit							
Yes	50(31.9)	107(68,1	157(32.9)	2.783(1.780-4.403)	0.001	2.407(1.430-4.052)	**0.001****

No	46(14.4)	274(85.6)	320(67.1)	1			
Fishing							
Yes	20(35.1)	37(64.9)	57(12)	2.447(1.345-4.449)	0.003	1.135(0.533-2.417)	0.743
No	76(18.1)	344(71.9)	420(88)	1			
Washing clothes along the lake shore							
Yes	19(30.6)	43(69.4)	62(13)	1.940(1.071-3.513)	0.029	2.016(0.934-4.350)	0.074
No	77(17.3)	338(82.7)	415(87)	1			
Involvement in irrigation							
Yes	44(37.0)	75(63.0)	119(25)	3.452(2.148-5.547)	0.001	2.429(1.377-4.287)	**0.002***
No	52(14.5)	306(85.5)	358(75)	1			

AOR, Adjusted Odds Ratio; CI, Confidence interval; COR, Crude Odds Ratio; *, Significant at $p < 0.$~~0~~

422

17

Infection intensity of *Schistosoma mansoni* infection

Out of 174 children infected with *S. mansoni* ~~infected study participants~~, 96 tested positive by the KK technique using a standard volume~~amount~~ of stool that enables the quantification of ova per gram of stool. The minimum and maximum eggs per gram of stool were 24 and 672, respectively. Likewise, the arithmetic and geometric mean values were 189.75 and 139.39, respectively. According to WHO classification, among 96 students infected with *S. mansoni*, 33 (34.4%) had light intensity, 54 (56.2%) had moderate intensity, and 9 (9.4%) had a heavy intensity ~~of *S. mansoni* infection~~ (Fig 2).

Fig 2: The prevalence of *S. mansoni* infection intensity across 477 schoolchildren in Tulla district Sidama Region, southern Ethiopia, 2024

There was no significant variation in the intensity of infection across age groups. However, the intensity of infection across age groups varies, with the younger age groups, 10 – 14 years, having less moderate intensity (49.25%) compared to older age groups, 15 – 19 years (72.7%) and the younger age group, 5 – 9 years (71.4%). The highest light intensity of *S. mansoni* infection (64.3%) and the lowest moderate intensity (35.7%) were observed among students at Tulla Elementary School, with no cases of heavy intensity when compared to students at other elementary schools ~~As shown in Table 2, moderate infection with *S. mansoni* among males accounted for 53.9%, followed by 34.6% with light infection and 11.5% with heavy infection. Among individuals in the 15 to 19 age group, most infections were of moderate intensity (72.7%), followed by light intensity (18.2%) and heavy intensity (9.1%). When comparing different schools, more than half of the infections reported by students from Bushulo Primary School were of moderate intensity (61.5%), while 27% were classified as light intensity and 11.5% as heavy intensity. In contrast, the majority of infections among students from Tulla Primary School were of light intensity (64.3%) and moderate intensity (35.7%)~~ **(Table 2)**

Formatted: English (United Kingdom)

Formatted: Font: Italic

Formatted: Font: Italic

Table 2: Gender, age, and school-based variation in *S. mansoni* infection intensity among schoolchildren in Tulla District Sidama Region southern Ethiopia 2024 (n = 477).

Formatted: Font: Italic

Variable	N	Intensity of infection n (%)		
		Light	Moderate	Heavy
**Gender**				
Male	52	18(34.6)	28(53.9)	6(11.5)
Female	44	15(34.1)	26(59.1)	3(6.8)
**Age**				
5-9	7	2(28.6)	5(71.4)	0(0)
10-14	67	27(40.3)	33(49.25)	7(10.45)
15-19	22	4(18.2)	16(72.7)	2(9.1)
**Schoolchildren**				
Bushulo	52	14(27)	32(61.5)	6(11.5)
Finchawa	13	4(31)	7(53.8)	2(15.2)
Gemeto	17	6(35.3)	10(58.8)	1(5.9)
Tulla	14	9(64.3)	5(35.7)	0(0)

Key - N: refers to the total number of infected; whereas, n: refers to the number of infected according to intensity classes.

Prevalence of soil transmitted helminthic infections

Aside from *S. mansoni*, the prevalence of intestinal helminths among the schoolchildren was 233 (48.8%). The most common STH was *Ascaris lumbricoides* 162 (34%), followed by *Trichuris trichuria* 39 (8.2%) and hookworm 27 (5.7%). There were also intestinsl Other helminth parasites reported in this, including included *Taenia* species 6 (1.3%), *Hymenolepis nana* H. nana) 3 (0.6%), and *Enterobius vermicularis* (*E. vermicularis*) 1 (0.2%) (Fig Fig. 3). The younger age group of 5–9 years was more infected (49.4%) than those aged 15–19 years (43.6%). The data revealed that children whose parents had no formal education were more likely to be infected with STH (59.4%) compared to those whose parents had a college education (27.3%). Additionally, higher STH infection rates were found among children from families where the parents worked as daily labourers (54.5%) or farmers (50.8%). Children who did not wash their hands after using the latrine exhibited a significantly higher STH infection rate of 52.8%, while those who practised hand washing had a much lower rate of 12.8%. Similarly, children who neglected to wash their hands before meals had an infection rate of 53.4%, compared to 33.6% for those who did wash their hands before eating. Furthermore, children who did not trim their nails had an increased risk of contracting STH infections, with an infection rate of 54.7% compared to 32.7% for those who regularly trimmed their nails, as shown in Table 3.

Formatted: Font: Italic

Formatted: Font: Italic

Formatted: Font: Not Bold

Formatted: English (United Kingdom)

Fig 3: The prevalence of intestinal helminthic infections across 477 school children in Tulla district Sidama Region, southern Ethiopia, 2024

Potential associated risk factors of Soil transmitted helminthic infections.

In the bivariate logistic regression analysis, both hygiene-related practices and socio-demographic factors were identified as potential risk factors for intestinal helminthic infections. STH infection was associated with latrine use, hand washing practices, fingernail trimming habits, educational

level, and occupation, with a p-value of ≤ 0.25 ~~Household latrine availability (61%, 105/477), hand washing after latrine use (52.8%, 227/477), fingernail trimming (56.4%, 194/477), and hand washing before meals (53.4%, 196/477) were all associated with STH infections, with a p-value ≤ 0.25. Additionally, socio-demographic factors, including attending Tulla Elementary School, grade level, father's educational status, and family occupation (farmer), emerged as candidate predictors~~ **(Table 3).**

Fially, the multivariate regression analysis revealed that latrine use, school location, educational status, nail trimming practices, and hand washing after using the latrine were significantly associated with STH infection, with a p-value of ≤ 0.05 as summarised in Table 3. Students from Tulla Elementary School had 0.47 times lower odds of STH infection compared to their counterparts at Bushulo Elementary School (AOR = 0.469; 95% CI: 0.256-0.861, P = 0.015). Specifically, children whose fathers had no formal education were found to be 4.2 times more likely to be infected than those whose fathers had completed college or higher education (Adjusted Odds Ratio = 4.161; 95% Confidence Interval: 1.276-13.570, P = 0.018).

Formatted: English (United Kingdom)

The presence of latrines at home also significantly influenced infection rates, with children having access to latrines exhibiting 0.63 times lower odds of infection than those without such facilities (AOR = 0.626; 95% CI: 0.393-0.996, P = 0.048). Furthermore, hygiene practices emerged as critical determinants, as children who washed their hands after using the latrine had a remarkable 0.2 times lower odds of contracting STH infections compared to those who did not (AOR = 0.199; 95% CI: 0.072-0.551, P = 0.002). Similarly, regular nail trimming was associated with a significant reduction in infection risk, with children who trimmed their nails having 0.4 times lower odds of STH infection than those who did not (AOR = 0.413; 95% CI: 0.250-0.682, P = 0.001) (Table 3).

Table 3: Univariate and multivariate logistic regression analyses for socio-demographic and other potential risk factors associated with soil transmitted helminthic infections among school children in Tulla district, Sidama region, Southern Ethiopia 2024 (n = 477).

Variables	STH Positive %	Negative %	Total %	COR (95% CI)	p-value	AOR (95%CI)	p-value
Gender							
Male	97(46.6)	111(53.4)	208(43.6)	1			
Female	136(50.6)	133(49.4)	269(56.4)	1.170(0.814-1.681)	0.395		
Age							
5-9	38(49.4)	39(50.6)	77(16.1)	1			
10-14	151(50.5)	148(49.5)	299(62.7)	1.047(0.634-1.728)	0.857		
15-19	44(43.6)	57(56.4)	101(21.2)	0.792(0.437-1.437)	0.443		
School							
Bushulo	76(53.2)	67(46.8)	143(30)	1		1	
Finchawa	28(52.8)	25(47.2)	53(11.1)	0.987(0.525-1.857)	0.969		
Gemeto	54(46.5)	62(53.5)	116(24.3)	0.768(0.470-1.254)	0.292		
Tulla	75(45.5)	90(54.5)	165(34.6)	0.735(0.469-1.151)	0.178	0.469(0.256-0.861)	**0.015***
Grade level							
1-4	118(48.2)	127(51.8)	245(51.4)	1		1	
5-8	126(54.3)	106(45.7)	232(48.6)	1.279(0.893-1.834)	0.180	0.848(0.495-1.453)	0.548
Educational status of father							
No formal education	55(59.8)	37(40.2)	92(19.3)	3.964(1.420-11.066)	0.009	4.161(1.276-13.570)	**0.018***
Primary school	115(51.6)	108(48.4)	223(46.7)	2.840(1.072-7.523)	0.036	2.957(0.991-8.818)	0.052
Secondary education	57(40.7)	83(59.3)	140(29.4)	1.831(1.072-7.523)	0.234	1.616(0.547-4.775)	0.385
College and above	6(27.3)	16(72.7)	22(4.6)	1		1	
Occupation of family							
Civil servant	6(31.6)	13(68.4)	19(4)	1		1	
Merchant	56(41.5)	79(58.5)	135(28.3)	1.536(0.550-4.285)	0.412		
Farmer	63(50.8)	61(49.2)	124(26)	2.238(0.799-6.264)	0.125	0.199(0.013-3.028)	0.245
Private (Daily laborer)	108(54.3)	91(45.7)	199(41.7)	2.571(0.940-7.037)	0.066	0.262(0.017-3.932)	0.332
Latrine availability at home							
Yes	128(42)	177(58)	305(63.9)	1		1	
No	105(61)	67(39)	172(36.1)	0.461(0.315-0.676)	0.001	0.626(0.393-0.996)	**0.048***
Hand washing after latrine							
Yes	6(12.8)	41(87.2)	47(9.9)	1		1	
No	227(52.8)	203(47.2)	430(90.1)	0.131(0.54-0.315)	0.001	0.199(0.072-0.551)	**0.002***
Shoe wearing							
Yes	198(49.25	204(50.75)	402(84.3)	1			
No	35(46.7)	40(53.3)	75(15.7)	0.902(0.550-1.477)	0.681		
Finger nail trimming							
Yes	39(29.3)	94(70.7)	133(27.9)	1		1	
No	194(56.4)	150(43.6)	344(72.1)	0.321(0.209-0.493)	0.001	0.413(0.250-0.682)	**0.001***
Hand washing before meal							
Yes	37(33.6)	73(66.4)	110(23)	1		1	
No	196(53.4)	171(46.6)	367(77)	0.442(0.283-0.890)	0.001	0.585(0.337-1.015)	0.058

Open field defecation					
Yes	24(48)	26(52)	50(10.5)	1.039(0.578-1.867)	0.899
No	209(48.9)	218(51.1)	427(89.5)	1	

Key - AOR: Adjusted Odds Ratio, CI: Confidence interval, COR: Crude Odds Ratio, *: Significant at $p < 0.05$, STH: Soil-transmitted helminths.

~~In the multivariate logistic regression analysis, several socio-demographic and behavioral factors were found to significantly influence the prevalence of STH infections among schoolchildren. Students from Tulla Elementary School had 0.47 times lower odds of STH infection compared to their counterparts at Bushulo Elementary School (AOR = 0.469; 95% CI: 0.256-0.861, P = 0.015). Specifically, children whose fathers had no formal education were found to be 4.2 times more likely to be infected than those whose fathers had completed college or higher education (Adjusted Odds Ratio = 4.161; 95% Confidence Interval: 1.276-13.570, P = 0.018).~~

~~The presence of latrines at home also significantly influenced infection rates, with children having access to latrines exhibiting 0.63 times lower odds of infection than those without such facilities (AOR = 0.626; 95% CI: 0.393-0.996, P = 0.048). Furthermore, hygiene practices emerged as critical determinants, as children who washed their hands after using the latrine had a remarkable 0.2 times lower odds of contracting STH infections compared to those who did not (AOR = 0.199; 95% CI: 0.072-0.551, P = 0.002). Similarly, regular nail trimming was associated with a significant reduction in infection risk, with children who trimmed their nails having 0.4 times lower odds of STH infection than those who did not (AOR = 0.413; 95% CI: 0.250-0.682, P = 0.001) (Table 3).~~

Detection and performance evaluation of the POC-CCA test

Out of 477 samples examined, the POC-CCA rapid test identified 86 (18.0%) true positives, 303 (63.5%) true negatives, and 10 (2.1%) discrepancies (false negatives) compared to the KK technique. Using KK as the gold standard or ‘reference test’ [29], the POC-CCA sensitivity was 89.6% (95% CI: 82.3 - 94.4), specificity 79.5% (95% CI: 75.2-83.3), NPV 96.8% (95% CI: 94.1–98.2), PPV 52.4% (95% CI: 44.6 – 60.1), Negative LR 0.13 (95% CI: 0.07–0.23) and Positive L4.3

(95% CI: 3.38–5.63) ~~89.6% (95% CI: 83.5-95.7) and specificity 79.6% (95% CI: 75.6-83.6)~~ **(Table 4)**

Table 4: Performance of POC-CCA test using Kato-Katz as a gold standard across 477 school children in Tulla district Sidama Region souther Ethiopia 2024

**Method**	**Result**	**KK (Gold standard) Pos**	**Neg**	**Sensitivity % (95% CI)**	**Specificity % (95% CI)**	**NPV % (95 CI)**	**PPV % (95 CI)**	**LR^- % (95 CI)**	**LR^+ % (95 CI)**
**POC-CCA**	**Pos**	**86**	**78**	**89.6 (82.3-94.4)** ~~**89.6 (80-99.2)**~~	**79.5 (75.2-83.3)** ~~**79.6 (73.9-85.3)**~~	**96.8 (94.1-98.2)** ~~**96.8 (90.7-100)**~~	**52.4 (44.6-60.1)** ~~**52.4 (45.2-59.6)**~~	**0.13 (0.07-0.23)** ~~**0.131 (0.057-0.19**~~	**4.3 (3.38-5.63)** ~~**4.39 (3.93-5.10)**~~
	**Neg**	**10**	**303**						

Key - KK: Kato-Katz POC-CCA: Point of Care Circulating Cathodic Antigen, NPV: Negative Predictive value, PPV: Positive Predictive value, NLR^-: Negative Likely hood ratio, LR^+: Positive Likely hood ratio, Pos: Positive, Neg: Negative.

The agreement between the two diagnostic methods was quantified using Cohen's Kappa statistics which yielded a kappa value of 0.55. Furthermore, the overall concordance rate between the KK and POC-CCA was 81.5%. However, to assess the statistical significance of the differences in results between the two tests, we employed the McNemar Chi-square test. The result revealed a value of 52.3

Discussion

Although large-scale deworming programmes targeting specific groups have been successful in reducing morbidity associated with *S. mansoni* and STH infections, the absence of a sensitive diagnostic method for detecting low infection intensities and reinfection without additional intervention measures remains unavoidable. This study assessed the prevalence and related risk factors of these parasites, and evaluated the intensity and diagnostic performance of the POC-CCA rapid test for *S. mansoni* among schoolchildren in the Tulla district of the Sidama region, Ethiopia. This this study aimed to assess the prevalence and risk factors of *S. mansoni* and STH infections among schoolchildren. It also aimed to assess the intensity of *S. mansoni* and compare the diagnostic performance of the POC-CCA test with KK.

Formatted: Font: Italic

Formatted: Font: Italic

Formatted: English (United States)

Prevalence of *Schistosoma mansoni* infection

Formatted: Font: Bold, English (United States)

The prevalence of *S. mansoni* infection was 20.1% (95% CI: 16.8-23.7) using the KK technique. This finding highlights the need for annual deworming of schoolchildren in the study area, as the prevalence falls within the moderate range (≥ 10% but < 50%) according to WHO guidelines The prevalence of *S. mansoni* infection was 20.1% (95% CI: 16.8-23.7) using the KK technique. This indicates the need for biannual deworming of schoolchildren in the study area with a moderate prevalence (≥ 10% but < 50%) according to WHO guidelines [4]. This result is consistent with previous studies conducted in the region, which reported prevalence rates as follows: Jimma. 20.2% This result aligns with previous studies from Jimma, 20.2% [30], Kersa, 19.9% [31], and Mekelle, 23.9% [32], in Ethiopia. Furthermore, the prevalence reported in this study is consistent with findings from Egypt, 19.1% [33], and Angola, 21.2% [34]. However, it was higher than the prevalence rates observed in Jiga, at 15.2% [35], Guangua 12.6% [36], and Wondo districts

11.4% [37]. In contrast, the prevalence found in this study is lower than that reported in Lake Tana, 34.9%

~~The prevalence in this study is lower than findings from Lake Tana in northwest Ethiopia, 34.9%~~ [38], Jimma town,~~ Ethiopia,~~ 28.7% [39],~~.~~ ~~The prevalence of this study is lower than rates reported in~~ Maksegnit, Debark, Sanja, and Chuahit districts ~~in northwest Ethiopia at~~ 33.5% [40], Abbey and Didessa valleys 53.9% [41],~~ and~~ Wolaita~~ southern Ethiopia~~ 81.3% [42], Kenya, 53.7% [43], Congo, 90.2% [44], and Tanzania, 90.6% [45].

The variations in the prevalence of *S. mansoni* infection across different regions may be attributed to differences in temperature and rainfall patterns that influence snail reproduction and water availability, which in turn affect the local transmission rates of the parasite. Differences in human behavior towards contaminated water sources include swimming or crossing infested water, as well as performing domestic chores in contaminated water bodies. Inadequate access to safe drinking water and the lack of proper sanitation facilities, leading to open defecation near water sources, influence the transmission cycle of the parasite.

Socioeconomic factors such as increased population density and migration to new localities contribute to the spread of schistosomiasis by increasing the potential for exposure to infested water. Altered agricultural practices and the use of untreated water for irrigation can enhance opportunities for human-water contact and snail proliferation that may also favour the rate of infection. Environmental factors may also impact disease transmission variation across different regions, including the proximity to snail habitats, the construction of dams, land cover (particularly elevation), rainfall patterns, seasonal land surface temperatures, and the presence of flooded agricultural areas. Additionally, it is crucial to consider differences in sample sizes, study designs and diagnostic methods applied.

~~The variations in the prevalence of *S. mansoni* infection across different regions may be attributed to several factors related to human behavior towards contaminated water sources, socioeconomic conditions and the rate of reinfection. These conditions include living environments, education levels, occupations, and the immune status of the individuals being studied. Furthermore, climate change may also influence human exposure to schistosomiasis. Environmental factors may also impact disease transmission variation across different regions include the proximity to snail habitats, the construction of dams, land cover (particularly elevation), rainfall patterns, seasonal land surface temperatures, and the presence of flooded agricultural areas. Additionally, it is crucial to consider differences in sample sizes, study designs and diagnostic methods applied.~~

Formatted: Font: Italic

Intensity of *S. mansoni* infection

Formatted: Font: Italic

Formatted: Font: Bold

In the present study, out of 174 participants infected with *S. mansoni*, 96 were confirmed positive using the KK technique, a standard diagnostic method valued for its ability to quantify EPG of stool. The intensity of infection ranged from 24 to 672 EPG, with an arithmetic mean of 189.75 and a geometric mean of 139.39. The noticeable discrepancy between the arithmetic and geometric means reflects the presence of a right-skewed distribution of egg counts. This pattern is consistent with the typical epidemiology of schistosomiasis, where a minority of individuals harbor high worm burdens, while the majority exhibit low to moderate infection levels [16].

The moderate infection intensity reported in this study is consistent with findings from various regions in Ethiopia [41], [46] and in Tanzania [45]. This difference might be due to repeated exposure of schoolchildren to water bodies infested with the infective stage. The intensity of *S. mansoni* infection was found to be slightly higher in male students compared to female students, which is consistent with findings from Kemissie and Wondo Genet [46] and Jimma Town [47] in

Ethiopia. The observed difference in proportion might be linked to male students' greater exposure to outdoor activities like fishing, swimming, and irrigation compared to female students.

A significant association was found between *S. mansoni* infection and schoolchildren who engaged in swimming and bathing activities in the lake, with infection risks being 2.4 times higher for swimming and 1.8 times higher for bathing than those who did not partake in these habits. This association between *S. mansoni* infection and swimming habits among schoolchildren aligns with previous studies [18], [36] and is further supported by studies linking bathing habits to infection [38].

Additionally, a strong association was noted between *S. mansoni* infection and participation in irrigation activities, with students involved in irrigation being 2.4 times more likely to be infected than those not engaged in such activities, consistent with prior findings [18], [36]. Notable differences in infection rates were observed among students across the various schools in the study areas. Children attending Bushulo Primary School were 4.7 times more likely to be infected with Schistosoma than their peers at Tulla Primary School. This finding aligns with previous studies [18], [38]. This might be associated with students' residence distance variation from lakes and other water bodies.

Prevalence of soil transmitted helminthic infections

Formatted: Heading 2, Tab stops: Not at 5.55"

Formatted: Font: 16 pt, Bold

The study area displayed a moderate prevalence of STH infections among schoolchildren despite them being part of the deworming target group. Deworming programs have successfully decreased the global morbidity attributed to STHs [48], but the risk of re-infection persists in the absence of improved water, sanitation, and hygiene WASH practices and health education [49]. A

systematic review and meta-analysis estimated that three months after deworming, the prevalence of *A.*~~scaris~~ *lumbricoides*, *T.*~~richuri~~s *trichuria*, and *hookworms* reached 26%, 36%, and 30% of their pre-treatment levels, respectively. By one-year post-treatment, the prevalence rates approached their pre-treatment levels again, with *A. lumbricoides* at 94%, *T. trichuria* at 82%, and *hookworm* infections at 57% [50]. The occurrence of re-infection highlights the importance of a comprehensive approach to controlling STH. This is especially crucial given the increasing interest in ultimately halting STH transmission in specific geographic regions [51]. The current WHO guidelines recommend the evaluation of any STH infections in school-age children after five to six years of periodic chemotherapy using the standard Kato-Katz thick smear [52].

Potential associated risk factors of STH infections

The results ~~findings~~ of this study also provide critical insights into the socio-demographic and environmental determinants associated with STH infections among school children. This research identified several significant factors linked to the prevalence of STH, including the lack of a latrine at home, inadequate hand-washing practices following latrine use, the educational status of fathers, improper fingernail trimming practices, and certain school attendance patterns. The lack of a latrine at home was identified as a significant risk factor for STH infections in this study. This finding highlights the strong connection between poor sanitation and the transmission of helminths. It aligns with extensive research showing that limited access to sanitation facilities, especially at the household level, plays a major role in driving STH infections [53], [54], [55]. Without access to latrines, many individuals are forced to practice open defecation, resulting in the direct release of helminth eggs into the environment and promoting continued transmission. Having access to household latrines significantly reduces the risk of exposure to infectious helminth stages in soil and water by minimizing the dispersion of feces in the surrounding area.

Formatted: Font: Not Italic

Formatted: Font: Not Italic

Formatted: Font: Italic

Formatted: Heading 2, Line spacing: 1.5 lines, Tab stops: Not at 5.55"

Formatted: Font: 16 pt, Bold

Notably, This finding aligns with previous research in the same region, which also identified open field defecation as a key factor contributing to the prevalence of STH [18]. This study reinforces existing evidence by examining sanitation status at the household level instead of relying on broader community-level indicators. By doing so, it provides a deeper understanding of how individual risks are influenced by domestic infrastructure. This highlights the need for targeted public health interventions that extend beyond general sanitation promotion. It is essential to prioritize equal access to latrines and ensure their sustained use at the household level, especially in high-transmission areas where environmental contamination continues to pose a significant challenge.

Hand hygiene practices, especially the failure to wash hands after using the latrine, were significantly associated with the prevalence of STH, which aligns with prior global studies [56], [57], [58]. This highlights the importance of hand washing as a key factor in preventing the transmission of STH [59]. In contrast to previous research in the same area, hand hygiene showed no significant effect [18]. This divergence may be due to variations in study design, changes in hygiene behaviors over time, or previous underestimations of individual practices. Furthermore, recent improvements in sanitation infrastructure, in the absence of corresponding behavioral changes, could increase the relative impact of hand hygiene on the risk of infection. These findings underscore the complex interaction between environmental and behavioral factors that influence the transmission of STH and emphasize the importance of ongoing local assessments.

Moreover, the educational status ~~of~~ was found to be significantly associated c~~correlate significantly~~ with STH prevalence among children, which aligns with previous studies [60], [61].

This highlights the importance of targeting educational programs not only for children but also for parents to foster a supportive environment for health-promoting behaviors. Certain school attendance was also significantly associated with STH infection, in line with previous studies [62], [63]. This might be due to differences in sanitation facilities, hygiene practices, socio-economic status, and health education among students, which can significantly influence the transmission dynamics of STH infections in certain schools.

This study also revealed that the practice of not trimming fingernails was significantly associated with STH infection, which aligns with previous studies [54], [64], [65], [66]. Since long fingernails can harbor soil and pathogens, serving as a reservoir for helminth eggs that can be ingested or transmitted through hand-to-mouth contact, public health initiatives need to promote personal hygiene practices among school-aged children, including regular nail-trimming and cleanliness.

Comparison of the POC-CCA test performance with KK on S. mansoni

Formatted: Font: Bold, Font color: Text 1

The current study found a better diagnostic performance of the POC-CCA assay using a single urine sample than duplicate KK thick smears from a single stool sample. From~~Thus, among~~ the 96 KK positive samples, the POC-CCA test identified 10 (10.4%) as negative. Conversely, with KK negative results, POC-CCA classified only 78 (4.9%) as positive, indicating that the KK technique has high specificity.

In this study, using the KK technique as the gold standard, the POC-CCA test demonstrated higher sensitivity but notably lower specificity. The improved sensitivity (particularly in detecting low-intensity infections) might be attributed to the POC-CCA test's ability to detect trace levels of circulating antigens in urine, allowing for broader detection

of *Schistosoma* infections, including cases that KK might miss due to the known variability in egg excretion and the limited sensitivity of KK, especially in low-endemic settings [67]. However, the specificity of the POC-CCA test was found to be 79.6%, which is consistent with previously reported ranges but remains relatively low [25]. This reduced specificity may be attributed to cross-reactivity with other helminth infections, the subjective interpretation of trace results as positive, and the continued excretion of CCA following recent treatment or in the absence of active infection [68]. Such limitations can result in false positives, particularly in low-endemic or post-treatment settings, potentially leading to an overestimation of prevalence. Despite its limitations, the POC-CCA test is a valuable diagnostic tool in field settings because of its ease of use, rapid results, and high sensitivity. However, its limited specificity highlights the necessity for confirmatory testing, especially in situations involving surveillance, program evaluation, and low-transmission areas where diagnostic accuracy is crucial.

Formatted: Font: Italic

The overall agreement between KK and POC-CCA was moderate, as reflected by a kappa statistic of 0.55 ($p < 0.001$) and an observed agreement rate of 81.5%. While this level of concordance suggests a reasonable overlap between the two diagnostic tools, it must be interpreted cautiously given the limitations of κ statistics, which are sensitive to disease prevalence and imbalanced marginal totals [69]. McNemar's test revealed a statistically significant discordance between the two diagnostic methods, with a chi-square value of 52.3 and $p < 0.001$. This discrepancy was primarily driven by the high number of cases that were positive by the point-of-care circulating cathodic antigen (POC-CCA) test but negative by the Kato-Katz (KK) method. This lack of alignment underscores the fundamentally different targets of the two diagnostic tools: the POC-CCA test detects active infections by identifying CCA in urine, while the KK method relies on detecting *Schistosoma* eggs in stool, which can vary from day to day

and show inconsistencies within the same sample [22]. Therefore, in field settings, especially in low-endemic or post-treatment areas, the POC-CCA test may identify infections that the KK method misses, making it a more sensitive tool for surveillance and monitoring of control programs.

Conclusions

The existence of moderate prevalence of *S. mansoni* and STH infections among school children emphasises the need of annual deworming for school-aged children in the study area. The findings from this study also provide valuable insights into socio-demographic factors, behavioural factors, personal hygiene practices and environmental sanitation association to STH and Schistosoma infections in these children. This highlights the need for additional control measures to supplement deworming programmes, including health education focused on personal hygiene and environmental sanitation. Furthermore, this study shows that KK has low sensitivity and may underestimate the accurate prevalence. In contrast, the POC-CCA test demonstrates higher sensitivity in the study area, making it a more reliable option for case detection and large-scale screening. Therefore, we recommend adopting the POC-CCA test in school-based *S. mansoni* infection control and elimination programmes alongside the KK method, as this could enhance diagnostic accuracy and improve management outcomes. However, to optimise the consistency and utility of this test, further research to standardise the scoring system is strongly recommended.~~There was a moderate prevalence of *S. mansoni* and STH, along with a moderate intensity of schistosomiasis and poor hygiene practices. This situation underscores for additional control measures, including health education. Furthermore, there is potential to incorporate POC-CCA testing alongside the KK method to enhance~~

~~diagnostic accuracy in areas with low *S. mansoni* infection rates to monitor and improve mass deworming program disease management outcomes.~~

Formatted: Font: Not Bold, English (United Kingdom)

Limitations

This study has a few limitations that should be acknowledged. (i), the cross-sectional design provides a snapshot of *S. mansoni* and STH infection at a single point in time, which may not capture seasonal variations or fluctuations in infection rates over longer periods. (ii), the use of a single stool sample per participant for the KK technique may have led to underestimation of infection prevalence and intensity, given the known day-to-day variability in helminth egg excretion. (iii), the purposive selection of schools limits the generalizability of the findings to the larger community in the study area.

Acknowledgement

Primarily, we would like to thank Hawassa University, School of Medical Laboratory Sciences, for funding this research. Then our thanks go to the study participants and the school community for their cooperation, as their contributions were crucial to its success. Finally, we would also like to thank Tulla Primary Hospital and Bushulo Mother and Child Health Specialty Center staff, especially laboratory professionals, for their dedication and hard work in collecting and conducting experiments.

References

~~[1] 2021 Schistosomiasis and soil- transmitted helminthiases: progress report, "Weekly epidemiological record Relevé épidémiologique hebdomadaire," *Wkly. Epidemiol. Rec.*, vol. 97,~~

no. 48, pp. 621–632, 2022.

[2] Natalie G *et al.*, "The prevalence of schistosomiasis in Uganda : A nationally representative population estimate to inform control programs and water and sanitation interventions," *PLoS Negl. Trop. Dis.*, vol. 13, no. 8, pp. 1–21, 2019.

[3] S. Hussen, D. Assegu, B. T. Tadesse, and T. Shimelis, "Prevalence of Schistosoma mansoni infection in Ethiopia : a systematic review and meta-analysis," *Trop. Dis. Travel Med. Vaccines*, vol. 7, no. 4, pp. 1–12, 2021.

[4] WHO, *WHO GUIDELINE on control and elimination of human schistosomiasis*, vol. 3, no. 2. 2022. [Online]. Available: http://dx.doi.org/10.1016/j.xpro.2020.100074%0Ahttp://dx.doi.org/10.1074/mcp.R115.052902%0Ahttp://dx.doi.org/10.1016/S0140-6736(13)61949-2%0Ahttp://dx.doi.org/10.1016/j.bbagen.2016.09.015

[5] H. Ahmed *et al.*, "Prevalence , risk factors , and clinical manifestations of schistosomiasis among school children in the White Nile River basin , Sudan," *Parasit. Vectors*, vol. 7, no. 1, pp. 1–11, 2014.

[6] Nathan *et al.*, "Review of 2022 WHO guidelines on the control and elimination of schistosomiasis," *Lancet Infect. Dis.*, vol. 22, no. 11, pp. e327–e335, 2022, doi: 10.1016/S1473-3099(22)00221-3.

[7] A. Dahal, E. Francis, J. Francis, and F. Wamtas, "Soil-transmitted helminths and associated risk factors among elementary school pupils in Dadin Kowa, Jos," *Niger. Med. J.*, vol. 60, no. 4, p. 181, 2019, doi: 10.4103/nmj.nmj_62_19.

[8] M. Getaneh, T. Hailegebriel, and A. Munshea, "Prevalence and Associated Risk Factors of Soil-Transmitted Helminth Infections among Schoolchildren around Lake Tana , Northwest Ethiopia," *J. Parasitol. Res.*, vol. 2022, p. 13, 2022.

[9] M. Wale and S. Gedefaw, "Prevalence of Intestinal Protozoa and Soil Transmitted Helminths Infections among School Children in Jaragedo Town, South Gondar Zone of Ethiopia," *J. Trop. Med.*, vol. 2022, p. 11, 2022, doi: 10.1155/2022/5747978.

[10] B. Beyene, S. Vaz Nery, T. Lambiyo, and T. Shimelis, "Schistosoma mansoni and soil-transmitted helminth infections among schoolchildren living along the shore of Lake Hawassa, southern Ethiopia," *Parasites and Vectors* , vol. 17, no. 1, pp. 1–14, 2024, doi: 10.1186/s13071-024-06578-x.

[11] R. L. Pullan, J. L. Smith, R. Jasrasaria, and S. J. Brooker, "Global numbers of infection and disease burden of soil transmitted helminth infections in 2010," *Parasites and Vectors*, vol. 7, no. 1, pp. 1–19, 2014, doi: 10.1186/1756-3305-7-37.

[12] I. Östan, A. A. Kilimcioğlu, N. Girginkardeşler, B. C. Özyurt, M. E. Limoncu, and Ü. Z. Ok, "Health inequities: Lower socio-economic conditions and higher incidences of intestinal parasites," *BMC Public Health*, vol. 7, pp. 1–8, 2007, doi: 10.1186/1471-2458-7-342.

[13] N. R. De Silva, S. Brooker, P. J. Hotez, A. Montresor, D. Engels, and L. Savioli, "Soil-transmitted helminth infections: Updating the global picture," *Trends Parasitol.*, vol. 19, no. 12, pp. 547–551, 2003, doi: 10.1016/j.pt.2003.10.002.

[14] N. Negussu *et al.*, "Ethiopia schistosomiasis and soil-transmitted helminthes control programme: Progress and prospects," *Ethiop. Med. J.*, vol. 55, no. Suppl 1, pp. 75–80, 2017, doi: 10.33140/aidt/01/01/00010.

[15] G. T. Leta *et al.*, "National mapping of soil-transmitted helminth and schistosome infections in Ethiopia," *Parasites and Vectors*, vol. 13, no. 1, pp. 1–13, 2020, doi: 10.1186/s13071-020-04317-6.

[16] R. Maddren *et al.*, "Soil-transmitted helminths and schistosome infections in Ethiopia: a systematic review of progress in their control over the past 20 years," 2021, *BioMed Central*. doi: 10.1186/s13071-021-04600-0.

[17] A. Terefe, T. Shimelis, M. Mengistu, A. Hailu, and B. Erko, "Schistosomiasis mansoni and soil-transmitted helminthiasis in Bushulo Village, Southern Ethiopia," *Ethiop. J. Heal. Dev.*, vol. 25, no. 1, pp. 46–50, 2011, doi: 10.4314/ejhd.v25i1.69847.

[18] B. Tadege and T. Shimelis, "Infections with Schistosoma mansoni and geohelminths among school children dwelling along the shore of the Lake Hawassa , southern Ethiopia," *PLoS One*, vol. 12, no. 7, pp. 1–12, 2017.

[19] J. Vercruysse *et al.*, "Monitoring Anthelmintic Efficacy for Soil Transmitted Helminths (STH)," *World Heal. Organ.*, pp. 1–64, 2008.

[20] A. Montresor *et al.*, "The global progress of soil-transmitted helminthiases control in 2020 and world health organization targets for 2030," *PLoS Negl. Trop. Dis.*, vol. 14, no. 8, pp. 1–17, 2020, doi: 10.1371/journal.pntd.0008505.

[21] M. S. Sousa *et al.*, "Performance of an Ultra-Sensitive Assay Targeting the Circulating Anodic Antigen (CAA) for Detection of Schistosoma mansoni Infection in a Low Endemic Area in Brazil," *Front. Immunol.*, vol. 10, pp. 1–16, 2019, doi: 10.3389/fimmu.2019.00682.

[22] C. G. Lindholz *et al.*, "Study of diagnostic accuracy of Helmintex , Kato-Katz , and POC-CCA methods for diagnosing intestinal schistosomiasis in Candeal , a low intensity transmission area in northeastern Brazil," *PLoS Negl. Trop. Dis.*, vol. 12, no. 3, pp. 1–16, 2018.

[23] A. Mueller, A. Fuss, U. Ziegler, G. M. Kaatano, and H. D. Mazigo, "Intestinal schistosomiasis of Ijinga Island , north-western Tanzania : prevalence , intensity of infection , hepatosplenic morbidities and their associated factors," *BMC Infect. Dis.*, vol. 19, no. 832, pp. 1–12, 2019.

[24] A. A. Haggag, A. Rabiee, K. M. A. Elaziz, C. H. Campbell, D. G. Colley, and R. M. R. Ramzy, "Thirty-Day Daily Comparisons of Kato – Katz and CCA Assays of 45 Egyptian Children in Areas with Very Low Prevalence of Schistosoma mansoni," *Am. Soc. Trop. Med. Hyg.*, vol. 100, no. 3, pp. 578–583, 2019, doi: 10.4269/ajtmh.18-0829.

[25] D. G. Colley *et al.*, "A five-country evaluation of a point-of-care circulating cathodic antigen urine assay for the prevalence of Schistosoma mansoni," *Am. J. Trop. Med. Hyg.*, vol. 88, no. 3, pp. 426–432, 2013, doi: 10.4269/ajtmh.12-0639.

[26] A. Straily *et al.*, "Evaluation of the Point-of-Care Circulating Cathodic Antigen Assay for Monitoring Mass Drug Administration in a Schistosoma mansoni Control Program in Western Kenya," *Am. Soc. Trop. Med. Hyg.*, vol. 106, no. 1, pp. 303–311, 2022, doi: 10.4269/ajtmh.21-0599.

[27] G. Bugssa, T. Teklehaymanot, G. Medhin, and N. Berhe, "Prevalence and intensity of Schistosoma

mansoni infection, and contributing factors in Alamata district of Tigray Region, Northern Ethiopia," *PLoS Negl. Trop. Dis.*, vol. 2024-Novem, no. 11, pp. 1–18, 2024, doi: 10.1371/journal.pntd.0012691.

[28] M. Casacuberta-Partal, P. T. Hoekstra, D. Kornelis, L. van Lieshout, and G. J. van Dam, "An innovative and user-friendly scoring system for standardised quantitative interpretation of the urine-based point-of-care strip test (POC-CCA) for the diagnosis of intestinal schistosomiasis: a proof-of-concept study," *Acta Trop.*, vol. 2, no. 17, pp. 1–4, 2019, doi: 10.1016/j.actatropica.2019.105150.

[29] C. Okoyo, E. Simiyu, S. M. Njenga, and C. Mwandawiro, "Comparing the performance of circulating cathodic antigen and Kato-Katz techniques in evaluating Schistosoma mansoni infection in areas with low prevalence in selected counties of Kenya: A cross-sectional study," *BMC Public Health*, vol. 18, no. 1, pp. 1–7, 2018, doi: 10.1186/s12889-018-5414-9.

[30] A. Tiruneh *et al.*, "Schistosoma mansoni Infections and Morbidities Among School Children in Hotspot Areas of Jimma Town, Southwest Ethiopia: A Cross-Sectional Study," *Environ. Health Insights*, vol. 17, pp. 1–8, 2023, doi: 10.1177/11786302231161047.

[31] H. Aliyi, M. Ahmed, T. Gobena, B. M. Alemu, H. A. Adem, and A. A. Usso, "Prevalence and factors associated with Schistosoma mansoni infection among primary school children in Kersa District, Eastern Ethiopia," *PeerJ*, vol. 12, no. 6, pp. 1–20, 2024, doi: 10.7717/peerj.17439.

[32] T. Ashenafi, D. Tadesse, and T. Zewdneh, "Infection prevalence of intestinal helminths and associated risk factors among schoolchildren in selected kebeles of Enderta district, Tigray, Northern Ethiopia," *J. Parasitol. Vector Biol.*, vol. 6, no. 11, pp. 166–173, 2014, doi: 10.5897/jpvb2014.0173.

[33] R. M. Ghazy *et al.*, "Determinants of Schistosoma mansoni transmission in hotspots at the late stage of elimination in Egypt," *Infect. Dis. Poverty*, vol. 11, no. 1, pp. 1–14, 2022, doi: 10.1186/s40249-022-01026-3.

[34] A. W. Bartlett *et al.*, "Burden and factors associated with schistosomiasis and soil ‐ transmitted helminth infections among school ‐ age children in Huambo , Uige and Zaire provinces , Angola," *Infect. Dis. Poverty*, vol. 11, no. 73, pp. 1–15, 2022, doi: 10.1186/s40249-022-00975-z.

[35] K. Wubet and D. Damtie, "Prevalence of Schistosoma mansoni Infection and Associated Risk Factors among School Children in Jiga Town , Northwest-Ethiopia : A Cross-Sectional Study," *J. Parasitol. Res.*, vol. 2020, pp. 1–7, 2020.

[36] B. Tazebew, D. Temesgen, M. Alehegn, and D. Salew, "Prevalence of S . mansoni Infection and Associated Risk Factors among School Children in Guangua District , Northwest Ethiopia," *J. Parasitol. Res.*, vol. 2022, pp. 1–9, 2022.

[37] M. G. Ansha, K. A. Kuti, and E. Girma, "Prevalence of Intestinal Schistosomiasis and Associated Factors among School Children in Wondo District , Ethiopia," *J. Trop. Med.*, vol. 10, pp. 1–8, 2020.

[38] A. et al Hailegebriel, Nibret, Munshea, "Prevalence , intensity and associated risk factors of Schistosoma mansoni infections among schoolchildren around Lake Tana , northwestern Ethiopia," *PLoS Negl. Trop. Dis.*, vol. 15, no. 10, pp. 1–16, 2021, doi: 10.1371/journal.pntd.0009861.

[39] B. Azalech Tefera, Belay, "Epidemiology of Schistosoma mansoni infection and associated risk

factors among school children attending primary schools nearby rivers in Jimma town , an urban setting , Southwest Ethiopia," *PLoS One*, vol. 15, no. 2, pp. 1–15, 2020, doi: 10.1371/journal.pone.0228007.

[40] A. J. Zeleke, A. Addisu, and Y. Tegegne, "Prevalence, Intensity, and Associated Factors of Schistosoma mansoni among School Children in Northwest Ethiopia," *J. Parasitol. Res.*, vol. 2020, pp. 1–7, 2020, doi: 10.1155/2020/8820222.

[41] A. Assefa, B. Erko, S. G. Gundersen, G. Medhin, and N. Berhe, "Current status of Schistosoma mansoni infection among previously treated rural communities in the Abbey and Didessa Valleys , Western Ethiopia : Implications for sustainable control," *PLoS One*, vol. 16, no. 2, pp. 1–15, 2021, doi: 10.1371/journal.pone.0247312.

[42] B. Alemayehu and Z. Tomass, "Schistosoma mansoni infection prevalence and associated risk factors among schoolchildren in Demba Girara, Damot Woide District of Wolaita Zone, Southern Ethiopia," *Asian Pac. J. Trop. Med.*, vol. 8, no. 6, pp. 457–463, 2015, doi: 10.1016/j.apjtm.2015.05.009.

[43] J. Masaku, N. Madigu, C. Okoyo, and S. M. Njenga, "Current status of Schistosoma mansoni and the factors associated with infection two years following mass drug administration programme among primary school children in Mwea irrigation scheme : A cross- sectional study," *BMC Public Health*, vol. 15, no. 739, pp. 1–9, 2015, doi: 10.1186/s12889-015-1991-z.

[44] M. M. Nigo, P. Odermatt, G. B. Salieb-Beugelaar, O. Morozov, M. Battegay, and P. R. Hunziker, "Epidemiology of schistosoma mansoni infection in Ituri province, North-Eastern democratic republic of the Congo," *PLoS Negl. Trop. Dis.*, vol. 15, no. 12, pp. 1–26, 2021, doi: 10.1371/journal.pntd.0009486.

[45] R. Hussein, M. Id, O. S. Minzi, S. M. Kinung, A. Kamuhabwa, and E. Aklillu, "Prevalence and correlates of intestinal schistosomiasis infection among school-aged children in North-Western Tanzania," *PLoS One*, vol. 15, no. 2, pp. 1–17, 2020.

[46] M. Aemero, N. Berhe, and B. Erko, "Status of Schistosoma mansoni prevalence and intensity of infection in geographically apart endemic localities of Ethiopia: a comparison," *Ethiop. J. Health Sci.*, vol. 24, no. 3, pp. 189–194, 2014, doi: 10.4314/ejhs.v24i3.1.

[47] M. Bajiro, D. Dana, and B. Levecke, "Prevalence and intensity of schistosoma mansoni infections among schoolchildren attending primary schools in an urban setting in southwest, ethiopia," *BMC Res. Notes*, vol. 10, no. 1, pp. 1–6, 2017, doi: 10.1186/s13104-017-3023-9.

[48] A. Montresor *et al.*, "12879_2019_Article_4730.pdf," *PLoS Negl. Trop. Dis.*, vol. 8, no. 8, pp. 1–8, 2020.

[49] K. Gass, D. G. Addiss, and M. C. Freeman, "Exploring the Relationship between Access to Water, Sanitation and Hygiene and Soil-Transmitted Helminth Infection: A Demonstration of Two Recursive Partitioning Tools," *PLoS Negl. Trop. Dis.*, vol. 8, no. 6, 2014, doi: 10.1371/journal.pntd.0002945.

[50] T. W. Jia, S. Melville, J. Utzinger, C. H. King, and X. N. Zhou, "Soil-transmitted helminth reinfection after drug treatment: A systematic review and meta-analysis," *PLoS Negl. Trop. Dis.*, vol. 6, no. 5, 2012, doi: 10.1371/journal.pntd.0001621.

[51] S. J. Brooker *et al.*, "Interrupting transmission of soiltransmitted helminths: A study protocol for

cluster randomised trials evaluating alternative treatment strategies and delivery systems in Kenya," *BMJ Open*, vol. 5, no. 10, pp. 1–12, 2015, doi: 10.1136/bmjopen-2015-008950.

[52] F. Giardina *et al.*, "Sampling strategies for monitoring and evaluation of morbidity targets for soil-transmitted helminths," *PLoS Negl. Trop. Dis.*, vol. 13, no. 6, pp. 1–17, 2019, doi: 10.1371/journal.pntd.0007514.

[53] A. Fenta *et al.*, "Prevalence and risks of soil transmitted helminths among Ethiopian school children: A cross-sectional study," *Asian Pac. J. Trop. Med.*, vol. 15, no. 3, pp. 121–127, 2022, doi: 10.4103/1995-7645.338438.

[54] F. Samuel, A. Demsew, Y. Alem, and Y. Hailesilassie, "Soil transmitted Helminthiasis and associated risk factors among elementary school children in ambo town, western Ethiopia," *BMC Public Health*, vol. 17, no. 1, pp. 1–7, 2017, doi: 10.1186/s12889-017-4809-3.

[55] B. Tolera *et al.*, "Prevalence, intensity, and associated factors of intestinal schistosomiasis among primary school children in Nono District, Southwest Ethiopia," *Front. Trop. Dis.*, vol. 5, no. February, pp. 1–11, 2024, doi: 10.3389/fitd.2024.1483164.

[56] G. Alemu, A. Abossie, and Z. Yohannes, "Current status of intestinal parasitic infections and associated factors among primary school children in Birbir town, Southern Ethiopia," *BMC Infect. Dis.*, vol. 19, no. 1, pp. 4–11, 2019, doi: 10.1186/s12879-019-3879-5.

[57] A. Zeynudin, T. Degefa, S. Suleman, A. Abamecha, Z. Hajikelil, and A. Wieser, "Prevalence and Determinants of Geohelminthiasis among School-Age Children in Jimma City, Ethiopia," *J. Trop. Med.*, vol. 2023, 2023, doi: 10.1155/2023/8811795.

[58] B. Bokicho, D. Hailu, B. Eshetu, M. Matie, and T. Tadele, "Soil-transmitted helminthiases among school-age children and their association with water, sanitation, and hygiene, Hawassa City, Southern Ethiopia," *PLoS Negl. Trop. Dis.*, vol. 17, no. 7, pp. 1–12, 2023, doi: 10.1371/journal.pntd.0011484.

[59] S. P. Luby, M. Agboatwalla, J. Painter, A. Altaf, W. L. Billhimer, and R. M. Hoekstra, "Effect of Intensive Handwashing Promotion on Childhood Diarrhea in High-Risk Communities in PakistanA Randomized Controlled Trial," *JAMA*, vol. 291, no. 21, pp. 2547–2554, Jun. 2004, doi: 10.1001/jama.291.21.2547.

[60] D. G. Feleke, A. Ali, H. Bisetegn, H. Debash, W. Birara, and A. Andualem, "Soil-transmitted helminthes and Schistosoma mansoni infections among primary school children at Ambasame primary school, North-West Ethiopia: a cross-sectional study," *BMC Pediatr.*, vol. 22, no. 1, pp. 1–7, 2022, doi: 10.1186/s12887-022-03534-5.

[61] A. O. Forson, I. Arthur, and P. F. Ayeh-Kumi, "The role of family size, employment and education of parents in the prevalence of intestinal parasitic infections in school children in Accra," *PLoS One*, vol. 13, no. 2, pp. 1–10, 2018, doi: 10.1371/journal.pone.0192303.

[62] R. W. Kihoro *et al.*, "Epidemiology of soil-transmitted helminthiasis among school-aged children in pastoralist communities of Kenya: A cross-sectional study," *PLoS One*, vol. 19, no. 5 MAY, pp. 1–20, 2024, doi: 10.1371/journal.pone.0304266.

[63] S. Debalke, A. Worku, N. Jahur, and Z. Mekonnen, "Soil transmitted helminths and associated factors among schoolchildren in government and private primary school in Jimma Town, Southwest Ethiopia," *Ethiop. J. Health Sci.*, vol. 23, no. 3, pp. 237–244, 2013, doi:

10.4314/ejhs.v23i3.6.

[64] A. Genet, A. Motbainor, T. Samuel, and M. Azage, "Prevalence and associated factors of soil transmitted helminthiasis among school-age children in wetland and non-wetland areas of Blue Nile Basins, northwest Ethiopia: A community-based comparative study," *SAGE Open Med.*, vol. 9, 2021, doi: 10.1177/20503121211063354.

[65] Y. Andargie, A. Alelign, and Z. Tekeste, "Prevalence and associated risk factors of soil-transmitted helminth infections among schoolchildren in Mekan Eyesus town, northwestern Ethiopia," *Parasite Epidemiol. Control*, vol. 27, no. March, p. e00379, 2024, doi: 10.1016/j.parepi.2024.e00379.

[66] S. Novianty, Y. Dimyati, S. Pasaribu, and A. P. Pasaribu, "Risk Factors for Soil-Transmitted Helminthiasis in Preschool Children Living in Farmland, North Sumatera, Indonesia," *J. Trop. Med.*, vol. 2018, 2018, doi: 10.1155/2018/6706413.

[67] C. Graeff-Teixeira *et al.*, "Low specificity of point-of-care circulating cathodic antigen (POC[sbnd]CCA) diagnostic test in a non-endemic area for schistosomiasis mansoni in Brazil," *Acta Trop.*, vol. 217, p. 7, 2021, doi: 10.1016/j.actatropica.2021.105863.

[68] A. Homsana, P. Odermatt, P. Southisavath, A. Yajima, and S. Sayasone, "Cross-reaction of POC-CCA urine test for detection of Schistosoma mekongi in Lao PDR: A cross-sectional study," *Infect. Dis. Poverty*, vol. 9, no. 1, pp. 1–9, 2020, doi: 10.1186/s40249-020-00733-z.

[69] A. Casagrande, F. Fabris, and R. Girometti, "Beyond kappa: an informational index for diagnostic agreement in dichotomous and multivalue ordered-categorical ratings," *Med. Biol. Eng. Comput.*, vol. 58, no. 12, pp. 3089–3099, 2020, doi: 10.1007/s11517-020-02261-2.

[1] World Health Organization. Schistosomiasis and soil-transmitted helminthiases: progress report, 2023. Wkly Epidemiol Rec. 2024;99:707-17.2021 Schistosomiasis and soil-transmitted helminthiases: progress report, "Weekly epidemiological record Relevé épidémiologique hebdomadaire," *Wkly. Epidemiol. Rec.*, vol. 97, no. 48, pp. 621–632, 2022.

[2] Exum NG, Kibira SP, Ssenyonga R, Nobili J, Shannon AK, Ssempebwa JC et al. The prevalence of schistosomiasis in Uganda: A nationally representative population estimate to inform control programs and water and sanitation interventions. PLoS neglected tropical diseases. 2019 Aug 14;13(8):e0007617.

Natalie G *et al.*, "The prevalence of schistosomiasis in Uganda : A nationally representative

Formatted: Normal, Line spacing: single

Formatted: Font: (Default) Calibri, 11 pt, Not Bold

Formatted: Space After: 8 pt, Line spacing: Multiple 1.08 li, Widow/Orphan control, Adjust space between Latin and Asian text, Adjust space between Asian text and numbers

~~population estimate to inform control programs and water and sanitation interventions," *PLoS Negl. Trop. Dis.*, vol. 13, no. 8, pp. 1–21, 2019.~~

~~[~~3~~]~~ Hussen S, Assegu D, Tadesse BT, Shimelis T. Prevalence of Schistosoma mansoni infection in Ethiopia: a systematic review and meta-analysis. Tropical diseases, travel medicine and vaccines. 2021 Feb 1;7(1):4.~~S. Hussen, D. Assegu, B. T. Tadesse, and T. Shimelis, "Prevalence of Schistosoma mansoni infection in Ethiopia : a systematic review and meta-analysis," *Trop. Dis. Travel Med. Vaccines*, vol. 7, no. 4, pp. 1–12, 2021.~~

~~[~~4~~]~~ World Health Organization. WHO guideline on control and elimination of human schistosomiasis. World Health Organization; 2022 Feb 14. https://www.who.int/publications/i/item/9789240041608

~~WHO, *WHO GUIDELINE on control and elimination of human schistosomiasis*, vol. 3, no. 2. 2022. [Online]. Available: http://dx.doi.org/10.1016/j.xpro.2020.100074%0Ahttp://dx.doi.org/10.1074/mcp.R115.052902%0Ahttp://dx.doi.org/10.1016/S0140-6736(13)61949-2%0Ahttp://dx.doi.org/10.1016/j.bbagen.2016.09.015~~

~~[~~5~~]~~ Ismail HA, Hong ST, Babiker AT, Hassan RM, Sulaiman MA, Jeong HG et al. Prevalence, risk factors, and clinical manifestations of schistosomiasis among school children in the White Nile River basin, Sudan. Parasites & vectors. 2014 Oct 15;7(1):478.~~H. Ahmed *et al.*, "Prevalence , risk factors , and clinical manifestations of schistosomiasis among school children in the White Nile River basin , Sudan," *Parasit. Vectors*, vol. 7, no. 1, pp. 1–11, 2014.~~

~~[~~6~~]~~ Lo NC, Bezerra FS, Colley DG, Fleming FM, Homeida M, Kabatereine N, Kabole FM, King CH, Mafe MA, Midzi N, Mutapi F. Review of 2022 WHO guidelines on the control

and elimination of schistosomiasis. The Lancet Infectious Diseases. 2022 Nov 1;22(11):e327-35.

Nathan *et al.*, “Review of 2022 WHO guidelines on the control and elimination of schistosomiasis,” *Lancet Infect. Dis.*, vol. 22, no. 11, pp. e327–e335, 2022, doi: 10.1016/S1473-3099(22)00221-3.

[7] Dahal AS, Francis EO, Francis JE, Wamtas FI. Soil-transmitted helminths and associated risk factors among elementary school pupils in Dadin Kowa, Jos. Nigerian Medical Journal. 2019 Jul 1;60(4):181-5. doi: 10.4103/nmj.nmj_62_19.

A. Dahal, E. Francis, J. Francis, and F. Wamtas, “Soil-transmitted helminths and associated risk factors among elementary school pupils in Dadin Kowa, Jos,” *Niger. Med. J.*, vol. 60, no. 4, p. 181, 2019, doi: 10.4103/nmj.nmj_62_19.

[8] Getaneh M, Hailegebriel T, Munshea A, Nibret E. Prevalence and Associated Risk Factors of Soil-Transmitted Helminth Infections Among Schoolchildren Around Lake Tana, Northwest Ethiopia. Journal of Parasitology Research. 2022;2022(1):4603638.

M. Getaneh, T. Hailegebriel, and A. Munshea, “Prevalence and Associated Risk Factors of Soil-Transmitted Helminth Infections among Schoolchildren around Lake Tana , Northwest Ethiopia,” *J. Parasitol. Res.*, vol. 2022, p. 13, 2022.

[9] Wale M, Gedefaw S. Prevalence of intestinal Protozoa and soil transmitted helminths infections among school children in Jaragedo town, South Gondar zone of Ethiopia. Journal of Tropical Medicine. 2022;2022(1):5747978.

M. Wale and S. Gedefaw, “Prevalence of Intestinal Protozoa and Soil Transmitted Helminths Infections among School Children in Jaragedo Town, South Gondar Zone of Ethiopia,” *J. Trop. Med.*, vol. 2022, p. 11, 2022, doi: 10.1155/2022/5747978.

[10] Beyene B, Vaz Nery S, Lambiyo T, Shimelis T. Schistosoma mansoni and soil-transmitted helminth infections among schoolchildren living along the shore of Lake Hawassa, southern Ethiopia. Parasites & Vectors. 2024 Dec 3;17(1):498.

B. Beyene, S. Vaz Nery, T. Lambiyo, and T. Shimelis, "Schistosoma mansoni and soil-transmitted helminth infections among schoolchildren living along the shore of Lake Hawassa, southern Ethiopia," *Parasites and Vectors* , vol. 17, no. 1, pp. 1–14, 2024, doi: 10.1186/s13071-024-06578-x.

[11] Pullan RL, Smith JL, Jasrasaria R, Brooker SJ. Global numbers of infection and disease burden of soil transmitted helminth infections in 2010. Parasites & vectors. 2014 Jan 21;7(1):37, doi: 10.1186/1756-3305-7-37.

R. L. Pullan, J. L. Smith, R. Jasrasaria, and S. J. Brooker, "Global numbers of infection and disease burden of soil transmitted helminth infections in 2010," *Parasites and Vectors*, vol. 7, no. 1, pp. 1–19, 2014, doi: 10.1186/1756-3305-7-37.

[12] Östan İ, Kilimcioğlu AA, Girginkardeşler N, Özyurt BC, Limoncu ME, Ok ÜZ. Health inequities: lower socio-economic conditions and higher incidences of intestinal parasites. BMC public health. 2007 Nov 27;7(1):342 , doi: 10.1186/1471-2458-7-342.

I. Östan, A. A. Kilimcioğlu, N. Girginkardeşler, B. C. Özyurt, M. E. Limoncu, and Ü. Z. Ok, "Health inequities: Lower socio-economic conditions and higher incidences of intestinal parasites," *BMC Public Health*, vol. 7, pp. 1–8, 2007, doi: 10.1186/1471-2458-7-342.

[13] N. R. De Silva, S. Brooker, P. J. Hotez, A. Montresor, D. Engels, and L. Savioli,. Soil-transmitted helminth infections: Updating the global picture. *Trends Parasitol.*, vol. 19, no. 12, pp. 547–551, 2003, doi: 10.1016/j.pt.2003.10.002.

N. R. De Silva, S. Brooker, P. J. Hotez, A. Montresor, D. Engels, and L. Savioli, "Soil-

transmitted helminth infections: Updating the global picture," *Trends Parasitol.*, vol. 19, no. 12, pp. 547–551, 2003, doi: 10.1016/j.pt.2003.10.002.

[14] World Health Organization. Schistosomiasis and soil-transmitted helminthiases: progress report, 2021. Wkly Epidemiol Rec. 2022;97(48):621-32.N. Negussu *et al.*, "Ethiopia schistosomiasis and soil-transmitted helminthes control programme: Progress and prospects," *Ethiop. Med. J.*, vol. 55, no. Suppl 1, pp. 75–80, 2017, doi: 10.33140/aidt/01/01/00010.

[15] Rayment Gomez S, Maddren R, Liyew EF, Chernet M, Anjulo U, Tamiru A et al. Predisposition to soil-transmitted helminth reinfection after four rounds of mass drug administration: results from a longitudinal cohort in the Geshiyaro project, a transmission elimination feasibility study in the Wolaita zone of southern Ethiopia. Transactions of The Royal Society of Tropical Medicine and Hygiene. 2023 Jul;117(7):514-21.

G. T. Leta *et al.*, "National mapping of soil-transmitted helminth and schistosome infections in Ethiopia," *Parasites and Vectors*, vol. 13, no. 1, pp. 1–13, 2020, doi: 10.1186/s13071-020-04317-6.

[16] Negussu N, Mengistu B, Kebede B, Deribe K, Ejigu E, Tadesse G, Mekete K, Sileshi M. Ethiopia schistosomiasis and soil-transmitted helminthes control programme: progress and prospects. Ethiopian medical journal. 2017;55(Suppl 1):75.R. Maddren *et al.*, "Soil-transmitted helminths and schistosome infections in Ethiopia: a systematic review of progress in their control over the past 20 years," 2021, *BioMed Central*. doi: 10.1186/s13071-021-04600-0.

[17] Leta GT, Mekete K, Wuletaw Y, Gebretsadik A, Sime H, Mekasha S, Woyessa A, Shafi O, Vercruysse J, Grimes JE, Gardiner I. National mapping of soil-transmitted helminth and

schistosome infections in Ethiopia. Parasites & vectors. 2020 Sep 1;13(1):437.

A. Terefe, T. Shimelis, M. Mengistu, A. Hailu, and B. Erko, "Schistosomiasis mansoni and soil-transmitted helminthiasis in Bushulo Village, Southern Ethiopia," *Ethiop. J. Heal. Dev.*, vol. 25, no. 1, pp. 46–50, 2011, doi: 10.4314/ejhd.v25i1.69847.

[18] Maddren R, Phillips A, Ower A, Landeryou T, Mengistu B, Anjulo U, Firdawek E, Negussu N, Anderson R. Soil-transmitted helminths and schistosome infections in Ethiopia: a systematic review of progress in their control over the past 20 years. Parasites & vectors. 2021 Feb 5;14(1):97.

B. Tadege and T. Shimelis, "Infections with Schistosoma mansoni and geohelminths among school children dwelling along the shore of the Lake Hawassa , southern Ethiopia," *PLoS One*, vol. 12, no. 7, pp. 1–12, 2017.

[19] Terefe A, Shimelis T, Mengistu M, Hailu A, Erko B. Schistosomiasis mansoni and soil-transmitted helminthiasis in Bushulo village, southern Ethiopia. Ethiopian Journal of Health Development. 2011;25(1):46-50. J. Vercruysse *et al.*, "Monitoring Anthelmintic Efficacy for Soil Transmitted Helminths (STH)," *World Heal. Organ.*, pp. 1–64, 2008.

[20] Tadege B, Shimelis T. Infections with Schistosoma mansoni and geohelminths among school children dwelling along the shore of the Lake Hawassa, southern Ethiopia. PloS one. 2017 Jul 18;12(7):e0181547.

A. Montresor *et al.*, "The global progress of soil-transmitted helminthiases control in 2020 and world health organization targets for 2030," *PLoS Negl. Trop. Dis.*, vol. 14, no. 8, pp. 1–17, 2020, doi: 10.1371/journal.pntd.0008505.

[21] Sousa MS, Van Dam GJ, Pinheiro MC, De Dood CJ, Peralta JM, Peralta RH, et al.. Performance of an ultra-sensitive assay targeting the circulating anodic antigen (CAA) for

detection of Schistosoma mansoni infection in a low endemic area in Brazil. Frontiers in immunology. 2019 Apr 4;10:682.

M. S. Sousa *et al.*, "Performance of an Ultra-Sensitive Assay Targeting the Circulating Anodic Antigen (CAA) for Detection of Schistosoma mansoni Infection in a Low Endemic Area in Brazil," *Front. Immunol.*, vol. 10, pp. 1–16, 2019, doi: 10.3389/fimmu.2019.00682.

[22] Lindholz CG, Favero V, Verissimo CD, Candido RR, de Souza RP, Dos Santos RR, et al. Study of diagnostic accuracy of Helmintex, Kato-Katz, and POC-CCA methods for diagnosing intestinal schistosomiasis in Candeal, a low intensity transmission area in northeastern Brazil. PLoS Neglected Tropical Diseases. 2018 Mar 8;12(3):e0006274.

C. G. Lindholz *et al.*, "Study of diagnostic accuracy of Helmintex , Kato-Katz , and POC-CCA methods for diagnosing intestinal schistosomiasis in Candeal , a low intensity transmission area in northeastern Brazil," *PLoS Negl. Trop. Dis.*, vol. 12, no. 3, pp. 1–16, 2018.

[23] Mueller A, Fuss A, Ziegler U, Kaatano GM, Mazigo HD. Intestinal schistosomiasis of Ijinga Island, north-western Tanzania: prevalence, intensity of infection, hepatosplenic morbidities and their associated factors. BMC infectious diseases. 2019 Oct 7;19(1):832.A. Mueller, A. Fuss, U. Ziegler, G. M. Kaatano, and H. D. Mazigo, "Intestinal schistosomiasis of Ijinga Island , north-western Tanzania : prevalence , intensity of infection , hepatosplenic morbidities and their associated factors," *BMC Infect. Dis.*, vol. 19, no. 832, pp. 1–12, 2019.

[24] Haggag AA, Rabiee A, Abd Elaziz KM, Campbell Jr CH, Colley DG, Ramzy RM. Thirty-day daily comparisons of Kato–Katz and CCA assays of 45 Egyptian children in areas with very low prevalence of Schistosoma mansoni. The American journal of tropical medicine and hygiene. 2019 Jan 2;100(3):578.

A. A. Haggag, A. Rabiee, K. M. A. Elaziz, C. H. Campbell, D. G. Colley, and R. M. R. Ramzy, "Thirty-Day Daily Comparisons of Kato – Katz and CCA Assays of 45 Egyptian Children in Areas with Very Low Prevalence of Schistosoma mansoni," *Am. Soc. Trop. Med. Hyg.*, vol. 100, no. 3, pp. 578–583, 2019, doi: 10.4269/ajtmh.18-0829.

[25] Colley DG, Binder S, Campbell C, King CH, Tchuenté LA, N'Goran EK et al. A five-country evaluation of a point-of-care circulating cathodic antigen urine assay for the prevalence of Schistosoma mansoni. The American journal of tropical medicine and hygiene. 2013 Mar 6;88(3):426.

D. G. Colley *et al.*, "A five-country evaluation of a point-of-care circulating cathodic antigen urine assay for the prevalence of Schistosoma mansoni," *Am. J. Trop. Med. Hyg.*, vol. 88, no. 3, pp. 426–432, 2013, doi: 10.4269/ajtmh.12-0639.

[26] Straily A, Kavere EA, Wanja D, Wiegand RE, Montgomery SP, Mwaki A, et al. Evaluation of the point-of-care circulating cathodic antigen assay for monitoring mass drug administration in a Schistosoma mansoni control program in Western Kenya. The American Journal of Tropical Medicine and Hygiene. 2021 Nov 8;106(1):303.

A. Straily *et al.*, "Evaluation of the Point-of-Care Circulating Cathodic Antigen Assay for Monitoring Mass Drug Administration in a Schistosoma mansoni Control Program in Western Kenya," *Am. Soc. Trop. Med. Hyg.*, vol. 106, no. 1, pp. 303–311, 2022, doi: 10.4269/ajtmh.21-0599.

[27] Bugssa G, Teklehaymanot T, Medhin G, Berhe N. Prevalence and intensity of Schistosoma mansoni infection, and contributing factors in Alamata district of Tigray Region, Northern Ethiopia. PLOS Neglected Tropical Diseases. 2024 Nov 25;18(11):e0012691.

G. Bugssa, T. Teklehaymanot, G. Medhin, and N. Berhe, "Prevalence and intensity of Schistosoma mansoni infection, and contributing factors in Alamata district of Tigray Region, Northern Ethiopia," *PLoS Negl. Trop. Dis.*, vol. 2024-Novem, no. 11, pp. 1–18, 2024, doi: 10.1371/journal.pntd.0012691.

[28] Casacuberta-Partal M, Hoekstra PT, Kornelis D, van Lieshout L, van Dam GJ. An innovative and user-friendly scoring system for standardised quantitative interpretation of the urine-based point-of-care strip test (POC-CCA) for the diagnosis of intestinal schistosomiasis: a proof-of-concept study. Acta Tropica. 2019 Nov 1;199:105150.

M. Casacuberta-Partal, P. T. Hoekstra, D. Kornelis, L. van Lieshout, and G. J. van Dam, "An innovative and user-friendly scoring system for standardised quantitative interpretation of the urine-based point-of-care strip test (POC-CCA) for the diagnosis of intestinal schistosomiasis: a proof-of-concept study," *Acta Trop.*, vol. 2, no. 17, pp. 1–4, 2019, doi: 10.1016/j.actatropica.2019.105150.

[29] Okoyo C, Simiyu E, Njenga SM, Mwandawiro C. Comparing the performance of circulating cathodic antigen and Kato-Katz techniques in evaluating Schistosoma mansoni infection in areas with low prevalence in selected counties of Kenya: a cross-sectional study. BMC Public Health. 2018 Apr 11;18(1):478.

C. Okoyo, E. Simiyu, S. M. Njenga, and C. Mwandawiro, "Comparing the performance of circulating cathodic antigen and Kato-Katz techniques in evaluating Schistosoma mansoni infection in areas with low prevalence in selected counties of Kenya: A cross-sectional study," *BMC Public Health*, vol. 18, no. 1, pp. 1–7, 2018, doi: 10.1186/s12889-018-5414-9.

[30] Tiruneh A, Zemene E, Abdissa Mizana B, Girma H, Dereje E, Sharew B et al.

Schistosoma mansoni infections and morbidities among School Children in Hotspot areas of Jimma Town, Southwest Ethiopia: a cross-sectional study. Environmental Health Insights. 2023 Mar;17:11786302231161047.

~~A. Tiruneh *et al.*, "Schistosoma mansoni Infections and Morbidities Among School Children in Hotspot Areas of Jimma Town, Southwest Ethiopia: A Cross-Sectional Study," *Environ. Health Insights*, vol. 17, pp. 1–8, 2023, doi: 10.1177/11786302231161047.~~

~~[~~31~~]~~ Aliyi H, Ahmed M, Gobena T, Alemu BM, Adem HA, Usso AA. Prevalence and factors associated with Schistosoma mansoni infection among primary school children in Kersa District, Eastern Ethiopia. PeerJ. 2024 Jun 14;12:e17439.

~~H. Aliyi, M. Ahmed, T. Gobena, B. M. Alemu, H. A. Adem, and A. A. Usso, "Prevalence and factors associated with Schistosoma mansoni infection among primary school children in Kersa District, Eastern Ethiopia," *PeerJ*, vol. 12, no. 6, pp. 1–20, 2024, doi: 10.7717/peerj.17439.~~

~~[~~32~~]~~ Teklemariam A, Dejenie T, Tomass Z. Infection prevalence of intestinal helminths and associated risk factors among schoolchildren in selected kebeles of Enderta district, Tigray, Northern Ethiopia. J Parasitol Vector Biol. 2014;6(11):166-73.

~~T. Ashenafi, D. Tadesse, and T. Zewdneh, "Infection prevalence of intestinal helminths and associated risk factors among schoolchildren in selected kebeles of Enderta district, Tigray, Northern Ethiopia," *J. Parasitol. Vector Biol.*, vol. 6, no. 11, pp. 166–173, 2014, doi: 10.5897/jpvb2014.0173.~~

~~[~~33~~]~~ Ghazy RM, Ellakany WI, Badr MM, Taktak NE, Elhadad H, Abdo SM, et al. Determinants of Schistosoma mansoni transmission in hotspots at the late stage of elimination in Egypt. Infectious Diseases of Poverty. 2022 Sep 23;11(1):102.~~R. M. Ghazy~~

~~*et al.*, "Determinants of Schistosoma mansoni transmission in hotspots at the late stage of elimination in Egypt," *Infect. Dis. Poverty*, vol. 11, no. 1, pp. 1–14, 2022, doi: 10.1186/s40249-022-01026-3.~~

[34] Bartlett AW, Sousa-Figueiredo JC, van Goor RC, Monaghan P, Lancaster W, Mugizi R, et al. Burden and factors associated with schistosomiasis and soil-transmitted helminth infections among school-age children in Huambo, Uige and Zaire provinces, Angola. Infectious Diseases of Poverty. 2022 Jun 25;11(1):73.

~~A. W. Bartlett *et al.*, "Burden and factors associated with schistosomiasis and soil – transmitted helminth infections among school – age children in Huambo , Uige and Zaire provinces , Angola," *Infect. Dis. Poverty*, vol. 11, no. 73, pp. 1–15, 2022, doi: 10.1186/s40249-022-00975-z.~~

[35] Wubet K, Damtie D. Prevalence of Schistosoma mansoni Infection and Associated Risk Factors among School Children in Jiga Town, Northwest-Ethiopia: A Cross-Sectional Study. Journal of Parasitology Research. 2020;2020(1):6903912.~~K. Wubet and D. Damtie, "Prevalence of Schistosoma mansoni Infection and Associated Risk Factors among School Children in Jiga Town , Northwest-Ethiopia : A Cross-Sectional Study," *J. Parasitol. Res.*, vol. 2020, pp. 1–7, 2020.~~

[36] Tazebew B, Temesgen D, Alehegn M, Salew D, Tarekegn M. Prevalence of S. mansoni infection and associated risk factors among school children in Guangua District, Northwest Ethiopia. Journal of Parasitology Research. 2022;2022(1):1005637. ~~B. Tazebew, D. Temesgen, M. Alehegn, and D. Salew, "Prevalence of S . mansoni Infection and Associated Risk Factors among School Children in Guangua District , Northwest Ethiopia," *J. Parasitol. Res.*, vol. 2022, pp. 1–9, 2022.~~

[37] Ansha MG, Kuti KA, Girma E. Prevalence of intestinal schistosomiasis and associated factors among school children in Wondo District, Ethiopia. Journal of tropical medicine. 2020;2020(1):9813743. M. G. Ansha, K. A. Kuti, and E. Girma, "Prevalence of Intestinal Schistosomiasis and Associated Factors among School Children in Wondo District , Ethiopia," *J. Trop. Med.*, vol. 10, pp. 1–8, 2020.

[38] Hailegebriel T, Nibret E, Munshea A, Ameha Z. Prevalence, intensity and associated risk factors of Schistosoma mansoni infections among schoolchildren around Lake Tana, northwestern Ethiopia. PLoS Neglected Tropical Diseases. 2021 Oct 27;15(10):e0009861.

A. et al Hailegebriel, Nibret, Munshea, "Prevalence , intensity and associated risk factors of Schistosoma mansoni infections among schoolchildren around Lake Tana , northwestern Ethiopia," *PLoS Negl. Trop. Dis.*, vol. 15, no. 10, pp. 1–16, 2021, doi: 10.1371/journal.pntd.0009861.

[39] Tefera A, Belay T, Bajiro M. Epidemiology of Schistosoma mansoni infection and associated risk factors among school children attending primary schools nearby rivers in Jimma town, an urban setting, Southwest Ethiopia. PloS one. 2020 Feb 27;15(2):e0228007. B. Azalech Tefera, Belay, "Epidemiology of Schistosoma mansoni infection and associated risk factors among school children attending primary schools nearby rivers in Jimma town , an urban setting , Southwest Ethiopia," *PLoS One*, vol. 15, no. 2, pp. 1–15, 2020, doi: 10.1371/journal.pone.0228007.

[40] Zeleke AJ, Addisu A, Tegegne Y. Prevalence, intensity, and associated factors of Schistosoma mansoni among school children in Northwest Ethiopia. Journal of Parasitology Research. 2020;2020(1):8820222.A. J. Zeleke, A. Addisu, and Y. Tegegne, "Prevalence, Intensity, and Associated Factors of Schistosoma mansoni among School

Children in Northwest Ethiopia," *J. Parasitol. Res.*, vol. 2020, pp. 1–7, 2020, doi: 10.1155/2020/8820222.

[41] Assefa A, Erko B, Gundersen SG, Medhin G, Berhe N. Current status of Schistosoma mansoni infection among previously treated rural communities in the Abbey and Didessa Valleys, Western Ethiopia: Implications for sustainable control. Plos one. 2021 Feb 25;16(2):e0247312.

A. Assefa, B. Erko, S. G. Gundersen, G. Medhin, and N. Berhe, "Current status of Schistosoma mansoni infection among previously treated rural communities in the Abbey and Didessa Valleys , Western Ethiopia : Implications for sustainable control," *PLoS One*, vol. 16, no. 2, pp. 1–15, 2021, doi: 10.1371/journal.pone.0247312.

[42] Alemayehu B, Tomass Z. Schistosoma mansoni infection prevalence and associated risk factors among schoolchildren in Demba Girara, Damot Woide District of Wolaita Zone, Southern Ethiopia. Asian Pacific journal of tropical medicine. 2015 Jun 1;8(6):457-63.

B. Alemayehu and Z. Tomass, "Schistosoma mansoni infection prevalence and associated risk factors among schoolchildren in Demba Girara, Damot Woide District of Wolaita Zone, Southern Ethiopia," *Asian Pac. J. Trop. Med.*, vol. 8, no. 6, pp. 457–463, 2015, doi: 10.1016/j.apjtm.2015.05.009.

[43] Masaku J, Madigu N, Okoyo C, Njenga SM. Current status of Schistosoma mansoni and the factors associated with infection two years following mass drug administration programme among primary school children in Mwea irrigation scheme: A cross-sectional study. BMC Public Health. 2015 Aug 1;15(1):739.

J. Masaku, N. Madigu, C. Okoyo, and S. M. Njenga, "Current status of Schistosoma mansoni and the factors associated with infection two years following mass drug administration

programme among primary school children in Mwea irrigation scheme : A cross- sectional study," *BMC Public Health*, vol. 15, no. 739, pp. 1–9, 2015, doi: 10.1186/s12889-015-1991-z.

[44] Nigo MM, Odermatt P, Salieb–Beugelaar GB, Morozov O, Battegay M, Hunziker PR. Epidemiology of Schistosoma mansoni infection in Ituri Province, north-eastern Democratic Republic of the Congo. PLoS Neglected Tropical Diseases. 2021 Dec 2;15(12):e0009486.

M. M. Nigo, P. Odermatt, G. B. Salieb-Beugelaar, O. Morozov, M. Battegay, and P. R. Hunziker, "Epidemiology of schistosoma mansoni infection in Ituri province, North-Eastern democratic republic of the Congo," *PLoS Negl. Trop. Dis.*, vol. 15, no. 12, pp. 1–26, 2021, doi: 10.1371/journal.pntd.0009486.

[45] Mnkugwe RH, Minzi OS, Kinung'hi SM, Kamuhabwa AA, Aklillu E. Prevalence and correlates of intestinal schistosomiasis infection among school-aged children in North-Western Tanzania. PloS one. 2020 Feb 5;15(2):e0228770.

R. Hussein, M. Id, O. S. Minzi, S. M. Kinung, A. Kamuhabwa, and E. Aklillu, "Prevalence and correlates of intestinal schistosomiasis infection among school-aged children in North-Western Tanzania," *PLoS One*, vol. 15, no. 2, pp. 1–17, 2020.

[46] Aemero M, Berhe N, Erko B. Status of Schistosoma mansoni prevalence and intensity of infection in geographically apart endemic localities of Ethiopia: a comparison. Ethiopian journal of health sciences. 2014 Jul 31;24(3):189-94.

M. Aemero, N. Berhe, and B. Erko, "Status of Schistosoma mansoni prevalence and intensity of infection in geographically apart endemic localities of Ethiopia: a comparison," *Ethiop. J. Health Sci.*, vol. 24, no. 3, pp. 189–194, 2014, doi: 10.4314/ejhs.v24i3.1.

[47] Bajiro M, Dana D, Levecke B. Prevalence and intensity of Schistosoma mansoni infections among schoolchildren attending primary schools in an urban setting in Southwest, Ethiopia. BMC research notes. 2017 Dec 4;10(1):677.

M. Bajiro, D. Dana, and B. Levecke, "Prevalence and intensity of schistosoma mansoni infections among schoolchildren attending primary schools in an urban setting in southwest, ethiopia," *BMC Res. Notes*, vol. 10, no. 1, pp. 1–6, 2017, doi: 10.1186/s13104-017-3023-9.

[48] Montresor A, Mupfasoni D, Mikhailov A, Mwinzi P, Lucianez A, Jamsheed M, et al. The global progress of soil-transmitted helminthiases control in 2020 and World Health Organization targets for 2030. PLoS neglected tropical diseases. 2020 Aug 10;14(8):e0008505.

A. Montresor *et al.*, "12879_2019_Article_4730.pdf," *PLoS Negl. Trop. Dis.*, vol. 8, no. 8, pp. 1–8, 2020.

[49] Gass K, Addiss DG, Freeman MC. Exploring the relationship between access to water, sanitation and hygiene and soil-transmitted helminth infection: a demonstration of two recursive partitioning tools. PLoS neglected tropical diseases. 2014 Jun 12;8(6):e2945.

K. Gass, D. G. Addiss, and M. C. Freeman, "Exploring the Relationship between Access to Water, Sanitation and Hygiene and Soil-Transmitted Helminth Infection: A Demonstration of Two Recursive Partitioning Tools," *PLoS Negl. Trop. Dis.*, vol. 8, no. 6, 2014, doi: 10.1371/journal.pntd.0002945.

[50] Jia TW, Melville S, Utzinger J, King CH, Zhou XN. Soil-transmitted helminth reinfection after drug treatment: a systematic review and meta-analysis. PLoS neglected tropical diseases. 2012 May 8;6(5):e1621.

T. W. Jia, S. Melville, J. Utzinger, C. H. King, and X. N. Zhou, "Soil-transmitted helminth reinfection after drug treatment: A systematic review and meta-analysis," *PLoS Negl. Trop. Dis.*, vol. 6, no. 5, 2012, doi: 10.1371/journal.pntd.0001621.

[51] Brooker SJ, Mwandawiro CS, Halliday KE, Njenga SM, Mcharo C, Gichuki PM, Wasunna B, Kihara JH, Njomo D, Alusala D, Chiguzo A. Interrupting transmission of soil-transmitted helminths: a study protocol for cluster randomised trials evaluating alternative treatment strategies and delivery systems in Kenya. BMJ open. 2015 Oct 1;5(10):e008950.

S. J. Brooker *et al.*, "Interrupting transmission of soiltransmitted helminths: A study protocol for cluster randomised trials evaluating alternative treatment strategies and delivery systems in Kenya," *BMJ Open*, vol. 5, no. 10, pp. 1–12, 2015, doi: 10.1136/bmjopen-2015-008950.

[52] Giardina F, Coffeng LE, Farrell SH, Vegvari C, Werkman M, Truscott JE et al. Sampling strategies for monitoring and evaluation of morbidity targets for soil-transmitted helminths. PLoS neglected tropical diseases. 2019 Jun 26;13(6):e0007514.

F. Giardina *et al.*, "Sampling strategies for monitoring and evaluation of morbidity targets for soil-transmitted helminths," *PLoS Negl. Trop. Dis.*, vol. 13, no. 6, pp. 1–17, 2019, doi: 10.1371/journal.pntd.0007514.

[53] Fenta A, Mesganaw B, Belachew F, Teshale G, Abebaw G, Tesfa E, Alemayehu G. Prevalence and risks of soil transmitted helminths among Ethiopian school children: A cross-sectional study. Asian Pacific Journal of Tropical Medicine. 2022 Mar 1;15(3):121-7.

A. Fenta *et al.*, "Prevalence and risks of soil transmitted helminths among Ethiopian school

children: A cross-sectional study," *Asian Pac. J. Trop. Med.*, vol. 15, no. 3, pp. 121–127, 2022, doi: 10.4103/1995-7645.338438.

[54] Samuel F, Demsew A, Alem Y, Hailesilassie Y. Soil transmitted Helminthiasis and associated risk factors among elementary school children in ambo town, western Ethiopia. BMC Public Health. 2017 Oct 10;17(1):791.

F. Samuel, A. Demsew, Y. Alem, and Y. Hailesilassie, "Soil transmitted Helminthiasis and associated risk factors among elementary school children in ambo town, western Ethiopia," *BMC Public Health*, vol. 17, no. 1, pp. 1–7, 2017, doi: 10.1186/s12889-017-4809-3.

[55] Tolera B, Mohammed J, Umer U, Abamecha J, Tebeje F, Sime A, Weldegebreal F. Prevalence, intensity, and associated factors of intestinal schistosomiasis among primary school children in Nono District, Southwest Ethiopia. Frontiers in Tropical Diseases. 2025 Feb 18;5:1483164.

B. Tolera *et al.*, "Prevalence, intensity, and associated factors of intestinal schistosomiasis among primary school children in Nono District, Southwest Ethiopia," *Front. Trop. Dis.*, vol. 5, no. February, pp. 1–11, 2024, doi: 10.3389/fitd.2024.1483164.

[56] Alemu G, Abossie A, Yohannes Z. Current status of intestinal parasitic infections and associated factors among primary school children in Birbir town, Southern Ethiopia. BMC infectious diseases. 2019 Mar 19;19(1):270.

G. Alemu, A. Abossie, and Z. Yohannes, "Current status of intestinal parasitic infections and associated factors among primary school children in Birbir town, Southern Ethiopia," *BMC Infect. Dis.*, vol. 19, no. 1, pp. 4–11, 2019, doi: 10.1186/s12879-019-3879-5.

[57] Zeynudin A, Degefa T, Suleman S, Abamecha A, Hajikelil Z, Wieser A. Prevalence and

Determinants of Geohelminthiasis among School-Age Children in Jimma City, Ethiopia. Journal of Tropical Medicine. 2023;2023(1):8811795.

A. Zeynudin, T. Degefa, S. Suleman, A. Abamecha, Z. Hajikelil, and A. Wieser, "Prevalence and Determinants of Geohelminthiasis among School-Age Children in Jimma City, Ethiopia," *J. Trop. Med.*, vol. 2023, 2023, doi: 10.1155/2023/8811795.

[58] Bokicho B, Hailu D, Eshetu B, Matie M, Tadele T. Soil-transmitted helminthiases among school-age children and their association with water, sanitation, and hygiene, Hawassa City, Southern Ethiopia. PLoS Neglected Tropical Diseases. 2023 Jul 28;17(7):e0011484.

B. Bokicho, D. Hailu, B. Eshetu, M. Matie, and T. Tadele, "Soil-transmitted helminthiases among school-age children and their association with water, sanitation, and hygiene, Hawassa City, Southern Ethiopia," *PLoS Negl. Trop. Dis.*, vol. 17, no. 7, pp. 1–12, 2023, doi: 10.1371/journal.pntd.0011484.

[59] Luby SP, Agboatwalla M, Painter J, Altaf A, Billhimer WL, Hoekstra RM. Effect of intensive handwashing promotion on childhood diarrhea in high-risk communities in Pakistan: a randomized controlled trial. Jama. 2004 Jun 2;291(21):2547-54.

S. P. Luby, M. Agboatwalla, J. Painter, A. Altaf, W. L. Billhimer, and R. M. Hoekstra, "Effect of Intensive Handwashing Promotion on Childhood Diarrhea in High-Risk Communities in PakistanA Randomized Controlled Trial," *JAMA*, vol. 291, no. 21, pp. 2547–2554, Jun. 2004, doi: 10.1001/jama.291.21.2547.

[60] Feleke DG, Ali A, Bisetegn H, Debash H, Birara W, Andualem A. Soil-transmitted helminthes and Schistosoma mansoni infections among primary school children at Ambasame primary school, North-West Ethiopia: a cross-sectional study. BMC pediatrics. 2022 Aug 5;22(1):477.

D. G. Feleke, A. Ali, H. Bisetegn, H. Debash, W. Birara, and A. Andualem, “Soil-transmitted helminthes and Schistosoma mansoni infections among primary school children at Ambasame primary school, North-West Ethiopia: a cross-sectional study,” *BMC Pediatr.*, vol. 22, no. 1, pp. 1–7, 2022, doi: 10.1186/s12887-022-03534-5.

[61] Forson AO, Arthur I, Ayeh-Kumi PF. The role of family size, employment and education of parents in the prevalence of intestinal parasitic infections in school children in Accra. PloS one. 2018 Feb 7;13(2):e0192303.

A. O. Forson, I. Arthur, and P. F. Ayeh-Kumi, “The role of family size, employment and education of parents in the prevalence of intestinal parasitic infections in school children in Accra,” *PLoS One*, vol. 13, no. 2, pp. 1–10, 2018, doi: 10.1371/journal.pone.0192303.

[62] Kihoro RW, Mulewa D, Okoyo C, Ayaa D, Korir A, Njomo DW, Mwandawiro C, Masaku J. Epidemiology of soil-transmitted helminthiasis among school-aged children in pastoralist communities of Kenya: A cross-sectional study. Plos one. 2024 May 23;19(5):e0304266.

R. W. Kihoro *et al.*, “Epidemiology of soil-transmitted helminthiasis among school-aged children in pastoralist communities of Kenya: A cross-sectional study,” *PLoS One*, vol. 19, no. 5 MAY, pp. 1–20, 2024, doi: 10.1371/journal.pone.0304266.

[63] Debalke S, Worku A, Jahur N, Mekonnen Z. Soil transmitted helminths and associated factors among schoolchildren in government and private primary school in Jimma Town, Southwest Ethiopia. Ethiopian journal of health sciences. 2013 Nov 11;23(3):237-44.

S. Debalke, A. Worku, N. Jahur, and Z. Mekonnen, “Soil transmitted helminths and associated factors among schoolchildren in government and private primary school in Jimma Town, Southwest Ethiopia,” *Ethiop. J. Health Sci.*, vol. 23, no. 3, pp. 237–244, 2013, doi:

10.4314/ejhs.v23i3.6.

[64] Genet A, Motbainor A, Samuel T, Azage M. Prevalence and associated factors of soil transmitted helminthiasis among school-age children in wetland and non-wetland areas of Blue Nile Basins, northwest Ethiopia: A community-based comparative study. SAGE Open Medicine. 2021 Dec;9:20503121211063354.

A. Genet, A. Motbainor, T. Samuel, and M. Azage, "Prevalence and associated factors of soil transmitted helminthiasis among school-age children in wetland and non-wetland areas of Blue Nile Basins, northwest Ethiopia: A community-based comparative study," *SAGE Open Med.*, vol. 9, 2021, doi: 10.1177/20503121211063354.

[65] Andargie Y, Alelign A, Tekeste Z. Prevalence and associated risk factors of soil-transmitted helminth infections among schoolchildren in Mekan Eyesus town, Northwestern Ethiopia. Parasite Epidemiology and Control. 2024 Nov 1;27:e00379.

Y. Andargie, A. Alelign, and Z. Tekeste, "Prevalence and associated risk factors of soil-transmitted helminth infections among schoolchildren in Mekan Eyesus town, northwestern Ethiopia," *Parasite Epidemiol. Control*, vol. 27, no. March, p. e00379, 2024, doi: 10.1016/j.parepi.2024.e00379.

[66] Novianty S, Dimyati Y, Pasaribu S, Pasaribu AP. Risk Factors for Soil-Transmitted Helminthiasis in Preschool Children Living in Farmland, North Sumatera, Indonesia. Journal of tropical medicine. 2018;2018(1):6706413.

S. Novianty, Y. Dimyati, S. Pasaribu, and A. P. Pasaribu, "Risk Factors for Soil-Transmitted Helminthiasis in Preschool Children Living in Farmland, North Sumatera, Indonesia," *J. Trop. Med.*, vol. 2018, 2018, doi: 10.1155/2018/6706413.

[67] Graeff-Teixeira C, Favero V, Pascoal VF, de Souza RP, de Vargas Rigo F, Agnese LH, et

al. Low specificity of point-of-care circulating cathodic antigen (POCCCA) diagnostic test in a non-endemic area for schistosomiasis mansoni in Brazil. Acta tropica. 2021 May 1;217:105863.

C. Graeff-Teixeira *et al.*, "Low specificity of point-of-care circulating cathodic antigen (POC[sbnd]CCA) diagnostic test in a non-endemic area for schistosomiasis mansoni in Brazil," *Acta Trop.*, vol. 217, p. 7, 2021, doi: 10.1016/j.actatropica.2021.105863.

[68] Homsana A, Odermatt P, Southisavath P, Yajima A, Sayasone S. Cross-reaction of POC-CCA urine test for detection of Schistosoma mekongi in Lao PDR: a cross-sectional study. Infectious diseases of poverty. 2020 Aug 1;9(04):66-74.

A. Homsana, P. Odermatt, P. Southisavath, A. Yajima, and S. Sayasone, "Cross-reaction of POC-CCA urine test for detection of Schistosoma mekongi in Lao PDR: A cross-sectional study," *Infect. Dis. Poverty*, vol. 9, no. 1, pp. 1–9, 2020, doi: 10.1186/s40249-020-00733-z.

69 Casagrande A, Fabris F, Girometti R. Beyond kappa: an informational index for diagnostic agreement in dichotomous and multivalue ordered-categorical ratings. Medical & Biological Engineering & Computing. 2020 Dec;58(12):3089-99.02261-2.

[69] A. Casagrande, F. Fabris, and R. Girometti, "Beyond kappa: an informational index for diagnostic agreement in dichotomous and multivalue ordered-categorical ratings," *Med. Biol. Eng. Comput.*, vol. 58, no. 12, pp. 3089–3099, 2020, doi: 10.1007/s11517-020-02261-2.

Abbreviation

Formatted: Heading 1, Left, Widow/Orphan control, Adjust space between Latin and Asian text, Adjust space between Asian text and numbers

AOR, adjusted odds ratio; CCA, circulating cathodic antigen; CI, Confidence interval; COR, Crude odds ratio; EPG, Eggs per gram; KK, Kato Katz; LR^+, Positive likelihood ratio; LR^-

, N likelihood ratio; MDA, Mass drug administration; NPV, Negative predictive value; NTD, neglected tropical disease; POC, Point of care; PPV, Positive predictive value; SOP, Standard operating procedure; STH, Soil-transmitted Helminth; WHO, World Health Organization, WASH, Water Sanitation and Hygiene, PC, preventive chemotherapy

Availability of data and materials

All relevant data are within the manuscript, and the datasets generated and/or analyzed during the current study are submitted.~~, subject to the approval of the Institutional Review Board of the Hawassa University College of Medicine and Health Sciences .~~ Restrictions apply to the availability of these data, which parents had consented for the collected information to be used for our research study only, and so are not publicly available.

PONE-D-25-23474R1

Assessment of Schistosoma mansoni and Soil-transmitted Helminth Infections and the Diagnostic Performance of the Circulating Cathodic Antigen Test among Schoolchildren in Tulla District, Sidama Region, Southern Ethiopia. PLOS ONE

Response to the PLoS ONE editor and reviewers' comments and questions

First and foremost, we sincerely thank you for reviewing our manuscript and offering valuable comments and suggestions. Your insights have been very helpful in highlighting areas for improvement, and we have carefully addressed each of the points you raised. We respond to each comment, suggestion and question raised by reviewers, and these responses are highlighted with green colour.

We thank you for your scientific contribution

Journal Requirements:

If the reviewer comments include a recommendation to cite specific previously published works, please review and evaluate these publications to determine whether they are relevant and should be cited.

Recommendation given by reviewer I to state background information if the two organisms are problems in the area under study. Information on ongoing interventions (in terms of number of rounds of treatment, Vector WASH services) on these infections should be mentioned and implications for the prevalence recorded in the present study should be investigated and reported.

We include the current global controlling strategies and background information on the prevalence of STH and Schistosoma in Ethiopia and in the current study area and control intervention measures at the national level and in the region of our study area in the introduction part from line number 118 to 147 which is highlighted in revised marked manuscript and we cite new references used for background information (reference numbers 14 – 20) in the reference section

Please review your reference list to ensure that it is complete and correct. If you have cited papers that have been retracted, please include the rationale for doing so in the manuscript text, or remove these references and replace them with relevant current references. Any changes to the reference list should be mentioned in the rebuttal letter that accompanies your revised manuscript. If you need to cite a retracted article, indicate the article's retracted status in the

References list and also include a citation and full reference for the retraction notice.

We cited references which are relevant to our manuscript. All references cited in this manuscript are revised to ensure its completeness and correctness to fit the PLOS ONE reference citation procedure; for this, it is highlighted in the revised marked references section

1. If the authors have adequately addressed your comments raised in a previous round of review and you feel that this manuscript is now acceptable for publication, you may indicate that here to bypass the "Comments to the Author" section, enter your conflict of interest statement in the "Confidential to Editor" section, and submit your "Accept" recommendation.

Reviewer #1: All comments have been addressed

Reviewer #3: (No Response)

Reviewer #4: (No Response)

We revised the manuscript to address the constructive comments and suggestions provided by the reviewers.   Thank you for your time and dedication

---

2. Is the manuscript technically sound, and do the data support the conclusions?

The manuscript must describe a technically sound piece of scientific research with data that supports the conclusions. Experiments must have been conducted rigorously, with appropriate controls, replication, and sample sizes. The conclusions must be drawn appropriately based on the data presented.

Reviewer #1: Yes

Reviewer #3: Yes

Reviewer #4: Partly

We revised the comments raised by the reviewer concerning this part and conclusion part is revised in advance

---

3. Has the statistical analysis been performed appropriately and rigorously?

Reviewer #1: Yes

Reviewer #3: No

Reviewer #4: Yes

We thank you all reviewers

In the revised manuscript, we clearly explain why we apply McNemar's test analysis and its purpose. We also explain the purpose of using logistic regression analysis which is indicated in data analysis section

---

4. Have the authors made all data underlying the findings in their manuscript fully available?

The PLOS Data policy requires authors to make all data underlying the findings described in their manuscript fully available without restriction, with rare exception (please refer to the Data Availability Statement in the manuscript PDF file). The data should be provided as part of the manuscript or its supporting information, or deposited to a public repository. For example, in addition to summary statistics, the data points behind means, medians and variance measures should be available. If there are restrictions on publicly sharing data—e.g. participant privacy or use of data from a third party—those must be specified.

Reviewer #1: Yes

Reviewer #3: Yes

Reviewer #4: Yes

We thank you all reviewers

---

5. Is the manuscript presented in an intelligible fashion and written in Standard English?

PLOS ONE does not copyedit accepted manuscripts, so the language in submitted articles must be clear, correct, and unambiguous. Any typographical or grammatical errors should be corrected at revision, so please note any specific errors here.

Reviewer #1: Yes

Reviewer #3: Yes

Reviewer #4: Yes

We thank all reviewers

6. Review Comments to the Author

Please use the space provided to explain your answers to the questions above. You may also include additional comments for the author, including concerns about dual publication, research ethics, or publication ethics. (Please upload your review as an attachment if it exceeds 20,000 characters)

Reviewer #1: The authors have adequately addressed the comments raised previously; however there few comments that need to be clarified in the current review. I found the manuscript technically sound and have value for policy and public health practice.

Thank you, Reviewer. We include the current global controlling strategies and background information on the prevalence of STH and Schistosoma in Ethiopia and in the current study area and control intervention measures at the national level and in the region of our study area in the introduction part from line number 118 to 147 which is highlighted in revised marked manuscript and we cited new references used for background information (reference numbers 14 – 20) in the reference section. We also added additional keywords significantly related to the study findings. We agreed to keep the research title as it is.

Reviewer #3: I advise the author(s) to take into account the following issues even if they made revisions to the text in response to the feedback and recommendations of earlier reviewers:
1. Uncorrected or Inaccurate Responses to Reviewers:
Ethical approval reference still unclear despite E.C. explanation; conversion not standardly recognized without explicit note.

Thank you, Reviewer, for your time and concern

In our initial submission, we uploaded the ethical clearance approval letter provided by the IRB of Hwassa University College of Medicine and Health Science, which was issued in 2024. So, I missed uploading the ethical clearance approval letter in the second submission. In the current revised manuscript submission, I have uploaded approval letter to the other files. The reference number provided on the ethical clearance application letter is not related to the

calendar. Instead, they are unique code number assigned to the ethical approval letter by the IRB. If you require confirmation, you can contact the IRB office at this email address: embialle@hu.ede.et

Data availability statement contradictory: claims data in SI, but no files uploaded; "available upon request" violates PLOS policy.

Raw data not publicly accessible; IRB restriction cited but not justified sufficiently for non-public sharing.

No supplementary files provided despite mention of questionnaire and raw data.

All data used for this manuscript have been submitted. We provided the raw data and questionnaire in a supplementary file during the second manuscript submission and we have no remaining data to submit. We have resubmitted the raw data and questionnaire in the supplementary files in the current revised manuscript submission.

Use of McNemar's test inappropriate for described analysis; original use of logistic regression more suitable, change unexplained.

We used SPSS regression analysis for descriptive analysis and for the significant association between risk factors and infection, whereas the McNemar chi-square test was employed to compare the statistically significant differences in sensitivity and specificity between the two diagnostic methods, focusing on cases where the tests disagree. This is clearly described in the data analysis section of this revised manuscript.

2. New Comments/Questions to Improve Quality:

Correct inconsistent verb tense usage (e.g., "data was analysed" → "data were analysed").

Subject-verb agreement is corrected in the revised manuscript

Clarify statistical methods: justify the switch from logistic regression to McNemar's test.

This one is described above; We used SPSS regression analysis for descriptive analysis and for the significant association between risk factors and infection, whereas the McNemar chi-square test was employed to compare the statistically significant differences in sensitivity and specificity between the two diagnostic methods, focusing on cases where the tests disagree. This is clearly described in the data analysis part of this revised manuscript.

Fix subject-verb agreement errors (e.g., "the results shows" → "the results show").

Subject-verb agreement is corrected in the revised manuscript

Provide DOI or repository link for raw data to comply with PLOS policy.

Our college is planning to develop a repository link; for this, we have submitted raw data in a supplementary file in this revised manuscript.

Include full ethical approval document with date conversion proof as supplementary.

This is described above. We uploaded the ethical clearance approval letter provided by the IRB of Hwassa University College of Medicine and Health Science, which was issued in 2024 in this revised manuscript. We address this issue in the ethical part. Ethical clearance was obtained from Hawassa University, College of Medicine, and the other Health Sciences Internal Review Board (IRB) with a reference number: IRB/172/16 in 2024. If you require confirmation, you can contact the IRB office at this email address.

Define the assent process clearly for children under 12 in methods

We explain the assent process clearly for children under 12 in methods as follow:

Explicit written consent was not obtained for children under 12 years, as they may lack the developmental capacity to understand this concept. However, these children were verbally informed about the study in simple language, and their willingness or unwillingness to participate was respected. This is included in the ethical clearance part

Update all WHO references to 2022 guidelines and complete missing citation details.

We revised not only the WHO references but all other references following the Vancouver citation style, following PLOS ONE guidelines, which are highlighted in marked revised manuscript.

WHO updated citation references include reference numbers 1 and 4 in the revised manuscript

Reviewer #4: Major Comments

1. What is your main objective? This might result in major revision of the whole

MS contents.
a. To assess the prevalence of S. mansoni and STHs (POC-CCA is additional)
b. To evaluate the diagnostic performance of KK vs POC-CCA (STH is additional)
c. ???

The objectives of this study are:

- To assess the prevalence of *S. mansoni* and STH infections.
- To assess risk factors for STH and S. manson infections
- To evaluate the intensity of *S. mansoni* infection
- To compare the diagnostic performance of POC-CCA test to that of KK. We clearly address these objective in the abstract and last paragraph of introduction section

2. Your result part is highly fragmented or not well organised: needs reshuffling.

We revised the results section and reorganized it to make the information clearer and more attractive for the reader.

3. What is the relevance of your study

Our study relevant to current WHO NTD control program as regional and local information are important assess ongoing controlling stratigies for the following reasons:

1. WHO has launched deworming programs for Schistosomiasis and soil-transmitted helminths targeting vulnerable school-age children? Although these deworming efforts have successfully reduced morbidity caused by Schistosoma and STH, re-infection continues in the absence of complementary measures to sustain deworming. This study is important for providing valuable supplementary interventions in the regional study area.

2. Intestinal schistosomiasis and soil-transmitted helminth infections are diagnosed by detecting parasite eggs in stool samples through microscopic methods, primarily using the Kato-Katz (KK) technique. Recommended by the WHO, this method is commonly used in epidemiological surveys.

However, the Kato-Katz technique has low sensitivity, making diagnosis difficult in areas with low prevalence and decreased worm loads. This difficulty results from several factors, including uneven egg distribution in stool samples, daily variations in egg excretion, and the random nature of egg dispersal. A more sensitive diagnostic method, called the POC-CCA test, has been developed to detect S. mansoni antigens in urine samples, especially useful in low-endemic areas, either as a replacement or supplement to the Kato-Katz technique. Nonetheless, the diagnostic accuracy of this method has not been thoroughly evaluated across different regions. This study is relevant to address this gap.

4. Your discussion is precise and not attractive to your readers.

We revised the discussion section and provided detailed descriptions of specific subtopics, which are highlighted in the marked revised manuscript. Additionally, we added subsections to improve readability (see from number line 563 – 606 in the marked revised manuscript)

Minor comments
Title
1. What do you mean by short title?

Short title is requested by PLoS ONE submission guidelines, which means writing the research title in a short sentence, keeping the main idea of the original research title
2. Check affiliation 3

We check, and it is correct
Abstract
1. A sentence but two different ideas. While S. mansoni infection leads to chronic illness and mortality, research on its intensity remains limited in the study area.'

We revised it and cleared the sentences in the current revised manuscript in the abstract part
2. Write acronyms of Point of Care Circulating Cathodic Antigen Test (POC-CCA) in its first appearance (in the background section)

We corrected acronyms

3. Modify your language throughout your entire document (e.g. a school-based cross-sectional study was conducted among 477 school children from April to June 2024 in Tulla district, Sidama Region, Southern Ethiopia).

Once again, we revised our manuscript throughout the document and marked the changes in the revised document.

4. Modify: Urine samples were also collected from the study participants and tested using POC-CCA for the detection of Schistosoma antigens.

We changed to urine samples were analyzed with POC-CCA technique.

5. Other helminths included … seams meaningless??

Yes, it may not be clear to all readers, so we removed others in the abstract part and replaced them with the name of the parasite species in the revised manuscript section

6. Avoid vague sentences: e.g. 'Moderate S. mansoni and STH infection and poor hygiene practice underscore for additional control measures.

Yes, we revised and cleared the sentence in the revised manuscript, which is highlighted in the marked manuscript

7. Your conclusion is not attractive.

We revised and rewrote the conclusion section (from the number line 747 – 760 in the revised marked manuscript

8. Reconsider your keywords (read about how to select keywords).

We considered and revised keywords in the revised manuscript

9. General comment: Critical language problems

We revised the manuscript once again to clear grammar errors and language issues, to eliminate specific errors and ambiguity. Any typographical or grammatical mistakes have been corrected during the revision

Introduction

Background

We deleted the word background and kept the Introduction in the revised manuscript

1. Put references in [..].

We put the reference number in [ ] in a manuscript word document

2. Paragraph 5 (line 106-111): It looks like you are talking about your own study

Thank you, we revised the paragraph (from line number 162 – 166) in the marked revised manuscript

3. The last paragraph: It is the main objective of your study, but not well structured, and I recommend re-write. Are you comfortable with 'to evaluate the prevalence…'? Also check your language (S. mansoni, S. mansoni).

Method

1. … among four governmental elementary school children (schools)

Corrected as: A school-based cross-sectional study was conducted from April to June 2024 among children in four governmental elementary schools in the Tulla district.

2 Add a sample collection portion and clearly indicate how you collected stool and urine samples from your study participants.

Stool and urine sample collection procedures are described in the manuscript from lines 244 – 253. Subtitle is given as Stool and Urine Sample Collection, in the revised marked manuscript

3. Infection intensities: You did categories (light, moderate and heavy) only for S. mansoni?

Yes, our research mainly focused on the prevalence, intensity, and performance of two diagnostic methods on S. mansoni. We only assess prevalence and associated risk factors of STH. Due to time constraints and resource limitations, we did not assess the intensity of STH

4. What is the relationship between quality and sample collection time?

Quality in a laboratory refers to its capacity to consistently produce accurate, reliable, and timely test results. The timing of sample collection is a crucial aspect of this quality, as it directly impacts the accuracy and reliability of the test outcomes. Furthermore, it is an important factor that is monitored throughout the entire testing process, from collection to interpretation. For instance, in our research context, maintaining proper timing for urine sample collection enhances the quality of the laboratory results.

5. Data analysis: indicate your reference test and give details about your analysis.

The data analysis and the reference test used for this study (the KK) are explained in the revised manuscript and highlighted in the marked manuscript from line number 287 to 315 in the analysis section

Results

1. I prefer if you put your results of diagnostics for S. mansoni and then to risk factors, STH,…

The authors agreed to put the order as follows: economic information, prevalence of S. mansoni, risk factors of S. mansoni, Intensity of S. mansoni, Prevalence of STH, risk factors of STH and finally, comparison of two diagnostic methods on S. mansoni detection

2. Table 1: move it under the risk factors sub-title.

We put Table 1 under risk factors

3. What is the importance of Table 2?

The importance of Table 2 is to show intensity variations across sex, age, and children's school attendance location

Response to the PLoS ONE editor and reviewers

First and foremost, we sincerely thank you for reviewing our manuscript and offering valuable comments and suggestions. Your insights have been very helpful in highlighting areas for improvement, and we have carefully addressed each of the points you raised.

1. Please ensure that your manuscript meets PLOS ONE's style requirements, including those for file naming. The PLOS ONE style templates can be found at

https://journals.plos.org/plosone/s/file?id=wjVg/PLOSOne_formatting_sample_main_body.pdf and

https://journals.plos.org/plosone/s/file?id=ba62/PLOSOne_formatting_sample_title_authors_affiliations.pdf.

We followed our manuscript to meet PLOS ONE's style requirements, including those for file naming.

2. PLOS requires an ORCID iD for the corresponding author in Editorial Manager on papers submitted after December 6th, 2016. Please ensure that you have an ORCID iD and that it is validated in Editorial Manager. To do this, go to 'Update my Information' (in the upper left-hand corner of the main menu), and click on the Fetch/Validate link next to the ORCID field. This will take you to the ORCID site and allow you to create a new iD or authenticate a pre-existing iD in Editorial Manager.

The corresponding author's ORCID ID is addressed

3. We note that your Data Availability Statement is currently as follows: [data are all contained within the manuscript and/or Supporting Information files, enter the following: All relevant data are within the manuscript and its Supporting Information files.]

Please confirm at this time whether or not your submission contains all raw data required to replicate the results of your study. Authors must share the "minimal data set" for their submission. PLOS defines the minimal data set to consist of the data required to replicate all study findings reported in the article, as well as related metadata and methods

We included raw data used for this manuscript results analysis

Reviewers' comments:

Reviewer's Responses to Questions

Comments to the Author

1. Is the manuscript technically sound, and do the data support the conclusions?

The manuscript must describe a technically sound piece of scientific research with data that supports the conclusions. Experiments must have been conducted rigorously, with appropriate controls, replication, and sample sizes. The conclusions must be drawn appropriately based on the data presented.

Reviewer #1: Yes

Reviewer #2: Yes

We thank all reviewers

---

2. Has the statistical analysis been performed appropriately and rigorously?

Reviewer #1: Yes

Reviewer #2: Yes

We thank all reviewers

---

3. Have the authors made all data underlying the findings in their manuscript fully available?

The PLOS Data policy requires authors to make all data underlying the findings described in their manuscript fully available without restriction, with rare exception (please refer to the Data Availability Statement in the manuscript

PDF file). The data should be provided as part of the manuscript or its supporting information, or deposited to a public repository. For example, in addition to summary statistics, the data points behind means, medians and variance measures should be available. If there are restrictions on publicly sharing data—e.g. participant privacy or use of data from a third party—those must be specified.

Reviewer #1: Yes

Reviewer #2: No

Thank you for your curiosity. We attached raw data and supplementary information, like the questionnaire

---

4. Is the manuscript presented in an intelligible fashion and written in Standard English?

PLOS ONE does not copyedit accepted manuscripts, so the language in submitted articles must be clear, correct, and unambiguous. Any typographical or grammatical errors should be corrected at revision, so please note any specific errors here.

Reviewer #1: Yes

Reviewer #2: No

We revised our manuscript once again for any topographical and grammatical corrections in the revised manuscript and indicated on the marked revised manuscript

---

5. Review Comments to the Author

Please use the space provided to explain your answers to the questions above. You may also include additional comments for the author, including concerns about dual publication, research ethics, or publication ethics. (Please upload your review as an attachment if it exceeds 20,000 characters)

Reviewer #1: The manuscript is well written, the methodology, results, statistical analysis and discussion were well presented. The manuscript is technically sound, and data provided in the manuscript. The statistical analysis

is appropriate. All findings were outlined in the manuscript. The manuscript is presented in Standard English.

The authors should address the comments made on the manuscript.

Reviewer #2: General comments
The manuscript presents a well-conducted cross-sectional study on the prevalence, intensity, and risk factors of Schistosoma mansoni and soil-transmitted helminth (STH) infections among schoolchildren in Ethiopia, alongside an evaluation of the diagnostic performance of the Point-of-Care Circulating Cathodic Antigen (POC-CCA) test. The study addresses an important public health issue in a region where these infections are endemic. The methodology is robust, and the findings are relevant to ongoing control efforts. However, there are several areas where the manuscript could be improved to enhance clarity, accuracy, and impact.
1. Ethical considerations: The manuscript states that ethical clearance was obtained from Hawassa University, but the reference number provided (IRB/172/16) appears outdated (2016). Given that the study was conducted in 2024, clarification or updated ethical approval documentation is necessary. Again, the consent process is described, but it would be beneficial to explicitly state whether assent was obtained from all children, including those under 12, or only those aged 12 and above.

We want to clarify that the ethical clearance reference number is not outdated; it is based on the Ethiopian calendar (E.C.). The reference number: IRB/172/16 E.C. corresponds to the Gregorian calendar date of 22/03/2024, which is indicated on the ethical clearance letter. We add E.C. to indicate the calendar, see the correction on line number 247 in the revised manuscript. Additionally, we acknowledge that our initial manuscript did not mention that assent was obtained from students aged ≥12 years following parent/guardian consent. This has now been addressed and appropriately revised in the manuscript. See the revised one from line number 250 – 252

2. Data availability: The manuscript claims that "all relevant data are within the manuscript and its Supporting Information files," but no supplementary files are provided. The authors should either upload the supporting data or clarify where it can be accessed.

Regarding the comment on the supplementary files, we have separately supplementary information. We also attached raw data

3. Diagnostic performance analysis: The comparison between POC-CCA and Kato-Katz is a key contribution, but the specificity of POC-CCA (79.6%) is notably low. The authors should discuss potential reasons for this (e.g., cross-reactivity, persistent antigens post-treatment) and implications for field use. Again, the McNemar's test result ($\chi^2 = 52.3$, $p < 0.001$) indicates significant disagreement between the two tests, but the discussion does not adequately address this. The authors should elaborate on the practical implications of this discrepancy.

Regarding the specificity of the POC-CCA test (79.6%), which is notably low, we were asked to discuss potential reasons and its implications for field use. We have addressed this from line numbers 516 – 540 in the revised manuscript as follows: **"However, the specificity of the POC-CCA test was found to be 79.6%, which is consistent with previously reported ranges but remains relatively low (20). This reduced specificity may be attributed to cross-reactivity with other helminth infections, the subjective interpretation of trace results as positive, and the continued excretion of CCA following recent treatment or in the absence of active infection (64). Such limitations can result in false positives, particularly in low-endemic or post-treatment settings, potentially leading to an overestimation of prevalence. Despite this, the POC-CCA test remains a practical diagnostic tool in field settings due to its operational simplicity, rapid results, and high sensitivity. However, its limited specificity underscores the importance of confirmatory testing, especially in surveillance, program evaluation, and low-transmission areas where diagnostic accuracy is critical."** Regarding the McNemar's test result ($\chi^2 = 52.3$, $p < 0.001$), we were informed that the discussion did not adequately address the observed discrepancy or elaborate on its practical implications. Therefore, we have addressed the issue as follows: **"Importantly, McNemar's**

test revealed a statistically significant discordance between the two methods ($\chi^2 = 52.3$, $p < 0.001$), driven primarily by the high number of POC-CCA positive but KK negative cases. This lack of alignment between the two diagnostic tools underscores their fundamentally different targets: POC-CCA detects active infection through CCA in urine, whereas KK relies on the detection of Schistosoma eggs in stool, which are subject to day-to-day variation and intra-sample inconsistencies (17). Therefore, in field settings, particularly in low-endemic or post-treatment areas, the POC-CCA test may identify infections that KK misses, making it a more sensitive tool for surveillance and monitoring of control programs."

4. Risk factor analysis: The association between STH infections and factors like latrine availability and handwashing is well-documented in the literature. The authors should contextualize their findings more critically, discussing how their results align with or diverge from previous studies in similar settings. Again, the multivariate analysis for S. mansoni risk factors is clear, but the odds ratios for some variables (e.g., swimming, irrigation) are high. The authors should consider potential confounding factors (e.g., frequency of water contact) that were not adjusted for.

As we were asked to critically analyse the association between STH infections and factors like latrine availability and hand washing, and to discuss how our results compare or differ from previous studies in similar settings, we addressed this in lines 464 –491 of the revised manuscript as follows:

- **The lack of a latrine at home was identified as a significant risk factor for soil-transmitted helminth (STH) infections in this study. This finding highlights the strong connection between poor sanitation and the transmission of helminths. It aligns with extensive research showing that limited access to sanitation facilities, especially at the household level,**

plays a major role in driving STH infections (49–51). Without access to latrines, many individuals are forced to practice open defecation, resulting in the direct release of helminth eggs into the environment and promoting continued transmission. Having access to household latrines significantly reduces the risk of exposure to infectious helminth stages in soil and water by minimizing the dispersion of feces in the surrounding area. Notably, This finding aligns with previous research in the same region, which also identified open field defecation as a key factor contributing to the prevalence of soil-transmitted helminths (STH) (22). This study reinforces existing evidence by examining sanitation status at the household level instead of relying on broader community-level indicators. By doing so, it provides a deeper understanding of how individual risks are influenced by domestic infrastructure. This highlights the need for targeted public health interventions that extend beyond general sanitation promotion. It is essential to prioritize equal access to latrines and ensure their sustained use at the household level, especially in high-transmission areas where environmental contamination continues to pose a significant challenge.

- **"Hand hygiene practices, especially the failure to wash hands after using the latrine, were significantly associated with the prevalence of STH, which aligns with prior global studies (52–54). This highlights the importance of hand washing as a key factor in preventing the transmission of STH (55). In contrast to previous research in the same area, hand hygiene showed no significant effect (22). This divergence may be due to variations in study design, changes in hygiene behaviors over time, or previous underestimations of individual practices. Furthermore, recent improvements in sanitation infrastructure, in the absence of corresponding behavioral changes, could increase the relative impact of hand hygiene on the risk of infection. These findings underscore the**

complex interaction between environmental and behavioral factors that influence the transmission of soil-transmitted helminths (STH) and emphasize the importance of ongoing local assessments.

- We were advised to consider potential confounding factors, such as frequency of water contact; however, this variable was not included in the final analysis as it was removed after the pretest due to participants' poor recall.

5. Prevalence and intensity: The reported prevalence of S. mansoni (20.1% by Kato-Katz) is described as "moderate," but the WHO threshold for moderate prevalence is ≥10% and <50%. The authors should explicitly state how their findings align with WHO guidelines for mass drug administration (MDA). Again, the intensity classification (light, moderate, heavy) is based on WHO criteria, but the geometric mean egg count (139.39 EPG) suggests a skewed distribution. The authors should discuss whether this affects the interpretation of intensity.

As we were asked to explicitly state how the 20.1% prevalence of S. mansoni aligns with WHO guidelines for MDA, we note that this has already been addressed in the discussion section of the previously submitted manuscript. We were also asked whether the interpretation of infection intensity was affected by the skewed distribution when using arithmetic and geometric means. we addressed this in lines 420 –423 of the revised manuscript as follows: "**The noticeable discrepancy between the arithmetic and geometric means reflects the presence of a right-skewed distribution of egg counts. This pattern is consistent with the typical epidemiology of schistosomiasis, where a minority of individuals harbor high worm burdens, while the majority exhibit low to moderate infection levels** (42)**.**

6. Limitations: The cross-sectional design and purposive school selection are noted as limitations, but the authors should also address potential biases (e.g.,

seasonal variations in transmission, non-random sampling). Again, the use of a single stool sample for Kato-Katz may underestimate prevalence due to day-to-day variation in egg excretion. This should be discussed as a limitation.

Regarding the comments on the limitations section of the manuscript, we addressed this in lines 551–554 in the revised manuscript as follows: "**This study has a few limitations: (i), the cross-sectional design provides a snapshot of *S. mansoni* and STH infection at a single point in time, which may not capture seasonal variations or fluctuations in infection rates over longer periods. (ii), the use of a single stool sample per participant for the KK technique may have led to underestimation of infection prevalence and intensity, given the known day-to-day variability in helminth egg excretion. (iii), the purposive selection of schools limits the generalizability of the findings to the larger community in the study area.**

7. Minor Comments: (i) Abstract: The abstract is comprehensive but could be more concise. The methods section, for example, includes unnecessary details (e.g., SPSS version). Again, the conclusion should avoid redundancy (e.g., "necessity for additional control measures" is repeated). (ii) Tables and Figures: Table 1 (infection intensity) is clear but could be merged with Table 2 (socio-demographic factors) to streamline the presentation. Again, Figure 3 (helminth distribution) is informative but would benefit from a clearer legend (e.g., full parasite names instead of abbreviations). (iii) Writing and Clarity: Some sentences are overly long or awkwardly phrased (e.g., "The absence of a latrine at home emerged as a prominent risk factor for STH infection"). Editing for conciseness would improve readability. Again, the abbreviation "STH" is used throughout but should be defined at first mention in the abstract and main text. (iv) References: References are generally appropriate, but some are outdated (e.g., WHO guidelines from 2011). The authors should cite more recent WHO

publications (e.g., 2022 guidelines). Again, a few references are incomplete (e.g., "Weekly epidemiological record Relevé épidémiologique hebdomadaire" lacks volume/issue details).

The minor comments have all been addressed in the revised manuscript, except for merging the tables, which we chose not to do because the current format is clearer and merging would make the presentation more complicated.

(i) Abstract: Data were processed in SPSS v26 using logistic regression, with p<0.05 considered statistically significant paraphrased to Data was analysed using SPSS and McNemar's chi-square test (line numbers 53 – 54). In conclusion, part necessity is deleted

(ii) We prefer to retain Table 1, which has been changed to Table 2 in the revised manuscript, as it aligns with the above explanation.
Figure 3 for helminthic distribution. We revised the figure and wrote the full name of the parasite rather than using the legend. See the revised Fig 3

(iii) Writing and Clarity: Once again, we revised the whole manuscript document for clarity, abbreviation consistency, and scientific parasite naming

(iv) We also revised the reference and made necessary corrections in the revised manuscript (line numbers 545 – 547)

While revising your submission, please upload your figure files to the Preflight Analysis and Conversion Engine (PACE) digital diagnostic tool, https://pacev2.apexcovantage.com/. PACE helps ensure that figures meet PLOS requirements. To use PACE, you must first register as a user. Registration is free. Then, login and navigate to the UPLOAD tab, where you will find detailed instructions on how to use the tool. If you encounter any issues or have any questions when using PACE, please email PLOS at figures@plos.org. Please note that Supporting Information files do not need this step.
