# Supplementary material for: Assessment of Schistosoma mansoni and soil-transmitted helminth infections and the diagnostic performance of the circulating cathodic antigen test among schoolchildren in Tulla district, Sidama region, Southern Ethiopia
Source: PLoS One. 2025 Dec 12;20(12):e0337919. doi: 10.1371/journal.pone.0337919 (PMC12700372; doi:10.1371/journal.pone.0337919)
Supplement: Attachment — Submitted filename: Response to editor and reviewrs comments and questions.docx [file pone.0337919.s007.docx]

**Response to the PLoS ONE editor and reviewers**

First and foremost, we sincerely thank you for reviewing our manuscript and offering valuable comments and suggestions. Your insights have been very helpful in highlighting areas for improvement, and we have carefully addressed each of the points you raised.

1. Please ensure that your manuscript meets PLOS ONE's style requirements, including those for file naming. The PLOS ONE style templates can be found at

<https://journals.plos.org/plosone/s/file?id=wjVg/PLOSOne_formatting_sample_main_body.pdf> and

[https://journals.plos.org/plosone/s/file?id=ba62/PLOSOne_formatting_sample_title_authors_affiliations.pdf](https://track.editorialmanager.com/CL0/https:%2F%2Fjournals.plos.org%2Fplosone%2Fs%2Ffile%3Fid=ba62%2FPLOSOne_formatting_sample_title_authors_affiliations.pdf/1/010f0197c0c92aa1-83196789-ea37-4515-9771-d34967cd572d-000000/yxJJXN7k4nGgrPMHHpp0wFHtqj4NdceZRTgqdG7N4XE=217).

We followed our manuscript to meet PLOS ONE's style requirements, including those for file naming.

2.  PLOS requires an ORCID iD for the corresponding author in Editorial Manager on papers submitted after December 6th, 2016. Please ensure that you have an ORCID iD and that it is validated in Editorial Manager. To do this, go to ‘Update my Information’ (in the upper left-hand corner of the main menu), and click on the Fetch/Validate link next to the ORCID field. This will take you to the ORCID site and allow you to create a new iD or authenticate a pre-existing iD in Editorial Manager.

The corresponding author's ORCID ID is addressed

3. We note that your Data Availability Statement is currently as follows: [data are all contained within the manuscript and/or Supporting Information files, enter the following: All relevant data are within the manuscript and its Supporting Information files.]

Please confirm at this time whether or not your submission contains all raw data required to replicate the results of your study. Authors must share the “minimal data set” for their submission. PLOS defines the minimal data set to consist of the data required to replicate all study findings reported in the article, as well as related metadata and methods

We included raw data used for this manuscript results analysis

Reviewers' comments:

Reviewer's Responses to Questions

**Comments to the Author**

1. Is the manuscript technically sound, and do the data support the conclusions?

The manuscript must describe a technically sound piece of scientific research with data that supports the conclusions. Experiments must have been conducted rigorously, with appropriate controls, replication, and sample sizes. The conclusions must be drawn appropriately based on the data presented.

Reviewer #1: Yes

Reviewer #2: Yes

We thank all reviewers

2. Has the statistical analysis been performed appropriately and rigorously?

Reviewer #1: Yes

Reviewer #2: Yes

We thank all reviewers

3. Have the authors made all data underlying the findings in their manuscript fully available?

The [PLOS Data policy](http://track.editorialmanager.com/CL0/http:%2F%2Fwww.plosone.org%2Fstatic%2Fpolicies.action%23sharing/1/010f0197c0c92aa1-83196789-ea37-4515-9771-d34967cd572d-000000/GaqFvMTwj8G3KA3RNYQCHsDakYW9KYZ2kLlQ83tL3D4=217) requires authors to make all data underlying the findings described in their manuscript fully available without restriction, with rare exception (please refer to the Data Availability Statement in the manuscript PDF file). The data should be provided as part of the manuscript or its supporting information, or deposited to a public repository. For example, in addition to summary statistics, the data points behind means, medians and variance measures should be available. If there are restrictions on publicly sharing data—e.g. participant privacy or use of data from a third party—those must be specified.

Reviewer #1: Yes

Reviewer #2: No

Thank you for your curiosity. We attached raw data and supplementary information, like the questionnaire

4. Is the manuscript presented in an intelligible fashion and written in Standard English?

PLOS ONE does not copyedit accepted manuscripts, so the language in submitted articles must be clear, correct, and unambiguous. Any typographical or grammatical errors should be corrected at revision, so please note any specific errors here.

Reviewer #1: Yes

Reviewer #2: No

We revised our manuscript once again for any topographical and grammatical corrections in the revised manuscript and indicated on the marked revised manuscript

5. Review Comments to the Author

Please use the space provided to explain your answers to the questions above. You may also include additional comments for the author, including concerns about dual publication, research ethics, or publication ethics. (Please upload your review as an attachment if it exceeds 20,000 characters)

Reviewer #1: The manuscript is well written, the methodology, results, statistical analysis and discussion were well presented. The manuscript is technically sound, and data provided in the manuscript. The statistical analysis is appropriate. All findings were outlined in the manuscript. The manuscript is presented in Standard English.

The authors should address the comments made on the manuscript.

Reviewer #2: General comments
The manuscript presents a well-conducted cross-sectional study on the prevalence, intensity, and risk factors of Schistosoma mansoni and soil-transmitted helminth (STH) infections among schoolchildren in Ethiopia, alongside an evaluation of the diagnostic performance of the Point-of-Care Circulating Cathodic Antigen (POC-CCA) test. The study addresses an important public health issue in a region where these infections are endemic. The methodology is robust, and the findings are relevant to ongoing control efforts. However, there are several areas where the manuscript could be improved to enhance clarity, accuracy, and impact.
1. Ethical considerations: The manuscript states that ethical clearance was obtained from Hawassa University, but the reference number provided (IRB/172/16) appears outdated (2016). Given that the study was conducted in 2024, clarification or updated ethical approval documentation is necessary. Again, the consent process is described, but it would be beneficial to explicitly state whether assent was obtained from all children, including those under 12, or only those aged 12 and above.

We want to clarify that the ethical clearance reference number is not outdated; it is based on the Ethiopian calendar (E.C.). The reference number: IRB/172/16 E.C. corresponds to the Gregorian calendar date of 22/03/2024, which is indicated on the ethical clearance letter. We add E.C. to indicate the calendar, see the correction on line number 247 in the revised manuscript. Additionally, we acknowledge that our initial manuscript did not mention that assent was obtained from students aged ≥12 years following parent/guardian consent. This has now been addressed and appropriately revised in the manuscript. See the revised one from line number 250 – 252

2. Data availability: The manuscript claims that "all relevant data are within the manuscript and its Supporting Information files," but no supplementary files are provided. The authors should either upload the supporting data or clarify where it can be accessed.

Regarding the comment on the supplementary files, we have separately supplementary information. We also attached raw data
3. Diagnostic performance analysis: The comparison between POC-CCA and Kato-Katz is a key contribution, but the specificity of POC-CCA (79.6%) is notably low. The authors should discuss potential reasons for this (e.g., cross-reactivity, persistent antigens post-treatment) and implications for field use. Again, the McNemar’s test result (χ² = 52.3, p < 0.001) indicates significant disagreement between the two tests, but the discussion does not adequately address this. The authors should elaborate on the practical implications of this discrepancy.

Regarding the specificity of the POC-CCA test (79.6%), which is notably low, we were asked to discuss potential reasons and its implications for field use. We have addressed this from line numbers 516 – 540 in the revised manuscript as follows: **“However, the specificity of the POC-CCA test was found to be 79.6%, which is consistent with previously reported ranges but remains relatively low (20). This reduced specificity may be attributed to cross-reactivity with other helminth infections, the subjective interpretation of trace results as positive, and the continued excretion of CCA following recent treatment or in the absence of active infection (64). Such limitations can result in false positives, particularly in low-endemic or post-treatment settings, potentially leading to an overestimation of prevalence. Despite this, the POC-CCA test remains a practical diagnostic tool in field settings due to its operational simplicity, rapid results, and high sensitivity. However, its limited specificity underscores the importance of confirmatory testing, especially in surveillance, program evaluation, and low-transmission areas where diagnostic accuracy is critical.”** Regarding the McNemar’s test result (χ² = 52.3, p < 0.001), we were informed that the discussion did not adequately address the observed discrepancy or elaborate on its practical implications. Therefore, we have addressed the issue as follows: **“Importantly, McNemar’s test revealed a statistically significant discordance between the two methods (χ² = 52.3, p < 0.001), driven primarily by the high number of POC-CCA positive but KK negative cases. This lack of alignment between the two diagnostic tools underscores their fundamentally different targets: POC-CCA detects active infection through CCA in urine, whereas KK relies on the detection of Schistosoma eggs in stool, which are subject to day-to-day variation and intra-sample inconsistencies (17). Therefore, in field settings, particularly in low-endemic or post-treatment areas, the POC-CCA test may identify infections that KK misses, making it a more sensitive tool for surveillance and monitoring of control programs.”**
4. Risk factor analysis: The association between STH infections and factors like latrine availability and handwashing is well-documented in the literature. The authors should contextualize their findings more critically, discussing how their results align with or diverge from previous studies in similar settings. Again, the multivariate analysis for S. mansoni risk factors is clear, but the odds ratios for some variables (e.g., swimming, irrigation) are high. The authors should consider potential confounding factors (e.g., frequency of water contact) that were not adjusted for.

As we were asked to critically analyse the association between STH infections and factors like latrine availability and hand washing, and to discuss how our results compare or differ from previous studies in similar settings, we addressed this in lines 464 –491 of the revised manuscript as follows:

- **The lack of a latrine at home was identified as a significant risk factor for soil-transmitted helminth (STH) infections in this study. This finding highlights the strong connection between poor sanitation and the transmission of helminths. It aligns with extensive research showing that limited access to sanitation facilities, especially at the household level, plays a major role in driving STH infections (49–51). Without access to latrines, many individuals are forced to practice open defecation, resulting in the direct release of helminth eggs into the environment and promoting continued transmission. Having access to household latrines significantly reduces the risk of exposure to infectious helminth stages in soil and water by minimizing the dispersion of feces in the surrounding area. Notably, This finding aligns with previous research in the same region, which also identified open field defecation as a key factor contributing to the prevalence of soil-transmitted helminths (STH) (22). This study reinforces existing evidence by examining sanitation status at the household level instead of relying on broader community-level indicators. By doing so, it provides a deeper understanding of how individual risks are influenced by domestic infrastructure. This highlights the need for targeted public health interventions that extend beyond general sanitation promotion. It is essential to prioritize equal access to latrines and ensure their sustained use at the household level, especially in high-transmission areas where environmental contamination continues to pose a significant challenge.**
- **“Hand hygiene practices, especially the failure to wash hands after using the latrine, were significantly associated with the prevalence of STH, which aligns with prior global studies (52–54). This highlights the importance of hand washing as a key factor in preventing the transmission of STH (55). In contrast to previous research in the same area, hand hygiene showed no significant effect (22). This divergence may be due to variations in study design, changes in hygiene behaviors over time, or previous underestimations of individual practices. Furthermore, recent improvements in sanitation infrastructure, in the absence of corresponding behavioral changes, could increase the relative impact of hand hygiene on the risk of infection. These findings underscore the complex interaction between environmental and behavioral factors that influence the transmission of soil-transmitted helminths (STH) and emphasize the importance of ongoing local assessments.**
- We were advised to consider potential confounding factors, such as frequency of water contact; however, this variable was not included in the final analysis as it was removed after the pretest due to participants’ poor recall.

5. Prevalence and intensity: The reported prevalence of S. mansoni (20.1% by Kato-Katz) is described as "moderate," but the WHO threshold for moderate prevalence is ≥10% and <50%. The authors should explicitly state how their findings align with WHO guidelines for mass drug administration (MDA). Again, the intensity classification (light, moderate, heavy) is based on WHO criteria, but the geometric mean egg count (139.39 EPG) suggests a skewed distribution. The authors should discuss whether this affects the interpretation of intensity.

As we were asked to explicitly state how the 20.1% prevalence of S. mansoni aligns with WHO guidelines for MDA, we note that this has already been addressed in the discussion section of the previously submitted manuscript. We were also asked whether the interpretation of infection intensity was affected by the skewed distribution when using arithmetic and geometric means. we addressed this in lines 420 –423 of the revised manuscript as follows: “**The noticeable discrepancy between the arithmetic and geometric means reflects the presence of a right-skewed distribution of egg counts. This pattern is consistent with the typical epidemiology of schistosomiasis, where a minority of individuals harbor high worm burdens, while the majority exhibit low to moderate infection levels** (42)**.**
6. Limitations: The cross-sectional design and purposive school selection are noted as limitations, but the authors should also address potential biases (e.g., seasonal variations in transmission, non-random sampling). Again, the use of a single stool sample for Kato-Katz may underestimate prevalence due to day-to-day variation in egg excretion. This should be discussed as a limitation.

Regarding the comments on the limitations section of the manuscript, we addressed this in lines 551–554 in the revised manuscript as follows: “**This study has a few limitations: (i), the cross-sectional design provides a snapshot of *S. mansoni* and STH infection at a single point in time, which may not capture seasonal variations or fluctuations in infection rates over longer periods. (ii), the use of a single stool sample per participant for the KK technique may have led to underestimation of infection prevalence and intensity, given the known day-to-day variability in helminth egg excretion. (iii), the purposive selection of schools limits the generalizability of the findings to the larger community in the study area.**

7. Minor Comments: (i) Abstract: The abstract is comprehensive but could be more concise. The methods section, for example, includes unnecessary details (e.g., SPSS version). Again, the conclusion should avoid redundancy (e.g., "necessity for additional control measures" is repeated). (ii) Tables and Figures: Table 1 (infection intensity) is clear but could be merged with Table 2 (socio-demographic factors) to streamline the presentation. Again, Figure 3 (helminth distribution) is informative but would benefit from a clearer legend (e.g., full parasite names instead of abbreviations). (iii) Writing and Clarity: Some sentences are overly long or awkwardly phrased (e.g., "The absence of a latrine at home emerged as a prominent risk factor for STH infection"). Editing for conciseness would improve readability. Again, the abbreviation "STH" is used throughout but should be defined at first mention in the abstract and main text. (iv) References: References are generally appropriate, but some are outdated (e.g., WHO guidelines from 2011). The authors should cite more recent WHO publications (e.g., 2022 guidelines). Again, a few references are incomplete (e.g., "Weekly epidemiological record Relevé épidémiologique hebdomadaire" lacks volume/issue details).

The minor comments have all been addressed in the revised manuscript, except for merging the tables, which we chose not to do because the current format is clearer and merging would make the presentation more complicated.

(i) Abstract: Data were processed in SPSS v26 using logistic regression, with p<0.05 considered statistically significant paraphrased to Data was analysed using SPSS and McNemar’s chi-square test (line numbers 53 – 54). In conclusion, part necessity is deleted
(ii) We prefer to retain Table 1, which has been changed to Table 2 in the revised manuscript, as it aligns with the above explanation.

Figure 3 for helminthic distribution. We revised the figure and wrote the full name of the parasite rather than using the legend. See the revised Fig 3

(iii) Writing and Clarity: Once again, we revised the whole manuscript document for clarity, abbreviation consistency, and scientific parasite naming
(iv) We also revised the reference and made necessary corrections in the revised manuscript ( line numbers 545 – 547)

While revising your submission, please upload your figure files to the Preflight Analysis and Conversion Engine (PACE) digital diagnostic tool, [https://pacev2.apexcovantage.com/](https://track.editorialmanager.com/CL0/https:%2F%2Fpacev2.apexcovantage.com%2F/1/010f0197c0c92aa1-83196789-ea37-4515-9771-d34967cd572d-000000/TZ-NtfuzHzaj78g6eXeJcIN19PZwo-QQpdIN7pvGAm8=217). PACE helps ensure that figures meet PLOS requirements. To use PACE, you must first register as a user. Registration is free. Then, login and navigate to the UPLOAD tab, where you will find detailed instructions on how to use the tool. If you encounter any issues or have any questions when using PACE, please email PLOS at [figures@plos.org](mailto:figures@plos.org). Please note that Supporting Information files do not need this step.
